# Novel human sex-typing strategies based on the autism candidate gene *NLGN4X* and its male-specific gametologue *NLGN4Y*

**DOI:** 10.1186/s13293-019-0279-x

**Published:** 2019-12-18

**Authors:** Stephan Maxeiner, Martina Sester, Gabriela Krasteva-Christ

**Affiliations:** 1 Institute of Anatomy and Cell Biology, Saarland University, Homburg, Germany; 2 Department of Transplant and Infection Immunology, Saarland University, Homburg, Germany

**Keywords:** Neuroligin-4, Amelogenin, Chromosomes, sex-typing, rhAMP genotyping

## Abstract

**Background:**

Since the early days of PCR techniques, sex identification, “sex-typing,” of genomic DNA samples has been a fundamental part of human forensic analysis but also in animal genetics aiming at strategic livestock breeding. Most analyses are employing the *AMELX/AMELY* gene loci on the X and Y chromosomes present in most mammals. We hypothesize that sex-typing in humans is also possible based on the genes *NLGN4X* and *NLGN4Y*, which represent X and Y chromosome-specific copies of a common ancestral *neuroligin-4* orthologue.

**Methods:**

Genomic DNA was isolated from human blood and buccal cell samples (total *n* = 111) and submitted to two different strategies: (a) a traditional two-primer PCR approach detecting an insertion/deletion (indel) polymorphism immediately upstream of the translational start on exon 1 and (b) detection of a single nucleotide polymorphism, SNP, on the translational stop carrying exon 7. The SNP detection was based on a quantitative PCR approach (rhAMP genotyping) employing DNA/RNA hybrid oligonucleotides that were blocked and which could only be activated upon perfect annealing to the target DNA sequence.

**Results:**

All indel PCR-tested human DNA samples showed two bands for males representing X- and Y-specific copies of *NLGN4* and a single band for female samples, i.e., homozygosity of *NLGN4X* and absence of *NLGN4Y*, in accordance with the self-reported sex of the donors. These results were in perfect agreement with the results of the rhAMP-based SNP-detection method: all males were consequently positive for both alleles, representing either SNP variant, and females were interpreted as homozygous regarding the SNP variant found in *NLGN4X*. Both methods have shown reliable and consistent results that enabled us to infer the sex of donor DNA samples across different ethnicities.

**Conclusions:**

These results indicate that the detection of human *NLGN4X/Y* is a suitable alternative to previously reported methods employing gene loci such as *AMELX/Y*. Furthermore, this is the first report applying successfully the rhAMP-genotyping strategy as a means for SNP-based sex-typing, which consequently will be applicable to other gene loci or different species as well.

## Background

A fundamental question in forensic sciences regarding the origin and identity of an unknown human DNA sample is whether it derives from a male or female donor. Since the identification of the *SRY* gene on the Y chromosome (sex-determining region on Y) [1–3], an essential distinguishing mark has been at hand as any other gene specific to the X chromosome is present in both, females and males. About 25 years ago, when testing for the presence of the *SRY* gene by PCR was technically novel to most research laboratories, the absence of a PCR product in any given reaction did not unequivocally indicate female origin since an internal control of the DNA quality and PCR performance was missing. This problem was eventually eliminated after two similar but not identical copies of the vertebrate *amelogenin* gene have been identified in mammals, which were named *AMELX* and *AMELY*, accordingly [4]. *AMELX*/*Y* are “gametologues” that have evolved early in mammalian (eutherian) evolution [5] and are localized on the X and Y chromosomes, respectively, just outside the pseudoautosomal region [6]. Sequence alignment of both genes revealed striking similarities but also single nucleotide polymorphisms as well as insertions or deletions (“indels”). Different combinations of primers have been designed hybridizing to highly similar or even identical regions. The resulting PCR product/s allow identifying indel variations by one reaction and, consequently, inferring the sex of the DNA sample: detection of *AMELX* serves as an internal control and the additional presence of *AMELY* concludes DNA origin of a male [7–9]. The *AMELX/Y* diversity as means of sex determination has been demonstrated in other mammalian species, such as dogs [10], horses [11], pigs [12], sheep [13], and many others. The identification of *AMELX* and *AMELY* genes has been widely accepted as means of testing for the presence of both sex chromosomes and is well established in forensic genetics and livestock breeding. In the light of the human genome project, potential alternative gene loci have been identified, such as the gene encoding for the zinc finger protein on X and Y (*ZFX/ZFY*) [14, 15], or predicted based on next-generation sequencing data [16].

Comparable to *amelogenin*, *neuroligin-4* also retains two separate copies on both human sex chromosomes, i.e., *NLGN4X* and *NLGN4Y* (cf. NCBI database). Therefore, this gene pair potentially qualifies for sex-typing as well. *Neuroligin-4* belongs to the neuroligin family of neural cell adhesion molecules, which are located at the postsynaptic side in neurons and interact trans-synaptically with neurexin proteins [17]. Mutations in several neuroligin genes have been identified to result in neurological disorders [17]. Most prominently mutations in the *NLGN3* and *NLGN4X* genes have been found to be underlying causes of autism spectrum disorders, ASD [18].

Considering human *NLGN4X/Y* as a suitable gene pair to identify the sex of unspecified donor DNA, we aimed at developing a simple protocol to detect a length polymorphism immediately upstream of the start codon applying standard PCR strategies available in many research labs. Many short nucleotide polymorphisms between both genes led us to consider rhAMP genotyping (IDT, Coralville, USA) as an alternative tool to distinguish both genes. This PCR-based strategy uses two “blocked” oligonucleotides, which incorporate a single ribonucleotide in a given deoxy-ribonucleotide sequence matching the single base discrepancies between different alleles [19]. RNaseH2-mediated cleavage of the correct RNA/DNA hybrid restores a functional 3-prime hydroxyl group upstream of this hybrid consequently allowing proper elongation by DNA polymerases. This method has been shown to be equally suitable or superior to other allelic discrimination technologies [20] and can also be used to detect alternatively spliced small exons [21].

Our study demonstrates that *NLGN4X/Y* gene detection is suitable for human sex determination across different ethnicities using two alternative experimental strategies. Besides conventional PCR-based strategies, this is the first report in which rhAMP genotyping has been applied to infer the sex of human DNA samples.

## Methods

### Bioinformatical analysis

The following genomic sequence information was retrieved from the NCBI database (www.ncbi.nlm.gov/gene), *NLGN4X*: *Homo sapiens* (Gene ID: 57502), *Gorilla gorilla* (101131855), *Pan troglodytes* (465474), *Pan paniscus* (100994646), *Pongo abelii* (100173414), and *NLGN4Y*: *Homo sapiens* (22829), *Gorilla gorilla* (Accession number: FJ532261.1), *Pan troglodytes* (Accession number: XM_009445767.3). For overview of annotated human *NLGN4X/Y* sequences, see Additional file 1. Sequences were aligned using MultAlin (http://multalin.toulouse.inra.fr/multalin) with default alignment settings for DNA analysis (“DNA-5-0”).

### DNA extraction

DNA was isolated from 105 retention blood samples from clinical routine diagnostics at the Department of Transplant and Infection Immunology, Saarland University, Germany, prior to discarding. To establish and optimize initial PCR conditions, we extracted DNA after the collection of buccal cells from six volunteering donors upon obtaining written informed consent and total anonymization of samples. Overview of sample ethnicity and sex distribution is summarized in Table 1. DNA sample preparation has been performed by two different methods based on the tissue sample, (a) buccal mucosa cells or (b) blood. Buccal mucosa cell samples were harvested from donors by collecting 40 ml of mineral water, which had been used in a thorough mouth rinse for 1 min. Cells and debris were collected at 4000×*g*, the supernatant was discarded, and the sediment was resuspended in 5 ml lysis buffer (0.1 M Tris, 5 mM EDTA, 0.2 M NaCl, 0.2% SDS, at pH 8.5), supplemented with 50 μl proteinase K-solution (20 mg/ml, Bioline, Luckenwalde, Germany), and incubated at 55 °C overnight. DNA extraction from buccal cell lysate was performed according to standard procedures using the phenol/chloroform extraction method. Genomic DNA from heparin/EDTA-blood samples was isolated using the Quick-DNA Miniprep Plus Kit (Zymo Research Europe GmbH, Freiburg, Germany). DNA concentration was quantified using a ND-1000 spectrophotometer (NanoDrop, Thermo Scientific, Waltham, MA, USA) and diluted to 5 ng/ml using IDTE-buffer (IDT, Coralville, Iowa, USA). All other chemical reagents have been purchased from Sigma-Aldrich (Darmstadt, Germany).

**Table 1:** Sex and ethnicity distribution. A total of *N* = 111 DNA samples were tested from male and female donors. Thirteen samples were from ethnicities other than Caucasian

Sex	*N* (total) = 111
Female	37
Male	74
Ethnicity	*N* (total) = 111
Afro-American	3
Asian	3
Caucasian	98
Indian	1
Middle Eastern	2
Sub-Saharan	4

### PCR analysis

The following primer combination has been used to identify an indel region (194 bp) immediately upstream of the respective start codons of the *NLGN4X* and *NLGN4Y* genes: MX17673 (sense) 5′-GAAGAGCCAGCCAGTGTTCTAGGTG-3′ and MX17674 (antisense) 5′-ACATGGTTCAAATCTGCATCCACATCC-3′. PCR reaction was performed according to the manufacturer’s recommendation using Q5 High-Fidelity 2x Master Mix (New England Biolabs GmbH, Frankfurt, Germany), 0.5 μM of each primer (MX17673/MX17674, IDT), and a total sample amount of 10 ng per run (reaction volume 25 μl). PCR reactions were loaded on a T100 Thermal Cycler (Bio-Rad Laboratories, Munich, Germany) using the following parameters: initial denaturation at 98 °C for 30 s, 35× cycle (98 °C for 10 s, 65 °C for 20 s, 72 °C for 30 s), and final extension at 72 °C for 1 min. Finally, PCR products were separated on a 2% agarose gel supplemented with MIDORI Green (1:10,000; Nippon Genetics Europe GmbH, Dueren, Germany) and documented using a Gel Doc System (Bio-Rad Laboratories).

### SNP genotyping

SNP genotyping has been performed using the rhAMP-genotyping strategy by IDT. Briefly, three potential base differences have been identified between both coding regions within the *NLGN4X* and *NLGN4Y* genes that appeared suitable to apply this strategy (Table 2). Potential primer combinations have been identified using the rhAMP-genotyping design tool (IDT). Fluorescent dye-conjugation (FAM or VIC) to and blocking specification of the respective oligonucleotides as well as further modifications to the alternative primers remained under the discretion of IDT. Each run consisted of 10 ng sample DNA (2 μl), 5.3 μl PCR mix (combined master and reporter mix), 0.5 μl rhAMP-SNP assay (20×), and 2.2 μl nuclease-free water. All qPCR reactions were run on a CFX Connect System (Bio-Rad Laboratories) with adjusted thermal cycling conditions: enzyme activation at 95 °C for 10 min and 40× cycle (95 °C for 10 s, 60 °C for 30 s, 68 °C for 20 s). The results of the individual runs were displayed as puncta on an XY scatter blot based on relative fluorescent units (RFU values) suited for allelic discrimination (CFX Connect System software, Bio-Rad Laboratories).

**Table 2:** rhAMP-SNP-assay sequences. Each rhAMP-assay consists of a set of allele-specific primers to distinguish between both alleles as well as a locus-specific primer common to both allelic variants. 5-prime modifications to each allele-specific primer regarding FAM/VIC-based fluorescence detection and 3-prime “blocking” immediately downstream of the given ribonucleotide remain subject to the vendor’s discretion (IDT)

Assay name	Allele-specific primer 1	Allele-specific primer 2	Locus-specific primer
SNP_A	CCCCGAGCCAAAGATG	CCCCCGAGCCAAAGATA	GCGGATTGAGGAGAATGTGGGA
SNP_B	ATTCAGAGCGGCACC	CATTCAGAGCGGCACT	GCGACCTTGTCTGCCAATATCC
SNP_C	AGAGAAACACCACAAATGATATCG	CAGAGAAACACCACAAATGATATCA	GCATCGTGTTCCAGCTGCTT

## Results

### Identification of indels and SNPs in the NLGN4X/Y genes

**Fig. 1:**
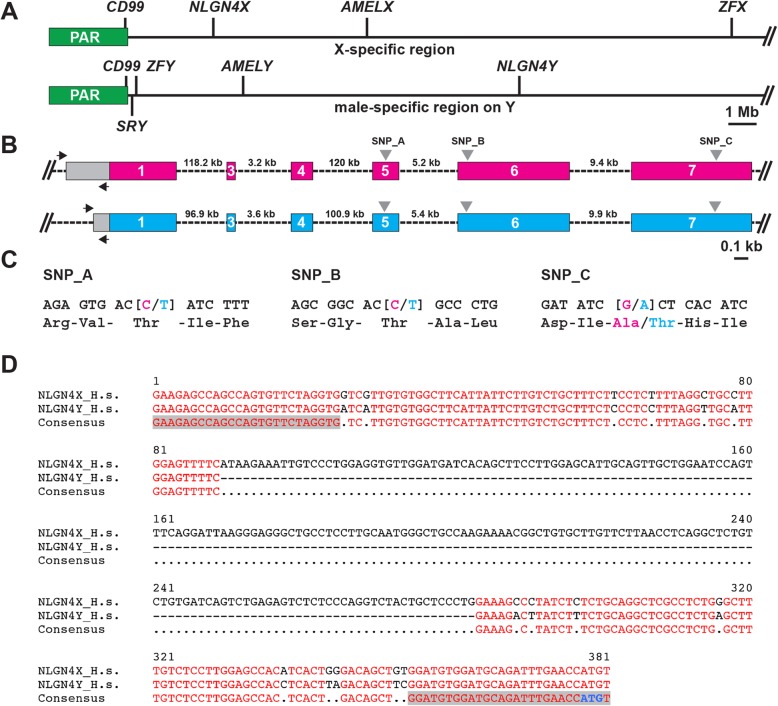
*NLGN4X/Y* gene overview and assay location. **a** Schematic encompassing the pseudoautosomal region, PAR (green boxed area), as well as the X-specific region on the X chromosome and the male-specific region on the Y chromosome. *CD99* is the most proximal, first gene located in the PAR common to both sex chromosomes. The *SRY* gene (unique to the Y chromosome) is immediately located at the pseudoautosomal boundary within the male-specific region. Gametologous copies of the *NLGN4X/Y*, *AMELX/Y*, and *ZFX/Y* genes are depicted according to their relative positions on the X-specific and male-specific region. **b** Enlargement of the respective *NLGN4X* (magenta) and *NLGN4Y* genes (blue). For immediate comparison, only the protein coding exons are depicted. Both genes share an identical structure except for the size of the untranslated region on exon 1 (grey boxes). All exons are drawn to scale, whereas dashed lines between two neighboring exons reflect varying intron sizes with the respective sizes labeled on top. Exons are numbered according to the general assignment of exons of the neuroligin family [22]; generally, *NLGN4* genes formally lack exon 2. Arrows flanking the 5-prime untranslated region on exon 1 represent relative primer localization to identify the indel variation allowing the discrimination of both genes. Grey arrowheads point to the relative position of three potential single nucleotide polymorphisms, SNPs. **c** Relative position of all three SNPs (A–C) within the coding region. SNP_A and SNP_B are synonymous changes; SNP_C is a non-synonymous change resulting in amino acid differences between NLGN4X and NLGN4Y proteins. **d** Sequence alignment of *NLGN4X/Y* PCR amplicons. Priming oligonucleotides are highlighted in grey with the start codon in blue. Identical bases are shown in red, mismatches in black

To validate our hypothesis that the gene pair *NLGN4X* and *NLGN4Y* could be employed to distinguish male and female DNA samples, we iteratively compared the available genomic sequence information for both genes encompassing all protein coding exons. The general numbering of exons regarding all four *neuroligin* genes is based on a previous publication by Bolliger [22], which in case for *neuroligin-4* results in the formal absence of exon 2 (Fig. 1a). Both genes are covering approx. 200 kb displaying their highest similarity in the vicinity of and within their exons. In order to develop a simple PCR strategy to discriminate both genes, two fundamental prerequisites needed to be met, firstly, the oligonucleotides should not contain ambiguous bases to accommodate identical annealing conditions for either gene, and secondly, both amplicons must be readily distinguishable after a short separation time using agarose gel electrophoresis. The validation of a SNP-based strategy by rhAMP genotyping meets different criteria (see the manufacturer’s recommendation). To distinguish both human *NLGN4* genes (“alleles”), we chose stretches in which both genes were identical except for a single base difference. Three potential loci have been identified in exon 5, 6, and 7, respectively, complying with this prerequisite (SNP_A, B, and C in Fig. 1b, c). Whereas SNP_A and B represent loci with synonymous base changes, the base difference between *NLGN4X* and *NLGN4Y* in SNP_C results in a change from alanine (*NLGN4X*) to threonine (*NLGN4Y*) (Fig. 1c).

### PCR detection of an indel variation upstream of the start codon

**Fig. 2:**
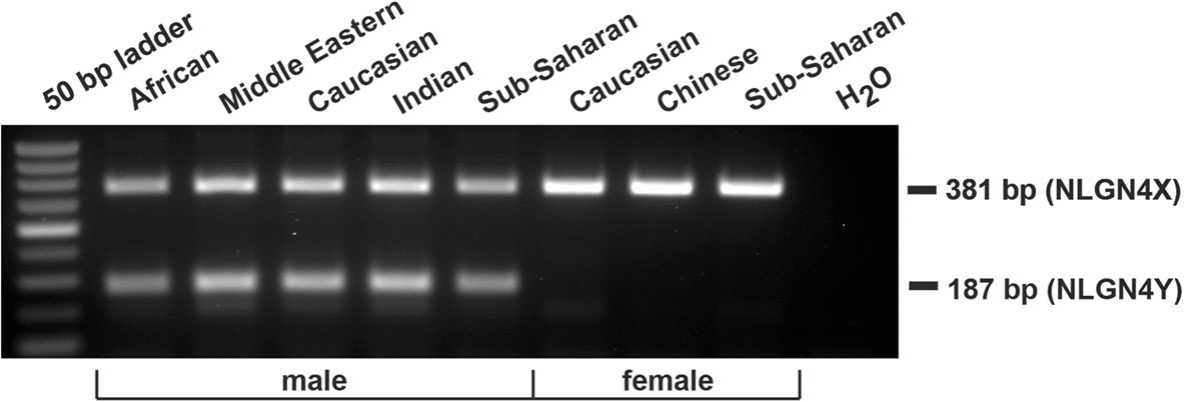
PCR-based *NLGN4X/Y* sex-typing strategy. Representative picture showing PCR results separated on an agarose gel testing for an indel polymorphism between both *NLGN4* gametologues. Male donor DNA of different ethnicities resulted consistently in two bands indicated by the presence of *NLGN4X* (381 bp) and *NLGN4Y* (187 bp). Female donor DNA resulted only in a single band (*NLGN4X*)

In an initial screening of several potentially suitable indel regions using different sets of oligonucleotides, we found one pair that robustly distinguished a region immediately upstream of the start codon (Fig. 1d). Human genomic DNA isolated from buccal cells as well as from human blood samples were submitted to PCR reaction. In the case for *NLGN4X*, a longer amplicon (381 bp) was expected than for *NLGN4Y* (187 bp) (Fig. 2). Theory has it that the presence of both bands represent male donor DNA, and a single band reflects female donor DNA serving as a control for PCR performance. Without any exception, all tested samples (*N* = 111) showed PCR results that were matching the self-reported sex of their donors across different ethnicities (74 males and 37 females, Table 1; Additional file 2). A comparison to sequence information available from other hominid genomes suggests that this PCR-based strategy should also apply to chimpanzee DNA (*Pan troglodytes*, Table 3).

**Table 3:** Sex-typing strategies applicable to hominids. Overview of several primate species closely related to humans. Humans and chimpanzees share the same indel confirmed by available sequence information. The rhAMP strategy is applicable in humans and western gorillas for the given SNP

	PCR genotyping (predicted amplicon size)		rhAMP-genotyping (base at SNP_C position)	
Species	*NLGN4X*	*NLGN4Y*	*NLGN4X*	*NLGN4Y*
*Homo sapiens*	381 bp	187 bp	G	A
*Gorilla gorilla* (western gorilla)	381 bp	n/a	G	A
*Pan troglodytes* (chimpanzee)	381 bp	187 bp	G	C
*Pan paniscus* (pygmy chimpanzee)	381 bp	n/a	G	n/a
*Pongo abelii* (Sumatran orangutan)	377 bp	n/a	G	n/a

### rhAMP genotyping of NLGN4X/Y SNPs

**Fig. 3:**
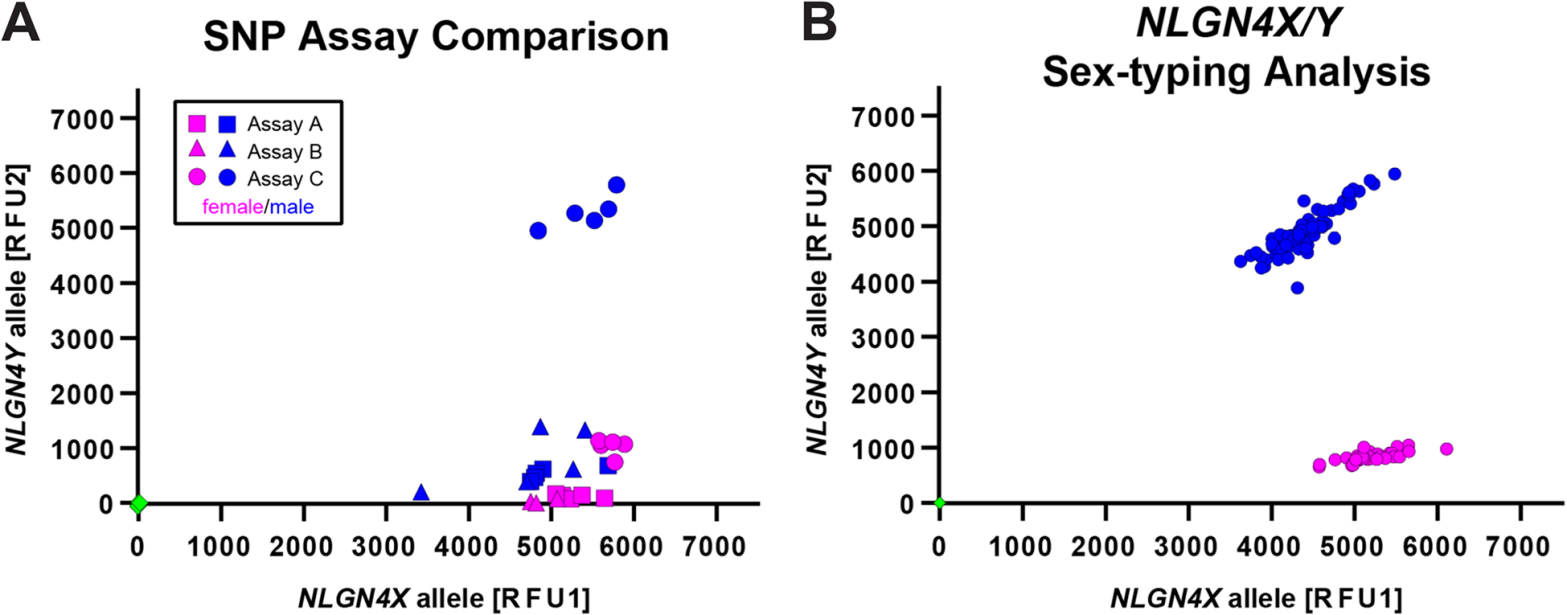
Sex-typing strategies based on *NLGN4X/Y* gene polymorphisms. **a** Allelic discrimination patterns based on rhAMP-SNP-detection strategies using SNP assays A (squares), B (triangles), and C (circles). Results for male donor DNA are depicted in blue, for females in magenta. Each assay was run on the identical set of male and female donor DNA samples (*N* = 5 each). Detection values of the respective “alleles,” i.e., *NLGN4X/Y* gametologues, are displayed in relative fluorescent units (RFU) calculated by the thermo cycler software. The performance result of assay C is the only one out-grouping male donors from female donors. **b** A total of *N* = 111 male and female donor DNA samples were subjected to rhAMP-analysis using the SNP_C assay. Results for male (blue) and female (magenta) donor DNA separated consistently (100%) and matched with self-reported sex identification of donors

Since *NLGN4X* and *NLGN4Y* retain high homology in their coding sequence (96.9%), we regarded single nucleotide polymorphisms as “pseudo-allelic variations” and, therefore, considered the rhAMP-genotyping system as potentially suitable means to verify the presence of either gene in order to infer the sex of the donor. As a proof of principle, we tested three different assays (one for each SNP) using five male and female samples that had been validated by PCR (Fig. 3a). Only the assay detecting the non-synonymous base change (SNP_C, see Fig. 1c and Table 2) resulted in a proper allelic separation of both genes reminiscent of previous publications [20]. Assays A and B detected solely *NLGN4X* but failed to detect *NLGN4Y*. Subsequently, we submitted our set of donor DNA to rhAMP genotyping using assay SNP_C and found that all samples (*N* = 111) separated in two non-overlapping clusters suggesting the “allelic” combinations *NLGN4X*/*NLGN4X* (female) and *NLGN4X*/*NLGN4Y* (male) (Fig. 3b; Additional files 3 and 4). The results were identical to our previous results using conventional PCR to detect an indel polymorphism upstream of the start codon. In silico analysis revealed that this particular rhAMP-based allelic discrimination should also be applicable to sex-type western gorillas but not chimpanzees (Table 3).

## Discussion

Sex identification based on DNA screening receives broad interest not only regarding forensic sciences but also in animal breeding, e.g., in horse breeding [23]. Sole detection of the male-specific *SRY* gene by PCR, however, is lacking an internal positive control to judge PCR performance or DNA quality. PCR protocols based on indel variations in the *amelogenin* genes are widely accepted, because X and Y chromosome-specific versions are present in many eutherian genomes. Since *AMELX* and *AMELY* have deviated from a common ancestral gene [6], we hypothesized that due to shifts of the boundaries of the pseudoautosomal region [24], similar indel polymorphisms might be present in the human *neuroligin-4* genes (*NLGN4X/Y*), with their highly similar copies located on both human sex chromosomes. We aimed at developing a simple lab protocol based on PCR to identify an indel variation immediately upstream of the start codon. This yielded robust results, which in all cases tested were matching the sexes specified by their donors properly. Subsequently, three potential SNP-based assays using rhAMP-genotyping strategies were tested detecting differences in three coding exons (exons 5, 6, and 7) roughly 200 kb downstream of the above verified indel variation. Only one assay, which was based on a non-synonymous base change between both genes on exon 7, showed the anticipated separation of two groups according to our hypothesis. In all samples, the rhAMP-genotyping strategy confirmed our results from PCR-based typing. Several reasons might explain the poor assay performance regarding SNP_A and B. These assays were based on synonymous base changes in the third position in both genes. Since both assays indicated the presence of *NLGN4X*, it might be possible that the bases in the respective *NLGN4Y* gene do not correspond to the ones present in the current draft of the human genome sequence. The donor material on which the human genome project is based derives from a small number of donors with unspecified ethnicity [25]. It might be possible that these bases are found in particular ethnic groups, which are not covered by our analysis. This inability to clearly define the sex, however, further strengthens the results that we obtained assaying the non-synonymous base change in exon 7 (SNP_C assay). In our small but diverse group of ethnicities (Table 1), both genes had been detected consistently within the male cohort classifying this “allelic” polymorphism as a valid distinguishing marker between *NLGN4X* and *NLGN4Y*.

Similar to current assays employing techniques to detect indel variations and polymorphisms in *AMELX/Y* [7–9], *ZFX/Y* [14, 15], or hypothesized human sex chromosome genes [16], *NLGN4X/Y* is primarily suitable to discriminate both genes commonly found in 46,XX or 46,XY pairs. At this point, we cannot predict the performance of these assays in rare cases of aneuploidy found in Turner Syndrome (45,X), Klinefelter Syndrome (47,XXY), double Y males (47,XYY), or triple X females (47,XXX) [26]. Furthermore, genomic rearrangements such as men with a translocated *SRY* gene, e.g., in cases of 46,XX sex reversal [27] or deletions including *NLGN4X* [28, 29], will affect the interpretation of the data regarding the PCR as well as the SNP-based strategy. Aneuploidy, mutations, deletions, and translocations, however, are considerably rare and are likely challenging any two-gene-based sex-typing strategy including tests regarding *AMELX/Y*.

Although rhAMP-genotyping strategies have previously been applied for allelic discrimination [20] or quantitative assessment of alternative splicing [21], our study is the first in which this approach was implemented to infer the sex of donor DNA samples based on the presence or absence of sex chromosome-specific gene loci. This methodical approach should not be exclusive to the *NLGN4X/Y* gene pair, but might also be applicable to distinguish *AMELX/Y*, *ZFX/Y*, or any other suitable gene pair depending on the designing criteria.

It appears that the close relationship of humans to other hominids suggests that at least one sex-typing approach (PCR or rhAMP-based) might directly be applicable to other hominids without changing any of the parameters that we have established for human DNA samples. However, due to the absence of complementary genomic information for all *NLGN4Y* genes (e.g., gorilla, pygmy chimpanzee, and Sumatran orangutan), a conclusive statement regarding the application of both protocols seeks further validation.

During the course of our study on the validation of *NLGN4X/Y* as a candidate gene pair for sex-typing applications, Jeong et al. have analyzed next-generation sequencing data from public databases and proposed several genes including *NLGN4X/Y* [16]. The distinguishing features are based on short sequence segments found in transcripts and remain solely a bioinformatical strategy comparing NGS data sets. In contrast, both of our protocols have been validated in over one hundred donor samples and are feasible in a research lab depending on sample numbers and equipment. The PCR-based indel detection is a considerable cheap approach for smaller sample numbers, whereas the rhAMP-based strategy allows high-throughput screening and could be combined with multiplexing PCR-technologies used in forensic sciences.

Primarily, we consider this study to be a proof of principle to successfully infer the sex based on the detection of *NLGN4X* and *NLGN4Y* genes. The use of this gene pair, however, is not per se superior to the detection of other gene pairs; we believe that it might be an alternative and/or complementation to previously reported gene loci. While human *AMELY* and *ZFY* are located more closely to the *SRY* gene and the pseudoautosomal boundary, *NLGN4Y* is located further away (approx. 14 Mb, Fig. 1a), and therefore, its detection basically confirms the presence of a different segment of the Y chromosome.

Based on the notion that *NLGN4X* and *NLGN4Y* are highly similar genes and located separately on the human sex chromosomes, we have established two protocols to detect both genes based on an indel variation upstream of the start codon or a single nucleotide difference in exon 7. Highly consistent results in sex determination in all human DNA samples originating from different ethnicities by both strategies are establishing *NLGN4X*/*Y* sex-typing as a novel alternative to previously reported strategies and employed gene loci.

## Perspectives and significance

In summary, our results introduce the human *NLGN4X/Y* gene pair as a basis for alternative strategies determining the presence of the X and Y chromosomes due to their shared evolutionary history. Testing for *NLGN4X/Y* is not just a mere addition to an exclusive club of selected genes, such as *AMELX/Y* or *ZFX/Y*; in just a single report, we suggest two different protocols encompassing both length and single nucleotide polymorphism for human sex determination. To our knowledge, this is the first report that uses rhAMP genotyping with blocked oligonucleotides to determine SNP polymorphisms allowing the inference of the sex from unspecified human donor material.

## Supplementary information

**Additional file 1. **Annotated human *NLGN4X* and *NLGN4Y* sequences

**Additional file 2.** PCR results of testing for the indel polymorphism

**Additional file 3.** Quantitative PCR-results on SNP_C

**Additional file 4.** Alignment for SNP_C

## Data Availability

The dataset(s) supporting the conclusions of this article are included within the article and its additional files.
