# Supplementary material for: Novel human sex-typing strategies based on the autism candidate gene NLGN4X and its male-specific gametologue NLGN4Y
Source: Biol Sex Differ. 2019 Dec 18;10:62. doi: 10.1186/s13293-019-0279-x (PMC6921425; doi:10.1186/s13293-019-0279-x)
Supplement: Supplementary file 1 — Additional file 1. Annotated human NLGN4X and NLGN4Y sequences [file 13293_2019_279_MOESM1_ESM.docx]

Primer annealing sites are highlighted in red;

Exons are highlighted in yellow

rhAMP-assay SNPs are highlighted in magenta

>NLGN4X_Homo_sapiens_GeneID:57502

GAATTTGTGTCCAAAGTCTACACTCTTTTCATTTGATGATGTTCCCTTTGTGGCCTGATAAATATCCACATCATGATGCCAGATTGACTTGGATGCATGCTTCCATCTTTCTCCTACTGGAAAACTTTTAGAGCTCCATGCATGTCTCCTTAGGAAAATGTGACAATTTCCTTAAACATTTGAGAAACAGTGTTTTGGAAGTACCCATGTATTGATAACCAGTCTGGTAAACAATAGCAAAACTGGGAGGTGTTGTTACTATAATCTGCATAACCTGTATAACTCTTGAACATCTGTTTGATCATTCAACACAGATTTGTTTAGTGTTTTCTAAATGTCAGGCATTGTTCATGGTGATAGGATGTACAGAGGAATTAAGACAAGTGGTGGCTGCTAGGCATGGTGACTCATGCCTGTAATCCCAACACTTTGAAAGGTCGAGGGGTAGGATCCCTTGAGGCCAGCCTGGACAACATAGGGTGACCCAATGTCTACAAAAAAATCCAACGAATTAGCCGGACATAGTGGTGCATGCTTGTGGTCCCAGCTACTCGGGAGGGTGAGGCGGGAGGATGGGTTGAGCCCAGGAGTTGGAGGCTGCAGTGAGCTATGACAGCACCACTGCACTGCAGCTTGGGCAATATAGCAAGACACCATCTCTAAAAAAAACAAAATAAATAAAGACAGGTGATGTTCTTGCTGTTGCCTACTATGTGGAGATGGCACTATACACATTTCTATACAAATGAATAGGAATTTCATAGAGAGATGTTGTGGATTTCGTGGAAGAGCCAGCCAGTGTTCTAGGTGGTCGTTGTGTGGCTTCATTATTCTTGTCTGCTTTCTTCCTCTTTTAGGCTGCCTTGGAGTTTTCATAAGAAATTGTCCCTGGAGGTGTTGGATGATCACAGCTTCCTTGGAGCATTGCAGTTGCTGGAATCCAGTTTCAGGATTAAGGGAGGGCTGCCTCCTTGCAATGGGCTGCCAAGAAAACGGCTGTGCTTGTTCTTAACCTCAGGCTCTGTCTGTGATCAGTCTGAGAGTCTCTCCCAGGTCTACTGCTCCCTGGAAAGCCCTATCTCTCTGCAGGCTCGCCTCTGGGCTTTGTCTCCTTGGAGCCACATCACTGGGACAGCTGTGGATGTGGATGCAGATTTGAACCATGTCACGGCCCCAGGGACTGCTATGGCTTCCTTTGTTGTTCACCCCGGTCTGCGTCATGTTAAACTCCAATGTCCTCCTGTGGTTAACTGCTCTTGCCATCAAGTTCACCCTCATTGACAGCCAAGCACAGTATCCAGTTGTCAACACAAATTATGGCAAAATCCGGGGCCTAAGAACACCGTTACCCAATGAGATCTTGGGTCCAGTGGAGCAGTACTTAGGGGTCCCCTATGCCTCACCCCCCACTGGAGAGAGGCGGTTTCAGCCCCCAGAACCCCCGTCCTCCTGGACTGGCATCCGAAATACTACTCAGTTTGCTGCTGTGTGCCCCCAGCACCTGGATGAGAGATCCTTACTGCATGACATGCTGCCCATCTGGTTTACCGCCAATTTGGATACTTTGATGACCTATGTTCAAGATCAAAATGAAGACTGCCTTTACTTAAACATCTACGTGCCCACGGAAGATGGTGAGTACCTCACTGGAACAGAAAACAATACCTCTTGTGCAGTGTGTAGAGAGATTTGCTAGGAGGGTTTTATAATGTCTCATGCATGATCTCTTCTATAACCCGTTTATTTTATTTTAATTTATTTTTCATATTCCAAATGCAATTCTTGCAGCAACTTACCACATGTTCCACTTGTATGTATTGGGCCATCTACTGACTGGACAAAACTATAAATAATAACTTTAATTATTTTCATATATTGCCTTCTTAACTTTTTATAATGCTTATTTGCAGATGAAAATAAATATGAGCATATAATGTTGCATGTTATACCTGAATCATCTGTAAAGGAATGAATCTATAGAAAAATAATAGAATTAAGTACACTATTATGCTCCAGTTTGCAAACTGAAAGATAGAGAAAATGGTTCTTTCTGCCTTAATGACTTAAGATATTAGCACCTTTTTTGAGTTTTCAAAGAAAAACTTGATTGTTTTTAATATACAAGTAGGGGATAGTTCATACAATGGTTGGATTTCATTGTTTAGAATCGGTTTTCTTAACGTAAATTTGGATGTTCTTTTCTTCCAATATTCGCTGCAATCAAGTGGCAAAATGTAATCAGATGATTCTAGCTACATTAGAGATGAATGCGTTTGTATTTTTAAAAATTTCCTTTTTTATATAAAACAACAATGAAAGTCTGTAGACACAATAACGTTTAATATATTAACCTAATGTTAGTAAAACATGAATAGTTTTATGTCTGTATAGATTTCAAATTCAGATTTCCTTGGAAGAATAACCAGACTAAAGTATGCCATAATGGTATCACATTTCCCAGTTAGCATTTCCATATGCCGTTTTTAGATGAGGAGAAAGAACAACAGAGAATAAAATATACCTGGAAAGAAAGGAAGTTAATTTGTGGGAATGATAGATGTATCTAATGTAGAAACTAGAGTGTGTCCTTTGTATAAAGTTCTTCGTGGAAAGTGTGATAAATTTCTTTTATGGAGAAATTTCTTCTTCTTCTTTTTTTTTTTTTTTAAACTTCAATCCCTGGAAAACATTTTTCAGTAAGATTTGGCTGAAAATAGTAAATCAACAACGACGTTAATCCACTGATCTCCAAAATTGTTTTGCATCTATCAGATTACTCTTTCTCCATATAAATGCCAGATAGTTTAAGTAGAGTGTCATGAAAAACCATACCAGGGTTGTGTGTCACTGAGGTTACAAATTGTCATTGAGATTACAAAGAACAGCCCAGAGAAAGAAATTAAAGGATTCTGCTTCATTATATTAGTGGTTTCTGGCATATTGCCCTTGTCGTTATGGTGACAGACCTCTCAATTATCTCATAAAGTCCAGGTCTGAATGTGATTCAAGGAGTTAAACTGACATTTGGACGCTGTACTTCCATGGGGTGTTCTGAGCTGTCTCCGTGCCTAACAGTCCCTCTTTGTGTGTGTGTGTGAGATGAATAAGAGCTCTCAAAAGCAATTAGGGTTCTCATTTGAGCAGCCACCTGGGTTGAGATCTTTCTCATAATGAACTATTCAAACAAAAACCAAAAAGAAAGGAAGACAAAAATGGGGAGAAAACCCCCCAAACAGGACAAAGGGTTAAAATTGCTTTCATAATACTTTGGATGTGCTAGAGTCTGGTGATTTTGTAGAGCTAGCCTTGGCAACAATGAATGCACTTCAAATAGAAGGCCTCCTCATATAGGAGTTGGACAGAATGAGACCACCCATGAAAAAGAATCAATAGCCTCCCTGACTGCAGAGCCCTGTATGTACAATTGTGTGGATGGAGACCACAAACGGTGTGGCCGTTTCATTGCAATTCGGTATTGAATTAAAATTTGAGGAATGTAAATATGTGAAAAATGCTATTCAGTGAAAAAGTAATCCAAACTTCATAATAAACCCAGTTCCACTTGTTTAGATCTTTAGGCTTTTTGAAGCAATATGTGCATATGATCTTGACAAGGGAATCAGAAATCTAATAGTGACTGAAAAGGTAGAATCGATCTCCCCACGATGTGTAAACTTTAGAATTTTGCTGGTGAGAGTTCAAAGCTACAGCCCTGCATGTTTGTACCATCCACAAGTCACAGCCTATTGGGTTAGGAGTTTTTATTTTTGGTTGCTTGCTTGTTTTCTTAACTCTATCAACGAAGAACCAGTGCAGGCCAGGCGCGGTGGCTCACGCCTGTAATCCCAGTACTTTGGGAGGCCGAGGCAGGCAGATCACGTGGTTAGGAGATCGAGACCATCCTGGCCAACATGGTGAAACCCCATCTCTACTAAAAATACAAAAATTAGCTGGGCATGGTGGCGCGTGTCTGTAATCCCAGCTACTCAGGAGGCTGAGGCAGGAGAATTGCTTGAACCAAGGAGGTGGAGGTTGCAGTGAGCCACAATCGCGCCATTGCACTCCAGCCTGGCAACATAGCAAGACTCCGTCTCAAAAAAAAACAAAAACAAAAAAAGAACCAATGCAGAGCTTTAGATGTTTAATTATTAATTATTCACTAAATGAATGAACTCCGCATCCACAACATATTGAAATGTTGGCATCATGCTGATTCTCTCCAAAGGCCTTCTCTTAGGGAGTATCTCAGTTCAGATCAATGCTTTTATTTAGCAGGAGAGAGAGCAATATTATTATTTGGAATTCAAAATTCCACTCTGACCAGTCTGACAAAGCCAGAAAGACAAATCTAAACAATAACAACAGCAAAAATCTACTTTTTTTGTTTAGCTTTGTCTTTCTGCCTTGATCAGATTGGCTCAAATTTCTATGTTTCTACTTTCATAAAATGTGTAGGTATATTAAAAATACAAAAATAGACTATTTTAGATACGTACTTATCCTTACATTTAAGAACTAACTTGCATGAGGAAAAGTGTTGGAAATTTCTTCGTAGTACAATAGTTTATGAAACATATATTTTTTTTCTGTAGAAAACAATACTTTTTATAATTCCCTTTAAAATAAATCAGGTCTTGCTGAAGGTGAGTCTTTTCATTTAAACTGGCATCATGATCTACTAAACTTAGGCTTGGGTCTTTATAACTATTTCCTACCTTACAAATTTCTTTATTTAAATTTTCATAGGTTATTAATTTCTCTTTGTTGTTAGACAACAGGCTAATTAATTAACTTGAATTGCATATTTAACCTTTTGATAGGTGCTCAAATAAGGTCAAAGTCAGTCAAGCCAGTCGGAAGCTCTAGTAGGACACGTGGGCCATTGTTGACAAGGAACAGTTGGAGACCGATTGACCGAATCTGCATGGTGTGTGTGTGTGTGTGTATGTGACAGAGAGAGAGAGAGAGAGAGATAGCAGAGAGAGTGTGACTGAGTGACTACTTTGAGGAAGCAATGCAGAATATGGCTTGGTAGCTTGATTAAACATAAATTGTGAAAGTCAAGCCGAGAAGTTCCAGTCTCACATACTAAGTCCACTTGAGTTCATACATGAGGGGATGGCAGTACAGTTCGTGATTCGTCTTGGTCCCCAAGGAGACTGAACACAGAAAGATGAGTTATGGAAACACTTAAGGTTTTTAATGAGAACCAGTGATACTGTTTAGAAGTGAGGTTAAAAAGTAAGGGAAAAATAAAAGACACATTTTGAAGGAGTTGCTCAGACAAGATATCATATTAAATATAAAGCTTGGAGGAGAAAGAGCCACAAGTGAGTCCAGATTGCCTTGGGAAATGGACAGACCCATGGAACCACTTCCTGAGTGACCTACACCTGTGCTTTTTCTCTGGATCCTTGGACATACATCTTAAGGTCTTATTCTTGAAAGATTTCAGGGGCGAGAAGCCCTTCCATTCTTCATCATGGGACTAAAAATACTGGGAAATATAAAGGAAAATATAAATGAAAGTCATTATCGCCCAGGCACAGTGGCTCATGCCTCTAATCCGAGCACTTTGGGAGGTCATGGTGGGTGGATCACTTGAGGTCAGGAATTCGTGACCAGCCTGGCCAATATGGTGAAACCCCGTCTTTACTACAAATACAAAAAATTAGCTGGGCATGGTGGTGTGCGCCTGTAATCTCAGCTACTTAGGAGGCTGAGGCAGGAGAATGACCTGAACTCGAGAGGTGGAGGGAGGTTGCAGTGAGCCGAGATCGCACCACTGCACTCCAGCCTGGGCAACAGAGTGAGAATCCCTCTCAAAACAAAACAAAGCACCACTCATTATCATTGTATTTTCATTGTAGCATAACAGCAAATGCCATTATGATTTCTAGAAAAGTGAAATTTTGGGTTGTTTTTTTTTTTTGCTAGCAATACAATTGAAAAAGGAAGATATTAAAAAAGAACAGATTATTGGATGCAAGGTGTCCCTATCATCTTTTTCCCCCAAGATGACACCTGACTCTTTGAATACTATGACTTAAGTAAGCTTGCTATGATTGTTGATTGAGGACCTATTTGGTGAAAACATGGAGCTTTATGATGAAATATAAACAGACACGACATGGACAATGACCTGTAGGAGTTTGCACAGTTAATAAACCTAGAGGTAGATAATAAGCCAGAGCATCCTAGTTAGGGAACAAAGAAAGCTCTGTGACAGCTCAGGGACAGGCTATTTTTTGAGGAAAAACTTGATGGAAGCTGTTAAGTTGTTGAGCTGTGCCATGAAGAATATATGGGTGATGGAAGGGATTCATCTATTAAAGCATCTGATGAATGGAACATTTGAACACAGAAATCTATGTTAAGCAGTTTGGTGTCAATCGTTGCTGTTGTTACTACTTGGGTGTTAAGTGTGGCGTGGTAACAGAAGCTGTGCTTTAGCATGGGCTGTTTCTGGCAGTGCCATATCATGAAAGTTCTTTTTTTTTTTTTTTTCCTTTTAGAAACAGGATCTTGCTCTGTCATCCAGGGTGAAGTACAATGGTGCACTCATAGCTCCCTGCAGCCTCAACCTCCTGGGCTCAAGGGATCCTTCCATCTCAGCTTCCTGAGTAGCTGGGACTACAGGTGCACTCCACCATACCTGGCTAATCTTTTTAGTTTCTGTAGAGATGGGGTGTCACTATGTTGCTCTGGCTGGTCTTGGGTTCAAGTGATCCTCCCACCTCGGCCTCCCAAAATGCTGGCATTACCAGCATAAGCCATTGCACTGGGCCCATAAACTTTTTTATGTTATCCACAGCTGCTGACCCTATACTTTCTAGGGTAGACAAGCTACCTAAGATGAAAGGGTGGCAGGAGAACAACAGGGAAAGAAGCTGGAAAGTCAACCAGCTTTGCTAGCGATTTTACAAAAAAAAAATGTATTCGCTTCTTTTATAGATACCACTGGATCTAATTCAAGATATAATTTATAGCATGGTTTTCATCCTTGAATAGCTCCCATCTTTTCTGAGGGTCTTACAAACTTTTCTGGCATTCTGCATTAGTCAAGAGATATTTGTGTTCAAATGGTAGAAGGCAACCTAGCCTCAATCTGACTTTGAGGGAAAAAATGGAAATTTATTAGAAGGGCTATGGGATATCCAAACTTACTGTAAAAGTTGAGAAATCAGATTGGCAGAATGGCAGGGATGCAGCTAGACTTTAGACACACCTGGAAGCATTGAATCCAAGGACATCACCAATCTTCATATCTCGTTCTTTGCTTCTTTCTGGAAATAGGCTTGCTTTAAATGGCAGTAAGAGGGTTCTCTGCAGTTTTTGTTAGTTGCATTTTGTTTTTCTCAGTACCACCAGTGAGGGACAAAGTTCCATAATTCCATACTAAAAATCCCAGGGCGGGGTTTTGATTGGCCCACTTGACTCAGGAGTAAGAAGAGATAAAACTGGGCTGTTCTTGTGTATACCAGTTGGCAGGGGGAGAAGGACAGTTCTCACCATAAGGTGTCTGGAATGAGCAGGCACTACTTCACTTCACTGTCCAAAATATTTTTGAGCATCGATTATATGCCAGACATGCCTTAGAGGCTGAGATTGTGAGAGATACAAGCATTCCTAATTTTGAGAGATAGGTACTTGTAGGCAGAAAAGTCATGGTCCCTGAGAGATGTGCAAGCACCGCCCTCCACCCCTACCCCCCAGCCAACTCGCCCATTCCTGGAACCTGGGAATAGGTTGGAGGCATGGCACCTGACTTCTTCAATACTCTGCCTTAAATAATGACTTCAAATGGCAAAGGGGAATTAAGGTTGCCGATTGAATTAGGTTTGCTAATCAGCAGACCTTCCAATAGGGAGAATCTATCCTGGATTCTCATATATATTAACAGAGACCCTCCACTGTGGATGCAGAAGACTCAAAAGGAGATCAGAGTTGGTGTAAAGCAACGTGAGAAAGAGATACCTGGACATTGCTGGCTTTGAATATGAGAGAGCCAGGAGAAAGGAACGCAGGTGGCAGTCTCTAGAAGCCGGAAGAGACAGGGAAACAGATTTTTCCTTAGAGCTTCCAGCAAGGAGCCCGACAGCCCTCCTGATACCTTGATTCTAGCCCCATGGAAGAAACTCTGACCTTAGAACTGTAAAAGAATAAATGTGTGCTGTTCTAAGCTTACTAAGTTTGTGGAGATTTGTCTTAGTGGTAATAGAAAACTAAGGAAGAGTTTTATCACCCTGTAATATTATTTGAAATTCATAATGAAGTATTACTCTGAAAACAAAAGTTCAGAGTCTCTGAAGTTGTTTGGTTTCGGGCCTTCTGGACCCCTCTCCATTCTGGGATTCTACTTCCAAGAATTTCTAGTTGAAAACACCCTTGGGCACTTAGAGCTTTCTACCTTGCTCAAGCATGCTAAGGAGATCATATCAATTCTTATTTTAGGGCAGACATTTTTCAGATTTTTAAAAATGTATTTTTTAAAAATTTGAGAGATAGGTACCCTGTCTCTGAATGGGGTCTTGCACTGTGGCCCATGCTGCAGTGCAGTGTCACAGTCATAGCTCACTGCAGCCTCGAACTCCTGGCCGCAAGTGATCCCCCAACTTCAGCCTCCTGAGTGTCTGGGACTATAGGCTGAGACTACTATATTGAGGTTCAGAGAAGAAGCATGTCCAGGTGTCTGCAAATTAGAAAATGGTGGCAGATTTTTTAAAAAAGAAACGATGAAAAATTATCCCTGATTAGATTTACATTACAATTTTCAGCCACCATGACTGGCTAGTTTTTAAATTTTTAAAGAGTTGGAGCCTTCCTATGTTGCCCAGACTGGTCTGGAACCCTAGCCTCAAGTGATCCTTTCATCTCAAACTCCAGAGTTCTGGGATTACAGGTGTGAGCCACCACGCCCAGTGACATTTTGCAAATTTGACATTTTGCATCATGTTAATATAGCCTCATGGCCAATTGTCCTAAATGGTATATTCAAAAGATAATACTGTTTTGACACAGAAAGGTACCAAAGGGTCATTTAGAATTTTTTCAGGAAGCTATAACAGATTTCCAGAGTAGATGGCTTTGAATGACATATAACAAAATACCGAAATTGTTCTTTCCTCATCTGTCTCCACAGAGTTTCACTCAAGATCGCGGCTGCACCTTTACATGTCTTATTTTCCTACTTACAAACACTGCTGACAAAATCCTCTGTGTTCCCCACTCCTTCCGGCTACACCTTAAGCTGTGGTCTCTTCTGGGCAAAGTGATTCTCTGACCTTTTCAAGCTACACCTTGTTTCCTCCTCCAACCAAAACTTGTTTGCTGGAGTTGAAATGCCAGTTTAGCCCCTTAGCAGATCAGTCATTATGGGCAAGTGACCCAGCTTGCTTGGGCCACAGTGTCCTTATGTCTAAAATAGAGGCGGCTGAGAGGTTTAAGGTTTTAATCCATATAAAGTGCTTAGTAGCCAGCACGTACAAGCACCCTGTAATCTGATGTTAGTGCAGCATCATTAATAACAGAAAAGGGAACCCGAAAATTTCAGCAAAATTGCATGTGCATAGTGGGTCTGGTATGTATATTAGTCTAGGCATAATAAATGTTGAACGTCTGTGACATAACTATTGTAGTAGTAGAGGGGTAAGCTTAAGAAGTAAGACCAATAAATAGCCCATCATTTCTGGCAGTTTCTAGTATGGTTTTAACAAAAGGGAATTTTGGGAGGAATAACATTTTTAAAAAGAGCCCACTATTATCATTCTGCTTTATTCCTAACTTTAGTCCTTTTGAGCCTGTGTTATCAAATGGATTTTGAGCATATGTGAATTAGAGAAATTAATCACTAGGAAAGGATTAGAATTAACTTTTTTGGAAAAGTTCCTTAAACCGTGAAAAGGCAGTAACACCATTCTTTGTGTGTGAGATTAAAGAGAAATTAATTTTCTTTCTCTTCTTGTCTAGACACACAAAGTCCAATTGTACGCATACAGTCACAAAATATAGGTGAAAAACGAAAACTGTGTTAACACGGTGAGACAGATGTTTTAACCAATCAACATCAACATGCAACTAGGTGAAAATAATTAAATTACTCCAGTTTTCATCTGTCAGTTGGATGTTTGACATTGTGTAGACACAGCTTATAAGTAAAGATAATTATGAAAGATTATTAAATAAAGATCTCCCTGACACGGATTAATTGAAAAGTATTTAGTATTTTTTGTAAGCACAGTTAAACTGGAGTGGATTTCCGATAGCATGTGTCTCTCCCCCAGCTCAAAAAGCTTTCAGCAATTTGAATACTGAGTAATAATCTTATTGAGGGTTTAGAAATTACATATGTTTGGAATAATACTATTTAGTAGTATGAATTATGCCTGTTTGAATAATTAAGAAATATCTTTTCCTAACAAAGAACATTTTCCCTTATGTACATAATCTTCCAATACATGAATTTTAATTCAATTCAATTTGCAATTTAGATTCTTGTCATAATTTGAACAAATACAGATTACCTAGAATATATTAAAAATCAAATTTTCACATAGTGCATATCATAAGAATTTTTTTTTAGAAATTGTCAGAGATAGAAACTTTAGGTACAACTAGTCCACTGGAATATTTGGCCATTTAAAACAATTAGCTCATTATTTATTTGTGGAGTCTTGCTTCCTAAGATGTTGTAGTCTTATTTGTTGTCAATTAATATTGCTGGTTTGAACATGGTTATTTATTTTCCGTACTATTTTAGCCAAGCTATTAATTTTTATTATTTATTTTTTTAATTTTATTTTTTTTATGTTTGAGACAGTCTTGCTCTGTCACCCAGGCTGGAGTGCAGTGGTATGATCTCTGCTCACTGCAGCCTCCACCTCCCAGGTTCAAGTGATTCTCCTGCCTCAGCCTGCCGAGTACCTGGGACTATAGGTGCCCACCACCACACCCAGGTAATTTTTGTATTTTTAGTAGAGATAGGGTTTCACCATGTTAGCCAGGCTCAAACTCCTGACCTCAGGTGATCCTCCTGCCTTGGCCTCCCAAAGTGCTGGGATTACAGGTGTGAGCCACCGTGCCTGGCCTAGCCAAGCCATTTAACCTTTAAATATTTAGTGTCCTCAGCTATTAAAAATAAGAGTAATATGATTATACATCCTATGAATTTGTTTTATAATTATTGTGATTTGGGAGTAAACAACTATATAAGAAATAATTATAAAAGAGATAAGATTAGTGCATATTAAGACTTTGATGTCAGGTTAATTGAATGTTAATCCCATGACTTTATCTTTCATTGCAAGATTCTTTGCCTGAGTGGGGTACTGGAAGCCATTGTTGAGAGTAGATCCGATCTTACTAGACTGTTGGCTGGTTCTCCTAAAACCAGGCTGTTTTCATAATGAGTTAGTTTAACATTTTGTCTTTATGTTTAAGCACCCCTTTCCTTGGTGCAGTCACAGCCAAACTGCAAACAGAAATCGAGAAGTTGTGAGCTCCAGATTTGAGAGCCACAGAGAGTTTGTGAGATCAAAAACATCCACTCTCAGTAAATAAATCAGAGCTACCTAAATCACACAGTCAGCTTAAAGGCAAGGGAACCAGAGGGAAAAACTCCAAAGGAGTGATCTCTTCATGCAATTGCTACTGGTAAAATAAAGCAAAGATGAGACAGTGTAGTCTCCACCTTATTATTTCAATCTAATATTCTATATTGAGGTTCAGAGAAGCAGGTCCAGATTTCCACAAATTAGAAAGTGGTGGCTTGCTCTTGTAATCCTAGCACTTGGGGAGGTCTAGGTGGGTGGATTGCTTGAGCCCAGGAGTTAAGACCAGCCTGGGCAACATGACAAAACCCTGTCCTTACCAGAAAAAAAAAAAATTAGCTGGGCATGGTGGTGCTGGCCTGTAGTCCCAGCTACTTGAGGGGATGAGGCGGGAGGATCACTTGTGCTTGGGAGATCAAGGCTATGGTGAGCTGAGATCACAGCAGTGCACTCCAGCCTGGGTGACACAGTGAGACCCTGTATCTAAAAAAGAAATAAAAGAGAAACATTTCCTTGTTAGACTTTACGTATCTGACGATGACTTTTGATGGTGAAGGTAGGCATTGGTATGTGGTCTGTGGTGTGTGTGTGTGTGTGTGTGTGTGTGTGTGTGTGTGTGTGTCTGTGTGTGAATGCTATTGAAGGAAACCCGGTAGGAGAAATATCCACAATTCAGTTAAGATCAAACATGTTACAATTTTCTGGGAAGTGCCAAGTTTTACAACACCTAAACTATATCCTCTTCCTCTCTGAAACCCCAAACATCCCAAAGTCTCCTTCAAGCCAGACATCCTCTTGGTCTACTGTGCATGGTGTCTGCACGGTCCTCAAGTTTGCCTCAGGGAAAGTGCCTGTTGCCATCAGAAAGAAAGAATGCAGCAGGTACTGATTTATCTCAGGCAAAGGAGCTCTTGTGGTGGGTTTCAACAAGATATGAAAATTGTAGGTTCTTGAACACTCCTTTTCTTCTTCCTTAAAATGGATGTCTTTAGCTACATTCTACTCTCTTCTCTGTCTTTTATGACATAATCAGTCATTCACTCAACAAGGGAACATCTAATATTCACCTAACATCCCATTTGCCTGTCACATATGGACTTTAGCCTCCAGTCGGGCCAATGACACTATTGATCTCCTAATTCCAATCTAGACTCTTTGGGTATTTTTTTCTCTTTTCCATTCCTTATTTTCTTTAGAGGCATTTTAGATAACTCATTTAAAAATTATTAGTAAATAAATCATTATTTGCAATCAGCATAGACAAGGCCTTGGGTGAGTCTAAGTGGATATCTGGAGAGATCTAAACCCGCTGCTGGAAAAGTGAGTGGGAAAGCCCCATTGATATGTGACCCAACTAAACCAACGTTTCATCAAAAGCAGTGTCTTCAGGGACTGCTTTAGGATTTCAGGGAAAAGAAAATGGAGGCAAATCTGAAAGTGGATGTTTTCTATGGAGGATCCTTGATAGAAAAGTTTTCACCCAGCCTTGAGTGAATATGCAGAGCGTAAACACATGTTTGTGCAGTGAGGAAATGCTGTCTATGTTTCCTAAAATGGAAGTTCTTGTTTATTGCTTCTTTAGCTGCACGGAGACATAAAAGATGCAAAACTGGGGAGAAGGGAGAGATAAAACTAAGACAAAACTGGAGGAGGGTGCAATGATGTTGTAATTTAACATGCAAAATACTCACTTGGGTATTTTTTAAATTGTTACATTGTGACATTGGAGGGTTCATAAATGGAATTCCATCCAAACTAATTCTAATGCCTATCTTTTCTTTTTAGCAGACTATAGAATAAAGTTAAATCAAAGAACATGAGGTCCCATTCTTACCAAATTCAAATATACTTTTTATCACCTGGTGTTTAAATCATTAATACAAAAGCTTTCAGTCTCCTCCAAATTTCTATTCTAGTAAAGTACTTTCATAATTTTATATTGGAAATGTACTAATCCAGATAACTAGTATGAAATCAAGTTATAATACTATTTTGCATGTTTCTAAAATGTTTACATTTAAAAATAGAGAAGTAAGCCTTAGGGAGAAAACTTCAGCTTTCCCAAGAATATTAAAATGTTAACAAATTATTTCATTTTGAGCTAAAATCAGATAATAATGAGAACAAATTTCACCATCGCACATTCTACAGGGATCTTTGCATTTTATACTTTTTTTTTTGTTTTGCTTTATAAGAGGGGATTTTGGTATATTGAATATCATACTGGAAATTTACCTGGACGGAAACGATAGAGTCAACTTAGACTTTAATCACAGAATGATAACATCTTCCAAGGAGAAGGAGCTTTTGAGGTCATTTCACCAAAACTCTTTCACCATACAGTATTTTCCCGTTCATTAACCTTTTGGCACTCTAAGCAGAGATGAAGTATCCTCCCCTGAGTTCCTAGAAGTTGAATTTAATCACCATTTTACGAGTCTGCCCTCCCCAGTAGATGGTAAACCCTTTGAAGACCCAGAGCATTTTTGAGATAAAAGAATGAATCATATACTTCAGTACATGGAACAAATGAATAAACCTGTAGTGCCTGGCCACCCAGCTTTTTTTTTGAACCTGACCGATAAAGACGTTTACAGCTTTTTAATTTCATTATCAGAGAAAGGGTTGGCAATATTTACCTGAGCACTCTCTACAAACAGAGATGAAGAAATTTGGAATGTTTCCTTTCTCTCCTAATACATAGCTTTGGAAGTCTTAGAAAACATGTTGGTATGTTCCTTCTAGGTAGTCTTTTGCAAGCATCCTCTTCAGTGTCAAGCATCTATTCTCATGCATCACATTACAGGTTATGAATATACCCAGAGTTTATGTGAGATCTTTTTTTGTCAAATGCATTAAACCCTTGGCTTATATATATTGAGCTGGAAGCCACAAGTTTTTGTAATATTTTAAAAGTAATATATTTTATAATATGCCTTAGAAATTAAAAAGAAAATAGAATACCTCCACTTCCTATGACAAAATGTCAGCATATACAGCAAGGCAAAGCCATTTGTTGCTGAAGCTCAGTTTTTCCCACCGGATGCTGAATGCACAACAATCACCAGCCAAGCCAGGAGTCTGTTTACTGCACGTTTCCCTGAAATGCCAAGCCCCTGAGGTGTTACAAGGAGGGAAGGCAGCATACATGTGTGATAGAATGGCCAATAAACTAATTGGTTTATAGTTTTGAGAAAGCAGCTGGTTGCCTGTTTTTAAATGCAGTGGTCTATAATTTGATAGAATGCAGAAGGAATCATTTCCAAGAAATTAATTAAAGTTCATAGGTTGGAAAATAATGGAGCTCATCATTAGGGAAAGCTTATTCTAAGACTTAGGATAAAATGAGCTTCCTCTTGCATTTCATTCAACTTAAGGTTTTGTAGTTACTTGTCATCATCAAAAATATCATCAGAGTCATCGCCATCATCATTATCTAAATTTGAGTAGCTATGAGAAGGTATTGTGAGGTCCTAGCTTTAGAGGAATCAATTTCTTTGAGATTTGATATTGTTATTTTAAGACTGCAGAGCATAGGTTAGAATCTGTGTTTTAAAAACTTTGACAGGCCACGTCATAGGTAGTAAAGTTTTCTCTTGGCATGAGTTTTGAGTTGACTTGTGTTATGGTTGAATTGTGTCTCTCAAAAAAATTGTTTATGTCTTAACTCCTGGTGCCTAGGAATTTCACCTTATTTGAAAATAGGATTTCTGCAAATGTAATCAAGGTAAGATGAGTTCATACTGTGTTAGGGAAGATCCTAAACCCAATATAATTGGTGTTCTTGTAAGAAGAGACACAACAACAAAGACAGAAACAGGGAGAACACCATGTGAGGATGGAAGCAAACGTTGAAGTGATTCATCCCTAAGCCAGGGAGCACTGTTGGAAACCACCAGGAACCAAGAACAACTCAATCCAAGACAGAAGCATGAAATGGATTTTCTTTAAGAGCCTCTAGAAGGAATCATCTTAATTTTGGACTCTGCCCCAGAACAGTGAGACAATGCGTTCTTGTTTCAAGTCACCAAGTTTGTGGTAATTAGTTACAAAGCCCCAGAAATGAATGCAGTCTGGATTAGGTATATTCTGCGTACATATGCTGCCTAAGAATGCCAGAAGCCAGAAGAGGTGATGTCTGCATTTTTGGTTCCTAAAATCCTCTCTCAGTACCCACTGCTCTGTCCAGGGCAAAGCTCCCCTGACACATTTTTAGCCTTTAGGCTATGTCCTATCTCCCCTGCTCACCAGAGAAGTAGGTCTTGGATTCCAGTCTCTCAGGGCTGGCATTTTCCAAGTGAAAGACACTGCCTTTGTGTAAATCCTTCCCCCTTGAGTGTAGGCAGGACATTGGATTTGTTTGTGTCTCATGGAATATGGTAGAGATAATGGAACACCACTTCCATGATTATGTTACATAAGCATATAAATTGTGTCTTACTAGTATACCCTTTTTGTTGCATTCTTGGTTTCCATGCTTTGATGAAAGAGCAGCCATATTAAACAGGTGCATATGGCAAGAAGCTCAGAGCTGCCTCTGAAACAACAGCCAGCAAGGAACAGAGGCTTTCAGTCCAGCAGTCCACAGGGCATTGAATCCTGCCAACAACCACATAAGTTTGGAAGCGAACCTTCCTCAGTTATTCAGCTTTAAAATGAGACCCCAGCTCAGGCCAACACCTTCATCAGTGAGAGACTTCAAAGCAGTGGACCCTGCTAAGGTTGTGCCTGGATTCCTGATATGCAGAAACTCATAAAATAAATACATTACTTGAAACTGTTAAGTTTTGGTTATTTGTTACATAGCAGTCAATAACTAATGTGGCATAATATGCAAAACATGGATTTCAGCTGAGCACAGTAATCCCAGCTCCTTGAGAGGCTGAGGTGGGAGGATTGCTTGAGGTCAGGAGGTCGAGGCTGCAGTGAGCTATGATAGCACCATTGCAATCATAGCTCATGGCAGCTATGAGCCTGGGAGACAGAGCAAGACCTTGTTTCTAAAAAAAGACATGGATTTCAAATTTGGCCAGATTGTAACCCAACTTCTACATAGATATTATGTCTCCATTGGAGGGATATATATTTTGAGACTTTGCAATCCTTAATTACTTAGGAACAATTAGTTAGCAAGTGAAAGAAATTCAGGTTGAATTCACTTAAGGGAAAAGAAGAGATTTTCGGGTTCCATTTACTAGCGGTGCATTTAGTTTCGAAAATGGTGTCCTCAGGTCTAATCATTGCTGTTAGGAATCTGGCACTTTGGCGCCATGTTTCTTCTTTGGCTTTCTTAGAGAGGCTTGTCCGTGTGTGGTGGTAGGCAGTCAACAGCATTTCCTAGTATGTCATCCTTTTCTCAGAGAAGCACATTGGCCTAGCAACTATGCGTACTGGCCTAATTTTAGTTGCATGCCAACCAATGTCTATATCCAGTGGAAAGAGATACTTGAATTGATATGGACTGCTTGGGTTATGTATACTCTTCAGAAATGAGAAGAGATTGGGTAAGTCCAGTAGGCTTAGGGTAGATGGAAGTAAGATTGCTCCCCAGAGGAAAATTGAATGCTAGGTAAGCAAAACTCATTGATGTCCATTGTTGCTTATATTACAAATAGTACCAAACAAGAAAGAATGGCATGGCTGCTTCATGGAAGAGGAGATGAACTTGGGGCAAAACCTTACCTAGGATATTTCCTTTTTTCAGCTAAAAAGAGGAACTTGGACATTCAGAAATGAGAAAACTTGTATATCAGTTGCTGTTGTTGTTGGTTTGTAAACAGCTGTAGCTCTTAGTGACATAGAGAGATAAAGTGACAGGAACAGATGAGGATATTTCTATTAGGATGTTATCCAGGCAGTTCTATGTTGGGAGTCACCCTCCTGGGACACTCCTGGGTCTGGAAGCTGTCAGCTGGTGGCAAATCAGAGATAGTCTGAGATTTAATGCCAGATGGGAAACGTGACCTCAAATGAATGAGGCTGTTTAGGAGTGGGCGCAACATGCTGTGCTTGCCATCTCTTTTAAGAGTTCTAACTGAAAGGTTAGGTTTACTGAAGGATAAGCCAATTTGGGGAGCTGATCTGGTGAACATGAATTTGGCCAAACTTCAGCCTAAGCGTTTAGCAGGGTGAAAGTTTGGGAAGAGTTTCGTTGTAGAACATTAGGCAAATGGCTGACAAAAGAGCTTCCAGTTCTCTCACAAGGAATTCTTCAAAAAGCAAAGGAGGTCCTTCTCAGTCAGCCTGCTCTTTCTGCTCAGTAGACTTCTTTGTGAGACTATGCTGTGAGTGAGTTCTCAGGCTGGTGATATAACCTGGTCTTCAATTCTTGTGCAGCTCTGTAAGTCCACGTAGGCACCACTAAATATCCTTACGACATTAAGTGTCATTGGATTGTTTGCTAACATTTGCTTCCATATGGGCCCCAGGCATTAGCAAACATGTAGTTTATTCATTTATTTATTCACTCAGTGAATATTTATTGAACTTATTCTAATTGTCAGGCCACTTTGCTAAATGTTGTTCCATCACTTTCCTTGCAGAACATACAGGGGAAAATGCACAACTAACTGGAATCATCATTTAGTGTAATCCATGCAATGATGCAACAAGTTGGGGAGATGTGAGAACATCTGGGAGAAGCATGTGTCCCAGACTGAGAGGGTGAAAATGCACTAAGGAGAAATTTGAAGAATCAGTAACTGACCAAATTGCTGGGAGGAGAGTCATTTCAGACAGACAGAGGAGCACGTTCAAGGCTGAAGTCCACAGCCTGACATTAATATCGATTCTCTTAGCTAAGTTTTGTTAAAGAAACCAAATGACAGTGAATTTGAAGTCCTGCACTCAGCCAACCGTATGAAGTGTAGTCACTGTATGGTCAGTTAATTACAGGGCAGCATCCTTCAGTCATCAGTCGAGCTAGAGAGAATATTGACAGATGTGCTCTTATGAAAGCTGAGAAGCTCAACCAGGACAAGTATTTAGCTAAAAGGGGGTCTGACCTCCTTTTAGAGATGGGAAGCAAGGGTGGACAGCATAACCTGTAGACTAAATCTATCACACTGCTGTTTTTGTGAAGGGTTTAATGGAACACAAATAAGCCCTTTTATTTATGTATTGTCTATGTCTGCTTTCACACTACAAAGACGAAGTTGAGTAGTTGCAAAAGAGACCATATGGCCTGCAAAGTCTACAATATGTACTATCTTACCCTTTATTTTAAAAAGTTTTCTGACCCCTGATGTAAAGGACCAACTTCATGAAGTCGCATGTGGATTTTCTAGTTACCATATAGACATGAATGGAAGAGTACAGAAGTTCCATGTCAGACAGCAATTGTTTTCAAACTTGCTATGAATTTTTTCCAAATGCAGATTCCTGGGCTCCATCCAGGCTTCCAGTGACTCAAAATCTGGGTATAGATTCCAACAATTTGCCTTTTAGTGACCTTAGAGGTGATATTGATGGCAAAAATTTTATATATGTACATATTCATGAAACAGAAAATTGGACGTGAAATATTTTTAATCCACATATAAACAGATACTCCTTTCTGTCATTAAAAACCAATTAGGAAAAAATGATAAAAGCCTGATTTTAAAACCATGGTCCATATGGCTTATGCAAGATAATTTTCTGAAGTGACCTTCAAGATGAAATAGTTGCAAAGTATATCTGTGTTCAGTTAAATTAGGAGGTGTGTGTGCAACAAGGAATTATTAGCCGTAGATCTTTAAAATCAAATCAATGTAAACAAAACACTGTCAGCCCAGTGGCCAAAGAACACAATCAATCAAAATATGAATAAATATACACAATTATACACTACTACTACTAGATGATGATGATGATGGTGATGATGATGGTTATGATGGTGATGATGAGGATGGTGATGGTGATAGTGATGATGGTGATAATGATGATGGTGGTTATCGTGATGACGATGGTGATGATGGTGATGGTGATGGTTATGATGATGATAGCAATGAAGATAACAATTATTGTGATGATAATTTATGGCGATAATAATGATTGTGGTGATGGTCTGTTTCTATGCGTCAATCTCAGTTGCTCCCCCAGACTCCATACAAACAGAACCACCTTAGAGATGTTTCAAACTTACCATGTTCGAAACTCAGCTGCTGCTTTTGACACAATGAATGCCCTCCTGTCTCCATTTTTACCATCTTAGGAGAACTCACACCATCCCCTCATCACTCAGTGAGCCAAGTGTGCTAGCTGCTGATCCACATGTCTGAATGGCCGCCTTGAGGAATTGACATTACCTTGGGGACCTACAGGGAGCAATGATGCTGGACTGGGGCAAGGATGAATAAAGGAGGGATAAGTCCAAGTTGTTGGGGGAAGACAGGGCAGCCAACTCTATCTGGAGCTCTCAGATGGGTTTAGCGGTTGTGGAGATATTTCCAATGGCATTTTGAAGACGTGGAAGAATGTTATTAGGCATAGCAGAGATTCTTAACTAAGAGCAATTTTGGCCCCACTGTAAGGGACATTTGACAATGTCTAGAGATATTGTTGGTTGTCACAGCTGGGGAGGTGCTACTGACATGGAGTAGGTGGTGACCAGAGATGCTGCTGAACATGGTAAAATGCAGAAGAACGACTCACACAGCAGAGAATTATCTAGTCCAAAATATCAGTAGTTCTGATATTGAGAAACTTGGCTCTGTATTGTGCATGTGTAATCGTTTTTTACTTACTGATTCTAGATTCAGCTGGCAAGGGGGTGTCAGCAATGTCTGGAGATATTTTGGATTATCCCATCTGGGCAGTGTGTGCTCCTGACATCTAGAAGGCAGAGGATGCTGCTAAACATCCTACAATGCACAGTACAGCCCTCACAACAAACATAATCATCCAGCCCCCAAATGCCCACAGTGCTGATGTTGTGAAACCCTGCTCTAAGTCAAAGCATTGTCTTACTCAATTTTTAATTCCTAGTGTATATCAGTGGTTCTCAACTTTGGGGAGGGGACAGGTTTGCTTCCAGTGTACATTTGGCAATGTGGGAAGACATTTTTGTTTGTTGTGAGTATGGAGTGTGTTACTGGGAATGGAGGCAAGGGATGCCACTAGACATCTTAACAGTGCATAGGACAGCCTCCACACCTCAGAATGATCTGGCCCCTAATGTGAACAGTACTGAGGTAGAGAAAACATGAGGTAGACTGTAGAAGCCTATAGAAGAAGAGAATCTGAGAAAATTGTTGTGCTTGGGGAACACTGAAGAATGTGGAGCAATTGAACAAATGCTTGTGCAGACAGATTGGCACCAAATTGCAATGGAGCACCAATGGGACAGTGAAAAGGGACAAGTCCTACAATGCACAGTTCTTGACCATCCCCAAAGTGCTCCAAAGCTACAGAAGTTGGTGTGCATGTATTATCTCATTGATCCTATTTGGGAATTATCATGTTGACAGCTGGAGTCCCATGAAGGAACATTTTTAAGCAGCAAAGTGACAAGCTCTGATTTGCCTTTTGAGATTAATGACTCAGAGACTGCCAGTTATTTGTTAACTTGCTTGATTCAGCCTAAGCAGACATCTAGAGGGTGTAATTTGATTTATTCTGCAGAGGGGTGATTGGCCCCTACATTATCTTGGCACACTGCCTGAATTTCTGAACACCAAAGACTTATTTATTTAGTGTATGGCCATCTCATTTCCAAGAGTCACCAAAGAAGTGAGAATGGATTAGATAGGGAACAAGCTGACCATTGGATTAGTTTATCAGATGATTAGCATGCCATGCTAATTTATCAAGACATGGAACATTTAAAGAAGGGGAGAGTAACATATACAGGGAAGATAGGAGATCTTTGTCCCAATTATTTCTTTTTTTTTAATGCATGAATAGTCTTTTGGTAAATATAGTTTATGTTTGTTTCTGCTTTCTAAGTTAGGCTGCAAAATATTATTTATCGGTGGTATTCTTTGAAATTGATTGGCATGGCAAGACTGTAAAAGAGTATCCATAGGTGTATTTAAAAATAAAAGATCGTCTTTTCATCTTTGCAGAAAAACATGTATTTACTATTGCTTGGAATAGAAAGCAGAATTTTGCTGTAGCCATTAGGAAGTGACAAACACTACGCCATAATTATAGTGAGAAGAAAGCATCAAAAAGAAATGTTTTGGTTTTTTTTATATACAGTTGGCACAAAAATGTCCACATATATGAATACTCTAAAGAATGCACCATAAAAAGAACCTTCCACCACTATTAACAGGATTAATCCGTGCTCATTACCATGGGATTGGGGATACATTTTTACATGTTCTTGATTAGATTCAAGAGCCAAAGAATAAGGCCTAATTGATGAAAGTGGGCTCTAATTTTGTGCTTTTAAAATAATGGCCTCTGGCCAAATATGGGCAAAAGAAACAGCACTTGATTTGTTACTTTACATTTGTTTCTTGCATCCTGCTCGAAAATAGAGATGATTTACAGTTTTAATATATTTTTCATGCACAATTAACATCATTGTTGCCAGTTTTATAGAAGAGGCAGGAAAGTGGGCCTTCTATGATTTATTGTGAGTGCATGAAACAGAAGTAATGCTACTAGCAACAGAGTTTTAGTAGGAAAAAGTTAAAGCACACAGTCTTAAAAAGGAAAGGTTGGTGTCAAAATTATGTTTGCTTTAGGTAAGCTTTATACCTCCATGGATGGCTTTTTTTATAGTAACAACAACAGTAACTGTATTTACATTGGGGCCTTTTCTCTGTTTCAGAGGCTTTCATGTGGAGTGCCAAAATGGTAAAATATATAACATTGTTATATGAAGGAGTGAGGGAAAATCCAATCAAGATTGGCATTTTTTAAAAAAGAAAAGGAGCATGGGGAATATTTTAAAGATTTGGGGCCAAGCCTCGTGGCTGATGCCTGTAATCCCAGTGTTTTGAGAGGCTGAGGAAGGAGAATCACTTGATCCAGGAGTTTGAGACCAGCCTGGGCAACATAGCGAGACCTCCACCTCTATAAAAAAGACTAAAAAGTTAGCTGAGTGTGATGGCACGTACCTGTAGTCTCAGTTACTAGGAAGGCTGAGGTGGGAGGATAGCTTGAGCCCAGGAGGGCCAGGCTTCAGTGAGCTGTAATCACATCACTGCACTCCAGCCTGGGCAACAGAGCAAGACGCTGTGTCTCAAAGAAAAAAAAAAAAAAAAGATTTGGTATCTTTCTTTCCCCCACAGTTTGCATATACATTGAAAACTGTGCATTTAAGCCAAAATAGTTTTTTTTTTTAAACATTTCACTATAAAAAAGGAGTCTGGCTTTCACATGGGTACATGATTTTGCTTTGGCTTCTTCAATTCCCACCTGCCCTGTTGTGAGACCCATGAAGTAAGCAAAGCATTCTTTTTGCCACGGAAATGAAACTCCTAAACATATTGTTTATTGTCACATAATGGAAAGGAGAAACGTTTCAAAAATAAGGATACATGAAGCCCTTATTGAAAAGCAATCATACATTGGTGAATTTAATGTTTTGGAGCAAAAACTGTTATGTTGGATACCTATTAGTCTTTTTAGCTAGTGAAATATGTACAAGGCAAAATCAAGCATCAATAGAAGGGTCTAACTAAGCTTGTTTCTCATATGGTTTCTCTGCCAGCTCACACCTCAAGGGTGCCTCCTGCCTGCAATGTGTACTCTCTGGTCCACACACTGATTTCCCCTTTTCTGTTTCATGGGGTGACTTGCTGACCTTCTCTGTGCATGGCTAGTAGTACTCTATTGACTGGCAAGGGTTGTGTCTTCCACTTGGGTCTTCCAAGCTGCTGAAGAAAGCAACACAGAAAGTATAGCTGACAATAATTATCTGTCAAATGTATGTGAATCACAGTGTGGATGGTCGACCTGTTGTTTCTTTTTTCTCTTTGAAAGGAAGATTTCAGTTTTCTCTGCAGCCATGGTACTTTATAAATTATTTCCTCTTCCATCTCTTAAAAGTCACTGTTATTTACCACCCCATTAGCTGTGGATGGGGTGAAATGCCCACTCATGCAGCACAGGAGGATACACAGATTGTCACACATCTTTTCAGGAGACCACACAGCAGTGGGTAGTGTAGTATTAAATAAATGCCTGAAATATGAGCTGGGAATGCATTGCACTTCAAGGAATTTTATCCATAGGATGTAACTGGGAAAGTGCAGAAGAATGCATATATATATAGTTGTTCATTGTTACATGTTTTATGATAGCAAAAAAAAATTAAAAAATATTCAACTTTCATTTTAGACACGGATTTGCAGGTTTGCTACATGGGAATACTGTGTGATGCTGAAGTTTGGGGTATAGATCCCATTACCCAGGTAGCGAACATGGTACCCAACAGGTAGTTTTTCAACCCACATCCCCCTGTCTTCCTCCCCTTCTAGTAGTCCCTAGTGTGGAGTGTTCCCATATTTATGTCCATGTGTACTCAGTGTTTAGCCCCCACTTATAAGCGAGAACATGTGATATTTTGTTTTGTTTTCTATTCCTCCATTAAGTAACCAAAATTTTTAACAATGTAGAATCCATTACATAATTAGAGATACAATACAAGCATTGAATACCAGCTGTTAAAATGGCATTACAGGATAATATTTAGTGATATGGAGGAATATTCAGAGTGTATTATATACAAACATTTTCATCATATCGTTTTTTACTAGAGTGGACTGTCATTTTCTTGTGGGCTCCCTTGTATTATTTACTCTATTGCATCTCAGTTTTGTTGCATATTATGTAAAATAGAAGATAATGATAGCTTGGCGCATTCTCTGCTGAGACTATTTACAGTGGTGTAAAAAGATGTTGCCAGGGGTGTGTGCCTCAGTCTGTCCCAGCCTTCGTAGGGCCCCATGTTTCAACTCCCTAATGACCCATTGAAGACACACGGGCACACAGGGGAGAATGCTCTGGTTTAAACAGTCAACCATAAGCCAGACACAGTGGTGCAACCTGTGTTGCACCTTGTGGTAGCCTCTTGCTACCCAAGAGGCTGAGACAGAGGATCTCTTGAGGTCAGGAGTTCAAGACCAGCCTGGGCAACATAGCAAAACTCCCATTCTAAAAAATTAAAGCAAACTCAACCATTTTGAGTTTTACATGTTGTAAATATCTTCTCCCACTGGCACCCACCCATCATTCCTGGTTTTGATTGAAACAAAACCATTAGTTTTAATGTAGCAAAATGCCATCAACATATTTTTCTTTCTAACGGTTTCTCCTACGTAGTGCCTGTTAAAGAAATCCTGTTCTACCCCAACATCACAAAAACATTTTCCTATAAGTATCAGAATTTCATTGTTCATACAGACAGTTTTTAATCCATGCAGAGTTTATTTTTATATATGAAATGAGGTGGGAATCTCATGTTATTTTTTTCCCCAATAGGGGAACATTGCTTTGACACATGAAGGAAGCAATGTATTCTTTTTTTTCTTTTGAGACAGAGTCTTGCTCTGTAGCCCAGGCTGGAGTGCAATGGTGCAGCCTCAGCTCACTGCAACCTCTCCCTCTCAGGTTCAAGCGATTCTCCTCCCTCAGCCTCCCAAGTAGCTGGGATTACAGGCACACGCCACCACGCCCAGCTAATTTTTGTAATTTTAGTAGAGATGGGGTTTCACCATGTTGGCCAGGCTGGCCTCGAACTGCTGACCTTGTGATCCACCCTCGGCCTCCCAAAGTACTGGGATTACAGGCATGAACCACTGTGCCCAGCTACAATGTATTCTTTCCCAATGATTTGTGGTGTCAGCCAGGACCTTGATAGGGATAAATGGCATGCAACTTGAGAAATGTAATTAAGATGGGGACAGGATAGTGGAGTCCTTATGTGAAGTTGCTGATGCCCGCTGAGGTTGAACTGGACCTACCTACCAGGGAGGGAACTGGAGGTCATATATACAGGCCTTACTCGCCTTCTGCCCTCCGGATTACCTGCTAGTGTCTTCCTTGGCTGAAACCCAGGAGCAGCCAGAAGGCAAGAGTGAACCTGTTTATTTACCTTCCACACCAGAGAGGAGTGGAGATGAGGAAAAGTCTTGAAGGGGACAGACTCCTCCCCCCACAAAATAGTACAAGCTTTTAAAATTCATCATATATACATCAGCCAATCCAAGGGCTTTATATTTGGTCTTGTTGATTTCCTGATCCATTCCTGCAAGATTAAAGTATGACTCAAATAGTACAAATGCCCATATATTTTTCATCTTCAACATTCTCGTTGCTTTTTGTAGAATTTATTCTTTCATATACAATATGGAATCAATGTATCAAAATCTGCAACATTCTTCTGTCTTTGCTGGGAATTGTATTTATTGAAATGTTGGTTTGAGGAAAAATAAACATCTTCCAAGCTCATGTTATCTCATTTGTAAACTGGCATAGTTCATTACTTGTTGAGATCTAATCATAGCTTTATTAAAGACTTTGAGCATTATGTGTTAATTGATTATTATTATTATTTTGCAAATGATATCTTCAATTACATTTTCTACTCCTGGTATAAAAGAATGTCGATCTTTTTTATACATTGATTATATGTTCAGCCATCTTTTTTGATTCCCTATTATTTCTAGTAGCTTTTCTGTTAAATTACATGGTTTCCATAAAAATGGTGACATTATGTACAAATAATGACCATTTTCTCTCTTTCCTTTCAATACTTGTAATTTTCATTTCCTTTATAACTTGTACCATTGTATGGCCCACTGACGTCCAGTGCGAGGATGAATACTGTTGGTACAAACTTTTGTTCCCATTCATGATTTTACAGGAAATGAGTCTAACATCTTTTTTGTAAATGCAGCGTTGAGGAGAGATTTTAAAGCATGCAGTCATTATCAGATAATATGAATTACTTGCAATTCCCAGTTTTTTCTAAGTTTTTAAAAAATGTTTTCTTTTGTTCATAAATGTTGATTATGACCAAATAATCAACTGGCATTTCTACAGCTGGTTATATGATTCTTCTCTTATAATTAATGTGCTCTGAAAATTAATATATTTTTAAATATATATTCAATTTCGGGAATAACACATTTTTAATCTTAAAAGAAACATTTTTAAAATGGCCATTATTCTATTATAGTGGAATATATTGTATATGAAAAATAGCTACTATTCTACTAAGTTTGGTTTGTAAATATTCCACTTAGGTTGTCTACATCTACCTTCATAAATGAATTTGATTTATAATTTTCTGATGTTATACACTCTATACTTTTGATATGAATGTTAAACTGTCCATACAAAAGGATTTGGGTAGCTTTCTTTAATTGTATATTTTCTGAAGAAAACTTAAATAAGTAGAATTACTAAAATTTTTGTGAAAATTATCTTGGGTGGTGAGTTTTTATGTGGGAGATTTTTAGTGATTCTTTCATTACTACTTATAGCTTTTAGTTTATTCATTTCTTTGCGTAAAGTTGCTTTGTTTGTTTTTTTCCTCAAATATTTCAATTTCTTTTTTTAATACCAGGGCTTATACTATTAAAATAGTATTTTGTATTTTTTATAACTTTGTTTATTTGTTATTTTAAAAATGATTTTCCTCTTTAAAGACTATTTGTTCTCATTATTTGTTGTATATTATTTGTTGTATATTGTTGTATATTATTTGTTTCATTATTTGTTGTATATGTTACTCTTCCTTGGTCAGTCTTGCCAGAAGTTTGTTTATATTATTAAGCTTTTCGATAAACTAGCTTTCATTTTGGTAATTAGCTCAACTGTTTTTTCTCTGTTTCGCTAATTTCTGCTCTTACCTTGATCATTTCCTATTTTCAGATTTATTTGGATTTATTCTGTTTTCTCTTCTTCCTGTTTCTTGACTTGCCTCCATGGCTCGTTTATTTCCAATTCTTCTTGTTACCTTGTAAAGATATTTGAAGTTTTAATTATCCCTTTTAAGCACTTCTTCAGTCCCATCTGACAAATTTTCACATGTGACATTTGAACTATCACTGGACTCTGACTGTTTTGTGTTTATACGGTAGCATAAAGGCACATGCACACATATACATACACACATAGATGTGTGTGTGTATATGTTTAGTGTTCTATCATTATTTTGAATGCTTTTTACTATTGATTTCTAATTCTGTTGACCGATAGAATATAGTGCTGAATGCTGCTGTTTCTTTAAAGTACTCTTTATGAAAGGCAGATTTTGTAAACGTTCGGTGTGTGCTTGAAAGCTATGGACACATTTACACATACATAGACATATTCACAAATACAAATACAGATATACGTGTATATGTGAGAATGTGTGTTTTGAGGAGCATAGGTTTCCATAGATACCCACCAGATCACATGTATGGGTTACTTCAGTCTTCTATATCTTATTTGTTTTGGTGGGTGGGGCTAGGGACAGAGTCTCGCTCTGTTGCTCAGGCTGGAGTGCAGTGGCCTGATCTCGGCTCACTGCAACCTCGGCATTCTGGCTTCAAGTGGTTCTCCTGCCTCAGCCTTCCAAGTAGCTGGGATCACAGGTGCACACCACCACGCCCAGCTAACTTTTGTATTTTTAGTAGAGACGCGGTTTCACTTTGTTGGCCAGGCTGGTCTCCAACTCCTGGCCTCAAGTGATCCACCAGCCTCGGCCTCCCAAAGTGCTGGGATTACAGGCGTGGGCCACTGCAACTGGCCTATATCCTCAATTACATTTTATTTCCTAAGTTTATCACTCCAAGAATGTTGTGTTTTATTCTACTGTAACATTTTATCTTTTCTTATCTGTCCTTTATCTTATATATTTAATGTATATGGATATACTATGTTATATATATGTAGTATGTATATATAAAATGTACTTATATACCTTTTACATGTTTTGAAGCTGTATTATTAGGATGTTACATGAAAGTGTCAGTTACACCTTTTTAATCTTCCATTCCTTTTCTAGTATTTATTATCCATTTTTGACATTTACAATTTTTGTTTGATACTAAATTTGCTTCCTGTGATATTTTTTCATTTATATTTTGTTTTATATTTAAAATTTTTAGTGTCTTCATTTTCAAGTTTATGTATCCATTTATTTTAAATATATCTTTTCAACAATATGTTGCTAAAAGTATTTTAATCAATATTTTATCTTTATTCTAATTTTATTTCTGCAGTTATCATTATTATAGATTTCACTTCTGACATTTTATTTTATATTTTATATTTATCAATCATGCTTTTTAAATTTTACCTTTTTTTTTTTTTGCTTTACCTGACTTCCATTATATAATTTTAAAAGTTTCTTTTACTGACCTTATTATTATATTTTTCTTTCTTCTGTTTTTTTTTCCTTATAGTTGGGATTCATCAAATTTCCCTCTTCCCATTTTATGCTGCACTTATATTTTAATGAAGATGTATCTAGTCTTATTAGCTATCAAACATTTCAGTATCCATAATTTTCCTCAAAACAAGATATTGATTTAGCATTTTCTCTACTCTTCGGCATCTCTCTCTCTCAATCACCCCACACTGTGTTAGATTCTAAGAGAATCTGGGCTCTAGATCATGTTAAAAATTTGATTTTAGATCATTGTTTCTTCGGAATAATTTTTTGTCGTTACCTGTATTATGTTGCTGTGTTCTGGGTTCCTCTCCTTGCAGAAATATATTGTGTCAAGATTTCTGTGATGTAAGTGGATTTGGATTTAAGCTATCATTTAAATGACAGTTTCACTGGACATAAAATCCAGGCTGATTTTCTTTCCCTTGTACTTGCTGGGGGTGAGAAGCCACTGCATTTTGTATCCTACGTTGCTTTGCAATTAGCCTGGTTTTCATTCCTTTGCACATCGCCTGCTTTTTCTCCTTGGAAAAATTAGACATATTTTGTTTACATTTGAGGTACTCAAAAATTGGAATTTGTTTTTGCTTTGTTCTGTTTTAAATCAACGTATTATTTACTTTGTGAGTACTTTCACTTTTAAGCCTTTTTTTTTCTTTCATTCTGGGAAATTCTCAGCCTTTCTGTCTAATGTAGTTCTTCCTAGTCTTTTTCTCTTTGTTCTCTTTCTGGGTCATTTTTTTTTATAGGACTGGTAACACTTCTATTTCCATCTTCCATACTTTAGCATTTGGAGGATGTTTTTCCACCATTTTTCATCCCAGATCCATTTTGGGAAAATGTATCTCTGTCTTTTGGCTCCTATGTGCATTGTTTGTGGGTATCCTTCCATTTCAGTCTGTTCTTTGTGCTCTCCAGTTCAACAATTTCATTTCTTCTCCCCGGTATCTCGTGTGACTTCCTTTGAAACCCTTTGTTCCAACTTTATATCGCTATCATTGTCTCTCTGTCCATTGGAGGGATCTGCTTCTTTTGAATCCCAGTTTGTTTACTTGGGTCATTTTATTATTATTATTTTTTAAATAGGATGTTCCTTTTCTTTTAAGTGCTTTGCTTTTTGACTGGCTCTTAAAAATTTCTTGGGAGTTCTTTTATTTTCTTGAGGCCGGTAGAGGTCTTGGAAGGTACCAAGTGTCCAATGGGCAATCAAAAGCCCACCTCTCTGCCTGGCGCGGTGGCTCACACCTGTAATCCCAGCACTTTGGGAGGCCGAGGCAGGTGGATCATCTGAAGAGTTCAAGACCAGCCTGACCAATATGGTGAAACCCCATCTCTACTAAAAATACAAAAATTACCTGGGCATGGAGGCATGTGCCTGTAGTCCCAGCTACTTGGGAAGCTGAGGCAGGAGAATCACTTGAACCCGGGAGGCAGAGGTTGCAGTGAGCAGAGATTGTGCCACTGCACTCCAGCCTAGGTGACAGAGTGTGACTGCATCTCAAGAAAAAAATAAAAAACAAAAAATAAAGGCCCACCTCTCGATTTCATGCCTCTGGGTAAATTGGAGGGAAAAGAGGGTCCCTCTGTGAAGAGCCCTTGGAACTCGAGTTCTAATTTCTAAACCAAGAACTTTATATTCTTTCCTCCCTCCCTATCACTTCCATCCACTGGCTGGCTCTTATCTGAAAACTGTCGTGTGCAGTTATAAATACTCAACACTTAGGGAAGGAGAAGGAATTCTGAGAGATTTCGCCAGCCTGATTCTTTTCATTGCCATAAAATTCCACTGCTTTACCAGAAATCCTTGGAATGTGGCTTTCCTAGCTTTGCACTGTGACCTTCTTCATTCGGAATAACGAAGATGAGAAAAGCATTGATCCGCCCAGACAGTGAGGAGCGAAGAGCAATACCTAGGTGGAAAGCTCTATCTCCCCTGACTGTCCTGTGAAATGCACCTGAGTCTCAGAGGACTCCACTGCCATCTGTCTGTCCAGGAATTTCCCATTTTGTATGGCGACTTCAAAGTAGGTAAATACTTTGATTAAAGGAATAGAGAACAGAATTTGGGTAGCTTGTTCAAAAGATGGCATGGAAAATTCTGTGACTGGAGTAGTTGTGAAGCATCACTCTTCCCGTAAGAATAAAGGAGGCATTTGCCAGATGTCTGAAAACACACAGACACACACACAAAGGAATTACTTCTGGCTGCAAGAATATTCTCTCTCAGCATCTTCCTGCATCTCCATGGGCAAACAGACCCACAACAGCCTGGGATTTTTTAATTGCCAACAGTTTTCATTGCATGAGAGCCTGACATGTCTGTTGCATGATAGGGTGTGTTTTTATTTTTGGCTTCCTATTGGTTTCAACATATCCCTCCTTCCATGTCATAATGACAATTACAAAGACCTGAGTTGAACCTAGAACGCTTTTTTTTTGTCAGACACAACAATGCAGTGGATGTTAGTCATAGGGTAATTCAAACAGAGATAATTTTGTATATTCTAGAATATTATGTTTTCAAACGTAGGTTTTGATGTACCATAAGATTTCTTCTGCCATTGAGGCGATATATATGTGTGTGTGTGTGTGTGTGTGTGTGTGTGTATGTATATATATGTGTGTATTTTAAATTTAAATTAGATATTTTTTAGAGGCCTTAGCCCTTAAGCAGAATTCCCTCCTAATTTAATGATTTTGGACGAAGCTCATTGTGAATCATTTAAAAACACATTCATGCTTCTTCAAACAGAGGTAACAAAGGATACAGCACCTTGACTTGTTGACTAAGTGCTGTCATGGTAGATGTTATTTAGCATAGAAGATGCCTGCAGGGTCAGTTCTACTCTCTAAAGTTTCTTGAGGCTGTGTTAAATGAAATCAAACACCTGTGGATTTTTTATTCTTGTTCACGCTTTTTATACCTCTCCTTTCTTCTCCCTGGGCAACCTGCTTTCACACTAGTGCCTACCTCTGTTTTCCCTTCAGAATGTGATCTATGCTACACAATCTGATTAACAAGCTCAACAGAGTTCTACTGGACATAGAATAAAGAAACCAGTATAGTTTTCTCTCTAGGGACAAGGCAGTGAGGAAGCCAGTTTGAATACAGGTTCTTGCTCTTGTAAGCATTGACATTCAGCAGGTTCCTTACTTTCTGAACACTGCAGTTATATGATGGGCAGACAGGGACTAAGAATAACACCTACCTCAACGGGGCTGTTGTGAGGATTACTGAGATAATTTATGTAAATCCCTAGCACAATGCCTGACTCATGCGAGATCTTTAATTCATGGTAGCAGTTACTAATTTCATTTATCATAATGAGCTGCCTGAGCTACCAAGGAGCTCTGCCACTCCCAGTACTGTTCTACAGTTCTTTAATTCAACAAAGAAATTTTTCTTTAGTTCCAAATAAGTGCCAGGCATCAGGCTAGGTGCTGGGTGTATGATGATGATCAAAACAGTGTTCGTATGGGGGTAGTCATCATTTTGTCGATGGGCCATTTTTTATGATGTCCCTCTTCATTATAGGTCTTGATTCTTGCCTCTGTTTTGTATACATATGTGTTGCGGCAGGGGCTTGCTATAAAAATCAGAATTGCCCAGGCTGAGCGCAGTGGTGCAATCATGGCTCATTGCAGCTTCGGGCTTCAGTGATCCTCCCACCTCAGCCTTCTTAGTAGCTGGGATTACAGGCACACTCCACCACACCTGCCTCTGTTTTGTGTAGCTGTGATTACGTAGCAATTTTCTGAATCAGTGACAAGATGCAATGCATATTTTTTTCAGTAGGTTAATTAATTTATCTAATCTACATTTGGAGCTATTTTTTGGAGTGTTAGTCATCATAATAAATATGGTGGCACTGTCAATAGTAATATAAATATAATGGTACCTTAATTCCATAATACAAAGATCACGTCTTCATGACTGATGGGCCATTTCAAACCCATAGGTACATTTGCTCGCTCTGTAAAGTATACAAAAGTAAGAATTCTGGACATCTTTAAAAGTTGTAAATTTTTACATGAAAACTTACATTCACACCATCTTTTGAATATTGAAAAGATTTGGGAACATGGGGCCTATATGTGACTGTGGATGAGGTGTGGCTGTTCCCTTTAGACACAGCACTCACTTTGCCATAGTCACACTCCCCACCGCTCCCTATTGTGTCTCCAACCCCCAGGCTGTTGTCTGTTTCTTTTCCAACGTTATTACCCACTCATAGATGGTCAACCTTATGATCATTGTTACTTTCTTTTCCTCAGAATCTTTCTAGTATTTGTGATTTTTTTCATGTGGTTATTTTGAGCTTTTTGCATTAAGAATTTGGGATCACATACTCAAAAGTTTAGTATTTACCAGTTTGTATTATTGAGCACTTCAGAAATTTATTTCTGTTGCTGTTATCAACTCATAAAATATCTGTTTAATTATCCAACTAAAGACTAGATAGGATAGTGATTCCTATTTTCTCCAAGCTCATATCTGTGAACTCCTTGATTGCCCAACATAGGCATTCAATCATTCATTCAACAAATACCCATTGAGGACCTACTATGATCTGGGCACTTTTCTAGGTGCTGATAATTGTAGTGAAATAGTAGACCACAGTGGACAGTGTTTCTTTATGGAATTTAAGTGAATAAGGAAGTTATTTTGGAGTATTTCAGATCGTGATTCCTGCTACGAAGAAAAATAATTCAGAATAAAGTAGATAAGGAATAATAGGAATGGACCCACACAGTTATTATTTTTATTGCTGTGGTCATACTGATATCTGAAGCAAGTAAGAGAAGAGTTTCCTATGAGGATGGAATAGCATGTGCAAAGACCCTGGAGTTGTAGAATCCTTGATGCGTCCAAGGAATATGGAGAAGACCAGTTGGGCTAGAGTTGACAAAATGAGGGTGAAGTGGGGGTATAAGAATAGAGAGGTGCTGGACAGTAGGCCGTTGAGAGGGCTTTAGCTTTTCCGTGATGAATATTGGAACCCACAATGTAATTTTGAGCATGAAAATGAGAGCCTTGATTTACATTTTTATCAGATCACCCTGAGTTCTGGTTGGAGAATGAGCTCTAAGGATCTGTGGGTATATTTAGGGAGATACTTAGGTGGCCTTTGCAATAATACGCTCAAGGGAGGATGCTGGCTTCACCAGAGAGCTGATAGATAAGCCATGGCCAGATTCTGGGAATATTTTAAAGGAAGATCCAACAAATCGATTATTCCTAGAATGCAGAATGAATGAGAAAGAGACAACTTATGGCCAACCCCAATTCCTTTGGCCGCCGTAACTGGAAGAATTGCGTTGCCATGTGCTGACAACAGGGAGATTGTGAGAGGAGCACTTTAGGGTGAGGGAATTAGGAGACTGCTTTTGTTTAAGTTAAGAACAACCAAGGAGAGATAGATGTCTTAGAGACAGCTGGGTACAGTAGTGTGGACATGAAGAGAGAGGTCTACGCTGGAGATACAAGGTCAGGAGACATGAGCATGTAGATGATATTTACAGTTGTGAGACTGAATCGCATTTCCAACACAATGAATGTAGATAGAGAGGAGAAGTAAGTGTACTAGAAGAAAAAGAAGGATGAAGAGGAGGAGAGAGAGAAGACAGTGAGGAAGAGGAAAGAAGCAGCGTGCATGTGTGCACTTGTATGAGAAAGAGAGAGAGAGAGGGAGAAAGTGGAAGATATAGATAGAAGGAGAGAGAGAGAGACTGGGGGAAGAATTACATCCACCCAAAACCCAAATTTTAATGACTTACAATATGAAAGCTTCATTTTTTTTTTCTCTTATGTTGCACCTCACTGATGGACTATCATCAGCCCCACTTCTCTTCCAAGTCTTTATTCCAGAATCCAGGCTGGAGGCCATGCCTGAACTGAGGAAATGGTGTTCATGTACAACAGTTCTTTCAGCTTCTGCTCAGATGTGGCATTGCACATCCACTCATATGCGATTGTCCAAAGCATTTTTCTATTCTCTGGGAGATACTTCAAGGGGCACAACAGTGGCTGGGGATTGAGGGGGCTGTGAATAGACTTTCAGGAAAAAGGATCAGCTGTGCTAAATGCTGCTGATGAGTGCAGTAACACAAGGATGAGTAACTTGAGTAGCTTGTAGAGAGGTATAGGCCATTTGTTTCATGCCCAGGAACAAGGCAGGACCAGGAATCCTGGTTGAGATGCTGCAGTTTGGGCTAGTTGGAGGTGGGGGCAAGTTTTTCTCTCACTGCTGGGACTTACTCAGGTTAACAGATGGGACGTTGTGGAGGAGCTGGAGACGGAGGAGAAAGTGTAGAAGAGTTAACTAGGAGATGGATTGAGAGTGTTTGATGTGAGAGGCAGTAGAGCATGCATTGAACCTAGGCTGTATGGTTGGAGGGTTTTTTTCCAGCCATGTCCTGTCTGCTCAGGTTCAGAGGAGGTAGGAGGTAGATTGAACCAGCCACAGGTGATGCTCCATGAGTAAAGAAGGGTTGAGAGTCAGGAATTGAGGAGTCCAAGGCATTAACTGAAAAGATGGTTCATGGAATTTAACAAAGATGCGGACAAATATGAGGAGAGGAGGCAGTCAAGGGAGAGAGAAAGAGTAGGGTTGGGATACAGGGAATGAAAGTGAGCTCCTTAAGATGAATGGCTAATCCCACAAAACTGGCCAATTCCCATAAGGTGAACGGCTAATCCCATTAGTGCATTGTTGACATGAAAATGTCCTCACCAAATAATGAAGAAAAATTTGATTTTCTTATGTGGAAAAAGCAGGACCAAAAGCAATCAACCAAAATCGTATCTACTACCTGGCAGTCCATTAGAACACACTAAACACACACATAAAGAGAAAAATGAAGTATGTTAATTGTGAAACTTGTATCTCCAAAAACTGGAAAGCTTCTTGGCACTTAAAAGCACTTCTTGGCACTTGGGATTACTTGCCTGTAATCCCAGCACTTTGGGAGGCTGAGACGGGCGGATCACTTGAGGTCAGGAGTTCCAGACCAGCCTGGCCAACATGGTGAAACCCTGTCTCTAGTGAAAATATAAAAATTAGCCGGGCATGGTGGCGCATGCCTATAGTTCCAGCTACTCGGGAGGCTGAGGCAGAAGAATCACTTGAACCTGGGAGGCGGGGGCTGAGGTAGAAGAATCACTTGAACCTGGGAGGCGGGGGCTGAGGCCGAAGAATCACTTGAACCTGGGAGGCGGGGGCTGAGGCAGAAGAATCACTTGAACCTGGGAGGCGGGGGCTGCAGTGAACTGAAATCGTGCCATTGCACTCCAGCCTGGGCGACAGAGTGAGACGCTGTCTCAAAAAAAAAAAAAAAAAAAAAAAAAAAAAAAAAAAAAAAAAAGAAAGAAAGGTTCAATACCTACTTGTTGAATGAAAGTGGACGTGTGAATTCAAAGTTTCCGCTCTTTCACAGTGTTTTTTTTTTTTTTTTTTTTTTTTTTTGACAGAGTCTCGGTCTGTCGCCCAGGCTGGAGTGCAGTGGCACAATCTTGGCTCACTGCAAACTCTGCCTCCCGGGTTCACGCCATTCTCCTGCCTTAGCCTCCCGAGTAGCTGGGACTGCAGGCGCCCACCACCACGCCTGGCTAATTTTTTGTATTTTGAGTAGAGACGGGGTTTCACCGTGTTAGCCAGGATGGTCTCCATCTCCTGACCTCCTGATGCACCCACCTTGGCCTCCCAAAGTGCTGGGATTACAGACATGAGCCACCGCGCCCAGCCTCATTCAGTTCTTTATTACATTTGTAAAGGTAACTCTAACTCCGTGAGAGCACTTTCTCGCTCACCTCTTAATTCTTGAGCAAACAGAGAAGCTGTGCATGATAAAGCTGGAGAATTGGGTGGTGTCTTCCTATTAAGCTTACAGGAAAGCACTGGGCATTTGGAACAGATGTTGCATCTTGAGAGCCACAGAGTCAGGTGTGCACGTTAAAACGATGCTTCTAATTGTTGCATAGAGACAGAAGACAATCACAAAGATTCTGCCTTGACCTCCTTACCTCTCCAGTTCTAAAAACATTTCTCCCACTACAGAAAGCATCCATCTATGTGTTTTTTGCCTCCACGTGGTCCTATTCCTGAAATGCTCCTTCCAAGTCTGTACTTTTCCAAGAGCTACTATTTCTGGATCTTTTGCAGTTGCTTCAGCAAGAATCAGTTCTGGCTTCCTTGGTTCTACCATGCCAACTTTACCTTCTCGTCCCTCAGTGGGATGCTAGGGCTTGGGTTAATTCATCTCTCTCCTTCAAGGCGACATGAAGCCCCTGAGAACAGGGGCATATTTTTGCCCAGCCATTACCTACAATGATACAGGAGTCCTGTAATATTCGTTAGAGAAATGTGTCCACTGAACATGAATTTCCTATCCTGTTCCTTCTAAAAAGGATGCATGAGTTATCCTATATTCCCAAGGCACAACATGACTTTGTTCTGATATGTGCCACCGTGATCCTGTAGAATTTGTTTTGTTTCCAGTCCCTAAGAATAAATGTCTCTTAAAGTATTGTAGTCATTCACTCTACATTTTTATGAGTTATTACTGGCCCACCTACAACCATATTTCCTCCGAAATTCATCCATCCTCCTGGAATTACCTGATTCTGAATTATTAAGTGGTTCTCTTGGCCATTTGCTCAAAAAAAGAGCACACTTATTCCAACACACAGGCATTGTTTCTAAATTATTATTGTTTTTTCTTCCTAGAAACCATTTAGAGATGAAGATCCACTTTAGAACATGAACCCATTTAGTTTAGACTATAACAATTGAAGATATGGTGACTACTGTTTATTTCTGTTAGGGATATATTTTTTGTAGATTTCACAAAAGACAGAACCTGCTGTGTGACAGCTTATCTGCAGGACACCGATGGTTTGTAGGACGATGGTGAGGCTTTGTGACAAGGCAGAAATGTGGAAGGCTGGCAAGATTGTTTACTGAGCTTCCCCTAAGGATGGAATAATTCACCAATCCCACAACTCCTCCACCCTCAGTCACTACCAATAGCTGTGCCTCAGTGTTTTCTTTTTAATGATTGTATGTATTAAGAAAAAAATCCTCATATGTAGTGTTTAGTTTATCTGATTTTCGTTACTAAAATAATAAAGGAGAAAAGTAAATAATTCATATAAAAGTAAACTTTCTTATTCCAAGCAGGTGTATGTGTGCATGTTTGTGTGTGTGTGTGTGTGTGTGTGTGTGTGTGTGTGTGTTTGCCACTTTGATGGAAAGAGGCTGACTTTGCAGAGACTATTTTTTGTTAAGAACTTTCCATTAAATTAGAGCTTTAAGTTATAACACTGATTGCATAGGCCAGGGAAAATGGTAGGATGTGGCTTAAAAGGCAATCTCACAAGAAGTATGACTTTTATCTTATATTATAAACAACAGCACAACCTTGGAATTTGTCCCAATAAATTCCATAAGTATAAAATAAACTAAATAAGTAAAGTGACTAATATCCTACTAAGTCTTTTCCTTCACACATGCTTTTTTGCCTAAAGCCATTTAAAGTCTCTGAGGATTTAAATCTATGATTCTTTCATGGAGTAGAAGAAACCCAGAGAATATAGAAATTTAGAAAAACTTTAAGACTTATTGGTTTAACAGAAGTAGGCCGGGTGCGGTGGCTCATGCCTCTAATCCCAGCACTTTGGGATGCTGAGCTGGGTGGATCACTTGAGGTAGGAGTTCAATACCAGCTTGGCCAACATGGTGAAACCCCCTCTCTACTAAAAATACAAAAATTAGCCGGGCGTAGTGGTGCACACCTGTAGTTACAGCTACTTGGGAAGCTGAGGCAAGAGAATCACTTGAACCCAGGAGACAGAGGCTGCAGTGAGCTGAGATTGCGCCACTGCACTTCCAGCCTGGGTGACAGGGCAAGACTCCATCTCAAAAACAACAGCAACAAACAAAACAAAACAAAAAACCCAGAGGTAGATCTAATTCTGCAGACTGCAATCACTCAGTTATGGATGGATAAGTCAGTCCTTAAGTCCATCTGCTATTTGTGTATCGTGCATTTTTTTTTTTTTTTGAAACAAGCACGTTCCCACCTGGATTGAATGTTAATATTCACTGAAAGCCAGGGCATTGCAACGAGCCCTTAGGATGTTATAATTCTGGGCCATTTTTACAGTTCAGGATTTCAGATTTATTGCAATGTTGTAAGTTTTTAGTTTCTTGTCTTTCTCTAACATCTAGTAAGTTCCAAAACTTAAAGAACTACAGGTTTTCTTGATAAATACCTGTGTCACTACTTTTTATTTTTAGATTTTTCTTTTTTACTACATGATCTGAGTTAAAAGTTAAATATATATGAATTATTGTTTTGAAAAATATTACCTATAATAGTTTTTTAAAAGAAACTTTAATTTTAGATTTGTGCTAAATTGGCGAAGATTGTGTAGAGTTTTCCTTATACCCCACCCTCAAATTCCACTACTAGAAACACCTTACATCATTATTGTACATTTGACACTATTAATGAGCCAATATGTGTGCAATTTTTTACTAAAGCCCACCCATTCTTCTGATTTCGTTGGTATTTTCCTTCTGTCTTTTTTCTTTCCTCAAATCCTATCCAGGATCCCACATTACATTTAGCCGTCATGTCTCCTTGAGCTCCTCTTGACTGTGACAGTTTTTCTTCTTTTGTCTTTCATGACCTTAACAGTTTTGAGGAGGGCTGGTCACGGGATTGGTACCTTGTTTGGTTTGTCTGATGTTTTTCTCATGGTTATACTGGGGGGCTATGGATTGTGCAGAGGAAGACCAGAGGTGAAGTGCCACTTTCATTACATTGTATCAAGGGCACATACTAGCACCATGACATTGCAGTTGATACTAACCTTGATCCCATGGATGAGGTGATGTTGGCCAGATATCTCCAGTATCACGTTCGTCCTCCTGCACACACACTTTCTATACTGTACCCTGTGGAAAGAGGTCACTACGTGCAGCCTACACTTAAGAAAGCAGGAGGCCGGGTGTGGTGGCTCACACCTGTAATCCCAGCTACTCCAGAGGCTGAGGCAGGAGAATCACTTGAACCCGGGAGAAGGAAATTGCAGTGAGCCGAGATCGCGCCATTGCACTCCAGCCTGGGTGATAGAGCGAGACTCCATCTCAAAAAAACAAAAATAAATTAAAAAAAAAAAAAAAAGAAAGCGGGGACTATAATCCCCTCCTTGAGGGCAGAGTATCTACAGAAATTATTTGAAGTTATTTTGCATGAGAGATGTGCCTATTCTCGCCTACTCATTTATTTATTCCCTCATTTACATATATCAGTATGGACTCATGGATATTTATTTTATACTTTGGGTTGTAATCTAATGTGATGTTGTTTATCTGCATAGATTTTGTGTTTACGTAACTTTTTTTCAAATTCCTGAGGGATAGCTTTTTAGAAAATCCCTGTTTTTACTTTAGATCCAAGGATTACGTCTGCAGGTGTGTTACAAGGGTATCTTGTGTGTTGCTGAGGTTCAGGCTTCCGTTGATCCCGTCACTAGGTTATTCTGTGCCCAGATAATGAGCACAGGAAGTTTTTTAGTCCTTGTCCCCCCTCTGCAACAGATTGTAGGAAATAATCTGAGACTGATCATTTTTAATTTTCAAGCACTGAACATGCAGTTATTTTATCTAGAAGGTAGACCAGCAAAACAAAATTATATTTGACATTTTAGCATATAAGTATTTTCTAGTTAACTTTGACATACAAGAAGCCAGGTTATGAATGTATTTGTTCATGACTCTAGCTTGTTTGGTTAAAATTATTCTCCTGCCAACCAAATGCTTTTTTGCTACCCTGAATATTTAAAAAATTTTTACAATATTTCATCTTTAAGAGCTATAAATGTATGTTTTAATATCCCAGGGTAAGATATAGGGATATTTTTTAGTCTGTCGAGGCTGCTATAACAAAATACCTTAGACTGGGTAATTTATAAACAATAGACATTTATTGTTATTATTATCATTAAGACAGGGTCTCTTTCTGTTGCTCAGGCTGGAGTGCAGTGGCTTGATCATGGTTCACTGTAGCCTTGACTTCCTGGGCTCAACTGATCCTCCCACCTCAGCCTCCTGAGTAGCTGGGACCATACGTGTGTGCCACCATCCCTGGCTAATTTTTATTTTTTTAATTTTTAGTAGCGATGAGGACTCACTACGTTGACCAGGGTGGTTTTGAACTCCTGGCCTTAAACATTTCTCCTGCCTTGACCTCCTAAAGTGTTGGGATTACAGGTATGAGCCACTTTGCCCAGCTAACAACACACATTTATTTCTCATGGTCCTGGGAAGTCCAGGATCAAGGTGCTAGCAGATTCAGTGTCTAGTGAGGGCCCATTCCCCCAAATGGCATCTTCTTGATTTATCCTCACATGTTGGAAGGGACAAGGTGGAAGGGCCTGCAGCCTCTTTTATAAGGACACTCATCCCATTCATGAGGGTAGAGTTATCATGTTGTGTATTGGATTTCAGCATATGAATTTTGGGAGGACACTACCATTCAGACTATATAACAAGATACATTAGGTTTGGGGTGTTCTGCACTTGAGTGAATCTATGTAAGCCCTTTCACATATTTTTACTTTCACTGAAATAAAACTAAATAAGGAAACCAATGCTATCCTATATCTTAAAATGAGAATGGTTTGTAACAGCTCATTGCCTTGCATCATGGTCTTTTAGGGTTAGGGTTCGGGTTAGGGTTAGGATTAGCTTCGCTTTGCTGGGCAGAGTAGGTATTTCCGCCTCGAACCACCTCTAAGGGCTTCAGCTTTCAGTAACGCACCTGTCACTTCTAATGCAAAACCTTGAGTCCTCTGTCTGTGTGCAGATTCAGGAACAGGTTTGAGGTCTAAGAATTTTCTTATTATTGCCTTCCATTTCAATTTCTAGTTCCTCCAAAGTCCTTCACAATGATGACCGAGAGGAGACACTCAAAAATTTGTTAGCCAGAGTCTCAAAGTACATAGAAGCTGTTTCTCTTGGGTGGATATTACAAGTGCCTCTACAGGCAACTGCATTTCTTTCTCTTTCCAGGATTTTTGCTTATTGTCCAGATATGCTCCTCCTAGTGAGAGGGACACTTCTGATTTTTCCTGCCTCCATGGAACAGGGGCTTCAGAGAAGAAACTCTCTACAGCCCCTTCGTTCCATTAATAATTTATAATTAAATGCATTTCCAGCATGAAGGCTGCCTAGGAGTAGAGAAGCATATTAGAAGAACCAATCTGCTGCGTATCTGCTTATAGGGTTTGAGCCCAGTCAAGGAGGGATGCACAGAAACTCAGGATTCTGACAGCCCAGCCCCCTTGCAATTGGGAGGGTCGCCAAATTTCTTTCTTGCAAGGGGTACTTACTGTCTGTGAGTGGGAGCCTCTTGTGGATAAGGAGTGAGGGCAGAGAGGGAACAGCAGAGCCCTGGGAAGTTCTTTCCACTTGACTCTGAGCGTCTAGACAGCAGCCTGCCCCCACCCCCTAGATTGGCTTTGTACCTGTGAGCAAAGTTTCTGACTGTGCCATACATCTCTGGAATACATTTAGTTGCTAATGGAGATATTACTATAATTCCACATATGTTTTTAGTCTCTCCTTGGGGCTGTGCCCTTCTGTGTGGCTTGGCAGAAGAGAAAGGAGAGAAAGATTATACATGGCAGCCTTGCTTTGGAGGGAGTGAAACCTGTGATTTTCCTTTTCTGTGTCAGGAAAGCGTTTTTCTGCTGCTTGACTAGCCACCTCCCAGGCACATTAACCAGTCAGGTGATGCTGACATTTGTACCCCCTAATCTGGCTTATTTCTGAAACCCTCCCTTTGAGCCCTAACTGCTATAATTAGGAGACTGGATCCTAACAGGTTTGGAAAAAGGTTTGCAATCTCAAAATAAAGTAGTGATTTTGAAAGAGAAATGTATAGTAGAGTTAGCTATGGGGTTTGCACATTCTACATTTATGTTTGTTTGTTTTTATTTTTTCGCTCAGACTGCTCACAGATGCAGTGAGCACACCCAAATGCATGTGATCAATGCATGTCTGACTTCTGCAGCTATGGAAGGTCTGGGTTTGTAAGATCACTGCTGTAGACCCTTGTTTGACCTTTTTGGATTGCTGGATCAGAAAGTGAGAGATTGCGAAAGTTTTCTTAAAAGAACAAGTCAGTGAATCAATTCATTAATTCTTTTGTTCATTAGGATTAGTTAATATACTGCTACAGTAAAACCTTTTGTTATTGTCTGTAATAATAAAAGTTGGATTATGGCATGGCTAACCCCAATCTCCATACAATCTGCTCATAGTTTTGACCTCATTCTAATATAACCCTGTATTTCACGTGATTGAATGTTTTGCACCATATTTATAATATTACATCCAGGTATTACTTGGTTTCTGAAGGTTTATAAAATTGTAAATGCAGTACATAGGGTATTAGAGATTTTGTTGTTTTATTTTTTTAGAGACTGGGTCTTGCTCTATCAACCCAGGCTGGAGTGCAGTGGTGCAATCATAGCTCACTGTAACCTTGAACTCCTGGGCTCAAACGACCCTCCACCCTCAGCCTCTGGAGTAGCTTGTATTATAGGTGCATGCCACCATATCCGGCTAATTTTTTATTTTGATTTTTGTAGCGATAGCATCTCAGTGTATTGCCCAGATTGGTCTCAAAATCCTAGCCTCAAGCAATCTTCCTGCATTGGCCTTCCAAAGTGCTGGGATTACAGGTGCCAGCCACTGTGCTTGGCCATTACCTAGAGTTTTTGTTAGAGATAATGAAATAAGAATGAGATTAAAATGAGGTTAGTCTCATGCTGCTTAAAACAGTGATATGCTTAGGAGCAGCTGCAGGAACATCTGATCCAATCTTGGAGGCAGCCTGGAGGGCTTCCCAGGGGAAGCACAATGTAGTCCAAAACCTGAGAGATGAGCAGGGATTGACTAACTAAAGAGCAGACCTACACACCAAATTCTGCCATCAGTTCCTTGCATGGCATGGAAAATTGATTTCTACAACTACGCAGTATTTTTCTTCCTTTTTTTTTGAAACAGATTCTCGCTTTGTCACCCAGGCTGGAGTGCAGTAGAGCGATTTTGGCTCACTGCAGCCTCGACCTCCTGGGCTCAAGTGATCCTCCCACCTGAGCTTCCCTAGTAGAGTAGCTGGTACTACATATGCACACCACCATGCCCAGCCAATTTTTTATTTATTTATTTATTTATTTTTGTAGAAACAGGGTTTTGCCATGTTGGCCAGGCTGCTCTTGAACTCCTGAGCTCAAGTGATCAGCCCACCTCGGCCTCCTAAAGTGCTGGGATTACAGGCATGAGCCACCATTTTTATTTGGTATGTGTGCATTCATAGTTATTCTACAAAAAATAATATTTAATAATAATTCACAGTATCCTGCAGATTCCAAAATAAAGTAAGCTTAAGTTCTGTTGGAAAATGAATTTCTGTGAGAAGGCTTTGGTGCTTTGACTTGAAGCTGACATCAACATTAGTGTTGGGCATTTGGCTACACACCTGTCACATTCAAAAGCCAATTCACTTTGAGTCTTTATTTTGTTGGCAGTAAGGGCTGCACATTTCGATCCACTGTGTATTTTCCTAGCCCAGATTCCACTCAAAGCAGAGGTTTAGAGAAAACCCTTGTTTATTGCAAATATTATGCCAAAAATAGGGATGAGGAACCAGCACTGTGTTGTGGGAAGGAACGAGAAATAATCACTATTTACAATAGCCGAGTTGTGGAATCAACCTAAGTGTCCATCAACAGTGCATTGGATAAAGAAAATGTAGTACATCTACAACACAGAATACTAGGCAGCCATAAAATAGAATGGAATCATGTCCTTTGCAGCAACATGAATGTGGCTGGAGGCCATTATCCTAGGTGAAATAACTCAAAAACATAAAATCAAATATAGCATGTTGTCACTTATAACTGGGAGCTAAACAATGGGTACACATGGATATAAAGATGGAAACAATCAACACTGGGGACTCAAACAAGGGAAAGGCTGGGAGGGGGTGAGGGTTGAAAAATTAACCTATGGGTACAATGTTCACTCTTTGTGTGATTGGAACCCTAGAAGTCCATATGTCACCAGTGTGCAATATACCCATGTAAGAAACCTGCACATGCACCCCTGAATCCAAATTAAAATTTAAAAACAAACAAAAACACAAAAAAGTGTATTGGCCACAGAGGAGTGACTGCTGCTTGACCCAGTGAGGTTGTCTGAAAACCCTTATGTTATGTGTCTCCAGACCACCTTTACCCGGTGAAAATGGAGGACCCATATTCACACCATCTTTCACCTCTTATTAGTTTACTGGGGGTAACCTCTCCAGGCTGCTTGGGGAGTGCTAAGTAGGTTTTAGTGTGCATCCACTGTGAGGCATCAGAGAAACTTCAGGAAATCAAGAAAAAGGCAAGTTTGCAGGTATGAAGTGAGGCTGCACCTGCGTGAAGCTGGCTGAAGTCTAGGCAGAGCAGATCACCACAAGAGCGGCTGGAATAAGCCATGTGGCCGAATGGCATCCAGCACAACGATCAAGTGAAACAGAGCTCCTCCAGCTGTGGTAGAACTAGGGCCAAAGTATGTGAAAGTGTTCAAAGATTCTTCGCATTGAATTCAAGCTCATCATTGTCCACAAATCAATGAGACCATGTCTATATTGGTAAAGAAAGAATAAAGCATAAATTCATATTTCAATTTTTAGGTTATCTGAATAAATGAATTTCAAGAGTGCTTAAGGTTTTTGCTAGATGTTTGCAGGTTTTTGCCTGGAGAGGCACAGGCAGTTCTTTGTCCTATCATTCTAGCCTTCCACTTGTAGGGATTCCCTGGAAAGTTGACATAACCGCTGATTCCTAGTTCTGTTTTGTGGGAAGTATCAAGATTAAGAGACCCTCTGGGTGAACAAGATGTCTTTCAATAGATGAATGGGTAAATAAACTATGGTGTATTCAGACAATGGAATATTATTCCATGCTATAAAGAAATGAGCTATTAAGCCATGAAAAGACATGGAGGAAAATTAAATGCATATTACTAAGTGAAAGAAGCTGATGGGAAAAGGCTACATACAGTATGATTCCAACTATAGGACATTCTGGAAAAAGCAGAACTGGGGGACAATAAAAAATCCATCATTGTCAGAGTTTCGGTTGGGGATGGGGAAAGAAAAGATAAATAGGTGGATCATAGAGGATTTTTATGGCAGGGAAGATATTCTGTGTTATACTGTAATGGTGGATGCAAGGAGGTTCTTTTTGTCTAATTAACTGTTCACATTCATCATAATTGATTCCATACAGTATGCATGGATTTTCAGGGTCCAAGTGTTAACCAACTTCAGTGGACTTAAACCACTCTGTAAATGGGGTGCTCTTTAGTGTTTGTTTTGTTTACTGTTCTAGGACTGGTTAATAGAAATCAGAGGACATACAGATCCAGAGTCCCTTATCTACAATTTGAAAGTCAAAAACAGTTCAAAACTTTACAGTGATATCAAAACTCATTTGGGGGCAAAACCTGATCTGACAGATGACTATTTGTGTTCTTTCTTTTCCACCTCAGGGTGGACATTTAGATATTTTCCTGCAGGAATATTAATGAGTTTGATTTGGGAGTGATGTTCCATATTCCTCTGAGGGTGCTGCATAAAACAGATGTAAAAAAATTAAAAAGTTCTGAGTCCCCTTCCTCTTGTCCACAAAAGCATACTCATTCCCAAGGGTTTCAGATCCCCATTGGTGGATCTGTGATATCAAAGGTCTCATTGATAATGTTGGTGGTCAGTGGAAAATAGTTGTGTGGAGAGAGATGTGTTAGTCTGGACCTCATGCAATGACTGCAGAAATAATTTTATGATTTCCAAAGAACAACAGACAATCTAACCACCTCCCTTACCTTTAAAGACTGACATCTGTGTTGTGTTCATGGATGATTATGCAAATCAAGAAAAGTGGCTTCCATCAAAATAATGTCATTTCTTTTTGGAGAAAAGAGCCTGGGACTGAGTTGTGTTATGTGTGCAGTTTGCCAGCTAAACTCCTGGCTTAATGATTGGGATGGGTTTCCAAGGGCTGGTTCTGAGACTCAGTGGCAGTTAGTTAGGTGGTAATTTCCCCATTAACATTAATGAGAAATGAAATAAGTTACTTAAGAAAACGTGCTAGACGATAGTCTCTAAGTACTGAAAAGTAAATGAACCCACCTACGTTTGTTCACATAAAATTTCTTAGTATATTTTAAATTTGCTAATCTAATGTACTTTTTTTTTTGCTTGTGCTTTAACTTTGTTAAATTATGTCACGTAAAACATTTTATTCCATATTCTAAATTACATAAATGTGTCACACACAATGTCATGAATCAAGTTTGTCTAAAGAGGAGATAGGCCAAGGCAGGTGGATCACTTGAGGTCGGGAGTTCAAGACCAGCCTGGCCAACATGGTGAAACCCCATCTCTACTAAAAATACAAAAGTTAGTGGGGCATGGTGGTGCACACCTATAATCCCAGCTACTCAGGAGGCTGAGGCAGGAGAATGGCTTGACCCTGAAAGGTGGAAGTTGCAGTGAGTCAAAATCATGCCACTGCATTCCAGCCTGGGAGACGGAGTGGGACTCCATCTCAAAAAAAAAAAAGGAGATAATACACTTTCACGTTTGTAAAATAATGTTGATTAAATGGTCTAATGTGATTTTATCTTGCTAATCCAGTTACCGTCCCAGTATCTGAATTATGATAACAGTTTACGCAGCATAGTTTTCTAACAGTTTTGGTTCCATCTCTGCTATTAAATTCAGGCCACTGGATCTGTTTGGTTCAACTTGGATTAGGGTGTGAGGTTCTGTTTTCCTACCTCTAACTCCATATACATTGTCCGTGCTCCTGACCTTCCATGCAGGAGGCTTGCAGGTATCTCCTTAATCTGTCTGTCATCTGTTTCTTTCTGCCATCTCAGGGACTCCTGATCTTTCCAGACTGCCCATCCTCTCCTGTCCCTTTGACTCTTCCTTTTTTGTTCACTTTCTGTAACTCCAGTCTGATCATCTAAATAGTCTGAGGGGAAGATGAGGTACTGAAGGCACTCTTGTGAGAATATTTCTCAGGTTCCTAGGTCCAAGTTTCCGTTGCATCTTGGTTTCTATTTCAGTCTGAGCAGAGAGAGAGAGAGAGAGAGCAAAAAAGATCTTCAGGATAAAAGTGAGAGAGAGAGAAGATGGAGAAATAAATATAAATGAACAACTGATAAATGCCTTGAGCTATAACTCTGCCAAATGAACACAGAAACTCATGTGCAGTTAGATATTATCCACCTGAGAATGTAGTTGATAACATATTTCATCATAAATAATATCGTCTAAAGCCCTTACTTGGGAAGATTATGAAGCAAGCCAAATCTTATGCAGTATGTCCTTCTGTTCTCTTGACAAGCATAAGTTTCTATTTCTGTATTGCTAGAAATTTTTAGTCACATGCAATTCCAACAGTGCTTTAAGCTGGTTATTACTAAGTAGAAGGTAAATGTTTGATGATGGAAGAATTTGCGGTGGAGGTGAAATTTAGGATAAATATTAGCAACTTTGAAAAGTAAGGTGTAGATCTGTGCGGTACCAGAAAACATTTAACAGATTCAGAAGTTAGTTTATGTGTACCTATATGTGCACACACATACACACACAATGCATGCACACTTATGCAAATCACACACACATGCCTCACGCACAAGTGCAACACTCAGGTGCACCCAATTGCACATACGTATTCTATTACTATTCTTTGCAATGCTTTGAATGCTCATCATGTACCACAAAGTTATGGTCTAATTCATAATACCATAAGGTGCGTGTGCTTTAGAGATACTGTGTATTTCCTTTCAACATCGAACTAGTGACTATTAATGTTTTAAAATCAAATTTGATAACATTCTGAAATAAAATACTGATGTATTAAGTACCAATGCGTTGACATCAGGTTTCATAGGTGTTGAACTGTAGCGAGGAAAACAGTTATCAGGTGTCCTACTGTAACTCTACCCAGCAGGAAAGCTCTATGTAATGATGGTAGAATATCCAAATGATGGTGTCCACATCTGCACAGGTACGATTTGAGATTCACTGACTTATTTAGGAGGATTCAGTAAAATTTCGCAGATGTTGTTATGTAGTAATATTTGGCTCATTCATATTCTGCACTCCTAGACATTGCAGAAAGACATGCAACTGTGATTTCCATCTCATCCCTTTCACCCTATTTTGAAACATTTAGTTATGTCTACTAGTTACCCTAAGTTGTATTTTTTACCCTCTAAAAAGGAACAAGAGAAGTTGGAATCCATCCCAGCTTTCCTTCCAGAAAATGGAGGGGAGGAACAATTGGAATGGAGAGGAACTCCAGGGAGAAAAAGACAAAAGGCACATGAGTGAGTTTGTCTAGGCTGGGAGAGTGGGCGATCACATGAGATTTGTGAACTAATTTTGTTCTCCTTCTGTTTCCACTGATAAGCACTTTATGAGTGCCACCAGTGTAAGTAAATATTAAACCTCATCTCAATTAGTATCTACTCTTTTCCAAATATATGCTTATGTCAGAAAATGAGCAGTAGAAAGCAACCACAGGATACCACCTGCACACCCACGGGCTGAGCATTGCATACTTTCAAGGAGTGCTGTTGTGTTTTCAAACTTAGTAATTTCCCAAAACAGAGAATTCACAGCTTCCCTAATCACCTTCCTCAGAACCCTGAATCTTGTTAATTGAGTCATTTTTCTGATGATCATGTACTCATACAATTGACTAAATGTCTCACTATGCCTTCCTGATAAGTAGTGTCTCTACATGTGAAGTATCTATTTAATCTATCTACCCCTCTCTCTCTATCTAATCTGTTGATTTCTTATCTATCTAATTTATATCTATCATCTCTATGTATCTATGTATGTATGTATGCATATATGTATGTATCTATATATATATCGATCTATCTTATCTATATGTATCTATCATCTCTATGCATCTATGTATCTATCTGTCTATGTATGTATGTATGTATGTATGTATGTATATATCTATCAATCCTCTCTCTCTCTCTTAGTTCAGCAAATTACTTACAGGTTTTTGTTATGTAACTGAGCAAAATTATATACACACACATAAGAAGGCTGGAAGTTCAAGATCAAAGTGCTTACAGATTCAGTGTCTGGTGGGGACCCACTTCCTGATTCATAGACAGCGCCTTCTCACTGTGTCCTCACATAGTGGAAAGGGCAAGGGAGCTCTGTGGGATCCCTTTTATAAGGGCACTGATCCCATTCATGAAACTCCACTGTCATGACCTCATTACCTCCAAAAGGCGCCCACCTCCTAATACTGTCCCGTTGGGGATTAAGATTTATATATTTTTTCTTTTTAATTTCTAATTTTTGTGGGTACATGGTAGGTATATATATTTATGGAGTACATGAGATATTTTGGTGTAGACATGCAATGCATAATAATCATATCATAGAAAATGGGGTGTCCATCTCCTCAAGCATTTATCTTTTGTGTTACAAACAATCAAATTATATTATTTTAGTTATTTTAAAATGTACAATTAGGCCAGGCACGGTGGCTCACGCCTGTAATCCCATCACTTTGGGAGGCTGAGGCAGGCGGATCACGAGGTCGGGAGATTGAGACCAGCCTGGCTAACACAGTGAAATCCCATCTCTACTAAAAATACAAAAAAATTAGCTAGGTGTGGTGGCGGGCACCTGTAGTCCCAGCTACTCAGGAGGCTGAGGCAGGAGAATGGCGTGAACCTGGGAAGCAGAGGTTGCAGTGAGCCGAGATCATGCCACTGCACTCCAGCCTGGGCGACAGAGCGAGACTCAGTCTCAAAAAAAAAAAAAGTACAATTAAATTACTATTGACTATAGTATTGACTATAGTCACCCTGTTGTGCTAGCAAATACTAGGTCTTATTTATTCTTTCTGACTATAATTTTTGTACCCATTAACCACCCCACTTCCCCACATCCCACCCCCACTACCCTTTCCAGTGCCTGATAACCCTTTTTTGACTCTCTATGCACATGAGTTCAATCTTTTTGATTTTTAGCTCCCACAAATAAGTGAGAACATATGATAACAGTCTTTCTGTCCCTGGCTTATTTCACTTAACATAATGATCTCCAGTTTTATCTATGTTGTAAATGACAGGATCTGATTCTTTTTTATAGCTGAACAATACTCCATTGTGTATATGTACCACATTTTCCTTTATCCATTCACCTGTTGATGGACAGTTAGTTTGCTTCCAAATCTTGGCTATTGTGAACAAAGCTGCAACAAACATGGGGGTGTGGATATCTCTTTGATATACTGATTTCCTTTCTTTGGGGGTTTGGATATAAACATATGAATTTTGAGAGGACAGAACTTTCAGACTATAGCATACTGTACCATCTATCTATCTGTCCATCCATCTGTTTATCTGTCTCCCATTCCTGAATATTGCATGGCATATTTTGTTAATTATTTCCAATGTCATATTGAGTTTTAAAGTAAGATTACATTTCTGAGAGGCCTCACGTGGGGGCATCCTGAAAAGTACATTCTCTTTATAGTTTAAATGTTTTGGTTTTTTTCTTTATTTTTTTCATATTTAATTATATTTCTTTCAAGTGACTCCTTTGGGAGACATGATTTTCCTACCTCCTGGGACTGCCACAATTCCCCTGCCTCTTGGAATGCAATCGATCTCTAGTCTGCCTCAAGTATAAAGATGATATTCATGTTGATGACATTGAGAAGGATGAGGAGAAAGGAGTTGATCAGAGATCTATATTCATGGTATATATGTTTATCGTATATATATTTATCTGCTTATCGTCTTCAGAATATAAACTCCAAGACTGTGGGTCTTTGTTTTCTTCAGTACTACCTTGCAGAGTCTAGGCCTATTTATTCAAAGCTTAATATTTGTGAAGTGCATGAATGAATAAATGAATTCTAATGTTATCACTGCCGTTGGTATGGTATCTGTTTCTCTATCTGTATTGTCCTCTCTACTTTTCATTATTTGTTTAATTCCCACTCATTGAGACAGATTGCAGAAGATTCCTTTGCCAACTACTTCTGGGTAGAGATAAATTTCCCTCCACGGAGCTCCCACTGGACTCTACCTGCAGCTATATGTTATCTTGTATTTTCCAACACTCAGCTGTACCACATAAGACTTGATTGAGTGAAGACCCTGACTTAGCTTTGCATAAAACCAAAGTAAATGCTTTCCACACATAGCCATTCACAGACATTTTCACATTTTATACAGCAACTGATGAACTAGGCTAGTGTTGGGAACAGGCCCCCTAAAATCTGGCCATAAACTTGCCCCCAAACTGGCCAAAACAAAATCTCTGCAGCACTGTGACATGTTCATGATGGCCATGACCCCCATGCTGGAAGGCTGTGGGTTTACCAGAATGAGGGCAAGGAACACCTGGCCCACCCAGGGCGGAAAACCGCTTAAAGGTGTTCTTAAACCACAAACAATAGCATGAGCGATCTGTGCCTTAAGGACATGCTCCTGCTGCAGATAACTAGCCAGAGCCCATCCCTTTATTTCAGCCCATCCCTTTGTTTCCCATAAAGAATACTTTTAGTTATCTATAATCTATAAAAACAATGCTTATCACTGGCTTGCTGTTAACAAATATGTGGGTGAACTGTTTGAGGCTCTCACCTCTGAAGGCTGTGAGACCCCTGATTTCCCACTCCACACCTCTATATTTCTGTGTGTCTTTAATTCCTCTAGCGCTGCTGGGTTAGGGTCTCCCGGACCGAGCTGGTCTTGGCAGGCTATAAAGACATTTTCTACTGGCTTAACAGAGAAGAAAAACAAAGCTTAGGGAGACTGATTATGCAGAATTTAATTTGCAACAAGCAAAGACAAGTCTATTGACTTCAAATGGACATCATCACATTGTCATCTGATAATTTTTCCAGCATCCTTTGCCTCCTCTGTGTTAAATTATAAATTAATGCTGATTTATACAGTTCAGTTCAGCTTCACAAATATTTAATGAGCACTTGCTGTGTACCAGGTATTATTATATAAGTAGTTCTTTATGGTGTAAGAATGGATAGTAGATACTTTTTTATCCATTCAACTTTAAAAGGTTGATGCCTAGTCATAGATACCAGGAAACACTTAAGTGAATGAGGACAAGTTTTCTGCTGTCAAAGAGAGAGATCAGACACCAACTAGAGTCCAAGAAAGAACAAAGTAATTTTGATCAACAAAACTCATAGAAGAAAATAAGCATTCTTTGTTGTTACATATACTTCAGAGCCATTTTAGTGCTCAAAGTTTGATAGAAATTGATACACAGGACTTGCTGCTCTGAATTGGCTATCCCAGAATATTCTACGAGCTACAACCAGACCTGACATTAACCTGTAGTTACTTGTGGTTTATTCATCTATCCATCTAAATGTTATGAGCATCTCCTATGTACTCTTCATGGTACTAGACTTTAGACATTGAATACGGAGCAAAAAAGACATAGTTTCTTATTTAATGTGGCTTATACTCTGATGTAGCATTTCTTCACCAGGGGTAATTTTGCCTCAGGGGACATTTGGCGATGTCTAAGGACAGTGTAGGTTGTCATGACTGAGATTTGTTGCTGATGTCTAGTGGGAAGAGGCCAGACCCCCTTCACAATAAAGAATTATCTGACCAAAAAAGGTCAGCAGTGCCAAGGTTGAGAAACTCTTCCAGCAGTTGAAGAAAAATAATCATCAGATCACCCACAAGTATAATTACAAACTGAAATACATGTTAGATGCTGGTAGAGCTGGTTTCCAAAGTTTCTGATCCAGTTGTGAGGATACATATTGATATTGAGAACGGGCGGTTGAAGGGGCAGTAGTAAGTTATTAGGGTAAGAAGGTCTTGGTGAGCAGAGGGACTTTCATGCGAAGACTCCAGGGCTCGAAGGAGCCCAGTGCAGTCAGGATCTGAAGTGACAGGTGTGGCTTGAGAACAGCGGCAATGGGGAGTTAGGCAGGAGGGGAAGCTGGAAATGCAGGCAGGGGTAGACAATAAAAGTACGCAGGCCGTTTATATTATACAATCCTGTAGACTTCTTTCTTCTTTCATTCTTGATACTTTTCTATAATAACATTCAAGCATTGGATCAGCACCCTTTGTTGTCTTCTGTCATGTAGCCCAAAGGTTTACCTTGGAGACACAAAGGCAACTAAGACAATGGTTTCTGCACTAGGGAGATCATATTCTCACTCAGAAGACATTTGCAGGGTGTGATTAGTGAGTCTCACATACATGTCAATTTCTTCCTAAGACCTTGTGCTTTTCTAGTTTTTATTTTTTTATTATTATTTTTATTTATGTATTTTATTTGAGAGAGCCTCGCTCTGCCACCCACACTGGAGTGCAGTGGTGTGATCATAGATAGCTCACTGCAGCCTCCAACTCCTGGGCTCAAGCAATCCTCTTCCCTCAGCCTCCCAAGTAGCTAGAACTACAAACATCCACAACCACACCCAGCTTATTTTATTTTTTGTAGAGGCAAGGCTGTCTCTACAAATTCCGTTGCCCAGGCTGGTCTCAAATGCCTGGGCTCAAGCGATCCTCCGGCCTGGGCCTACCAAAGTGCTGGGATTCCAGGTGCGAGCCATCGCGCCCCACCCTCTAGTTTTTAATTGGTTTATTTTCTTCTCATATTTCAGTTGAGCATTATTCATTTATTGCTGTTGAGGTTTTACTTTTTTTTTTCTTCCCAAAGGTAGATTGTAGACAGCTCACCTTTGTTACCAATTTGAAATGCTAGATGTTAATTCTTAATGTTGTAGCTGTAAAGGGCCATGATTTGAGGACGTGTTATTTTTTTAAGCCTGAGTTTGGATTGGTCTGAGTTGAATGCAGTTGCTAAGCCATCGAATGAGGGAGTGTCCCTGAACTAATGAGTGACATGGACCTTTTCTTATAGGTGAGAGTCCATTTGTGATAAAGGCATTGTTTTAGGATACATAAGGGTCATGGTGTATATTCTTAGCAAGTGTTATGAATACATTCGATCTATTTCTTTTGAATTTTAGTGTTTCTCTACTCTCCATCTTACTAAACCAGGTGTCCCAGATTTCGGGTTCAGCACATTTGTGTCTGGGTTCACATAGAGGGACTAACTAGGTGGAGTTTAGGGTAAGGGGGTATTCAGAGTCCTGCCCTCCTGCAACCACAGCAACACCCCCAAGTCTCTCTCATTAGATTGTATTTGTTCTCCTACTTATGTTCTTTGGCCTCTGCTATAAACATTTTCAAAAAAGTATCCAATGAAAACAATGTTGTCAATGACTGTCTTTAGTAAGTCTGTAGTCAGATTCATATCTTTAAAATATGTACACTGTGTGAATATTTCAAAGTATGTATCATGAAAACAAATAAGGAAAAAAAAAAAAAAGCCAAGAAAGCTGAGATGGCTCTATTAATATCAGGCAAAGATACCTTCAAGATAAGGATTATTTCCAAAATAAAAGAGAGACATTTCATAATGATACAAGGAAGAATTCACCTAAGAGAACTAATAATGTTAATTTGTGTACACCTAATAAGAGAGCTGTTAATTATACAATTAGCAATAAATGCAAAGAAAGACTCATCAATAATGACAGTTGGAGATGTTAAGATGTTACCACAATAGATGAAAGATGAAGATAGAAAACACACACACACACACACACACACACACACGATATGAAAATTTTCAACAGCACCATCAATGTCCTTGGCAACTTCGTACTTCGAGTCCAACCTCCCTTCACAATCTAATACAGAAACAAACAACCCATGATTTTTCTGCATTTCGTGGTTAGGTTCCCTGTGGCTCAAGGCCTCTGGCGCAAATGATGTTGTCTTTTAGATTTTCATGCTAAGAAGATACTCATGTTCGTATGTGTGTGCTTTTTCCTCTATAGCATCCTTAATGTTGGCCTCCAGATGAGAGTCTCTGACAATGGGGCTTTAACATCAAACAGCCAAAGTCTCTCAGCGAGTTAACCTCTTTGGCCTTAAATTTCTCACATAATGACATACAACAGTCCGCTCTTCTTCAAGTGGCCTTTGAGGAGTCTAGGGACACTTGTGAATTCACTTCCACAACTCAGCTGCATTGCGAATTCAATTATTGTGCTGGGAGATGTTGTACCATTATTTTTTTTTAAAGGTGCATATTCTAAAGGTTAATCTTGAGGCTATCACATTAAGGGTTAACATTTTATCGGGGGCATTATAGAGTGCATTTTTGATGGCTGTGATTTCAGATAACAAGCTTGTTGTTTCTATTTTTCAGCTCTAGCTTGGCCTCTAATCTGTAGGGAAGGCTGGTTCCTAAATGCAGGAAATGAGGCTCAATAGAACATGAAAAGCCAGTGTTAATACACCATTCAATCTCAAGAAAGAGTGGGAGGAAGAATGACAGAGCTGTTTTTTGACAGATGAGTGGTTAGGCATCCCCCTAGCTCTCCAAGTCACCACTAGGATGAACTTTCAGGATGCAGTGTCCTGTGGAATTTGGCTCTGAAACATAACTTCTTCATAAGGCAGATATTGTAACGCAGTTCTGGATTTTGTACCTACAGACAGCTCTGTGTTATGGTAACTGTTTTCTGTTGGCACAACAAACAATTAGTTAGCTTCATGCTGTAGAATATTTCCAGATGCCCTGATACTCCAAACCATTGGTCATTGCAGCCTCCATATTCAGATGTAGCGGCTATAAACAGGTGATGCATGCATCCTGGCCAGGGACCATTTTGATTTTTCCACCTTTTTCTTTCCCAAATTCAGGGTTTGTCCACATTAGCACTATTAAAACTTTTGGGGCGCTTCCTGTGCGTTGTAAGATGTTTAGCAGCACTCCTGGCGTCTACCCACTCCAAGTCTTTAACACCTAACGCCCATCCTTAATTGTGACAACCAAAACTACCTGCAGGCATTGCCAAGTGGCTCCTGAGGGGGCAGCATTGTCTTCATTGAGCACCAGTATGGTAATCCTAGCCTAATCTATTGTGTTACCTTATTGTTCCTTAACATATATGGGGTAGAATCAGAATTACAGGAACGTGAATTTCTTTCAACAATTATTTCTTTACAATTATGTAATAAAATCATAAAAGGTAAAACTGTATCTTTTTAGAAGCCAAGAAGCAACAGTTTATGAAACAAAACCTCTTTTAGTATTTCATATTAATCAATAGATATTGTGGAAAGGCTAGTTCTTCTTTAAGGTAACAGTTGCTTAAGAGTTGAAGTGCAGCTTATGAGTTTTACAAGCCCTGATTTATGCACAGCTTGAGGCATTGTTGTTTTGCAACTATTGTTTTCCAGCAGCACTGCTATTTTATAAAAGCATGTATCAGCAATAGTATAGAATTGCATATATGCTTCAGAGTCAATGCAATCATTAAATAGCATGCAATCTGAGTAGAGTCTACCCAAAGCTGGAATTCAGAGCGCATATTTATGCACTTAGCAACATTGCCATAATTACACACACACACACACACACACACACACACACACACACACACACACACGCACGCACGTACTTAAAGCCTTAGCCATTTAAAAATAGAATTCAACAACTAAGGCTCGTACACATGGAACTCTTTTCATAGCAGGATTTCCAATGTGCAAATTTGATAAAATTACTCTTTTTAAAAAAAAAATTGCTGCAACGTTTTTCATTAACACCATAAACATTTACACATGATTCACCCCAAATTGCACCCTAGATGTATTTACCCTGACTTGGCAATTTCATACTTCATGTCTCTACTTCCCTTCATGCTTCAATACAGAAACAGACAACCGATGACTTTTCTGTATTTCTGTGGCTCAAGTCCTCTGGCCAACCTGATAAATGGCTTAGGCTATTCGATAACCTGCAGCAGATCCTCTGAGATCTTCTTTAGAAATTTCCTCCAAGATCCTAACTACATTCATTTGTAGAAATATTTGAGATGCAATGCATACCCTGTCTAGTATCCCCCCACCCCATAACAGAAATGTGAAGTAGGGTGATCTGTCATCTTTGTGCAGGTCATTGCCAGCTCTAGCACCAGAATCTCCTCACCTGGGGAATATCTCAGTCCCAGGCCAACTGGGACTTGGATACTCTAATTCTAGGTGTGGTTGAAGCATCGGTGGGTTCCTATAACACTGGCACAGGGAAAAACATTAACAGTGGGACAGAATAGAGAGTCCAGAAGCCAGAAGTGCATATGAATAGAGAAGCTGGTCCCAGCTGGGACCAGCTTTTTAACCTTGCCAAATCTTGCTATTGCATCTTTAGCTTTTCTTCTTTCCTTTTTATACCTTCTTCCTTCTACTTTCTGTTTAGTTTCTTCTGTTTTTCTCCACTAATTTCTTAAGTGGGATGATTCACTCATTACTTTTTGCCCTTGTGTTTGTTACTGATGTCAGTATTTATGGCTTTAAATTTTCTCTACTGCTAATTTTCCTGCCTCCTGTAAATTCTAAAACACAGTATTTCAGTATTTGTCTATTAAGTGTTAAGTGAGATTTGTGTGACGTTCTAATAAACAGTTAATTTTTAAGTGTTTTGTGTGTATTTTCTAATGATGAGATACAAAATTATGTAATTGTCTATCAAATCATCGGTTAACTGTTTATGGCATCTGTTTTTCCTATTTTTTGATCTATTAAAATTGAAAATAGGTTTCTTTGTATCTTCCATTAATGAATGAATTTATAAATTCTTCCTATAATACTACTGATTTGGGGTTTTTTAAAGAACGTATGTGGCATAAAATATATAACAAGTTATCTCCTTGAAGAATGAAATATTTTACTATGTAATATTCTTGCTATCTCTTAAAATGCTTTCTGTTTTACAATAGATATCCAATATTAGTAGAAATATGCTTGTTTCTTTTTTACTTTTGGGTTGGCTATTGCTGAGAATATATTTTTTATATTTTCACCTTTAGTAATTTCAGATATTATGGTTGTATCATTTCATATGACAGATATCTATAATTTCTTTTTTTCAATGTGACAGTTTCAGTCTAGTAATTGCATAACTTATGCTATTTATGAGTTTGAAGATATTTGATATAATTCAACGTATTTTAATCTTTCGGATTTCCTTTTTTATGCATTCCTTTTAATAAATGAGTTTGTTCTTTTTCTGTATTTTCTTCTTAATTTGCCATTTACTTGATTTCTACTTTTCAAGAAAAGCTTGCAGTTGTAAAACTCACATTTAAGTCATTAAAGTCTAAAATTAAGCAAGACCTTAGCTCCATTCTAGAAAATACCAATCACCTGTCTCCCTAGTTACAGGCTATTATTATGTATCATGAATATTTGTTATAAACTCTTTCAGTTTTTGTTTGATTGAATACCTTTGTTCCCTGCTCATTCCTGAAAGATAATTTTGCTTATTATGCAAATCCAGGTGGACCATTATTTCACATTTCACTGTCTTCTGGCTATACAGATGTCAGTTGGTTTTGAGTTAAACTTTATGCACAGGTTGTCTTTGGCAAGGGCTAAAATTTAAGATCTCCTGTTTATTTTTGGCATTCATCAGTTTCATGTCAATATTGATTTTTTTTTTGCTTTATCCATTCTTTCTATGGTTTCTGTGCCTTTGGATTCATATATTTAATCATTATTTGAAGATCTTAGGGATCACCTTTCAAATACTGACACTTCTCCATTCTTCCTGTTTTCTCAAATTTTGATTTGATATATGAGATTCTCATTTTTGCACCCATGTCTCCTAAATTGACTTTTATATTATTAGTTTCTGTCTTCTGTTTTTTGTAAGATTTTCCCAGACATATCTTTTTTTATTGTCTTTTCTTCTGTGTCTAATCTCTTTAGCTAATCCATTAATTTCTATTTATTTCAACAAATACAGTTTTTATTTATTTCATTTCTATGTGGTCATTTTTCAAATCTTCCTTGTCCTTTCCAGTAATTTCCTGTTTTTTGTTTATTGTTTCCTGTTTCAAACTTTATTTTTTAAATAGCTATTTTAATACCACAAGTTTTGTGTGCAGCACCTATAATACCTCAGTGTTCATGGGCTTAGTGATCTTTGACTGTGAACTCATGTTTGTTTGATCTTAATCTGTGGGAATTTTCTGGCCTATGCTGGCATTCTTTCCCCAGGCAGGTAGGTTCGCTTTCCTTCTGATAGAAGCTAGAGTGTAAGACTTGAGCCCTTTCAAGGGTCCAAATTCTCCACCTTACTGGAAGCCAAGCTTGGGTTTCTGGCCCCAGCCCCTTGTCTTACACATCTGGCTGCCCTTCCAGCTACCTGCTCCCTTTGTCTGAGGTCAGTGCTACTATGGGTGTGTTACATAAGGGCAGACTTCCCTTAGGTCCAGTTTTCCCTTTGCTCAGGACACCCAAATATTCTTTTGCTTACACTGTTGGAGGAGCTTTATGTGGGAAAGCTTAATTTTGGATATTTCTCTTACTTCCTTGTGCCCAGAAGTTCACTAGCAAGTGCATCTTATCAGGAGGTAATTGTTTTGTTCAGGGAAGGTCTCCCAGAGTGATGTGTTACCTGCTGATGATAGGAGTGGAAGCTTTTCCTTTGAGAAGGTTTCACCAATGGAAAAACAGGAAGGAATGAGGGAGGGAGGGAGGGAGGAAGGGGGGAAGAAAGAAAGGAAGAAAGGAAGGAAGAGAGAGAAGGAAGGAGTAAAAAAAGAAAGGGAGGAAGGGAGAGAGGGAAGGAGTGAAAAAAGAACAAAGGAAGAAAGGAAGGAAGGAAGTAAAGAAGGAGAGGAGGAAGAAGTACTGAGGAACATCTTACTCAATGGTGAGACCCAGTTCGTACATGTTCTTATCCTATGAGCTAATTTTTTCTCTTTTGTTTTTCTTAAGAGAATTGGCTGTCTCTTACTCTGTAATACAGATCTGTGAGAAAATAGCTTTTATAAAAAGAGATTTTGTAGTATTACACACTTGGCAGGAATATAGTTGTCTGTTGTAATAATGAATACTAATCTAGAATAGGAGGCTGAGAAGAAAAATATAATTAAAATGGTAATGGCTTTTTTTTTATGTGAATGAAACTCATCCAGTATTGGTTTTGAAAGATATCTAAGTTCTAGGAGCAGACTGTAGCAGAATCTCCTTTAATACTCTAAGGAAAGGACGCTTTTAGAAAGTAGGCATTGCCTCCTTATGTGAAAACTGCATTCCTTTCATGAGGGTTCCATTTTCTGGAACACAGGATGTAAGACAGGAGACATAAGAAGGGATCTTGTAGCAGTGCAGATGAATCAAGTCACTGCACTTTCTTATTTGATCTTATTTTAAAAAGATGCTTCCAGGGAAGCAGGACCTTGGAACCCACAAAGTCTGGAGCAAGTCATTGACCTCGCAAGGTATTCACGTCCTCACAGTAAAATGGAGAATAAAATTGCTAGTTTTTAAGGATACTCTTAGGAATAAATAACTTGTTATAGCACATATCAGACCATCAGTCATGCCAGCCTGTTTTCCTTTCTCTCTTACTCTCTCCCTCTGTTCATTTTCTCCATCTTCTCTCCAACTATTCCTCCCTCTCTACCACTGTTGCTCCCTCCCTCCCTCCCTCCCTTCCTTCCTTCCATTTTTTCCTTCCTTCCTTTCTACATCCCTCCTTCCCCTCTCTTTCTTTTCCTTTGCTTCCTTTTCTTTTTCTTCCTCTCTCTTTTTCCTACAAAACAGTATTTGTCAACTTTGGCACTCATGACATGTGGAGCTGATCACCCCGTCTTTGTTGTAGGAGGTGTCCTATGCATTGTAGGAGGTTTAGTTTAGCAGCATGCCTGGGCTCTGCCCAGTAGATGACAGTGGCACCACTACCACAAGTTATGAAAACCAAAAATATCTCCAGACATTGTCAAATATTGCCTCTGAGGCGAAACCACCCCTGGTTGTGAACCACCACTCAAAAATACACTTCATATCAATAAAAATCCTGCTTTATATATATATGTTTTTTGCTCAGTTCAGGGTTATTAAGATTGTAAGACACTAGTGTTTTTACAAGATTTCTAGGGATGTTCTTTGATTGAGTCTTAAAATCTTACTGTTGATGAAAAATTGAAATTATGTTGTTATTTTTATATTCCTTCATATAGCAGCATAAAACTTGGTATTTTATGGGAATGAGTATGCATCTTGTTCTGATTCTATGGTCTACTTTTATGTGTCTCAAAATGAGATTCAGATCAAAGAAAATTAAAACGAGAGCAAAAGTGAATATTAAGGTAAAGGTATAGCATTCTGATTATCTGCTGCTTGTCCATCTCAGGTATGCAATACTGACACTGTGCCACTAGTAGCTTCTTGACATTCTTAAGATGAAAATAGTTTAGTTTTTATCTAAATATATTAATAGAGAATATACAATATATATTTATTCATATATTAATACTGGAACAATAGAGTAAGGTTAAACACTCAAAATTTAGCTCAACCCTGAGATTATTATGAAGTACTTACAAAAATAAAAACTAAAAAGACATTAGTAGCGTACTTCCCAGCTTCATCTCTGCAGGAGGTGTTACCTTAGCTCAGGGCTTGGAGAATAGGACATGTGTTTACGTGATTGACTCTTGTTGGGATTGTTCTCAGAGCTCTCCTGACCTTGGTCCACACACTTGGGAGCACATGATTCCTAATACTGATAACCACAGTCTCATGAATTTTTCTCATTTTGCAGAGGAGGGAATTGAGGCACTAGATGGTAATATCTTTTTCATTTCACATAGTTGCTGGTGGCTAAGGGAAGCTGGTGCTCAGCTTGTCCCAGGCCATATCTAAGACATTTGTCTGGCCCCTTGCTTTCCTTCCTTTCATGCATACAGCAAGCATATCCAACTTTTCTATGCTGGTCTATTTCTAGAAGGTGTTATTTGACATGGCATCACCTCCTTTGTAGCCCTCTGACTATGAGAATGATAGAATGACCTCTCTTTTAAACCTATCTCCTTATCCGCCCCAACACATACCCCTTTGGGGTGGGGTCATAAGGGGGTATCCCTTCTCCACACTAACTTTACCGACTTCTCTCTTCATTGTCTCTCTGCAGCAGATAATGTAAGCAAGAAAAAGATTAAGTTAATTACATGCACCTCAAGTTTCAGTAGGAATATCCCACAATTCCTCTGTCTCTTAATTTAACTGTTATTTATTGAACACCTGCTGTGTTCTTGGGAAAATTCCAGGTGCTGGATGGAATTAGTTTATGATGATAGCTAAGACTTGCAGAGACATTAATGTGCTGTTCTTCTTCTTCTTCAGAAAGTATAGCCATGTACAAACTACTAAAGGGCGATATCAAATGTTGGGGAGATAAATATCAAAATACAGAGCTTCCATACCTGTAGTTTTGGTTAGTTTAATAGGCGTTAACATTTACTCATTTTCAGCTACCTACATTTATTGAGCAGTGCCTATACCACTCATTGTAATTTAATTGCATAATAAATTACACTGTATTTGCTGTTTATAGAAATTTAGAAATTTAGTTTAACGATATGTTTATAATTTTCTTACTACTATGGATAATACATTTAATGACTATAATTAAATTCTTGCAAAATTTTTGAATTGTTTTTAGTAATTTGCCAATGATTTTCCCAGGTATTAATTTAATATATTGAAATTTTGTCTTTATAGCATAGAGGTTTTTTATTTCATTCATTTATTTAACAGGCATTTATCATTCATCTGCTTTATGCAAGGAAAAAAATGGTCAAGACAAGGATGCCAAGTCTTTAACCTCAGGGAACTTACAGTTTATGTACAGGGACACATACTTATCAAATAAACAGAGAAAGGAATGTATATTCATATGAACTCGGCATCATATATTCTTCTTTATGTTATCATTAATAACATCCAAATGTCAACAACACATCTATTGTTACTTTGGTTAAAAAGCTACACAGACAGTAGTAGATATGGTACTTGGATGAAGAAAGCTGAAGTTTATTATTTTCTCTTTCTAGTTTTAATCCCTAAGGGTCATTGATAAAAGACTTACACAAACCCCCCTTTAGTAACCTAATAATGTATAATAATCCTGTTCTTAAAATGGTGATAGAGATTTGCTTGGTTTCTACTACATAACACCATAATACCATATTAAGACTTGAATCTCTTTATATCATGGAACAACTCAGGTAGTGTTACAAACTGCTGTTACTGAATAAATGCGGAGAAGAACAAGCTCTCCAGAGCAGTGCCATGCCTGTGTCTGATGTTTCCCAGGATAGAAAACTGCGCAGATGTTGATGGTTTGTTTCAGGTGCTTTGACAGCCTGATCATGGGCTCTAGCCGTGGACCATGAAAAATGGCTTCTGCAGGGGCTTAAGAAAGACAATGAAGAGCTTCGCATTTTCTCTTGGCATTTCCTGCTATTGTTTAAAAGGTCACATATGCAATTTAAAATGTTCCATGCATGGAGCATGACAAATGCCACGTAGAAAATGAAACTGCTTTCGTTGACATTTTTGGCCAATTTCCAAAGGGTACCATTTTCCGCCTTTTCCCTTTTGTGGATTTGCAAAATTTGGCTTGTGCAAAATGCGTGCCCCACGGTGCACTCTAGGTTGGGAAGTGCCACATGTTAGGTAGAAAATCGTGTGTAGATGAGAATGGCACATTCAGAATAAAAGTGAGAAATTAAATGACATCAAAAAAATAGAGAAAAATAGAGAAAAACTTGTAAATGAGTCCATCAGAACTATCAGAAGCTCAAAAAGAAAGAAAGGCTTAGAACTCATCAATAACAATGTCCAGTCTCATTCATATGTAAAGAAAGTGAAATCAACTTTATTTTAGTTAATTTTACTTTATTTTATTTTATTATCCTTTTACCTAGCTGAATGGCAAAACTCAGTTCAGTTATCTTTGGGCATGGAAAAATGAGCACTCTCACAGTTTGCTAGTTGGAGGAAGAATTGAAGTAGAGTTTTAGAAGACATTGGGTATTATACAACAAAATTTAGAAAGAGACCCACTTTACTCCTCTGGAAGCATTTTTGCTTCCAGGAATCTATCTTACAGATATATACACAAAGATATATGTACATAGGTGATCATTGCAACTGAAATTTTTCTCATCAGGAAGATGAGTGAATTATTTTAAGCACTTAGAATATTAAAACTATCTTTCCCTTGAAATTGAAGAGGCAGAGCAAAATGTGAGGACACAGAGTAATATTCACATAAACTCCTTAAACCTATGTATGCACGTATAGATACTTGTATATATACATAGATATGAATGCACAATAGTATCCATACACATATGTGTACATATGTGTGCATGTGGGTGAATGCTTATGTGTAGATTTGTATACAAATGTGTGTATGTTGCTGTATTAAAAAAAGTCAAAAAATAAACAAATTATTAACAATGTTTGCCTCTTAGAAGGTGACTATGGTACGGTGCCCTTAGAGAGAGGCTTTGATTGGCAGAGAAAATGAAAAACCATAACTGCACCTATATTTAAGATTTTAAAAAATTCTTTGTAGTGAGTTTGAGTAACTTTTAAAAGTACATTGACATTTCATTTATGCAGATCTTCTAGGTGTGTATAAAAAGCCATGAGAAAAAGATGATTTCATGTGATAGAGAAAACTAGCACAGGTTAGAATTTGGACTCAGCTGATGAGACAGTATCTGCCCAAACCAATTTAATCAAAGCTTTGTTGCATGAGCCGGGTGTGGTGAGTCACACCTGTGACTGCAGCGCTTTGGGAGACCGAGGAGTGAGGATCACTTGAGGCCAGGAGTTCAAGACCAGGCTGGGCAACATAATGAGATCCCTTCTCTACAAAAAGTTTAAAAAATCTAGCCAGGCGTGGTGACTCAGGCCTGTGGTCTCAGCTACTCAGAAGACTGAGGTGGGAGGGTTGCATGAGCCCATGAGTTTGAGGCTGCAGTGAGCTATGATCACACCACTACACTCCAGCCTGGGGGACAGAACAAGACCCCGTCCTTAAAAAAATTTGTTTTAAACACTTCATTGTGTGGAAGAAAGCTGTATATTTAAACAAATATAACCAAACCCGTAATACTGGGGAGAAAGATTGATGGATTGTTGAAAGGATTATACCCGTTAGGCCAATTTTGAGATGTAGGCAAGGAATCTCAGAAGTTCCAAAAAGTTCTGCTGTGGTTCAGTGTTACAGGGAAATCTACTCAAGGGAATAATATATGGCTTGCAATCATTTTGCTTTTTTGTTACATTTCCTATTATTCATTGCTTCATTGGGCTTGAGAGAAGCCCCACAGAGGAATAAGAAATACCCTACATCATTCACATCTTCTTGGCTTTTGAAAATTAAATTTTATATACTTAAAAGCAGCCATGACACATGAAAACATTTTCTTTCTTCCTCAAACCATCTTTACCTAGCCTCACCCAAACCAAACTTTAATTTTTACATTAATTTTTCTTTTCCAAAGCTATGCAGCTGACACTCATCTGCTCACTTGGCATAATTCATTTGGTATCCAGTAAGTTTAAGAAATTCTGTCTGGGCTTCATGCAATCATAACCTACATCCAAATAGCAACACTTATAATAACAGTAATAATAGTATTTTTTAGTGTTCACATGGATTTTCTCCCTTAATTTTCATGACATCTCAACAAAATAGACAAAATACATGGGCTTCTCCTCAGCCCTGAGCTTTGCCTATCGTTAACCCCTTGAAGAAAAATGGCGCTGAGCTATCAGTCAGTCATTCCCTGGCAGAAAGGGAACAGAATCAGTATAGATGGCTTTCTGAAGACATTGACTTGATTTCTGTCACCAACAATGGCATATTCAGGCTGTGCTCCATGCCAGGTGCCGTGTGGGCATGGAGTCCACCACACCAGGGGAATTCTCAGAAGCAGTATTGAAAACACATAGGAAAGCATTACTTAAGCCTGTATAAACATAAGCTCTGTCCAGACATGGAATACAGTGGGAGTTCTTCCTAGGATAATCCCAAAAACTAATACATCAGAAAGCTTACCTATAACATGAGAATTCAAGGCAAAGGCATTTTTGGTATGTAAGTAAAATATTAGGTTGAATCCATCTCTTAATGCGGATGTTGAAGAATTAATGTTATATCCATGAAGCCAGTGTTGACTGGAAGGACTCAAAAAAATCTGAAGAATATAAATTCCTTGACCTTCTTTATTGAAGACTTCAGCTCCATTACACGACCACCTCACAGTCCTCATTCGGTTGCCTTTTGCCTGTTTCTGACTTACTGAAGGACAATGGTGTGGAGCTACGATTTATCACCCAGAAAATGATTACTAAAGTCCGTATTCTACTCTGAATACTGAAAACTCTGAAGTAATGACCCTAACCTAAACCTCCTCTTCTTCTGGCTATCACTTCTTCCTTCCCACTTTGATCACTCTTCCATGAATCCTGGCAAACCTCCTAGTACTGAGTATCCTTCCAGCCACCAAACGTCTGACATAGATCGCTGGATCTGACTTTAATTCTCTCACTAAGACCCTCAATTTCCTCCTCTGCTTGTGGTGGGCTCACCCTGTTGTTTCTCAGCTAAGGGTGCATCCAGATATCAATTTCTTGTGTCCCATAGCACTGCTAGCATTAAGTGAATTACTGCATGGTTTGGTCTCATTAGTGTGTGGTTTCCAGAAACACTTGAGATCTTACTGTTGGCTTGTAATCTGTCTTAGTCCATTTTGTGCTGCTATAACAGAATACCTGAAACTGGGTTGTAAAACATATAAATTTATTTCTCCTAGTTCCAGAGGCTGGCAAGTCCAAGATCAAGGCACCATGATCTGGCAAGACCTTCTTGAACATCATCAAATGGCAGAAGGGCAAAGAGCTTAAGAGAGTGAACCCACTCCTGCAAGCCCTTTTTATAATTACACTCATCTGTTCATGAGGGCAGAGCCTTTGTTACCTAAACACCTGCCATTGTCCCCTCTCCTGCAACACTGTCTTACTAGGGTTTAATAATATTCATGTCAACGCATGAATTCGGGGAACACATTCACACCATAGGACAACCCATTTACACTCTCTCCTCATCGGGGTCAAAGGGCATCAATTTAAGGTTTTTTGACCTTTTTTGTTTTCATTATATCTCATTTTTATACTAACAGATTCATTTGTTCGTATAACTCTCCTGTCTTCCAGAATCTGGGACAGTTTTCCACCTCCCAAGTGGGATCTAGGAGTTAACCCCCACCATCAACCCAAGTACTCCTCCTGTGTCCAATGGCCAGTCAGCCTCAATCCTGTCTTCTCTTGAGTTATGACATATTTTTCTCCTTCCATTAATAGTGACCATTACTGTAATAGGAATTTATAGTTCTTTGTCCTCCAGTTCTCCAAAACTGGTTCTCTATCCTTTCAATTTTATGCTAACAAATCTCATTAAAGTATGACCAGTGATTTCTACATTGCCAAAACCCAGTGGTGTCTTTTTAGTGATGATCCTATATCAATTTGATGGGCACTTTATCACTTGCAGAATTCTTATTCCTTTTCATTTTATCACTATGTTCTGGTTTTATTCTACAATTGTGAGAAGCTCTTCTGTATTTTCTTCTCTTATTATTCTTAAATGTTGACTTTTCCTAGGATTTGTTCTTGACTTCATTCTGTATATTGTATGTCTAGGTAATTCATTGCATCTTCTTATCTTCAACTATCTGCCTCTATGTGGATGATTCTCAAGTCTTTATTTCCAGCTCAGGCCACTAGCTTCAGTTACAGTGTTTGTAATTTTAGCCCCTATTAGAAATCTCTAGTTGAGTGTCACATAGACACTCCAAACACAACACATTCAAATATTAAGAGATGCTCTTCCTCTAAAACCTATTCCTCTCTGCACCCTCCTGTTAGTTAAAGGTGCCCCATATACCAGTGTGTCCAAGATACAAACTCTGTTGGATTTTACTTCTCTTTTCTCAGCACTTATGTAAATGGATGTCTACTTCTCATTTCTGCCCTGCAGAACATTCCTAGCTATGTGCTGTCTTCCTGTGGCCCACTGTGACAGCTTCCTTATCTCAGTTTAGATTGTTATGCAGTCCATTACTCTTCTGCCTCCTACCTTCAAGCTACTATTGGAGTCATCTTCCTGATTCTCACATCTGATGGCTTTCAGTGGCTAAGTGATGCATTCCAATCTTTCTTAGTTCATTTTATGCTGCTACAACAAAACACCTGAAACTGGGTTATAAAAAATAGAAATGTATTTCTCATAGTTCTAGAGGCTGGGAAGTCCAGGATCAAGGCACCATCATCTGGCAAGACCATTTTGCACATCATCAAATGGCACAGGGGCAAAGAGCTCAAGAGAGTGAACCCACTCCTGCAAGCCGTTAAAAACGCATCATGGGCCGGGCGCGGTGGCTCACGCCTGTAATCCCAGCACTTTGGGAGGCTGAGGCAGGCGGATCATGAGGTCAGGAGATCAAGACCATCCTGGCTAACACGGTGAAACCCCGTCTCTACTAAAAATACAAAAAATTAGCCGGGCGAGGTGGCGGGCACCTGTAGTCCCAGCTACTCGGGAGGCTGAGGCAGGAGAATGGCGTGAACCCCAGGGGGCGGAGCCTGCAGTGAGCCGAGATTGCGCCACCGCACTCCAGCCTGGGCGACAGCGAGACTCCGTCTCAAAAAAAAAAAAAAAAAAAAAGAAAAAAACGCATCATGGCAAAATCTCTTTTTTTACCACCTGGGAAAACCTAAGACCCTTGGGACAGCACAGAAGACTCCTTAATCTGCCCATGTGTCCCTTTCCAGTGTTAGCTTCTTTTACTTTTTCTTGTACACCTCGTGCCCTTGCCCCTTGGAACAAACAGCTCACAGTTCCCTCAGCACACCCACCCTTCTACCTGCCCGGGAGCTGCCTTCCGATAAGTTGTATCTCGATGACTTCCTCCCCACTCTCCATCTGGGAAGATCCCAGTCATTCATTTGTTAAGGCCCAGTGAAAAAGATTTTATTTATTTTCCTTCATATAATATTTTTATGTATACATATATATGCATATGTATGCTATCTATCTATTAGATACATCTTGTTTTGGCTTATTTTTATTTTTTATGTTTTGAGACAGAGTCTCAGTCTGTCACCCAGGCTGGATTGCAGTGGCATGATCACAGCTCACTGCAACCTCGACCTCCTGGGCTCAAGCAATCCTCCCACCTCAGCCTCCCGAGTATCTGGGACTACAGGTGCATACCACCATGCCCAGCTAATTTTTGTATTTTTTTTTTTGTGGAGACACAGTCCCACTATATTGCCCAGGCTGTTTTTGAATTCCTGGGCTCAAGCAATCCACCTGCGTCAGCCTTCTATAGTGCTGGGATTGCATGCCTGTGCCCCTGTGTCTGACGTTATCCTTGTTATTTTAATGCCTACCTCATTTGTCTTTTTCAAATAATAATCAACAAATGATTTCTGGATTGATAAATGCATGAATGAAATGATAGTTTGCCAAAATACAGAATATTAAAACCATAGGGTAACCTTGAGACAATTTAGGTAAAAAATAGGGGATTATTTTATATTAGAAGATTATTCAATGTATTATTAAAATGTTTGTTTATTGCATGTGTTTTAAGTGTTGAGAATTTAACAGAGAACGAGACATGAATGGTCTAAGTGTTTATGCATCATAATAAAGTTGAAGAAATGTAGGGTTCCCATGGTGTTTCTTTTCAAACTTTGATAATAACACTTCTTTATTGATCGCAACTGTACATTGGCAGCACCGCCTCCAGACTGGAAAATAAGATCGATTTCTCCTTTGTGTTTCTTTTATAACCTTGCAATTTTATTCCTCTTGGGCTTACTGTTATGAGTTTGGTTTCTAGTTTCTAGAGCATGAGTTCTAAGAAGTGGAAATCAAGATGGAAGGAAGTTACTATAGTGAGAGGGTGTCATGCCCTGCAGGCTAGGTATCTTAGAGTCTGACTGCAACTCCCTTGACACAGGCAGTTCTTTTTCTTGCCTGCAGCCCTTTCCAAACAAATATCACCAGCCTCATATTCCCCTCCCCTTTATAGATGGAGCCCCTTTGTCAAGCAGGCCAGTTTACTGGGAAAAGGCCCTTCTCAGACATGCTTTCTCATCCTGATGCTTTGCCTTTACCAGGAGTGAGGCCAGAACCTTCAGCATGCATTTATATCAAAAAAGAGAGATGTGCTGTTTTCATTTAAATTCCGCATTTCCACTGGGCATAGTGGCTCATGCCTGTAATCCCAGCACTTTGGGAGGCTAGGGCAGGAAAATCGCTTGAGACCAGGAGATCATGACCAGCCCAGGCAACATAATGAGACCCCGTCTCTACAATTTTTTTTGAGAAAGGGTCTCAGTCTGTCACCCAGGCTGGATTGCAGTGGCATGTCCACAGCTCCCTGCAGCCTCAACCTCCTAGGCTCAAGCAATCCTCCCACCTCAGCCTCTGGAGTAGCTTGGACCACAGGTGTGCACCACCATGCCTGGATAATTTTTGTTTTTTGGTAGAGACAGGGTTTTGCCATGTTGGTCAGGTTGGTCTTGAACTCCTGACCTCAGGTGATCTGCCTGCCTTGGCCTCCCAAAGTGCTGGGATTACAGGTGCGAATCACTGCGCTCAGCCTCTATAATTTTTTTTTTTTAATTAGTGTGCTAGTAGTCTCAGCTACTTAGAAGGCTGAAGCAGAAGGATTGCCTGAGCCCAGGAGTTTGAGGATACAATGAGCCATGATCACATTCCACCCTGGGTGACATAGTGAGACGCTGTCTCTATTAAAAAAATAAATAAACAAATTATAAATTTTCACATAGTCGTAAACCTCTGAAGATGTGGATACTTCATTTGTCACATTTAGGTCTTTAATACACTAATACCTTCTCTTGGGAAACAGTGTTTCTCAGTCTCTCCCGTATTGATAATGTTTCCACTTTGCCCTTGAAGATTTTGTGGGTTATGGGGAAACAGTTTATGGGGTGTCTTTCAGCAGAACCACAACCCTTTTTAGGAAGAAGCTAATTATGGTGTGAAAGGGACAGGTGCTCTTATTAGGTAGTGATAGTAAGAGTTAAAACCCAGTTCTCTTGAGCTGTTACTTGGATTCTTCAACTGAGGGTGATTTTGCATCTTTGGCACTAGATGTCATTCAACTGACAGTCATGGACTCCCAGGGGACCCCCAAACTCTATGTCACCTTTATGAGTAGGCGAGAATGGATTTTTCTTGGAGAGGAGTGTCTCCTCAAAGAAGTCTGTGACCTAGAAGAAAAGATGAAAAATCTCTGCTTTGGATTCGGAATGTCAGGACTGTTCACTTGGAACTTAAGGAGAGTTTCTTCCTAGTATATACGAGACTGAACCTTATGGGGTTGCCATTTTCTTAGACCCAAAGCTTTCAAATACAGTCATTTTCATATGACTTCTACTTAGACAATAAGATCATCATGTATTCCTTTTTTCCTCTTTCAGCATCTGGCATTTTTCTCCTCTTGGGCTTGTTGTTCTGGTTTTTTTTTTTTTTCTGGTTTCTAGACCATAAGCATTCATGCATTCACATTATGTTGCCTCCTAAGTTGTAAGCTCTCCAAAGAGAGGGAATATAGCTGCTTTATGTCTTCACCCAACTTTGAGTAGAGATGATGGCAGGAAACAGAGAGCATTTTCACAGAGAAGATGGAGTCCATTTGAGTCAGGGGATCTTGTTTGAAATCTTACCTGTGTGATCTGGGGTGAATTAATACAGCTGTCTGGAAAATTTAGAACAGAGACCTCAGAGGATTGCAGTAAGGAGTCCTAGAAGTTAGGATCTCCTCAGTAAATATAAATACTTATTCTCTTGGGTAATGAAGCTGACCCACAGGATGATGCCAATTATTTCCTTGGTATTATAAGCACATAAACAATAGTTCACATTTATTGAGTGCTTACTATGTGTAAGATACAATTATGTGCTTTGGGATATGGGTTCACACATGAAACAAGTGTTTATTTAGTGCCTACTCTGTGCCCAACACTGGAGATGCAGCTGTCATGAGCACTAACACCATCCCAATATCATGGTGCTCATGTACCCATGTGGGAAAAAGTAAAGACAGGCTCAAGCATATAAAATAGGGAAGGTGGTCTTAGGATAATTCAAGCTGGATTGGGATCAGTAGTGATTGAAGGGCTAGATTAAATGAGGAGTTTAGGACATGCATCTCTGCAAGATGGCATTTGAGCAAGAAACATAGGCAAGACTTATCTACTTTAATTTTCACAGTAGGGTCATGAGATTACACTGTTTATTAACTCTGTTACAGAGATGTGGAAACTGAGATTAGGATGATTGAATAACAGCCAGATTAGTAATAGGGCTGGTAGTCTTTAATGCAAGTCTCATGGGCTATGCTGCACACAGTCTTAACAACTTGCCACCTTCCGTGGTATAAGAGAGGAACCAACCCAATTCCCGTTGCCTGCCTTCCCTGCTATATTAGTCTATTCTTACACTGCTATAAAAAATACCTGAGACTGGGTAATTTATAAAGGAAGAGGTTTAATTGACTCACAGTTCCGCATAGCTGGGAAGGCCTCAGGAAATTTACAATCATGGCAAAAGGTGAAAGGGAAGGAAAGCACCTTCTTCACAGGGCAGCAGGAAGGAGAGAAGTGCTGAGCAAGGAGGAAGAACCCCATATAAAACCATCAGATCTCATGAGAACTCACTCGCTATCATGAGAACATCGTGGGGGAACTGTCCTCATGATCTAATCACCCCCCATGAGGTCCCTCCCCCAACACGTGGGGATTACAATTTGGATTACAATTCAAGATGAGATTTGGGTGGAGACACAGAGCCAGACCATATCACTTGCCATCTAATTACCTTGATCAACTACCCTGCAACCATTCCTTAGTGAGTAATAGGGCCACACTCAGGAATGGTTTTAATAGAATTTAAAAGTTATCAGTATTGTAGTTTAATTGTAATTTTAAAAATGGTGAACCTCACATCAGTGGCTAGGATCAGCACATGATATGCTGCATCTTGGGGTCAATAATTGCCGCAAGCACATTATTAGAGTTGCTGTTAATAGTCATGGAAACCACCCTGTACCTTCTTCCCCCAGTGCAACCAACCTGGCAGTGATTGACCTACTCGGTAGCGAGTTGCTAGACATCAGGAGAAGTCAGAAGTAAGTGGAAGAAGGCCAGGTGTCTAGAAGACCCCCCCACTACCCATAGCAGTAGCAACACATATGCATAGGAATAGGTTAAATGAGTCTTCACTCATTGATCCATTCATTCATCTTTCATCCATGAATTAACTATTCATGACCCATTGTTGTTGACTCTGAAGATACGATAGCAAACAGGATGCACAAATTGTCCTGCTGTTACTTTAGTTATGGGGACAGAAGATAAAGCAGTGATCAAATGCATGAAGGACAGAATTGCTGATGGTGATCATAGCTTTGAGGGAAATGAAGCAACGATAACATCTAATGTGGGTTATGAGGATCTTTGAGATGGAGTGGCCAGGGCATGTCTTTATGAGGGTGAGGAATTTAAGCATCCCAGACACAAGTTCTGACTCAAACATCAGCCTTTTAATTATGTGAAAGGGTCTCGCAAAATTTAATAAACTTAGTGGTAGGAGTTCAGGTAACACTACAAGAAACCAAGCTTTCTTTGTGAATGGTGAGGTTAGAAGGGGTTTGTTGCTGAAAATCCCATTTGCAGGTTCTAAGGCTGGGGATGAAGTAGAAGGAACAATCTCTTGTCATTTGCCAATCAAAGAACAATCCCTGTATCTGGCAAAAGAGACATACCTTTCTATGAATCCTGGTTTTGGTCATAAGCCAAACTTCTATATTAGTTTTCCCTTTTTGGTTGAGTTAGTGAACAATTGGATGATTAGCTAAATGTTGCTGAAATAGGAGGAAGGCAGATTAAAAATACAGAAAGTAACTCTTATTTAATGATTTGAAAAAATGAGGTTAATCCGACAAAATTTTAAGGAAAAGTGAGATAATTTTGGTGTATAAAACTATGAAATTTTAGGCTGGGCATGGTGGCTGACACCTGTAGTCATAGCACTTTGGGAAGCTGAGGCAGGAGGATTGCTTGACCCCAGGAGTTCGAGACCAGCCTGGGCAACATAGTGAAACCCCGTCTCTACAAAAATTACAGAAATTAGCTAGGCATCCTGGTGTGTGCCTATGGTCCCAGCTATGAGGGAGGCTGAGGCAAGGAGAATTGCTTGAACCTGAGAGTTCAAGGCCTCGGTGCACTCTGTCCTGGCTTGTAGAGTGAGACCCTGTCACACACACACACACACCACACACACACACAGACACACACACACACACACACACACAAAATAAAATTTTGGAATGTAATAACATTGATGCTGAAGTGAATTGTGGAAAAATATCATATAAAATATATTTTAATCACATAGTATAAATTTCTCTCTGTGCATTAGTTACCAAAATTTGAACATAAACATTTTCAAATACACACTTGTGCAAATGTCAGGGATAGCAGGTGGTATATCACTTTTTATATTTAAAATGCATGTAGGAATGAAAGGAAAAAGGTAAAAATATGTTAAGTGTAGAATTCTAATGAAAGAACATATTGGAACTATGAAAACATTATGGAGGACTTTGTTCATTTATGGTCTGAGCACAGATGATGCTAAACATGGTCCTTCAACTTTAGCTGGCAGCCATTTGAAATGAACACACTAAACACCATGAGAAGCAACTGCATGAAAAGCAAAGAGAGTTATCCAAGTGAACTTCATATCTCATCATTTGCCTGTGTTTATGTAATAGTAAAGACCCAAGGAATTGGTCTAATTAATTGGTATTTTATTTTAGTGATGAAATAATGAGTGCGGTTGAGCATGCCAGATGTATTCATCTGATACATTCTTCCAGTCACATGGTAGGCTGCATTAGGTGATAATGCTTCACCCTGCATTCATTTATAAGTTAGTGAAGGGAAGTCCACAACTCTGGTCTCAGAGCATTTATCCCATTGTTGATCAGCTAAGCTGTTGCTCTTACTTAGCTGCTAAGGAATGAAGCTAATTGGACCATTCCAGCATGTAAAATATGTAAAATATGTCCTTTCATGGAACTCTGAAACAAACAATGAGAACAACCAGAAAAATTGCCAGAGTCATACAAAAGCTGTCTATTTCTAAATGATCATTCCTCAAGCTCTTGTCATCTACTGGGAGCCCCTAGATGGATGTATAGTTGTTGCTGTTGTGGCTGATTTTGATAGGACTAACATAGGACCAGTGTATGGAGCTGTTTATTAAGATGCTTTTGTTGCTGAGTATTTACATTTTGGGTGTTCTCGGATAACATACGTTAATTCCTACTGCAGTATTTAATAAAGTGTAACTAGTGCCTGTCTCACCTGTCTGAAGACATTCAAATATGGAGCGTTTGTTTCTTTCTCTAGTGCAGATACTAAATATCATATTGTAATTAGAGCTATACAGAGATTTAGCATATAGGACTGGCAAGTCTTGGAGGCCAATTTTTATGATGTGGGAAGAGGGGGGCGTGATTTAGAGTGGACAAATAAAGTGTGGGAAAATTTTGTGTTTCTGGCTTGAGTGACCAGCTCTTACCTCTCCTCCCCATATTCTCTTCCTTGCCTCAGTGCAAATTCACACTGTCTTCATTTTGTATGATCACCCTCTGTCTTAGTCCATTTAGTTTTGCAATTAAGGAATCTCTGAGACTGGGTGACATATAGAGGAAAGAGATTTATTTGGCTATGATTCTGCAGGCTGTACATGAATCACGGCATCAGGATCTGCTTCTGGTGAGGGTGTCAGGAAGCTTCCACTCATGGTGGAAGGTGAAGAAGAGCTGGTGTATGCAAAGATCACGTGGCAAGAGAAGAAGCAAGAGAATGGGGGGAAGGAGGTGCTAGGCTCTTTTAAACAGTCAGCTCTTGGGGGAATGAACAGAGCAAGAATTCAGTCATTACTGCAAGGCTGGCACCAAGCTGCTCATGAGGGATCCACCTCCATGACTCAAACACCTCCCACTAGGCTTCATCTCCAACATTGGGAATCAAATGTCAGCTTGATACTTGGAGAGGACAAACATCCAAACTATAGCACTCTGTCTCCTTAGGTGCACCTTTCTTCTTCAGTGACTAATCTAGAGTTCTCTTTGGAAAATGCAAATGTAGTTATGTTTCTTTTTTGCTTTTATGCCTTACTGGTTCCCTGTTCTTTATAGCATCAGGTTGCATCTTCATCAACTGGGGAACCAGTTGATGAAGAGAAGATCAGCATCCTGAAGTATCTTGTAACTTCTTGAAGTATCTTGAAGTATCTTCAAGATTCAGAATGCATGTTACCTTCTCTGCAAAGTGCTCTTTGCACCTTGTCCAGTGTAGCTGTGTTAACTCCAGTGCACCTTCCTGATGATCTTCCTAAGGCTCTTACCTTCTTGTCATTAGTCGTTTCTGTGACCATCTTGCCTATAGGAATGTGGGCTACTGTGGGCAAGTACAATGCCTGGCATGCAGCAGGCTTTCCAGAAATGCTTGTTTGGCTTCTAGAGTTCTCTTTGCTGTTACCACATCCATCCCTTTATCATCCTTTTTTCCCTAGTCATCTTTCCTCTGTACCTTTGCCGTTGGTTCTTTCTCCATGAATCAATATAAATAATACAAGCTTTGTGCATAGCAGACCTTCACTCTTGTCTCATGATTTCATTTCTTTCTTCGGCATACTGAAAGGCAAGTACCTTTCTCTCTCTGACTCTCAATTTACTCATCTGTATAATTTTGATGGTTCTTTCAATTGTCTGCTATTGCTGATGATGGCACGAACTCAGATATGCAAAGTATCAGACTTTCACTCTTGTCTCATGATTTCATTGCTTTCTTCTGCATACTTAAAGGCCATTACCTTCCTCTCTATGACTCTCAAGTTCCTCATCTGTATAATTTTGATAGTTGTTTCTACTGCCTGCCATTGCTACGACAATGGCACAAACTCAGATATGCAAAGTACCTCTGGGTTAAATGTGAACAAAACCTTCAACCTGCTGCAAGATAATCTGACCTCTGCTTGACTGTCTAGCTCTGTTTTCCTGGCAGTTGGATGAAGAACATGGCAACAATATTCTTGGCCACATTGCTTACAATACAAACGATCCCCTATTTGTAAATAGCATCATGACCAGGAGAAACCATAAAGACCTGAAAGAACCTAGTGGTAATACCACCCCACCTCAGGCTTCCCGGAGGGCAAGTTTTGGAGTCACTTTGCAGCTGCTCTGTTCACTCTAGGAACCATGGAAACTCTGCTCATGGAGTATTTACAGGGAATATTGGCTGCTGTGAAGGCTGGGACTTCAATGCCAAGGAATACCCAATTCCCGTGGATATGGACCTTGTAGGGATCTTTGCATCTCAGCTGTCCTTTGTGGAGCAGATGGTTCCCATATGCCTGCTGCAGCCTTCCTGATGAGCTGAGCTTCTTGTCTGTATTGTTTTGAGTCGGTTGGCACCATGGTAACTTTGGGGGGGTCTTGTGATTCTGCATGTTTAATGGAACCTGAGAAGACCCTTACTGGGCATTAAAGAACAAAGACAAATGTCCCTGTGACAGAATACTGGCTCAACAATTGGTTTTCTCTCTGATGCCTCTTCCCTGCTTGGAAAGCCCTTTTCTTTTATCCTTCATAATCACTTCTTACATCTGGCACAGCCTTCAGCTTTGCATTATTCCTTCATTATCTTTTCTCATCCCACATTAAAAAAAATTCTTTAAATTGTGGCCAAATGAACATGACATAAAATGTACCATTTTCACATGTGCAGTTCAAGAGTATTAAGTACATTCACATTGTTGTGCAAACATGCTTTTTTTCACTCTGTGCCCTCATTTTGCTCTTTCCTGGTTTCCAATGCAGTATCTTATATATGATCTAATAAATGTGTCCTGGGCATCTCAGTCTTGTATATTTTGGTCCTCTGTTATATCAGGTACACCTTAAGGATAGACATTGTGCCCTACTAATCTTCCTCCTTCATCACATGAAATATTGTGCTTGCATAGTACATTTTCTTCACTCCCCTCCCTGTTATTTTTTATGTATATCATGACACTTATTTGCCAAGGATGGCTTTGGCCCTCTATGCAAAATGTCACCAATGGGAACAATGCTAAAGTCTGCATAAATCTTAAGTTTAATTCTAATTTTAAATATTTGAATATAGTGCTAGTGTTGTCATTCTATAGGATTCATTAATTCATCCCATCAACAAACACTTATTGAGTTCCAAATTTGTTCAAAACATGGCCGTATGTGCTGCTGTAGAAAAAATGTAAAAAGTCAGTTTCTAGTGTAAGGGAAATAAAATATGGATATCATTAAGTCCTGGAGAAGGCAGGGGGTGACTGATTTCAGGCTTGTACCATAGGGATTCCCAGGAGGAATAAGTAGGTTGCAGCATTTAAGAAGGGATCATGAAAGACATGCCACTTTAACTAGTTCCAAATGGAATTTTGGAAGCAGAGCCATTGGATGTTATAGCTGAAGTAATATTTTAAGCAAGGTGTCAGAACAGGATTGAGGCATAATTTCAGAAGAACATGAAGTCCTTGTTTACTAATGCAGAATATGTTTTATGATAGGCTGGAAAGTGAATCTGTGACTAGATTTGGGAGTGATTCAGTGTACAATGAATATGGCAGTAAAGAGCTTGGACTTAATTCGGGCTGCTGGTCTGGTCAGCCCTTGTGTTTGGAGAGATGAGTAACATTTGCAAAGGTGGAGAGAAGGAATTGGAGATTCTAGTTAGGTGCTTTGGGCATATGTTCAGTGAGGGATGAGGCATTAATGTTCATCAAGGCAGCATTCACAAGGGCTATGGCGGCACTGAATGGGAGAGCAGACAGACACAGGTGTCATCCCAGAGGTGGACTCCGTATGGCACAGCGGCAAGGGAGTGTGAAGGGTTATGACAGATGCTGAGTAGGTGCTAGCAACATATTTTTTAAAATAGTGGCAAAATGTATGTAAGATCTATAATTTTTGCATGTACAGTTTAGGGATATTAACAATATTCACACTGTTGTGCAAACATGCTTTTTTCACTCTGTCCTCATTTTACTCTTTCCTCATTTACAGTGCAGTATCTTATATATGATCTAATAACTGTCCCCTAAGCATCTCAGTCTTGTATATTTTGGCCCACTGTTCTATCACGTACACTTTGAGGGGGCATTTTCAGATAATTCCAGGTAAAACGTAAACCTCACGATGGCAGCTAAGAAAACAGGGGCGTTCTCTGCATTGGTTAGTTGCAGGGCTATTAGTCAAAATTCCAAATCTCATATGCAGAAGGCCAGGATCTGCAGTCTTAAGTAGTTCAGTTTGTTTCACGGAGGTAAATAAAAGAAAAAAGGCATGCTGAAGATACATATCCCTGGCCTCTAGATAATCAGACAGTAAGATCTCTCCCACACACCAGAGAAATCTATTTCCAGCTTTCTGTTGCAGTCCATGAAAATGACAGAAAATACATGCCCTGCTTGGACCACAGCCTAGCTCATGGGAAAAAAAAGGAAAATAAAAAAGAACCCGAGCTTGCTGTGGATGGTTCCTATGGAGTGTTTTTGGCACTGTCAGAGTGCACACTCTGACAGGCTGGGCATGGTGGCTGACACCTGTAGTCGTAGCACTCCATGGCACTGAATTTACGGTGGAAGGATCACATTGGCAAGTCAAATCCTTGGGCTACAGGAAAGACTCCCATGTGCTGCTTTTATGCTCCCCAGCAGCCAGGCTGTCGTTCACAAAGCACTCTCCAAGCATCTTCATTTAATGTTGTTGGGCACAAGGCCCTGGTGACCCCGTTAAAATTTAAATCTTGCTCATACAAAGTGAGGGCAGGTTTTCAGTTGACATTTGGAGGTTTCTCCAGCCATGTTAGAAACAAAATGCATTTAAGTGATGAGCCCTTGATACATAAGAAGGTGTAGAGCCAGCTGGATTTCTCCGGGACCATGAGGGGATCCATCTGATTAGGGCTTCTGAAGCCGAAGGAAACTACAGAGAGATGTAACTTGGCTGACTCTCAGTTCATTATTTTCTCTTGGTAAGAGCACTTCTCATATTGGACAATCTTTTCTTCACTGATTTAGATATTATTTTAGATGCACCTTTTCTTTTTGTTATGGAAGCTTTATTTTAAAATAAAGTTAACCTAAAATGGGCGTATTACTCTCCCCCCGCCCCACCGCTAATGATTTAGAACATGAAAATAATCCACAAGACCATGGGTGCTGTCTTCAGCTACAATTACTACTTTCTTAATTGTCATGGAAACATGATTTATTATTGGATGGTTTTTTACTGTCTTATGCAAAGATTTCATATGAGCCGCAATACACACTGTTTCATATGGGTAAGTCTCAATATTATCTGACAAAGAGAGCTTCTCTGCCCAAGTTTATGAAAAGTACATTTTTTTTTAAGTCACTGTCTTGCCCAGGCTGCAGTGCAGTGGTACCATCATAGCTCACTGCAGCCTCAACCTCCTGGGCTCAAGCAGTCCGCTCACCTCAGCTTCCTTAGTAGCTAGGTGTTTTGGTTTGGCTTTTTATCCCCACTTGAATATCATCTTGAATTGTAATCCCCAGATGTTGAGGGAGGAATCTGGCGGGAGATGATTGGATCATGGGGGTGGTCTCCCTTATTCTGTTCTAATGATAGTGAGTGAGTTCTCACGAGATCTGATGGTTTTAAAAGTGTCTGGCAGGTTCCTCCTTCGCACATTCTTCTCTCTCTTCCCACCATGTGAAAAAGGTCCTTGCTTCCATCCCGCCACCTTCTGCCATGCTTGTAAGTTTCCTGAGGCCCCCCATGCCATGCGGAGGTCAATTAAACCTCTTTCCTTCTTAAATTACCCAGTCTCGGGTATTTATTTATAGAAGTGTGAAAACAAACTAGGACACTAGGACTACAGGCACATGCCATCACGGCCAGCTAGTTTATGTTTATTTTTTAATTTTTGTAGAGATGGGGTCTCACTATGTTGCTCAGGCTAGTCTCAAACTTTTGGCCTTGAGCAGTCTTTCCACCTAGACCTCCCAAAGTGTTGGGATTACAGGCATGATCCACTGCACCTGGCTGAAAAGTTTCTATTGAATGGAAAGAACAATGCTGTGAAAATATATTTTATTAATGTTCAGGAAATTGTGGAACTTGAAAAACTCTAGCTTTTTAGCAGTTTTAATGGCTACTATGTGCTTCTAAAATTTGTACCTGCTTTTTTGAAGTGTTATATGCATTTTTGTTTGTTGATGGTGGTGATGTTTTTGCCGTTGATCTCACCTGCTAACGTGGAAACATTTCAAGAAGTGGAAAAATGTCTTATTTTAGTACATACTATGGTGTCAGCTACATTAAAAAAAAAAGCCTTAAAGAATGTAGCTTGAATTGAGGGTTGCTATGACTTTTTGTTGTAGTAGATTTATGAATTGTGTATCATCATTTTCCTTCAGTGGAAAATTCAGTAACTAGTATGTTACTGGTTCCTGGATTCCAAGGGAGGAGAACATGAAACATTGCAATGGAATTAAACTCCAATGAGCTTGACCCAGCTACGATGTTGAAGTGAGGGAATACATAAAGACTTGGGTGTATGTGTGTGATCTGTTGGTATTAAAGTGCCAGGATTACAACATTCTATGAAAATGGCTAATCATATTCAATATTTATTTGAGACGCTTAAGATGCATGGTTTGGGTGGAACTAGGGTTAGGGGGCTGCTGTTTTGAACAGCCAAACTAGAATTCTGCTCAATTATCTCACACAGGCACACTTCTGAGGCATTTTTTACATGATGCCTCAAGAAAGCTTTGCTCCATTTTGTATTTCAGCATGAATACAAATTTTTGAAATTTCCACAGTAAAGTGTTTAGACTTACCAAAAGGTAGGCCTTGTTATAATAACACCAGTAGGACCGATGTAGTCATTTCTAAAATGATTCAAGCACTTTATGTTTCTGGATGAGCTATTAGATCTTACCTTATGTGTCTGGATAAGCTATTAGATCATTACATATTTTAAAGTGAATTTTTGAAATTGTTGGTTCATTGTTTAAATTTTCAATTTTGTTTCTGTTGCATTAATCTCTGAGATTTGAAAATGAGAAAAGAAAAAAGATGGATACACATTAATGCTTTTATACCTTCCTTTGTAACAGCAATTGATTGTGCACTTGCTTTTGGCTGTAGTTAGTCCTTTTCTTAAATTAGTTTCTGGTATGGATGTCTACTTTTATTTAATTTTTTTTTTTTTTTGAGACGGAGTCTTGCTCTGTCACCCTGGCTAGAGTGCAGTGGCGCGATCTCGGCTCACTGCAAGCTCCACCCCCGAGGTTCAAGCAATTCTCCTGCCTCAGCCAGCTGAGTAGCTGGGACTACAGGCACCTGCCACCACGCCAGGCTAACTTTTGTATTTTTAGTAAAGACGGGGTTTCACTGTGTTAGCCAGGATGGTCTCAATCTCCTGACCTCTTGATCCACCCGCCTCAGTCTCCCAAAGTGCTGGGATTACAGGCGTGAGCCACCGTACCCGGCCCCACTTTTATTTAATTTTTATTCAATTTTACATTTTATATGCCTTGTTACTTCATTTCTTAGCACCAGAACTACAAGTTTAATTCTTCAGACATCTTCTCTAGCACCTCATAAGGTATTCTTTGTTACTTGGTGATAGAGAACTATGTAATTTGATTTTCTTCTTTTGCAATGGAGTGTTCAAATACGTCGTTGCTTTTAGGTGAGGGATGTGATTAATTAGAAAAATGAGTGGATCTTAGCTCAATGAAATTTAATCAGCAGAATGGAATTTTCCATTCAGAGCAAATGAGTTCCTAGGACTGGACACACCTAGATCTGCTGACCCAAAACCCTTTATAGATTTCATTTCTGAATGAGCTATTAGATCATTGTATATTTTCAGGTGAATTTTTACAATTGTTGATTCATCGTTTAATTTTTAGTTTTATTTTCTGTTGCATTAATCTCTGAGATTTGACATATAGAAGAAACTCTCATGCCAGCCCCAAACGCTTTCCCTATCTCCTCCTCCCATGCCTTCCTGGAGTGGAGGGAACGTCAGGCATAAGCAGAGCCCAGGAGACACTCATAGACATTCTGAGAAAGCTTTTCTCTGTAGAAGGGACCAACACATCTTGCACCCTCTCCCTCTCTTGCCCCCTGCCTGCATGTGGGTGCAGGTGCTTTTGTCAGGACCCCACTGCTTATCTCAGGTCAGGAGCTGGCAAACCTATGAACAAGATGGAAACCCAACTGCTGACCAGGGTGGTGTTCTGACAGGAGAGAAGACTTGAGCCCTTATAGACACTGTTGAATCACTAAGCTGTAAACAATTTTCTTTGGTCTTCTTGTCTGGTAAAATCAATTCTCTTTCATCCTTTTTAAAGACCTCAGTTTGGGCTTTAGAATCCATACTGGCAAATGCTTCCTCACTAATATTGTGAGATTTAATTAGAGATAGCATTTTATGTGCTCACCTAAAACTATACGGTAGACACAAAGGAGTCTGGGTCTCAGATCCCAACACGTGGATTATAGAGAAGGCAGAATGCTATAATGCCTTGAGGGTGAGCCATCCATTATTTGGGGATTTGAAAAAGGACAATTTCTGTTTTATGTTTCTGTCCTCCTAAATGGAGTTGAGAGACAGCTTCTTTTCTCCTTAGCATTTGGGCAAGAACAGAATCCAGTAAAACCACTGAGGAAGGTCATCATTGCAGCGTTTATTTAACATGAGTAATTCTAGCATGAGCTGGCATGCCATTTACATCCATCTGTTTTAAGTGTTTGCAAGCAGAATGGTAATAAGAAACTGGGGTAAGTGTTAAAAATAATTATATGGAATATAGATTGCCCCAGATGCACTATCTAATGCTGATGGGAAAGGAGAGAGCAGGGGGTACCTGGAACCTGGACTTCTCCTTGGAAACATGCCATGACCGGGTATGTTACTGGATTGCATAGGTGCAGAACATGGAACATTGCAGTGGAATTGAACTCCAATGAGCTCAGCCCAACTACGATATTGGAGTGAGGAATGCATGAAGACAAAACCTTTATTATAAGTCTGTGTGTGTGTGTGTGTGATCTGTTGGGATTAAAGTGCCAGGATTACAGCATTCTATGAAAATGGTAGTGGAGAAAAGGAAAGGTAGAGGAAAAGAGAAAAACCAAAGCAAGAGGAAAACCACTGGAAGAAAAGAAGATGGGAAGGAGAAAGGGCATCTCTGAAGAATGTAAGGAGTACAAGATCCCTTACAGGCAGTGAACACATAAGAAGGCATCATTCACCAGAAAGTCATACCAGTTTATGTATTAAAACTGGGAATGGCAATGATAGGCATTAGTTAGAGATTATGCTTTAAATTGTATGCATTTGCATATTTTTATATGTTTTATTTAATTTTGTTTTGGGGGGGGGACTGTATCTCACTCTGTTGCCCAGGCTGATGTGCAGTGGTACAATCCTAGTTTACTGCAACCTTGAACTCCTGGGCTTAAGTGACCCTCTCACCTCAGCCTCCCAAGTAGCTGGGACTACAGGCATGTGCTACTATGTCCAACTAATTTTGTTATTTTTTTGTAGAGACAGGGTCTCAATGTATTGCCCAGGCTGGTCTGGAACTCCTGGGCTCAAGTGATCCTCCTGCCTTGGCCTCCCAAAGTGCTGGGATTACAGGCGTGAGCCACTGTGACCAGCCCTTTTGCATATTTATTGTTTTTGTTTGTTTGTTTGTTTTTTGAGACAGAGTCTCACTCTGTCACCCAGGCTGGAGTGCAATGACGCGATCTTGGCTCACTGCAACCTCTGCCTGCTGCGTTCACGCGATTCTCCTGCCTCAGCCTCCCAAGTAGCTGGGATTACAGGTGCCCACCACCAAACCCGGCTAATTTTTTGTATTTTTAGTAGAGACAGGATTTCACTATGTTGGGCAGACTGGTCTCGAACTCCTGACCTCATGATCCGCCTGCCTCATCCTCCCAAAGGGCTGGGATTACAGGTGTGAGCCACTGTGACCAGCCCATTTGCACATTTAGTGTTTATTTTCTTAATCAGTATCGAAACTGTGAAAGGGAATGTTAAAACGGTGGAGCCAGGTGAAAAAGAAAATCCAAGAGTCAGAAGAGAGCATCCAAAGAAGAAGGCAGAGGCAATAACAAGTAGACTCTGAGACTGAAATTAAACTGTATGGCTAGAAGATGGGCTAGCATAGGACAAGATGAGGTAACATGCTAACATGGAAGATTGAGAAGAATTGCAAATGAGAAATCACGGATAAAACACTGACCGCCTAATAGGATAAAAGCAGAGGATGTTCATAAGCAGCTGTCATCACCAAGGAAGAGGAAAACATGGGAAAGGTTTTGCCCTCTGAGCAGAACAATCCTGCATGTCAAGGGGGAGCCTCATATACCATGTAACCTCATGTTAAACCATAAATACTTACCAATACCTCTTACAGTGTGACAGGACACAAACTATTAAACCTGATGCAGATAATGCCTTTTAAAATGAGTATTATATTTGATTATTATTTCTAATAATGTTATAACTATGTTTAAACCATCCACTTTATTCCCTAGATGAAATATAATTGAATTAAATGTTAAACATATTTGACATGCATTTCTCGGGGCTTTTGATTTAACATTTTAAAATATGCAATTTAGCTATTTTAAAAAACAGTCTTAAAAAATAACATAGTATATCAAGATAGGCAGAAGGAAAATTTAGGCACCAAATAATAGAGTACATGTTTCCTATTATGTGTTTTGGTTGGGAGATGATCTTTGGAAAGTGCTGATTCTGTTTTTGTTTCCATAAAACAAAATTTCCAGAGATTATATATTGGATTCTGCTTGAAAGAGTTCAGTAGACATTGCACTTCTATCACACTGATAGCCCAGGAGGAATTTTAACTATGTAATTATTTAACCGCAAAATTTTCCACCTTCTCCCCTTAAACATTTGGCGGATAAATTATGATAAAAGCAGTCATGATATGCAGTTCGGTTTCATAGTTTCCTTTCTCTTCCTTTTTGCTATATTTCCTAAAGTTCTATTATGGAGAGATACCAGTTTTAAATGTCAAGCAATGTTAACATCTTTGCATCTTTATCTTTTCCTATCCACTCTTCTCTCTTTTCTTTTCTTTTTTTTTTTAAGGGCCAGAGAGTGACACTTAGCCAATACTTAACCAGTACTCTCTTTCTGTTTGTTTGTGGGAATTTTATATCTATTTTTTCTTTTTCAATTTTTATTTTAGGTTCAGAGGGTACATGTGCAGGTTTGTTACATGGGTAAATTGGGTGTCGCTGGGGTTTGGTGTACAGATGATTTTGTCACGCAGGTAGTGAGCATAGTACCTGATAGGTAGTTTTTTGACCCTCAGCCTTTTCCCACCCACCACTTTGAAGTAGACCCTTGTGTTTATTGTTCCCCTTTTTGGGCCCGTGCGTCCTCAATGTTTAGATCCCACTTGTAAGTGAGAATATGCAGTACTTGCTTTTCTGTTTCTGCATTAGTTCTCTTAAGATAATGGCCTCCAGCTGCACTCTTGTTGCTTCAAAGAACATGATTTTGTTCTTTTTATGGCTATATAGTATTCCATGATGTATATTACACCACATTTTCTTTATCCAGTTCACCGTTGATGGCCATCTAGGTGGATTCCATGTCTTTGCTGTTGTGAATAGTGCTGTGCTGAACATGCAGGTGCATGTGTCTGTTTGGTAGAATGATTTATATTCCTTTGGATAGATATCCAGTAATGAGAATGCTGGGTCGAATGGTAGCGACTTGTCTCTTAATAGTTTTTACTTTGCCTCGATCTCCTGATTCTCTCCCTTTTTTTCCTGGCCATTCCCGCTGCACTTGCCTCATTTGCTATTGATGACATGCTTGTCCCCTGCTTCCATAGATGTGTCCACAAATGCATGTGCACACGTGCTTCAGCTAAAGATTCCTCAGCTAAAGATTCTCCCTCTCCATCAGGGTTTCTCTCTTTAGCTCACCTGCCCTTCTCTACATGGTTTTAAAGTGAGATGATTGTAAATGTGTTTTTCACAATGGAAATTCTCCCAGCGGGCGGGGAGGAAAAAAGACATCTTGAAATATTTTCTGAGAACTATGAGGACCGGCAGAGTTTGACATGTTTTTGAGGCGATAAAGTCATGTGTCCATCTGTGAAAGACAGGCATTGGCTTTATCCACATCCACACAGCCTTCCCCGCTGTGTGGCTTCATTATTGATTTGCTGTCATGTAGAGTCGATAATGAGAAAACCTAGGTAGCCTTGAACCCAACTTTGCAAGAACCTTTTAGGACTCTGGGACTTCTAACCCTCTAGGAAGGTGGAGTTAAGGGGATATAGGCACAGAATGGGGCAGAAGGGAAAGACATTAAGAGACAGCCTTTAGCAGACCAGAGAATACATGCCGTTTATCAAATTGTTAGATGTCTGTGCACCAGGAATGTTGATTCAATTATGGTATCTAAAAATAGGACAGAAATAAGGAGGAAATAAAAGGAAATGAAATAGCAGTTTACCTCTGGCAAAAACAAAGAGCCCAATCAGAAAAACTAGACAAAGCCACCTGTAGGACTGGAAGAAACCATGTGAGTTAGGTATCACTAACCTTGGAAGGACAAGGACTTCCTAGTATTTTTGTATTTTGTGAAGCACTTTCTCTGCATTTTCTTAATTTGTCCTTAAGTGATTATCTCTCAACCAACCCCAAAATTTGACTCTTCAAATCATTTATTCTCTAAGATTTTTAAGCATTCAACTGTAATGGCTTATGTATCAGCATAGTCTTATATAATTCTAAAACAACATTCATAGCATGGTATCTTGTAATATTTGACTTTCACTATTAATTCTTTCAGTTATTATTTGAGTGCCTGTCACATGCCAGGTATTGTTCTAAGCTTCAGGGATGCATCCATGTACAAAATAAATAAAATTTCCTCCCTTGTGCCACTGATATTCTATAGGTGGATGGAAAACAAACTTAAGAGTTAAATAAATTAGGTTTTATTTAAAGACAGGGTCTTGCCCTGTCATTCAGGCTGGGTGCAGTGTTTAATCATAGCTCACCGTACTCTCCAACTCCCGGGCTCAAGCAGTACTCTCACATCAGCCTCCCAAGTACCTAGGACTACAGGTGTTGCCACCATGCCCAGCTATTTATTTTCTGTATGTTTTTCTTTTTGTAGAGATTGGGTCTTGCTATGTTGCCCAAGCTGGTCTGGAACTCCTAGGTTCAAGCAACCCTCCCTCCTTGGCCCCCTAAATTACTAGGATCACAGACATGAGCCACCATACGTGGCCAAAGTTTTGTATTATTTTATAAGGTGATGAGTGCTGTGAAGAAAACTAGAAGAGGATAAGTGGAATTAGAATTGCTAGGGAAGTTGCAGTATTTTAAGTAGGGTGGTCAATGACAACCTCAATGAAAAGGGGATGTGGGAGTAGAGAATTGAAATAGCTAAGGGAAAAAGCCATGATGATATATGAGAAGGATGTTCCAGGCAGAGGGAACAGCCAGTGCCAAGGCTCTGGGGTAGGAACATCCCTGTTCTGTTTAGGGCAGAGCAGTGTATTAGTCTGTTCTCAAGCTGCCAATAAAGATATGCTCAAGACTTGGGAATTTATAAAGGAAAGAGTTTTAGTGGACTCACAGTTCCACATGGTTGGGGAGGCCTTACAATCATAGCAGAGGGCAAGGAGGAGCAAAGTCATGTCTTACATGGATGGCAGCAGGCAAGAGAGAGCGTGTGCAGTCCCTTTACAAAACCATCAGGTTTTGTGATACTTACTCACTATCACCAGAACAGCATGGGAAAGACACACCCCCATGATTCAGTTACCTCCCATCAGGTCCCTCCCACGATTATGAGAGCTACAATTTAAGATGAGATTTGGGTGGGGACACAGCCAAACCATATCAAGCAGTAAGATCCACATTTCTAGAGTATCAGAGTATGCCATCAGAATGGCAGGTATCAGAGTAGGGTGGTGCTATCGAGAACTTTGTAATTCTGAGAACCAGGGAGAACAAATGGAAGGATTTCAACAGATAATTCATGTGTCAAGGTGTGTTTTAAAGGAGCACTTTGCTTAGCTGAGGCTTGTCTGTAGGGGCAAAGGTGGAATGTGGGAGACCAGTTAGAAGGCTGATGTAAGAGTCAAGATAAGAACCTACAGCTGGGAGGTGAGAAGTGGTTGGAGTTTTTTATACATTTGAAGTAAGATTTGCTAGTTATATGGATGTGGAGTGTGGGAGATCGAAGGAAGTCCAGAGTTTTTGGCCTAAACACTGGAAAAGGTAGAGGTGGTCACAGGTGACATTGGAGGATGGGCTAGTAGAGACATTCTTAAGTTATCATCAAAGTTTAAATGTTTGAGTTTGAAATGTCTATGAGACATCAAACGGAAGATATCCCATAAGGAGATGGATGTCAGAGTCTGAAGTTCAAGGCAGAAATCTGTGCTGAAGAGAAAAAATGTCAGCCTAGATAGTGTCGATGGTATCTAAAGCTATGAGGCGGAATAAAATTATCAAGAGAGTTCTGTGGACAGAGAAGAGAAAGGACCAAGGCTGGAGCTTGCCAACAATTTGAGATTGGTAATAATACGAGGAACCTGGAAAGGAAATGAACATAATTGTCCAGGGTGTAAAAGAAAGTCTGGTAATGTGGAAGTGAAGGGGGGAAAAAGGCATTTCAATGACAGAGAGGTAGTCAACTGGGTGTAATTCAAATAGGTCATAAAATGCACATCTGCTGCTATGGTTTCCACTACAGATGCAAGGAAAAAGTGTCCTCGTCCTTTTGTCTGTCTGATTGTGGCAGTTGAGATTGAATAGAGGTAGACAGAGGGGAAAAAAGAATGAGGAAAATTGAGAACATAGCAATGCAAATGTCATTTTTGACCTTTAGTAGAAAAGTAATAATTTTGGTGGAGTGTTGGGGGTAAAAGCCCCAATTGGGGCAGGTTTCAGAGAGAATAAGAGCAATAAAATTGGAATCAATATCAATAAATATTTTCAAGGATATTTTCAGAAAAGGAACAATATAGACACACTTTTTTTTTTAAGATGAGAAAATTGTTTTATTGCTTTTAAGATGGAAAATCTAACCACATTTCTGTGTGCTGTAGGGTTGATCTAGAGGCGTGGTGTTATCAATCAGTACAGTGTATAGTGTGCTACATTAACAAATATCCCTAAAATGGCAGCGACATCCACAGCCACTAAAGTTGATTTCTCGCTCATGTTCAAAGTTCGCTAAGGGTTGACTGTGGCTGTTTTCTGTGTATTCTTAATTCTGGGACCCGGGCTGATGGAGAAGACTCATTTATTCTTATTATTACTAATTATTTTTGTTATTTTAGCAAAGGGGGAAAATGGGCAGAACCACATTATAGCTCTTAAGGTTTTCGCTTGGAAGTAGCCCCACTAATTTCTGTTCATGTTTCATCTGCCAAAGCAAGTCAATTAGCTATAACTGAAGTCATGGAAGTGAGTCAGTGAAATTCTTTCGAGTTAGGGACAGGGAAAGTCTTGCAAGTGTGTATTTGTCCCCTTGAGAGGTGTGGACAGTTTTTTACACAATAATACAACATACAAGAGGAAGACAATTCTGAGGATATAGCAAGAGCAAGGTGTTCTATTGTTGGGTTGTCAAGAGTTGATGGAGTTTGATGGGTGAGAGTCAGCCTTATATTGGGTTCCTATCATTATTCTCTTATGAAAAGAGGAGGCACAAAAGATGGGGCCATTATTGTCACATGGGTAAATGGGTTAGTGGTGGTTTGTGCATGTTTTCTTGAGATAGAATTTCTTCAGTGTAGTAAGAAGCCAGGTCATATTCTAACAGTGAAGATGGAGCACGAGGGATTGGGGATTAGAAGAGGAAGAAGAAGGTGCTATTTAGCAGAGCCTTTAAGGGAATTCATCAGAGAAATTTAGTATGATATACAGGCATCTCGATTAACCTACTGGAGGTTTGTGTTCATGAATTTAATGTGAGATAAGTCAGCATGATTAAATATCTTCTTTCATCTGTGCTGATCAGTAAAGGTGAGGCGGATGCATGCTGGGTGGGGAGGTGGATTTCACCAGGGTTGGAGTTTTGCCAAGGAAGAATCAAGAATTAAGGCTGGATTAGAATTGAGGGTGTCTAAAGGATCGTGGATCTGCTATGACTCCACAACTCTAAGAAAAGAAGATTCGGTACCACCATCCTCATTATGGAAATAACAAACGAATGAAACAAAACCATTTGTCACTTTCTACAAGATTCAGAGGGCTTGTATGTCTATGATCTCAGGCCTCAAAAAGAGTAAATCAGTTACCTTTTTCCCACATAACTCTGTGTGTGTGTTAGTACAATTTTGTATGTTTGCCCTAGAATGTGAACCATGAATTTGTGAAATGAAAGCAGTGAATAGGAAAAAAGGTAAAGATACAGTTTTGTATTATCTGTAGCAAAAATATTACCACAGCTATGTAATCCACAAAAATGGAAGAAATTTATTAGGTATTTAATTTTTATCCAAGAGTAGTAAAATGAAGGCAGCTATATAATTATGTAGGTGACTGTTAAAATATTAGACTTTTTGTTGAAATTTTTTGGCTCAGAAAACAGGTTTCATGCCATGCTGAAAAATTACTTAGTTTGATGAAAAAGTAAACAAGACATGACAGTGAAATCATACAGTGTTGAAACAGGAAATAGCTAAAATGTATTTTTCTCAGTAAATAAGTGGCTGGCATAAGTTGTCCTCATTTTGGGGTCAAGATCTTATTTTGGTGTCTCAGCTGAAGATGACCTCTTCACAATCCATTAGGTATTGTGACACTGATTAATTATTATCAAGCAGAAAGTATTTTTTGGAAGTACTTTGCACTAGGCAGGTAAGGCAGTCGCTACCACAGGGGCACAGGTTTCGAAGCAGTTCAGGAGGAGCCAACGTCTTGCTGAGAAACCCAAGGCAGACAGCAATTAGAGGATAAGATAATGTATAATTAACTGCCACCGTGTGTGGGGTAGACAATTAGAGAACAAGGCAACACAGATGTTGTAAGGTGCTGATTATGGGTTTTAACAATAATGAAAAATGGAAGACAACATCATCAGCGTGGGCTGACGCTGTCAGGGGTGGTGTGTTTTCTCATGTGCTGTTACCCTCTAATCAGTGTTGAGTTGGATAGTATTCCCAGGAATGGCTGTTTGGCTTCGCTTCTCTTACCAGAGAATTGCTCTGCCTTATAAATGTAGAGACTGACATGTAGACACACTTGGATCATGAATTTCCATTCTACTCTACAAGAAGTACAGCTGCAAAGAAAATCAAATCATGTTCAGTACCTTTCTGGAATTTTCCCAAGTACTCAGTAGTCATTCTAGCTCACATCTTAACTCTGCTAGGGTTCAATAAGTATACCAAATGCATATTTTTTTTTAGCTAATTCCAAAATCTAATTCACTTTGATCAATAGTCATCTCCTATGAATTCCTTGTGTTTTCTTCACTATAAAATATTTTTGTGATTCATCTTTCAGTAGACGAAAGGTGAGGTACTTTGAGATTATATTTCTACTAAATCATGAATGATTCATTATTTTACTGAAAGTAAACACATCCATCATATTAAATCCATATCATGTTCTGTTGTATATTGTCACTTAAGTGTTTTTATTATTTTTAAACAGGTTGTATAATTGCATAGAGCTTCAGGCTATCTACATAGACAAAATATCTGAATAAAAGTACAACGATCATATTTTATCTTGTCAGTTTAAATTATGTTTAATGATTTTAATTCCAGGGAAAACTCTAATGTACCAAGTTACCAACTGAAATGTGCCCAGTATCAATCCTTTATTTTTAAATATAACATTGTAAGTTGTTAAGTAAGTTGTTAACTCTTATCCCTAAAAAGACATAATGTTCCCTTTTCTTATCATATGCTAAAATAAAAATTTCTAACAATGAATGTGCCATTTTTATAAGCCAGCAAACTATGCAAGTAAGGATCTCAATAGAAGATTTAAACAAAATAATTATTTTGCTCCATATTCTGTTGCTTTTGTTTTTTGATGAGATAATTAATTTTCATGGAATTTTAAATGATCAATTTGTAGTAAATTTTGGGAAATATGTCCATTATTTAATCACAGATTTAGTATCTTAAACACATTGACAACGTCAAACTTGTCTGCAGCAAATGGTTACTGTTAAAAATTTGCCATAGGGGTGAGAACTGCAATTTATACTATTTCTAAGCTATCAATGCTTCAATTATTACATGTGTTTATATATATATGTGTGTATGAACATGTGTGTGTGTGTGTGCATGTATACACAAATTTTAAAGTAATGGCTTACTGAAAGGCCTTTTTTTCTCTTCATATGACTAAGATATCTGAAATTCTGCCCAAAATTGCTAAGATTATATACCCTTCTGAAAAATTGCAATGTGTTTATGACGTATTTTTATGATATTTCAGTACCGGATATGTTCATTACCCCATGTATGAAGTCTTATCTTGTGATGATGAGTTGATCAGACCTATTACATTGAGAATATTTTTAGGTATAAACTTTATATAGTCTCTGATGGTGAGTGTGTAGGTAAATTGCTTTGGGCTCACCTGATTGTATTTTCATTGTTGTTGACTTTCATTATTTCACTAATTTGGGAGCAAGGGCTTCTTTTTTATGGTCTATTTCTAGATCATCTTCCCTTAGATTACATCATGTAATGAACTGGCAGAAGATATTAAGTAGATCTTATTCAAACAAGAACTTTGAACCTAAATGGAGATTTATCAAGCTAAATTAGCCTAATTGTCTGTAACAATGACCACAGCATATTAATAAAACCTGTGACCCTTACATATATACATGTGCATTTTAATGTTCTTCCACTATGAAAGGCATTTTGTGATTTAATCTGCTTGATGAACGATTAATATGATATTCACTAATTTTTACTCATCTTATTCTTAATTCATCTAATTTATCTAATTCTTAGTAATCTAAATGATTCAAGCCTCTTACAGATTTTTATCTCTACCCAGTTTTTCATCCAGCTGTCCGTGTGGTCATCTCTGCCTTGGTGTGCTTGAGAATTATTTCTGATTCTATGACACCAATGCACTTTGCAGTCTTTGAACTTGAATTGGCAGAATCAAGCTTCCTCTAGACAAATCACTGAATCTCTTTTCTCACGTTAAGGTTTGTAGGAACCCTATTCTCAAAGCTGCCAAAACACTACTGCTTAGTCTATGCAAATCAACAACTACAAATGCACGTCACTCAATCAACATTATGAAACTCCTTTTTGGAATGATTGATGATCACAAAATGTGATCTTGTGACAATATGATATATTCATTTAAGCCACATTGAGGTTTCAAATTGGCACCATTGACAACGTACCTCTTTCATGCTAAGTGTAATAATTTGTTGCCTCTCATTTTCCTATGCTGCTTCACTTCATTAAATCTGAATAATTAAAAATTTTCGTAGCATCGCCAAAGTCACTTCCCAGGAGCTAGGGAATGTGTCGATCTGTACACTGATCCAGTTCCTGCTGACGTTTGCTTGGATGCAGAGGCCATCCATCGCTTTCCATTGATTTTTGTCAATTGATGCTTTTCTTCCTTCTTTCCTGGTGACTTAGGAAATGTTCTGAAACTGTGCATTCAAGTCAACACATGTTAGATTCATAACTAGGATTCACCTTCACAGTGGACTGGTCCCAATTTGCTGTATTTTTATTCAGCCTGTCAACTCACACTATCTGACTAAAAGACGCTAATGCAGTGTTGGCCAGTCCCCTGTCATCTCTTTCTAATTGTTTGGTCTCAAAGCAATGGTGCATGTTACACATATCCATTTAACTGTCCAATTAACGCATGTTTCTAGACAATTCTGATAGAAAGGGTCTCTTTTCTTCCTTCAGCCCAAACAAAGCAAAACAAAACAAAAGGGCACTTACACGATGTTGATCTATGTTTTATCTTTTTTTTTTTTTGAGATGGAATCTCCCTCTATCACCCTGGCTGGAGTGCAGTGGCGCGATCCCCGCTCCCTACAACCTCCGCCTCCCAGGTTCAAACAGTTCTCCTGCCTCAGCCTTCCGAGTAGCTGGGACTACAGGCATGCACCACCACACCCGGCTAAGTTTTGTATTTTTAATAGAGATGGGGTTTTGCCATGTTGGCCAGGCTGGTCTCAAACTCCTGACCTCAAGTGATCCACACACCTTGGCCTCCCAAAATGCTGGGATTACAGGTGTGAGCCACCACCCCTGGCCTGTTTTTGTTTTATCTTAAATCTCTTAGGCTGAGACTCATATGGTCCCACTTACCCATCTTTTTACAGCATGAAATTGTCCAGTTAAAATTACAGCTCTTTATTAATGGCCTTAAGACTCTTCATTTTGAATGGATAAAATAGTAATAGGCTGTGAGCACCAACAGTATTAATGTATCATTCATGCATGATATAGTAGTGTTGACATCTTTCTTTTCCTTTTCTGTTTTTAAATGAAGTTCAGGAAACCAATATGAAAGGTAAGAAATTGCCAACATCTTGGACTATCAAATCATGGCAGACAATGAATTAAAGAATTCAACAAATCTTTGGCAGCATCAGTTTCAAAGGTATTTAGATACAACCACCGTGTAATTCTACACAATTTAATTAAATCATTTATCAAATCCTCTACAACTTGAATAATTTAACTGATATCAGAATAATCCATTTTTCAGATAATTATTTTTATATTTAATGTGTTAAATATAAAAATATGACACTTCTCTTGCATAATTTGCAGAATGTTATTTATTTCATTATTTTATTATTATTTTTAAAATTTCAACTTTTATTTGATACATGTACAGATTTATTAAATGGAAATATTGCCTGATGCTGGGGTTTGCAGGAAGGATCCTGTCACCCAGGTAGTGAGCATAACATCCAATAGGTAGTTTTGTAAGCCCCCCCACAACCAGCACCCTATAGTAGTTCTCAGTGTCTTGCTCTTTTGCCCAGGTGCAATCAAAGCTCACCACAGCCTCCAACTCCTGGACTCAAGTGATCCTCCTGCCTCAGCTTCCTGAGTAAATAGGACTACAGATGCCACCATGGCCAACTAATTTTTTAATTTTTACTTTGTAGAGATGGAGTATTGCTATGTTGACTAGGATGATCATCCACTCCTGGCCTCAAATGATCCTCCCGGCTAGGCCTTCCAATGTGCCAGGATTAGAAGTGTGAGCCACCTCGCCCAGCCCCAATGCTTGATCTTTAAGAGCTTCAGGCAGTTGAAGGGTTTTGTCTGCCTGCCACAGCCTTCCATCTTTTTGAGATGTGTTTACCTGAGACAGCTAAGTAGGTGACAACCTGAACTACGGTTGCTGGCAATTGGAAAACAGAAGATTGCTCTGTTGATCCATTGGGAGAAGTACAGTAGTCTGTAGAGGAACAGAATCCCAGGGTTTTTTTCTGGCATGGAATCACTCTAGAGAGCCACATTAAAAATTTAATTCCTGCTGAGCACAGTGGCTTACGCCTGTAATCCCAGCACTTTGGGAGGCCGAGGAGGGCGGATCATGAGGTCAGGAGTTCGAGACTAGCCTGACCAACATGGTGAAACGCTGTCTCTACTAAAAATACAAAAATTAGCTGGGTGTGGTGGCGTGCACCTGTAATCCCAGCTACTCGGGAGGCTGAGGCAGGAGAATTGCTTGAACCCGGGAGATGGAGGTTGCAGTGAGCCAAGTTTACACCATTGCACTCCACCTTGGGCAAAACAAGCAAAAAACTCCATCTCAAAAAAAAATTAATTCCCCTTTGACTGTTGATTTTATTTATTTATTATTATTTTTTTAGAGACAGGGTCTTGCTCTGTCTTTCAGATTGGAGTGGTATGATCATAGCTCACTGCAACCTTGAAATCCTGAGGTCAAGTGATCCTCCCACCTCAGCTTCCCAAGTAGCTTGGTTGACAGGCATGCACCACTACACCTAGCTAATTTTTCTATTTTTATTTTTGTAGAAACAGGGTCTCGCTCTGCTGCCCAGTCTGGTCTTGAACTCCTGGCCTCATACGATCCTCCCACCTAGTTCTTCCGAAGTGCTGGGTTTATAGGTGTGATAGTGCCGAGCCATTTGGCTGCTGTTTTTACATTTATACCATTATCTTCATCCTAAATAGGAATTCTGATAGTATTGTTGGCAGAATAGGGTCAACTGGAACACACATTTTTGTTCTCTAGGTAAAGATGATGAAACTTAAAATGTAGCTAATGTTATTCCTGCAATGAATATGTCAATTTCTAATCTGGGGACAAAAATAAATAAAAAAAAAGTTGCACGTATTAAACACCTTCTTGACTAAGTGGCAGCTGTAATGATTTCACTTGGGGATAGCCATTGCTTCTTAACTCATGCTAACAGTGCATTAAAGCTATTGATTTTTAGTGGCTGCTGTGCTTTCGTGATTGTAGATCATTTCTCTCTTTGGAAACTCTATTTGATGACAAAGCTGGCTCTGTTGCAGAGTAATGATAAAAGAAAGGACCTACCAGAATTTCAAGTGAAATGTATAACATATGTGATAATGCATGGTGACTGCAATGATTATTTCCCGATGTTGCTGTTTAATAGCCATGAAAGCATCCTACTGAAATAGAGTATTTCTGCTTTGAATGGCTTAGTTAGCTCAAAAATTTTGAAAGCTTTCTCAGTAAAGCATGGTGCCAGGCACTGAAAGATTCCTTTTGGAGGAGCCAGAGTCAATTTGGATGATGTTTATAAAATGCTGCTGGAAAATTGGGTGGTGTTTTCTAAATGATCTTCCTAGTAATGATTTATGCTGTAAATCAGAAAGGTTGCCATCTCTCTGGATGGAAATGCATAGTCATATGCCCGTAAATGCAGGGATTTGACCTCCTATAAAAAAGCTCTCTCTTCCCCCTCATTTATGTGATGATTGTATACCATCTGAGCGCTGAGAAACCCATTGGCCATCTTCCACTTGTGTGTGGCTGGAGGTGCTTGCTGCAGCTCTGTGATGCCCTGAGCCAGCATGCTCGTGGAGTTCCAGTCTGCTGCATGAACAAGTGGAGAAACATGATCTTCCTAAACTGCTCACAAGCTGCTAAATGAGTGATTTGTGTTCCCTTTGAATTCATGCTGTAAATGGAAATGCTTGCTCCTTCCCGGGTTATTACTCTGTGTACACGCCATTTGAGGATGCAGATAATTGTTGCATCTTCACTGAAGCATCCCATCTTAGTCCAGATTTCCGTTTTCACAGACCAAAAGGGCAAAGTCAGACTTGGCAGACAGCGCAGCTTCAGTCTCATGGGGGGATTTCTTTGTCTCATCAGCCTCAGTCATGGGCTTTCCAGCCATTATAATTTCACATGTAATATGGTGGGTGTCCATCTGAGCAAGTGTGGTGCCTCAGTAGGGTTGGAGGAGGCACTTGGAGCTGATGTAGAGAAAGGAGAGTGAATTAAAAGTGGAAGGAGGCAAATTAAAAGAAGCGAGGAAACATTCTTTTTCACACCAGAGAAACGTTTTCAAAACACCAGGGAAGCCTCAGAACCAATCCAGGTACTGCTTTTATTTCTGAACTCTGTTATAATTTGTGATGTCAGAAGCTTCTATGGAATCTACTGATATGTGCAGAAATAATGTGCTGCTGTGCCCATTCTGTGTTATACATTTAGAAGCAGTTGCGGTATCATGGGATACATAATATTCTTTAATCCCAATAGGGGCTTCAATTCTAAATATAACAAAAACAGTTGGGAAAGGCACACATACACAGGTTGGCCTGTAGAGATGGAGGTGGCCAATTTGGTGTGTTTTGAACAGACGGGGATGCTCTCTGCGTACTGCCCCCACACCACAGGACAGCTGACAGGCAGCCCAAATGCCCGTGCAGACTGCTGAACTCCAGATGGCTTGCTGGTGCTGGCTGGCACGCCTTCAAGTCCTGCCTTTCTTGGGTCCCTAACAGAATTCACATTACCTGAAATTTCAGGGAATTTGTGGGGCTGGCTAAACAGATTCCTTACATAACTGGTGATGTGCGGTCAGAAAGAGAATAGATGAGTAAGATTGCATTGGCTGCCTGTGTGTATTAGTTTTCTTTTGCTGCATATTGAATTACTGCAAACTCAGTGGCTTAAAATCACACACATTTATTATGTCACAATTTCTGTGGTCAGGCGTCTGGGCATGTCTGAGCTGGATTTCCTCCTCAGTGCCACACAGAGATGCTATCAAGGTGTCGGCTGGGCAGCATGACTCTCGGGAGGCTCATGGTCCTTTTCCAAGGTCACTCAGGTATTGGCAGAATCTGGTTTATTTTGGTTGTAGGATTGAGGTCCCCTCTTTCTTGGTGGATGTCAGCAGGGGTCGATGTCAGCTCCTAGAGGTCCCCCAGGCAGCTTCTTGCCATGAAGCCATCTCAGGGACTGTCTCCCAATACGGCGACACGTATCTTCAAGTCCAGCAGGAGAATCTCTTACTTCCAGTCGGCTAATAAAATAATCTTAGATAACATAACCTAATCAAGGCAATGGCATCCCATCCTATTTCCTAGGTAATGTAATACACTCAAGGGATGACTTCTATCAACCTCATAGGTCCGGCTCAAATTCAACTTCCTGGGATTACGGGAGGGCATGGCTTATTAGGTCCTTCTGAGTCATAAATGCTCTAATGTATAAACTTCCTAGGGTTTCTATAATATATTAACACTGGGTGGTAAATGGTGTAAACTGGGTGACTTACAACAACAGAAATATATTCTCTCCCGGTTCTGGAGGCCAGAAGACCAAAATCAAGGTGTTGGCATGGTTGGTTTCTTCTGGAGCCTCCGAGGGAGAATTTGTTCCTTGTCTCTCTCCTACTTTCTGGGGGGCTGCCGGTTAACTTTTGGCTTTTCTTGGAAGCGTCACTTCAATGTCTGTCTTCATCTTTACAAGGCCTTCTTCTCTCCATATGTTTCTGGATCCTCTCCTCTTCTTAAAAGGATACTAGTCATTGGGTCTAGGGACCACTGCAAATCTGTGATGATTTTATCTCCAAAGAAATTACGTGATCACATCTGCAAAGACCCTGCAGTAGTACCTTTTTATCCATGGTTTTGCTTTCCAGGGTTTCAGTTCCCTGTGATCATTTAATCTCTAGGCTTAGTCAGTTAACTCTTGAGATATTAAGAGTTAATCTCTTGCTGTGTATAATTTATAAATTAAACTTTATCATAGACATTAATACATAGGAGACAACATAGTATCTATACAATTTGATACTAGCTGCAGTTTCAGGCCTTGAAACATATCCTCATAGATAAGGATGTGGGGTGTTATATATTTCCACATAGGAACACATTCTGAGATTCTGGTGGATGTGAATTTTTGGGACATTATTCAACACAGTACACCCCGTCAAGCTTTGCCCATGACCTGACACTGCCCAATCCTCTGGTCTCATCTTGTGGGGACTCTCCTTCACCTTTTCTGGAATATTTCCTTACAACTTCCTTTCTAACTCCTTAACTCCTAATTCAGATCATCTTGGGCTAGGAGTAATATTCAGTACTCAATCATTAGAGAAGATGGGGTCACCAGGAGATAAATAGGTAAGCAGATAGGTAGGTTGATAGATATAGATAGTTAGATAGATAGATAGATAGATAGATAGATAGATGGACAGACAGACAGACATGGGTGAATAGATGATGGAGATGGATAGATAGAAAGGTAGATAGATGATATATGTGTAAGAATAGATAGATAAATAGATAGATATGAATAGGTGGATAGATGATAGATCGATGGATAGATACATGGATAGAGATGATAGATATAGGTAGATAGAGATGGATAGATAAATGATAGAAAGGCAGATAGATACATAGATGCATAGACAGATATGGATACATGGATAGATGATAGATAGAGATGGGTAGACAGGTAGATATATGGTAGATATAAAGATGTTAGAGATGGATAGATGATAGAGATGGATAGATAGGTAGGTAAATAAGTAGATATAAATTTAGATAGAGATGAATAGACAGGTAGATAGGTAAATAGACAGACAGGTAGGTAGGTAGAGGACAGAGATGGATAGATAGACAGGTAGATGATAGATGGTAGAGATGGATAGATAGACCTCTTAATCCCTATGTATCAATCCATCTCTATAGCTATCTGTAATCACACATGTATATGTCTACATGCTCATTAATAACATTTTCACAGCAGGAATTCAGTGATTTAGTGATTATTGAATTAATTGTTGCATAAGGCTCCCTGAGGGCAACACTGGGTCTTCTTGTTCACTATCCTCAGTGCTATCATTTTACAGTGGGAGGAAGCTTACCTTCCTACCAAAAGCATTCTGTGGCTCTGAAGTGGGAGAAAGATAGATTCTCTGCCACCTTTCCCAAACCAGGGATCCTGGTTCCAACATCAGGATTTACCTGGCGCTGAAAGGATTCATTCCATTGCATTAATTGTATTCATGCACATGAGTATTTTCTGAGCATCTCTGAGGAAGGCAACAGTTTCTATGGTGAACGGTGTGGAGAGCACAGTCACTCCTCATTACAGCACTGGAAGTAATCACAATGATGATAACATACCCTGCATTCTATCCAGAGCCATTTTTAAGATTTAAAAAATTTACTTGGCATTATTTTCTTCATTTGAGTAGCTCTTTAAGGTATTTTGTGACCGCCCCCCCCCCCCATTTTATTTTTCCTTTTGTAGAGAAGGCATAATTTTACTTTCACCCTCTTAAGAGTTTTTTCTGATGGTCCTGAGAATTAAATGGACAAAGGACAGATCAGCAGGAGAAAAACATACAAACCCATGTAATTTAAGGTTTCTGTGACATGAGAAAACCCTCAGATGGAAACGAAGACTCAAAGAAGTGGCGACACTTCAGTGCTTTTAGAGAAGGTTGAACAAAGACAGACGATGATGGAAAAGTAGCTAACCTATGTGGAGGCTAAAGAAATATGTGGTTTATTTTAACATGGTCTTTTAGTACACAATTCTCTTATTTCAGCCTCCCCTTCTCAATGACAAGAATGCTTTTTCCTTCTGGTATAGGGAGGGCACAGTCCATACAGGAGTTTCATCTCTTGCTTTCAGAAAGGAAAACAGGATCAGAGCAGCTTTCTTGTACCTGCTGTTTTTTTCCTCCCCTCCCCTCCCCTCCCCTCCCCTCCCCTCCCCTCCCCTCCCCTTTCCTGGCCTGGAGCTTAAATGACCATACACCAACATAGCATTTCTGGGGTGGCAGATTCTGCCAGCCTTTCACTTTACATCCTCCTGTTATCATCTGAATTTTTGAATTATCACTCACAACTTTTGTACATGGTTTCTTAATATTTTACAAATATCTATATGCAAAAATAATGTTCATTTGGCATACCCTTATTCTTTTTTAAAATTTTATTTTATTTTATTTTATTTTAAGTTCTGGGATCCATGTGCAGGACATGCAGGTGTGTTGCATAGGTAAACGTGTGCCATGGGGGTTTGCTGCCCCTACCAATCCATCACCTAGGTATTAAGCCCCGCATCCATTAGCTATTTATGCTAATGCTCTCCCTTCCCCCCGCCCTCCCTGACAGACCCTAGTGTGTGTTGTTTCCCTCCCTGTGTCCATGTGTTCCAATTGTTCAGCTCCCACTTATACGTGAGAACATGTGGTGTTTGGTTCTCTGTTCCTGCATTAGTTTGCTGAGGATAATGGCTTCCACCTCCATCCATGTCTCTGCAAAGGACATGATCTTGTTCTGTTTTATGGCTGCATAGTATTCCATGGTGTATATGTACCACATTTGCTTTATCCAGTCAATCATTGATGGGCATTTGGGTTGATTCCATGTCTTTGCGATTGTGAATAGCGCTGCAATGAACATACACTTGCATGTCCACATTGAGAAACCATCTCACGCAAGTCAGAATGGCGATTATTAGAAAACTCATATTCTTTAATAACATCTTTGAAATGATGATTCTTCAGTCTTGAATCATCAGTGCTTCCAGGCCATACCTTCCCCATTCTTAACTTGAATCCTGACTTCATTCTTGAGCTTGTTGGAGTTGCCCTGAGCTTGATTTCTTAGAGTGAATTATCCTGTGATTTTTACTCTATGCCTAAGTTAGATGGACTTTCTTAGCATGCTAATCTCTAAAAATACCTTTTCAAAGGAGAGATTGGGAAAGGTTTTGTACCAAAACATGGTAGATCTTGTTCCATTATCAACTGCGTCTCGTGTCAGAGAGTTCTAAGGTGAGTGAAATTGTGCGTGTTTGTAGCGTGGTCATAAAGACATTTCACAGAGTGGATCGCAAACAAACCAACAGAGCACAGAGGGCTTGAGAGCAATGGCAGCTGGTGGAAGCACAGGACAGGGCACAGCGGGAATTTCATGGGACCACGAACCAAGAACAGAACCCATGACCAGGCTGTTTTTCCTTCCAGGGGCCCAGGCTTTCTCAGCTCAGCCTTCACTTGCATGCTGCTTTGAGCATGTTTGGCTTCTTTGAGAAAATGAGCCACCCAAGAGGCCTACATCCAAGTCACCTGCACTCAGATCCCAGCCAGGAGTATGGAGGGCCCATGTGGGGTGGAGTGGTGCACGTCCTCACCACCTTAGACACAGGGACCACCTACCTCATTTTAGATGGAGTGGGCAGATAATCTGCACACATACCTCCAAAGGTGTCCTCTATTGTAGAGACACCTTTTGTTTTTCTCCCTCAATCCTGGACATTTTGTTTGTTTTTCTTTATTTCACTAATTTTACAATAAACTGCCAGGATATGTCTCCATGTCTAGCTCTTTTTGTGAATTATTCTGGAAATAACAGCCTCTGCAAGGCTGCTAAAGTGACAAAGGTATTTTTCAATCGCGTCTGATTCCTTTCAGATATTTCCATCTTCCTACTCCATCATCCATCTCTTTTTAAAAATTTTGTTTTGTTTTTGAGACAAGGCCTTGCTCTGTCACCCAGATTGGAGTGCAGTAGCATGATCGTAGCTCGCTGCAGCCTTGGTCCCGGGCTTAGGTGATCCTCCCACCTCAGCGCCCCCAAGTAGCTGGGACTGCAGGTGCACACCCCACGACCAGCTAATTTTTGTGTTGTTAGTAGATACTGGGTTTTACCATGTTGCCCAGGCTGGTCTCGAACTCCGGGGCTCAAGTGATCCGCCTGCCTCAGCCTTCATGTTTTCTTTACCAGTTGGTTCCCTCTCTTTCCCACACTTGCTAAGACCACTACTGGTTCACTGTCACGATGTCACTTACTTTTTTGACTACCTTCAGTGATCTTTCTTTTCTGATTTATGTATATATTTCCTGAGTAATGTCATTCTTTATTAAAAATGTATATGTATATATGTGTACACAAAAGTATACATATATGTGTATATATCCTAAATGATTCTATTATTTATTGAAATAAATAATGTATGTATAATTATATATTTATATATAATGTAAGCATTAGTATATAATGTATATTATGGATACATTATATATACATTTTATACACAATTAGGTTCTGTGTATACTATATATGTATGTATACAGACATGTGTATATATATATGTGTTTATAATATATACAAATGATTGTAACAGTGTGTGTATATATGTGTTTATGTGTATATATAGTATATATATAACATTAATGTGATAAAAGTGTATGTGCATATATGTGTATTTGTGTTTTTGTACATACTCATGACCACATTTAAAGAATACCATTGTAAAAGCTGACCATATAATCGTCTATGCGCATATATATATGCAGCAAAAATGCCATCATCTTCATTAATAAATGCCTTCTTTATTAATAAATATACATTGGTTCACAATATCAACCTCAGCATTATATACATTTCAACAAACATGCTCATTGTTTTAAGCATACATTATTAATTCATATTTATTTTGTTTTAAGTTGAGATTGTTATAACTCCCTCTTTTTTCAAATTTTTAGCTAATGGTACTTTTTAAAAAGAATGACTTTATTGTATTCAAATTATCACTAGTGGGATAAATAATGTAATGATGGGAAAAAGCTTCCTTTGTTCCAGCTATAATTATCTGTAGTTGTTTATTTGTTTTATTCAACTTAACATTCATGTTTTATTCAAATCATCAATATATAATGATTTTGTTCTGTTACCAAAGATCTTATTGGGAATTCTAAAGTAATAAATTATTTTGAAGAGGTATCGATACTATTACACTCTTGATTTATACCTGGATCAATGAATGTTTTTAAATATGTAAGCGTTCTTTTATGTTTCTTGTTATTTTATATATTTTATGTAACATGTGCTGTACACTTCTTAGAGTTATTGCTAGAACATTTATCATGAATGTGCAAAGAATTTTTTCAAATATATTTATGTGCATATATATGACAAATCATTTTGTGTTAATTTTATACAATTCTAAATAATAAGTGACTCATTCTAAATTATTTAGCTGATTCTCTAGATTCTCTTTCTCTTGTTGGATAGTCATATGCAGGAGTGACTTTATTTTGTCTCCTTCTTTCTGATATTTTCAGTTCTCAATACTTTTTAATAAAAACATATAGGCTTCGAGTCTGTAGAAGTATCTTGAAATATGATGGTGATGATGAACATCATTGCCCTGTTTATACTTTTAGTGAAAATTCACTTAGTGCAACATTTCTTTTCCTATTTGTTGATAAGATTAAAAAGGATTTCCTGCCAAAATAAATATTCCATGTACTCTACTTTTTAAATTAAATACATTAATAGTACCAGATACTATTTGCCATCTTTCAAATAGCTTTTTTCTCCTTTGATCTTTCCCTCAGCTATCACCTGACTTCTTTCCTTCAACTGTGAATGAGACAAAGCAAAACACCCTACTTCTTCCCATTGAACCATCTTACTGTATTTGTAGAGTCAACCTAATTCCTTATTAGGTCACTGCATAGTTTTTTTTTAATTTAATATTTTACGCTATTTATTATAATGATCATTGGAGGAATAATCAGAACGTGTTAAGATTCTTTACAAGTAACTTTTACATTTTAGTGTTCTTGGCCTTTGAACTGCGTTTTGGATGAAGAACTTTTAGGATTTTCTGTGCTTGGGGGTGCTAAAGGTGTTTACACCTGAGTGAATGCCCAGAATTTGATCATATAGATTTTTCTATTGACAGTCTCACCTTCTTATGGTTATTCTCTTGTAAATTATCTTTACCTCAAGACCAAGATTTGCAAATATATTGATTTTCAGTAGATGCAGTGTTCACATAGTATCTCCTGAAACAATCACTTTTTGCAGTGTCTTTTGTATATCACTGGTTGCGTCCCTTTACTCAGATCTAAGGTACATCTGTTTCTGTATTTTTCCTTATGAGTGGTCTGGATTTTAATTCTTTCAATACACTTTATATTTTATTGGAGTATGCTTTGCCAACGCATCCTTTTTATCTCAGACTGTTCTTATGTCTCTGTAATAAAGAAACTGCATCTTATTTTACTCCATGAAAAATCACAAATGATTCCCTAAGTGTTCCTTTAGAGTGTTCCTGAGAGGACTGTGGTTGTCTTTTATTCTACATTGTGTGTCTTTTTTAAGACTTTATTAGCGCAGTTTTAGGTTCACAACAAAATAGAGGGGAACGTACAGAGAGTTCTCATATATCCCCTGCCCCCATACATGGACGGTCTTCCCTATTTTCCACATCACCCACCAGAGGGGTGTGTTTGTTACAATCCATGAACTTACACTGACATCTTCATCACCCAAAGTCCGTCCTTTACAGTAGGCTACAGTCTTGGTGGTGGTGTACATTCTGTGGGTTCAGACAAATCCGTAATAACATAAATCCACCATTACAGTATCACACAGTATAGTTCTGCAACCCTAAAAATCTTCCATAAAAAAACCTCCACAATTTTAGCAGTTTGTAACAACAAAGGCTTATTTCCTTTTTCTGAAGTTCATGTCGGTTGTGGGTGGACTTGCTTGTTACTTAGGTAGACTGATATTAGAAGGTGGGAAAAGAATAATACCTCTCCAGGAAAGGATAGGAACTATTTTGAACCAATAATACAGCTCACTACACAAAATGAGTGAACACAGTCACACTGAAAGAGAGATGAGTGACATATGCTTAAGTTATGCTTATGTTGACAAGGTCTCACTCACCTAAACTGGAGTGCAGTGCCACAATTATAGCTCACTGCAGCCTGCAATCCCTGGACTCAAGCAGTCCTCCCACCTCAGCCTCCTGAGCAGCTGGGACTACAGGCACACACCTGTGTGATTTTGTTATTTATTTATTTATTTATTTATTTATTTTTAATAGAAACAGGTTCTCATTATGTTCCTAGACTGGTCTCAAACTTCAGCGTTCAAGCAGTCCTCTTGCCTTGGCCTCTCAGAGTGCTGGAATTACAGGCATGAGCCACTGCGCCCAGCCTCCTTTAGTGTTTAACTGAACAGAATAAAGAACCTCTTCATTATGGTGAATTGGCTAAGTTCAAAAGAGTAGCAAAAGCCTTCGTGGGCAGTAATAATTACTCTATCTTCCAAATACTTGAGTGACCTTATGCTTCTTAAAATATATATTTTAGGGCTCTTAATTGAAATCAATTGCCTTTATAGCCTCTATTACAGCATACTCAGAAATTGAAGAGCGGGATGATTTTGTATAAATCTAGACTAATTTTGTTTTTCTGGAATGACTAGAACCATTTACCATGTCAGGTACACACACAAGAAACGCTAAGGGCGAGTTGTGAATGATTTGACTAGGAACAATAGTTGGGCTGCTTTTAGATGTCTCCTTTTGCTACATAGACAGCAAAAGGAGAATTCACCAAAGGTGCCAGCCCTTCAGAATCCTTGTCCCACACCACCAAAAAGTCCTGTGACAGAAATTCCACCTATTAATCAGCTGCTGTGTCCTGACTACGGAGAAAAGTATGATGCAACAGAACGCAAACTTTTCCACAATCTCATAACAAGGAAAAAATATATGTATGTATAATATGTGTACATATATAAGAAAATGTATATTACATATATAGTAAATACATACAAATACACGTATGTGTGTATGTATATATACACACATATTTTGTTTTGTTAGGTATTTTTTATGACTATTTATTTAAAAAAGTCACATTGAAAATAAAATTGACTTTTATTTGCCCTAAGTTACCTCTTGAAATATTGTGTTAAAAACCTAATAACTTCTGACAGGTATATATATACCTGTAGAGGTTAATATATATACGTGTGTTTGTGTGTGTGTGTGTGTGTGTGTGTATGCGCGTGCATAGAAGTTATTAGGTTTTGTTTGTTTGATGGTTTTGTTGTTGTTTTTTGAGATGGAATCTCACTCTGTCGTGCAGGCTAGAGTGCAGTGGCGTGATCTTGGCTCACTGCAGCCTCCGCCTCCTGGATTCTAGTGATTCTTGTGCCTCAGTCTCCCAAGTAGCTGTGATTACAGGCATGTGACACCATGTCTGGCTATTTTTTGTATTTTTAGTAAAGATGGGATTTCACCATGTTGGCCAGACTTGTCTTGAACTCCTGGCCTCAGGTGATCTGCCTGCCCTGGCCTCCCAAAGTGCCAGGATTACAGGCGTGAGCCACTGCGCCAGGCATTATTAGGTTTCTAGTACAACATTTCAAGAGTTATATGTATAGATATGTGTACGTGTGTGTGTATATATATATATATATATATATATATATATATATATATATATATAAAACCTCTATGGGTATGTTAGGTTTTTAATACAACATTTCAACAAGCATCTTAGGACAAATGAAAGTCAATTATGTTCTCAACATGACTTTTCTTAATAAACATACATTTAAAAATACCTAGCAAAATACATTATTTAGTACCTATTTTTAAACACACTGTGGTTTAATCTCAAGCTCATAGATTCTTCGAGATAATATTGTCTATCAGCTGAAAATTCTAAAAAAAAAATGGGAAAGGCTCATGTAAATATAATAGGATTTGTATTTCATTTCTGAGGACAGAAACATTTCAATAGTAAAATTTGCAACAAAAAGTGCTTATGGAAAGTTAGACAATGCTCTAGGACTCTAATAGTAAGCACAGGAATATGTCAGAGACCCATAAAATCTTTAGATTTATTTTGATTCCTACCTGTAAAAGTGTGAAATCAATTATTGCTAAATCCAGCAAAACAGCAAAGGAAAATTACTATTCACCTTTTTCTCTCAGTCTGTCTTCCAAAGCTACTAAGAGAAAAACAAGAAAAATACAGAAAATCCTACTTCCATTATTACAATGAAGCATTTTTGAGCTAGTAGAAAATTAGAATTAGACCTTGCTTTTACTGGCATCACAAAAGCATTTCATCCTGTTTTTTGAAATGACAAATGGCAGAATTCTTATATACAATATGCTAACCAAAATCATGTTATTGCCACGTCATGAATTATAATTTAATTTCTACTCTCAAAGTTAAATAAGAAGATACAATATTGCATTTCCCTGCTTGAAGAGGAGAATTAGTTACACTTGTTACGTAAAGGCTGTATTCATCACTGGTTGTCATAGCTGTTATGACTGTGACTCTTATAATAGAGGTGGGCTTGCAGCCAAAAATATATGATTCATCCAAAAGATATTTACCATGTAACTTATATTATATGTGCTGAATATTTTGGTAGTCATTGCAAATTAAGGAATATGGTGTTGAAAAATCACAGGTAACACCTTTTTCTTGTTGCTAACAATCTAACAGGGAGACCTTATTTAACAAGATATCATATTACACATTACAATTCATCTTGTGAAGAAAAATGCCAACTACAGTGAATAATTGAGGAACCCAAGTTCATTTACGAATGGAAGGTTGGGATGAACAGGGAATGCCTTTCTGAGGAAATGGAATTTAAGCTGATCAGTAAAAATGAATCTTCCAGGAGCATATGGGCTTTGCAGATGGGAGAAACAGCAGAGAATGCCCAAAAGTTCTAAAGGAAACCTGATGATGAAATGAGTTAAGCCATGTTCCTGGTAGTGTATCAGTTAGCTTTTGCTACATAAGGAACCATCTCAAAGCCGAGCATCTCAAACCACCTTTATTTAGCTAAGCATCTCAAACAACCTCTATTTAGGTTATGATTCTTGGCTGGACATCTGGGCTGTGCTCAGCTGGGAGGCTCTTCAGTCTAGAGTCAGCTTCCAGGTCTGTTGGGTGCTCATTGGCCAAGCACTATCTTAACAGGGTGCTTGACAGTGCTCCATGTGGAATATCATCCTCTAACAGGCTAGTATAGACTCTTCATGGAAGCTTGTCAGGGTTGCATGTAGGTGTGTTCAAGTCCTCTTATAATGAAAGCTAAGAATAAGGACAGTGTGTCACCCCCCACATCCGGAATGTCCAAATAAGCAAATCCAGAAAGACACAGATGAATGGGTAGTTTCCAGGGGCTGAGAGTGACCACTAAATGGTACCATATTTTTTTGGGGGGGATCATGAAAATGTTCTGCCATTAGATATTGTCAATTATTGCACAGATCCATGAATATATTAAAAACCATTGGATTGCATACTTTGACACGGTGATGTGTATGGTATATTAATTATATCTCAATTAAGCAATTATATCTGTCTATCATTTATCTGTAAACCAGATAAAATAAGACAGGCTAGGTATATAGAAAAATAGAACAGAACAAGGTAGGCAGAAACAGAATCTAGCAGATATAAAACTTGGCATGTAAGTAAAGAGCTGTAATACCTATGTAGCTGAAAATGGAACTGTTCTCTAAGGAAATAATTAAAATAATCTCTATGCTCTAGCATCCAGATAAATAAATTCCAGGTGAGTTATGACCCAGATGTGAAATAAAACCTTAAAACTGTTAGGAGAATATGTAAGCAAATAAAATGTCTTTATGTTTCTGGATTAAGTAATCCTTTTTTTTTAAAAAAAGCAGAAATTATAGAGAAAATAGTGATAAATTATAATACTTATGCATTTTAAAGCATTAGTTTAGATAATTAAAAATCAATAAAATGGTTAAAGACAACAGACTAGATATCACCAATGCTCAACTGTGTAAACTTGGGCAAATTATTTAATATCTGTATACCTAATTTTCCTCAGCTATAAAATGATATTAGTTACACATCTCATAAGGTATTTATGAAGATTGCATATTCGGAGCTGGACACAGTGGCTCACACCTGTAATACAGCACTTTGGGAGGCTGAGGTGGGAGACTTGCTTGAGGCCAAGAGTTCAAGACTAGCCTGCACAACATAGTGAGACTTTATCTCTACAAGAAATAGAACAAAATTAACCAGGTGTGGTGGTGCACACCTGTAGTCCCAGCTACTCGGGAGGCTGAGGTCGAAGAATCACTGGAGCCCTTGAGTTGGAGGCTGCGGTAAGCTACAGTTGTGTGACTGCACTCCAGCCTGTGTGACAGAGCAAGACTTTGTCTCTAAAAAACAAACAAACAAAATGCATATTCAACATGCATAAAGCCCTTAGAACCATACGCAGCACTGCTATGCACTGTTAAATGTTTGCTTTTACATGCTCAAAAAGAGGCCAGCATCCATGAATATAAAGATTTCCTACAAATCAATAACAGACATTCAGCCAGTCAAAAATTGGATTGCTATTCAAGATGGGAATTTAGAATGGGAATATAGAAATGCATCTGTACTAGTTTTAAGGAACATGCAAATTGAAATATAAACTGTTAATATTTTATACTCATCAAAGTGGCAAATGTATTGTCTGATAATGTCAAGTGTTGGCAACAGGGTAAGGGCCAGGAAATTTTCTTACCTGCTAGTGGGTGTATAGCATAATACAACTTGTTTGGAAAGAAATATGCCAGTATCTACTGAAGATAAAATTAGTATTACCCTATGTATCAGTTAGCTACTGCTGCATAACAAAGGACTCTAAAAGTCAATGCCTTAAGACAATAAGCGCCTATTACTGCTTATGAGCCTCTGCATCTTGTTAGCTGGAAATTTATTTTGGTCTTGGCTGGGCTCATTCATGTGTATGCATTGTTGATTTGGAGTGAGTTCTCTTAGGTAATTGGGGGTTGCTGGAGGTAATTTTGCCTAGGTTAGGGCCAATGGGTTCTTCTCTATGAGATCTTTTGTTGTGCAACCTGCTAGTCTGATTTTTCACAGGACAGTGGCAGAATTTCAAGAGAGTAAGAATAGGTACAGGGGATTTGAGTCCCAGTCTTGGAAACAGCACATCATTATATTTTTTCTTTTGAAAAAATGCAATCTTAAAGCCACTCAAGATTCAAGGGGTGAAAGTACAGACTCTCTATGTATGAGGAATAGTAAATTCATGGGGAGGATTGTAGAACTGGGAACCTTTTGCCTGTCAGTGGACTACACCCTGTAATTCAACAATTGTCTATCTAGTAGTTATGTGCCCTGGAACTGGGGTCTTCAAACTGGCAGATGTCTTTTCAAAATTTTTCAAAGTATGACTCTGCTGATGATTTTAAAGAAACTAATTTTCAGGTACTCAGCCCCCAGATGTTCTCCTTTCTAAGCCTTCCTGGTCACCAAAAGCTTCTTCCCACATCACAAAAGGATGACCTTCAGTAGGCATGACACTTTGTTACCAACCTTTTCTGCCAGGGTTTATAATACAAGAAATATCTTTTTGAATGCTGCTTTCTGGAAAGCCCCTTTGCTGAAGGCTCCATAAAATAAGCCTCCTATCTTATACATATTTCCATTAAGAGTGAAGTTTGGTCCTGTTCAGGTGTTCTGATTTCAGAAAAAGAAAAAAGAAGCCATAGGTCAGCTATGGCAGTTCTTTCAAATGCAGAAACTGAACTTTTCTGTTGCTAACCAATTTTTCAAGGTGCATATACATTGGGTGAAGCCCATCGGTAAATGATCCAATCCGAAAATCATCTGAAGGTCATCTTTCAAATTCATTGTGGTAGTGTTATTCAAGTGGAGGCTCAAATATATTTCAAGTGTATGCATGGAATATTTTCCCCAGCTAGAGTCTGTTCTCCAGGTGTATGGAGGAAAGAGGAGTTGTCCAGGTTGTGTACCTGTTCTTCTCATCTTTCTGGGGCTATTCATGTCCTTTCTGTGCCCTCAGCCTCCAACCCATGCTTCTGCTCAGAGCAGCCTGTTTTCTTTGCTCCCATAAATGTATTCCTGGCCCCAGATCTTCTGTGCATATTTAGAAGCCCTAACCCACTTCCTCACCAGCCACCCCTCTATCCCCAGACTCTCCTACCAGGAACAGCAGAGGATCCTAAATTCATGCATGCATTTTCCTGCCCCGTTGGAATGATCTGTGTGCATGTCTGTCTCTGATGTTCATCTCCTTCTTCAGTGTGGGTGTGTCATTACCTCTTTTAGCCAGGACTGCATGGCATTACCTGTCTTAGTCGGGACTGCATGTTAAAAGGGTCAACACATATTTGTAGAAGGAATTGGCTTCTGAGTGAATGAACCCATGTGTCATGGGCAGTCTGTGAGGACATACCAGTCACTTCCTTGCTGCCGAGAGCTGGGGATATTGCATTGGATTAGAAGATTAAGCCCATATTACTCTATGGCCAAGTGACAAAATAATCAATCACATCCACATCTGTGATAGCCAGGAAAACATTTCTTTCCGTGCCCCTCCCCCACCCCCCGCCGTATGCAACTTTCCCTGTGTGGAAATAATGTACTTAGCTTAAAAAGTCTCTTTCTCTACTTAACAAGACTAAGTTGAAAATTAACCTTGCCCACTTAAAAGAAAACGAATATGCAGTAAACTATGAACTACTAATACAGTTCAATATGATATCTCATGCAGAACAATAATGCTGAAGGTTCTTTTTGGTTCTATTATTTCCTTATATTCTTGCTTAGATAAGATCACATTTGTATCTATTGACTTTCTATGATGATTTAGATACATAAGTGGCAATAATTAATATATATTAAAAATACAGATTTAAATTGTTTTTCTGACTTGTAATGTTAACAGCAGTATATGTGACTGTGAGGTTTTCCTTTGATGTTAATTTTCACTTTGACAATAGTCTTCGTTTTCCAATTTTTTTTAATTTTTTTATTTTTATTTTTATTTTTTTTTGTGATAAGGTCTGGCTGTTTCACCCAGGCTGGAGTGCAGCAGGGCGATCTCAGCTCACTGCAACCTCCACCTCCCAGGCTCAAGTGATCCTGCCACCTCAGTCTCCCGAATAGCTGGGACTACAGGCATGCACCACCATGTCTGGCTAATTTTTTGTAATTTTCATACAGAAGAGGTTTCACCATGTTGGTCAGTCTGTTCCAGAACTCCTGACCTCCGCCCACCTCGACGTCCCAAAGTGTTGGGATTACAGGCATGAGCCACCGCGCCCATATCATTTTCCAAATTCTTTACAAAGTTTTTCTCTTACATTCATAACATAAAGTGCTATTTTAAATAGACTAACTTTTGAAAATAACATAGATAAAGCACTAAATGGGGACATCAGAGGAACAGGCTAAAAAAAAGCTGGAATATTCTTCAGGATTAGGGACATTGAGATTTTATTTATAAAATGATATTTAAATTTTAATAATAGAATTGTTGTACTTTTGCTTGGAGTATTTAAATCTTCTCTTTAATATTTAAAGCCAGTTCTGCACAGAGGTTTTACGGAGATGCTAATTGTTGTATGAAAAGGAATATTATTCTGGAATTTTGAGGAAGGGTAGACATAGAGAAGATAAAGGAAACTCACAGCCTACCTAGGTTTTATTTGGGCTGTGTGTGTGTGTGTGTGTGTGTGTGTGTGTGTGCGCCAGCCACAAGCTGGGTTTATTCTTGAATAAACTGTAGACAAATTGTTTTTCCTGAATCTTCTAAAACCTGCATTTACATAGTCCATGGTTGTGTCTAAACTAGATACTCAAGAGAACTTGGTTTGTTTTAAAGGCATTTAATTAGTTATATTTACATGGACAAATAGAGCAGCAGTTTATTAAAAAAGAATGAAAGGATAAACAAATTAAATATACGTAGAACAGGAAAGACAGCATCTAATTATGTTTCTGGGTCAGGCTCTGATATACAAGATTAATTTAAAATTGGGATTTGGCAAGTAATTTCTATCGAAATCTCAGCAGGAGTTTTTATTGCAACTAACAAGCTGATTTGGAAAGTTTCATGGAAAGGCAAAGGATCTAGAGCAATCAAAAAGACCTTGGAAAAGGGGAAGAAAGTTGGAGGGCTTCCATTTCTCTATTTTAAAAGGTACTATAAAGATATAGTAATCAAGATAGCAGGCAACTCACATGGGTATAAATTTAGACCAATGAAATATAATTAATTACAGTTGGCCCTTGAACAACGTGAAGGTTAGAACCCCTGCACAGTCGAAAATTCACTTAAAACTTTTTACCCCCCCAACACTTAACAACCAATAGTCTACTGTTGACTGGAAGCCTTACCAATAACATAAACAGCTAATTATCACATCTTTTGTATGTTATATATACAATGCACTGTATTCTCACAATAAACTAAGTTAGAGAAAAGAAAATACCATTAAGAAAATCATAAGGAAGAGAACATATATTTACCACTCATTAAATAGAAGTGGATCTTCTTAAAGATCTTCATCCTCATCTTCAGGTTGAATAGGCTGAGGAGGACGAGGGAGAGGAGAGGTTGGTCTTGCAGTCTCAGGGGTGGCAGAGGCAGAAGAAAATCCACATATAAGTGGATCTGCACAGTTCAGAACTGTGTTGTTCAAGCGTCAATTATAAGGGTTTAGAAATAAATCCTTCAATTTGTAGTCAATAGATTTTTAACAATGGTGCCAAAACAATTAAAGGAGGCAAGGATAGTCTTTTCAATAAATGGTGCTGAGACAATTGGATATTCATATGTAAAAAGATCAATTTCAACTCTTACCTCTTATTGTACCCAAAAATTAACTCGAACGACAGGTGGCAATATAAGAATTAAAGCTCTTAAACTTTTAGGAAACTTCAGCAACACAGGAGAAGGTCTTCAGGGCCATGGATTGGGAAAGATTTCATAAATATGACCTCAAAAGTACAATCCTTAAAAGAATTGATCAAGTGAAACTCATCAAAATTAAAAACTTTTACACTTCAAAAGGCACTATTGAGAACATAAAGTGCTATTTGTTGAGAAAACCAAAAGACAAGCCATAAACTGGGAGAGGAGATTTGCCAACCATATTCCCAATAAAAGACTTTTATTTAGAAAATATGTAAACAAACCACTTACTATTCAATAATAAGAAGGAAAGAAATTATTTTTTAATGGGCAAAAATAAATTAATAGACATTTCTGCAAAGACAGTGTACATGAGAAGATATTTAATATCATTAGTTACTAAACATTAGCTAAATGCAAATGAAAACTACAATGAGGCCAGGTGCAGTGGCTCATGCTTGTAATCCCAGCACTTTAGGAGGCCAAGATGAGTGGATCGCTTGAGGCAGGAGTTCAAGACCAACCTGGCCAACAGGGCAAGACCCATGTCTACTAAAAATACAAAAATTAAACAGGAATAGTGGTGCATGCCTGTAGTCCCAGCCACTTGAGAGGCTGAGGCACGAGAATTGCTTAAACCCAGGAGGTGGAGGTTGTCGTGAGCCGAGATCGTACCACTGCACTCTAGCCTGGGCAACAGAGCAAGACTTTGAAAAAAAAAAAAAAAAAACCTATGATGAGACACCATTTCACATCCATTAGTATGGTTATAACAAAAAAGGATATTAGCAAGTGTTGGCTAGGTATTAGAGAAATAGAGACCCTTTATACCACCGTTGGTGAGAATGCCAGGTATTGCAGCTGATTTGGAAAATAGTCTGTCAGTTTATTAAAACATTAAGCATAAATTTGCCTTATGAAACAGCAATTTCACCCCTAGGTATCTATGCAATAGAGATGAAAACATATATCCATGCAAAAAATAGTACACAAATGTTCATAGCAGCTTTATTAATAATAATCAACAAGTAGAAATAAACCAAATGTCACTCAACAAATAAATGGATTTAAAAGATGTGGTATACCCATACAATGGAAAATAATTTAGCCATAAAAAGGAATGAAGTATTGATGCATGCTACAGTATGAAAGGACATTGAAAACATATGCTAAGTAAAAGAAACCAGACACAAAATACCGCATATTATATGAGTTCATTTATATGAAATGCCTAGAGAAGGCAAATCTTATAAAGACAGAAAGTGGATCAGCAAGGCTATCACACCCACGCACCACCCAGGTCTGGTTTTAAAAGGTATTAAGCCCCCATGAAATGGACATTACTTGACTTTTGTTTGATATATGGAAACAGCATTATCAAGTCTTGGTTTCAAAATATGTTTAAGCTCTTCTGAGTTATGTAGAACAGAGGAGTGTTTTCCATTCACAAGTGTTGGAGATGACAGTATTTTCCCTTTGCCTTAATCCGCTTATCCTAGAACCCTATAGGAAGGCAAAGACTGTCTTGATTGATTGACGCAGTTAAAGTTATTGATAGTGGGATATGCACATATGGGCTGCATCTGTCTATGAGAAGGAAGCAATGGAGCCAATTAATTAATTCAAGCAAAATTAAATGTTCACACCTTTTAAATGTGGAAACTATAAAAACCAAAATGGTGCTCTGTGCACTAAGAGCATAAGCTAGTTTTTTGCTATCCTTAAGGGCCTCTTCCTGCATTTTGCCTATATTAAAATTCCTATGCAGATCTTATTGAGGTGATCAAGGTAGATGACTTCGATTTTTATTTTCTTCAACAAATTCACGTACCAATAACTTTCAAATGATATTTAGTAACTATTTTAAACACAGAGGACATGATCTTCAAACGATATTTAATAGCTATTTTACACACAGAGGGCATAACTTTCAAATGATATTTAATAACTATTTTAAACACATAGGACATGGTCTATAATGTTTTGTCCTGACTTAAATATTTATTGCATGTAGTAGATTTTAATAGAAGAAAACAAGAGTGAATAGTGGGTAGTGCTTCTCTAAACACAGAGTAGAGGTAAATCTTAGTGATTTAAATTAGTCACAATTCTGACTTTTTGAGATTGCATGTTTATAAGTTTTTAATGCATGAAATTAATGTCAATTATATAATATTTTGAATAAAGTCCTTCCATGTTTACTGTGTTTTTGCTTGCCTTATGAAAATTTCTAACCATAATGTGTCAGTAACATTTCAAAAATTTATTTAAATTACAACATGTTAACATCAGAGGACCATTGAATACGCCATAAGCATTTCTTTAAAGAATGTGGGAAATGTCTTTTCTAATAATTTAATTTTTTCTTTTTTTAAAACAACTCACGTTAGCATTTTTTTTTTTGCAGTAGCATCATTTTAACCCCCAACTGCATATTCACAGGATATCTAATATTTTTTGCAAGTAACATTTTGAATTTGTTCTTCTTGACATCTTTATGTTTATATGCATTTTGCATTTCCCTATCTCATTTTTTTGAAATCCAAATGTAACAAATTTCAACTTTTTGTGTTACATTCTTTTCTTTTTTTCTTTTTCTGGGTAGCATCTCTCTCTTTTCTGAATTTTTTGAAAACCTGTTGTTTTTGAATTCTCTTTTTTCCCTTTATTTTCCTTCTCAATATGACCCCAGGAGCCAACACAAAGAAAAACGCAGATGATATAACGAGTAATGACCGTGGTGAAGACGAAGGTATTTTTTGTTTTTTCAAAGCTCAACCCCAGTGCATGATTTTATATCTATCTATCTCTCTTTTTTTTTTTCATTTCAATCTGTTTTTTCTCCCCTTATTTAAAACTAGTACACTTTGGTGTGCTTCCTTAATTATTTTCTTCTTGTATAGAAACCACTGTCATTTTTTAATCCCAGTTACCATGTACAGGAAACAAATCACTGTGAGAAGTATAAACATTGTTTCTAAACATGAAAAGAGTAATGAACTACTGTTTACAGAGAAGCCCTTTTTTTTTTTTTTTTGGCTTGGTCGCAAGAAGAGAAAATGGAATTTTAAAACATGCATGTATAGTCTATTTTCTCCCTTCCAAATGTTATTTTGTAAGTTAATATACTACTTTGGAGCTTTGGTCTTCTTAATTATTTTTATGAACTACAAAACTGTACAGCACCTTAGAAGAATTTTTTTTGGGGGGGGGGGGGCTGAAATATCAGTTTTTTTTTTCTTCACAAACATATTGATTCCAACATAGATTTCTGATAATCTGCTCACAGTGAAGTACACCAAAAAGTGTTTTAATGAGATGCTGTTGTTAACGAGCCCTGATGCATTCAGGACTGCCTTTTACAGCATTTAAGGGGGGGTGGGGAAGATAAGAGTATCTCAGAACTGAAAAAGGACAAAAAGCTAGCTATGTTCATCTTTCTTTTCACACCACGGCTTTTTTGAAAACGTTTTTCTCCTTAAAATGTTTTGTTGCTGTGAAGTTTCTTCTTAAGGCTACCAAATTGCTCAACACATTGTCTACCAGAAGTGAAAGGATTTTTTTTTAAAAGATGGTAGGTCTGAGGTACTCATGCAGACAACTCGCATGCTGTTTTTCTGCCCTTTCTGCACAAGAAATGATTTTTTTTTTTTTTAAAGAGGAGAAGCAACAAAAAAAGTACTCAAGCAAGCCCTTCTTCATTGGTAAGGCTCTATAGGATTAGCTAAAAGCACATTTTTCCCATCTGGGTAGCAAAATGCATGGAACTCCATTAAGGTCCTGGCTGGACCTTTGGGTCTCTGTCTGAAAGGCAATTTAAAGCCCAAAAGTGAGTCCTGAATTATCCTTGCTGGTCAAGCCCAACGTCCATGACAGGGTCTTTTGACCAATTCTTGTAGTTGCTCCCCTCCTTGCTTATCTTCATAAATCAACTGTTCTCCAAGAAAAGAAATCTTGCCAACACCCTTGCTGTGCCCAGTCTTCCCTTAACATTTTGAGTATTGTTACTTTTACTGAGCTCATAGAGCTGTCACTGTCTCAAGTAGCTCTCTGAGAGATCTCCATTCTGATGGCCATAGGAGATCAAAATCTACACCTGCTTCAGGTAGCCCCTTCTTTGATAAGGGCTTCTGAATGCCTGACATTTTATCAGTATTGAGCAAATACATAAAAATGAAATAAACTTTTGTCTCATATCTTATACTGCTCTAATTTGTATCCTGTTTGGCCTTCTCTTTTTAATACATTTCCTCTCGATAATTAGAATCTGTTTTCACAGTGTTCCCAGTGAATCTTTATTACCATTAAAATGCCATCTAATTTTCATTTCATATTGTTAAGTTATGATTTTTTGACTTTGCATTAATATAACAGCTGGTTATTACTTCCACAAGTTCAAGAGAGTCTTGTTCTATATTTTATGAAAGGTAAGAGATGTTAATCTCACATATTTTCCAAGGGAGCACTTTAAAGCAGCCCTTCAAAATCTCTACTTACTCTTTTTTCCACAATTTACTAGGCAACCGCTGGTAATGGTAAAAGAAATGAGGCCAAAAACAGCAAATTAGGAACCAGAAAGAAGCAGTGGATCATGAGAAAAGCCATTTCTTATTCATATAGCAGAAGACATTTCCCGTAGTGTATGATGAATAAATGATTAATAGAAGATTTTTACTTCATATTTGAATTTTATATGAGAAAACAAAAGACACTTTTCTGCCGTGGATTAAATATCTGCAAATAAATACTTGGGTAACTTGACACTCTTTTGTGTGCTTTACTGTGACCAATGGGTATGTCGTGTCTTCTGTATGCACCCAGTAAAATTGTGATCATAATTCATTCAAATTGGAGCCACCATCCAAACGATGGTAATTCATATCCTCAGAATTCCTTTGTGGTATTTCAAAAGTGTCCCTGTGGATTATGAGGAAAAAAAAACTTTATTGATGAAGAAATTGAAAATAAATATGCATAAATACTTGAGTTTTCTTTTAGTTACAAAGATATTTAAATTGTACACACACACACACACACACACACACACATATCTGTATCCAGAAATATTTATACGTGAGGTCAGTCTTCCAAAGATTAAATGCAGCCCTAATGGCTGATTAATGTTATAAAACAGGTCTTTTTCACAAAGCAGGCCCTACAGATGGTCTCCAACTTTCTATCATCACAGATCATTGTTTTTACATCATTGTTAATTTAAATAATAAAGTAAATTACCAAGAGGAATCATTGGTTGCAAGTCACAATGGGAGTTTATATTCCCTGTGAAAATATAAAGCATTTAAATAGTTTGGATTCTTTTGCCATTTTTTATTACATCTCTTTTATTTTTGTCACCTAAGTATGTTAGTATGTTACTGTAATCACTGGAACAAAGACATTTGCTTGGACATCTTTTCTTTTTTTTCCCTATTTCTGTTCAGTTAATAATTTTTAACTGTTGATTTTGCTTTCTTGTCATTATCTGTCCCTTATTGATAGTTTATAGCTTCACTACTACTTTTATGTTTTTATTGTTAAATTGAAGATGAATCTGTACACTCACCTGCGAATTAAGATGCAACTATATTAAAATTAATTATAATTTTGAAGTTGATTTTATACTTAATTAGAAGATAAAATATATTTCATCAAGGGTCCCATGTGTTTATTCAATTTAAATCACATTTTAGGGTTTGAGCAAAATTTAGGAAATGTGTACTTTACCTAAAACCATTTCTTTTAGTGCTTTAGATATATATAGAAGCTTAGATGAGCAGAGTACGCTAAATGTCTGTATGCTTCTTAAAATACCATTTCCATAAATAGAAAACGTAATAGCATTGATCATTTTCCTTAGACACTCTTATCAAGGGTCATATCATCCATAAAAATAAATGTGCTTAATTCAAGTCAAAATAGGGAAATCAGTGAATCTCCTTTTTTCTTAATTTAGCATTGGTGAGTCAGTGTGATTCTTTATTGTGTTTCCTTACTTGGCTTTTTTTTCCAGATATTCATGATCAGAACAGTAAGAAGCCCGTCATGGTCTATATCCATGGGGGATCTTACATGGAGGGCACCGGCAACATGATTGACGGCAGCATTTTGGCAAGCTACGGAAACGTCATCGTGATCACCATTAACTACCGTCTGGGAATACTAGGTAAGTGATTTCATCATGTGAATGACTGAGCAAGAGGAAACATGAAAAGTCCACTTCTCGTTTTGACGGGGCTCGTGGATTTGAATCCTGTTATTCCAGTTCCTGGTTAATTCCACTTCACGGTATTTACTTTATGTGATTGGATATGTTTATTCCTTTTACTACCTTTGTGCAACATGGTCATGAATCCCTTCTCAAACCAATGCAGACTTTAAGATCTTAAAGATGAAATGAAATTTTATTTATAGCATGTTTCTCCCTTGGAGTTCAATGAATGTATGTTTGTCTACATAGACCTGTACAATGAACACATATTTGGTGATATTATAGTTGGGAATGGCCATAGATCTTAGCTTTCTTTTCTGATTGTGTCATTGTATGAATCAGTATATTGTGTGGAGGAAAAGATTTTATCCAATTCTCTAACTGATTATGTTGAGCCTTTGGAAGATCTGTTGTTTTGGTTCCATTGCATTTGCATGCAGGGAAACTTAGCTGTTAGTTGACTTTTGTCCATTGATGATCTACGATTAAAGGCTAAATACATGGAAATTCAAGTTTAGTTCCTCCTTGTTTTGATGTTTCATTTCTTTTCTTTCTTTCTTTTTTTTTTTTTTCTTTGAGATGGAATCTCACTCTGTCGCCCAGGCTGGAGTGCAGTGGTGCGATCTTGGCTCACTACAACCTCTGCCTCCCGGGTTCAAGTGATTCTTCTGCCTCAGCCTCCCAAGTAGCTGGGACTACAGGCGCATGCCACCACACTCAGCTAATTTTTGTGTTTTTAATAGAGACAGGGTTTCACCATATTGACCAGGCTGGTCTCGAACTCCTGACCTCGTGATCCGCCTGCCTCGGCCTTCCAAAGTGCTGGGATTACAGGTGTGAGCCACTACGCCCGGCCATCATTCATCTTCTTCTAATTGTAGGTTGGAAAATTATACATCTTCAGAGTCAGATTTCAGTACCTTCTGAGATGGCCTTTCCTGGTGTTGGTTAGTTTGTGAATAATATTCCTAAGACCTATGTAAAAACATTTGTTTTCCAGGCAAAAATGCATTAAAATGGTATAGAAGATAAAGTTTTTAACAAGTTAGCCATGAGAGAGATGTGTATATTGGTTCCAGTGTGATTATGATACAATATGAAATACAAAACAAAATGAAGGCCAGGTGTGGTGGCTCTCGCCTATAATCCCAGCACTTTGGGAGGCCCAGGCAGGCAGATCACTTGAGGTCAGGAATTAGAAAACAGCCTGACCAAAGTGGTGAAACCCTGTCTCTACTAAAAATACAAAAATTAACTGGGCCTGATGGCAGGCGCCTGTAATCCCAGCTACTCAGGAGGCTGAGGCGGGAGAATCTCTGGAACCCAGTAGGTCGAGGTTGCAATGAGCAGAGATAGCGCCATTGCACTCCAGCCTGGGTGACCGAGTGAGACTTTTCTCAAAAAAAAAAAAATAATAATAATACTAGTAATAAATTAATTAAAATAAAAAGCAAAATAAGATGGACTAAAGGAGGTCTGTCAAACAAGAAATATGACTGAAAATGTTTTCTTCAAATATGGCCAAGAATATTTTCTTTTCAATCAGATGACTTCATTTCATTTTGAGTGGGTTTTTTTTTTTCCTATGTGAAAACATTAACCTGTAAGAAGCCCTAAAAGGTGGTGAATTGCTGAGAAACCCTAAGAGGTGTTGTAAGAAACCCTAAGAGAAATGCATTTCTTACTTTGAAATGCAAATCAGTCACAGGTGTTGCTAAAGTTGTATCTTTTGAAACATTGATAAAGAACTCAAAATTCCAGGTTGGTTTCTGCATTAAAGAAAATAAACACCACCAAAAAACCTTTTAGTGTCAAAAAACTTATTATGTCGTTGGCTTTATTTCCTATATTTTTTGTAGTTTTCTGTGAGCCACATCTTGGCGGAATAATGTCTCTGAACTTTTGCATAGCAGTAATTGCACGCTTCACTGAATAGTTTTCAGAGGCGCTGGATAGTTGCTTTGGCTACTAGTGTTGGAAACAGGAAATTGTGCTTCTTGATGTTTTACAAAAGGTTCATTCTGACAAAGAGGTGGAAGGAGGAAAGTATGTGTGAGGGCATTGCACAGGCCCTCTTCAAAGGGAGCAGTGTGTGCACTGCCTGTAGCACGGCCACACGGAAGAAAGCTTGGGCATGCTTTTCTGAGGGAAGCAGTGGGCATCAAGAAAATTCTTGCTTTGCTGGAACCACACAATATTCTGTTGCATGCGTGATGAATTGATGTGTCTGATAAGATAGAGTTTCAAAATAAATTGATCTCCTTTTCCCCCTAAAGCTCAGTTGTATCAAGCAACTCTACACTATGATTTTTTTTTTATCAGTTTTGTCCCTTCGTGAATCAATTGCACATCTTGCAAATTAGCCTGGAAAGTATACACACTTTTTTTAGAGGAAAAAAAAACTAATTGAAAAATTGTTAAGTCTACTTTTTGTTATGGAGAGTTTTTAAAAGTCATAAGATAACAGAGAGCTGTAAAATTGGTGGGGAAGAAATAAAAGAAGCGATTTAGCATCTCTATGCCGGTCTATTTACATTCCTCCAATGAGCTAGTGTGGAACAGCCAAGCACACTACAGACCCCCTTTCATTTGATGGAATGAAATGTGCCAAGTTTGCCGATTTTACAGGACGATAGAGACTTTAAAATGTGACTGCGTTGGTTTTTATCATGGATCTTGCATTTACTATTGTCCTCTTGAAAACAGCTAGGCGGCATTTACTTTTTGCTTGCAGGAAACTCCTATTATCGGTCTTGAAAAAATGTTTTTAAACCTTTGGCATCCAGATATTTAAAAAGATGATCAAATAAAATACACAGCAGGCACTGCAATGATCATTTCAGTGAGTGCATTTCATACAAGTAGATACAATTTTAGGCAAAAAGTTGAAATATTCTTTGAGTTCTTTTTCTTCCAGTAAAAGTCATAAATGCATAAATGTTATCTTCCTACCTGAGGAATGGAAAAATATTGTTTTAAGATTTTTTTTTTTTAATGGAGTAACAAATGCTATTCTCTGTTACCCAAAAGAGAGGATTAAAAAGATGAAACATGCCCATAATGGAAGCGGAATGCTGGCATTGGAAAGAATGTAGATCGCAGCCAGAGACAGACAGGAGCTAACAACTTTCCTCTACCTCTGCCTTGAGAAAGTCAGCTAGCGTTTCCTCAGACTCTTTCCTTAGATGTAGAAGGCAGTGGTCTCTCCCTTGCAAGGTTGTTGTACAGTATAAAAGTTCCATGGTTCAAAATACCACACTTTACCTCATTAATATATAATCTGCTTGTCAATAAAAAAATAACTTTTTTCTTTTCTTTTTTTTTTTTTTTGAGATGGAGTCTCGCTTTTATTGCCCAGGCTGGAGGGCAGTGGCATGATCTCGGCTCACTGCAACCTCTGCCTCCCGGGTCCAAGCGCTTCTCCTGCCTCAGCCTCCGCAGTAGCTGGGATTACAGGCGCCTGCCACCACGCCCCGCTAATTTTTGTATTTTTAGTAGAGACGGGGTTTTGCCATTTTGGCCAGGCTGGTCTCAAACTCCTGACCTCAGGAGATCCACCTGCCTTGGCCTCCCAAAGTGCTGGGATTATCAGCATGAGCTACTGTGCCTGGCCAAAAAATAACCTTTTAAAAAAGATTTAATGGACTCATGTAGATGAAGTTTCATAGGCTCTCAGCAGCAACCATTATACCCAGTCACACTACAATTTCTAGTGTTATTAATACCATTATGCATTGTATTAATACTACTGTTTATCCACAGTAAGAATTGTAGCTGACCCAACCTGTAATGGCTAACTAATATCTATCAAATATTGGCATCCAGACTGAACCATGTTAATTTAAAATAACATTACAAGACACTTGTAGACATTAAATAAATCAGAAGATCATCATGTTTGCTATTTTTTAAAAAATAATCAGAACTGTGCTACACAATCTTGCTAGCCATTGGCCATATAATTTATGATCCAATCCAGGACATGTTTGAGAGTTGCTCATGTGCTATGAATAAACTGGGATTGTCCCAGGCAAATTGAGATGTATCATTATAGCTATAAAGTAATTATTTATATCTACATGAAGTGTCTTCTGATTGAATTGGTGTTCAGTTTGTTTTTAAAGAAGCTGCACTTCTATAAACAGATTTCCTATGTGTTCTGCTATACACCCTTGTCACTAGGAAGGTGTATATGTTACCAGAAAGGGATCCTAATCCAGACCCTAAGAGAGGGTTCTTGATTCTCGTGCAAGAAGGAATTGGAGGCAAATCCGTAAAGTGAAAGTAAGTTTATTAGGAAAGTAAAGGAATAAAGAATGACTGCTCCATAAGCAGAGCAGCCCGAGGGCTGCTAGTTGGCTATTTTTATGATTATTTCTTGATTATATGCTAAGCAAGGGGTTGGTTATTCATGAGATTTCCGGGAAAGGGGTGGCAATTATTGGAACTAAGGGTTCCTCCCCTTTTTAGACCATATAGGGTAACTTCCTGACATTGTCATGGCATTTGTAAGCTGTCATGGTGCTTGTGGAAGGGTCTTTTAGCATGCTAATGCATTGTAATTAGTGTATAATTAGCGTATAATGAGTAGTGAGGATGACCAGACATCACTCTAGTTGCCATCTTGGTTTTGGTGGGTTTCGGCTGTTTTTTTTTACTGCATCCTTTTATCAGCAAGGTCTTTGTGGCCTGTATCTTGTGCTGACCTCCTGTCTCATCCTGTGGCTAAGAATGCCTAACTTCTTGGGAATGCAGCCCAGTAGGTCCCAGCCTTACGTTACCCAGCCCTTATTCAAGATGGAGGTGCTCTGGTTCAAACGTCTCTGACATATATATTCAAGAATTTGGAAAACCTCAAGTTCACCAATGCCTCTCAGATTAGTCATTGCCAGGGTGTGTGGTGTTCCTATCTGCTCAGAAGCCAGAAGCCAGCAAAATCCTTGCTGAGCTGTACGTGCCAGGGCATTTGCCTGGTCTCACCTACCCACTTGAGTACCTATGCCCTATCACCCATTCACCTCACAACATCCATACGTATCATTTACCCCTAAGAAGATTAGACATTAATCCAGGTAATAAACTTTCAGAACAATCACCTCCAGACAGAAACTGCAGAGGATAATCTGATAAATCTGAATCCCTGTAAGGCCATTACTGAATCAATAAATACTCTTTTCTCCATCTTAGTTCCTTACTTTAGTATAACTTGAGTTCTCCCCAATCTGTTTTTTTTTTGTTGTTGTTGTTCATGATAGTCCAAAGACCTTCGATGTAAAAGAGAATGCATCTTGCTCATGCTTTTTGATGGAAATACCTGGAACTTATTTATTCCTTCCCCTTTCCAGTTGTCTCCAAGTGCAAGTCTGTCTGTACCTGCAGTGGATTTCATCTACCTCCATTTAAATATGTATTTCCGTTTAGCTCACATGGTACTATCACCTTTTTGGTGATCCTATGACTTCATGCTTCATGTATGCTGAAATTAATTGTTGCTTCAAAAGAGTCCCAACTATGTAACATCAACTCATTGTGTGCCTCTATGTGGCTGGCAGATATTACTTCATTTAATCTTCGTAAACTCCCTTGGAAGAGTTAACCTTATGTCCTACCTATGAGGAGATGAATGCTTTGAGGTAATGGGATTTACTCATGGCATCACACCTTCTAGCAGTCAGAGCAGGGACTGAAACCCGGGTGTAACTGAAGCCAGAGCTCTGACTTACCACTCAGAACTCATCCACAGCCTTCTTAATTAATGTCAAGTATGAATTAGTAAACCATGGAATGAGTGAAGAAATTGAGTATCACTTTAGCATCAGATGTAGCTTTTATCATTATGCAAAAAAGTTCTTACTGCTGATCAAGATACACAATTGTGATAAGATGCTTACAGTGTATTTTTAAGTTCCTCAAAGTGGGTCCTTGAAGGCTGATTCATTTCCATTCAATCGATACTGGTTTGCTTTGGTTCACGGTGATGGTGGCATTAACCACAACAATGGCATTTGTCACATCAAAGCTCTTCGGTGCAGTAGAACTAGTGTTTCATCAGGAAATTTGGTGTCCTACCCCCAGTTCCCATGTCATTGCTGGCTTGCTGTGTCGTGTGCATAAATTGAGTCAAATGATCATTTCGGTGCATTTCTTACAATCTTTCACATATTATAGCTATCCTGAAAATTTTCATCTGAGGGTAGATTGCGTCATGGTCTTCTGAAGTTGTCTTTCTCTTTAAGACCATTCATTGAATAAACCTATTAGACGCTTTGGAGTCATAATTGAATATAAGACAGAAATGGTTTGATATAAAAGCAACCAACATGCATAGCAGAAACAGCATTTGTAGTCATAATTTGGGTGACTTAACCCATATGCACGTGCTCAGCCTAATAATGTGGTCACTTTCCCTGTTCTGGTGTCCCTTGTAGGGTTTTCCTCTGAAATTGAGGGAGGGTGGGCTGAGCTCTGAAGCATTCTTGCAACATCGGCCAGAGTGGTCTCACCTTTATGCTTTTGTGATATGTGTGAGCCATGTAATATTCCACTCAACAAAAGAAGCCTGGAAATCATTAGAAGAGAGGACCAATACGTTCTTCCCAAGAGTTACAGCCTCAATTCCATGGGTGTGCATTTATGTGACATGCATCTGACATTAGTGGGAGTTCAATGGGTCACTATAATTTCCCTGAAGCACACCTGCTGAAAAATGTCAAGCTATCTTATAAATGACCTGTATGTTCTTCTCCCCTTTGGAAGTTAGAGGAGTTGCTCTATTTTTGGTACATTTGCTATTTTATTTCTTTTTTTCTAACAATATTTCTTTTCTTTAATGCTTTATGAAGGATTTTATTTGAAATGATAAATGGAACACATCTTATGTATCAAGTCAAAAGTTCATAAGCGTATATATTAAAAAAGAAAGCATCATTTCCTTTTTCGAGAATCAACACACCTTGATGCCAGTCTCCTGGTTTCATTAGAATCCCTCTCTTCTCTTCCTCTAACCAAAATGTCTCAGATTCCCCCGATTTGATTTCTGTAAATGGCCTACTTTGACTGGAAGAATTGCCTCTCTCTGTCTAAAACAGGACCCAGGCGTTACTAAAACAAAACACTGCAAAAAGTTAAATGAGGAGAAAGGAAAGTTAAGCATTGTACTTAGTGAGAAATACATAAACAAAAGTAGAGACGTAAAAGAAGCATGAGAGAAGGGTGAGAAAGTGAAATCCTGAGACAAGATGAATGGTGTGTGAGCACTCAAACCCAGGAAGTAGCAAAAGGTGGAAGGAAGAATGGGAGCCTTTAGAATAAGATTCTTTGTGGGCTGGGTGGCAGATGTTATCGGTAAAGCCAGCCTGGGGAGTTGGCAGGGGTCCATGCAGTAGATAACACAGCAATAGAGTGAACACATTGCAGAAGATAGGGCAACCTCTAATCCAGAAATTATCAGATAAAGAAAAACCAAGACACTTTGCAAAACAAAAAAAAAAACAAACAAAAAAACACAACACAATGTCTTGTTTTTCATCATCATCTTCTTTATAATGAGGTTTCCATGCATTGAATACACACTTGGAAACACTGTAATCCCATGGTTGTTGTGGCTGCAGATTGATAGGTGTGGACAGGTCTTTGGTGGGGCAAACAAAACCAGGATCATGTTTTTTGCTCTCAGAATGATCGTTTGCTTGGACTTTCCTCTTCTGCCTCCTAGTGGCTCAAAATGCCCACTGCATTCATTGGATTTATTCAGGATGTGAAGAAGGTCAGGGGAAATTAAGGATGAGTGCTTTGTCATTAGGACCTGAGAGGCAAATGGAGCAGAGATGGGGACGACTGCAGTGGGATAAGGACTCTCTCACCAGGAAGGTGCCATTGATGTAATAGTTGATGGGAACAGCAGAGCAAAGAGGCTCCCTCGTCCTCAGCTGACTCAACAACAAGCGAGACATCAGATGGAACGGTATTTATTGGGCAAGGAAAATCAGGGGAAGGCTAGGTGCAGTGGCTCTCACCTGTAATCCCAGCACTGTGGGAGGCCAAGGTGGGAGGATTCCTTGAGGCCAGGAGTTCCAGATCAGCCTGGACAACCTAGTGAGACCCTGTCTCAGAAAGAAAGAAAGAAAGAGAGAGGGAGAGGGGGAGAGAGAGAGAGAGGGAGGGAGGGAGGGAGGGAGGGAGGGAGGGAGAGAGAGAGAGAGAGAGAGAGAGAGAGAGAGAGAGAGAGAGAGAAAAGAAGGAAGGAAAAAGAAAAAATTAGCCAGATGTGGTGATGTATGCCTGGTGTCTCAGCTACTTGAAAAGCTGAGGCAGGAGGATTGCTTGAGCCTAGGAGTTCGAGGCTGCAGTGTGCTGTGATTGCACTCCAGTCTCAGCAACAGAGTGAAATCCTGTCTCAAATTTTTAAAAAAGACTCAAAAGAAAATCAAGGGAGGGAGTGGAGACAAGGTAGAAAAGAATTTTTTTTATTTTGTGCTTTTTTCCCTAATGTATTCATTTAATCATCAAATAAAAATTGAATATATTGATCATGTACAAAGTGATGTTTTGAAATATGTATCCATTGAGAAATGGCTAAATCGAGCTAATTCACAAGTGCATTACTTCAAATGCTTATTTTTCTGGTGCAAACACTTAAAATCTACTTTCTTAGAGATGTTCAAATATTCAATTCCTTGTGATTCAACTTTGTTTGCCATATTGAACAGATCTTTTGAACTTTTTCCTGCCAACTGAAACTTTGTAACCTTTGGCCAACATCTCCCGTTTCCTCTCCACCTCCAGCTTCAAGTTCTGTAAGAGAACATTCTACTCTCTGCTTCTGTAAGCTTGACTTTTTTTTAGATTCCACATATAAGTAAGAACATGTGATATTTGTCTTTCTGTGTCTGGCTTGTATCACTTAACATAATGTCCTCTGGTTCATCCATGTAGTCCCAAATGACACAACTTCTTTCCTTTTTTTTGAGGTAGAATAATAGTCCCTTGTGTGTATAAACCCCATTTTCTTTATTCATTCATCTAATGATGGACATTCAGGTTGATTCCATATTTCAGCTGTTGTGATTAGTGCTGCAATGAACATGGGAGTGCAGATTTCTCTTCAAAGACTTCTTTTTTCCAATCCCAAATACACAAAATTATCATCTGGCATCTGTCATGCTATGGAGACTCTCCTTGATCTATTTATAAACGATTCAGGATTTCTTTAAAGAAGCTGAAATTTTATTTTTACATGCATAACCATATTTAGAAATCAAAATATTCAAACAGAAATCACAGAAGAATCTATTCCATCAATATATAATTCCCAGTTAATTGATTATATAATGTCATTTAAGCATGAGTTAGTAGTCACAGAGAATATGCCTTAAAAATGTTCTGTCTTTGAAAGTTTTACATTCAAAACAGTCTCTTAAGATTATTAATTCTAAAAGACACCATCCCTTTCTCTCTTCAGCCTGTTTTCTTCATTTTGCTTCTCATCCAGTATGTGAAAGGTTGATGATTTTTAGTTGATGAGGTTGACGTGCCCTCTTTCTCCTTGGGGACAGAAGGACATAAGTTGTGCTTTAAATGAAAATAAGAGTATGATGAGTATCCCAAGGGATGATGGAAAGTTCCAGGGAGAAGCATTGAAATTGAGAGCCAAATTCAAGTACATTGGAATTAGGGTTCTGGTGATAATTCTGTCAGTATCTACATATATTCAAGGAAATTAGTCCTTTCGAGTAGGATAATGGAAAAATCTCTAAAAGGCAATCTGAGCGGGATGTTTAAAGACTACGTGATTATTATGCAGTGCATGCCTGTACCAAAACATCTCAAGTACCCCACAAATGTATACACTTACTATGTACCCATAAAGTTTAAAAAAATGTAAGACTACTACACATATTCTGGCCTGCAGCTTTTTTTCCCCTGACATTTGCCTACCCGCCTGTAATAGCACAGGCAATTCTACAAGAAGCATGAATATGCACATATGTACATGCATGACAGCAGTGATACAAAGACAGATGTGTTGTGTTCTAGTATAATTGTCTTATTTTTGTCCATTCCAACGTTAATAAGTCATTAGCTTTATGGAAATGAACCCTAGGGGATGAAACATACAGGTGCAAAGTAAATTTCCTAGGGACTAAATTATAACCAAATTATGGCAGGTACACCCTGCATTTAGCGATATAAATATATGTTTCAAATAAAATTGTAACATATTGATTGGCACGTCCAGCCATATTCTTAAGATACTTTATCCTTGGACTAAAAATAATAATAATCGCTTTTTTGAATGAAGTGTTTAATTTTCAGTGTAAAAAGTCAGGAATATTTTAGAATGCTCAACGCAACATTGCTTCAATGAGCTAGGGCCTTTATGAAGATAAGTCACTAGAAAGTCTGTGTTGATTCGGTTAATTATTTGAGATTGTATGCACTGATTTTCACTGTGTTAAGTATAGTGGCATTTATTAGAGGCTCAGATGTTATAGAGAGAAGGCTGTGTCCAGTTATAGGGCTGTAGTCATAAACAGATGGGTAAAATCAACACATCATTGTAAATCATAAACAGGCAGGTATGATAAACACATAATGATAAGCATTTCAGCACTGGGTGCAGTGTTGCATGCCTGTAGTCTCAGCTACTCGCGAGGCTGACGATCTTTGGAGCTTAGGAGTTCAAGAGCAGCCTGGGCAACATAGTGAGAACCCATCTTTAAACATTAAAAAGAACAACAAAAAAACATCATTTCAGTGTAGACAGGCATAACATGATCTCACAGAGAAACACTACGATTTGTACACAAGAAAACTAAGCTTTGCACTGGTGTTGGGAGAACATTTTGGAATGATAAACTATTTCCTGTTTGTTTTAAGAAATATTTGGTAAGGTTTAAAGTAGTGTCTGCCTCTTTACTAAAATATTCCAGTATCTGTTTAGATGTCCCAGTTGGTCTTAGATACTTGGTGGTAAACATATATATACACATATATAGCGCATATATGTGTATATATGTGGGTGTGGGTGCATATGGGTGTGTATAATCTATGTGTGTATACATACATATATGTGTACATACATACATATGTGTGTATACATATACATGTATCAGTTGTTTGCCCTTGTGATGCACACACAGATCTATATGTGTGTATATATATGTGTCTATATATGTATACATGCTAATGTGTATGTATACATATATAAAATATGTTCCTTGATTCACAGTGGGATTATATCCCAATAAACCCGTTGTAAATGTAAGATGTCATTAGTTGAAAATGCATCAATACATCTAACCTACCAAACATCATAGCTTAGCTTGGCTGACATTGAACATACTTATAACACTTACATTAGCCTACAGTTGGGTGACATCATCTAACACAAATCCTATTTTATAAATAAAGTGTTGAATGTTTCATGTACACTGCAGAGTAGCAGTTGTTTGCCCTTGTGATTGTGTGGCTGACTGGGAGCTACAGACCGCTGCCTGGCATCCAAAGAGACTATGGTACTGCATATTGCTAGCTTGGGAATATATCAAAATTCAAAATATGATTTCTACTGACTGAATATCATTTTTGTATCATCTTAAGATCAAAAATCATAAATCAAACCATTGTAAGTCCGGGAATGTCTGTGTAATAATTTGGCTATAGTCTTAAACAGGTGGGTAGAATAAACACATTATTATAAATCCATCCTGTGCTTTTGAACACATGGAGGCTACCCCACCAAAATGCCTGTGTTCAATATATTGCGAACCTCTAGGTATCTTTTTCCTTCATTGCTGTTTAATTTTTCCTTCTAAGCATGAACTTACAAGATTACTTAGGAATAGCATTCATCCTTCTTCATTCCTCTTTGTTTAAAACATGCTTAGCATTTCTCATCTTGAAAGAAATGAGTAGCTTTCTTCTTTTCAATCATATTTCATCAGAACTATTCTCTTGAGGGCCACAGAAATGTCATAAGCATTTTCTCTGGCACTTCTGATACTTTTAATGGCTTTTGATACATCTTCATGTTTCTTAATCTTCTTGTGATCCTTACCATGTAAGTGACCCGTTGAGCTTATCTCCAACTCCTATTTTTCATTGTCTCCTTCCTTTATTTGAAACAACTTACATCCAGCGTGCACGTTTGAAGTGTGCAATTCAATGGCCTTTAGTATATGCACAACATTGTGACACCAGCAACACCATCTAATTTTTGAACATTGACGTCATTCCAAAGAGAAATCCCATACCTCTTCTCTCCCAGGTCCCCAGGAGATAGGCTTCCACTAACTATCTACCTGTCTATATAGATTTGCCTTTTGGGGGCATTTCATGTAAATTAAATCATATAATACATGCTTTTTTGTGTGTCTGACTTCATTCCCTTAATGTTTTTGAGGCTCATCCATGTTGTAGCATGCATCTCTACTCTTTTATTTTTTATGGTTCGGTAATATTTCATTTTATGGATATACCACACTTTGTTTATCCATCCATCTGTTGCTAGACATTGGGATCATTTCCAGTTTCTGGCTGTTCTCAATAATTGTGCCATGAACGTTCATGTGCAAGTTTTTGTATGGACATATATTTCATTTTTCTTGATTGGGGATATAGGAGCCGAATCGATAGGTCATATCATGAACTCTGTGTTTAAATATTTGAGAATCTTTCAAATTATTTTCCAAAATAGGTGTACCATTTTACATTTTCACCATCAATGCACAAAAGTTTTAACTTCTCCACATCCTCACTCACACTTGTTCTCATCTGTCTTTTTAATTATAGCCATCCTAATGGGTGTAAAGTGATATCATGTTTGGGGGTTTATTTTTGAATATTTACATCATTCCAAAAAGAAGTCCCGTATCTCTTCTCTCCTACATCCCCAAAAAGTAGGCAAGAGGTAATCTACTCAAGAAATGATACCAGCTTAAACCAGGGCAGTACCAGTGAGAATGCAAAGAAAATAAAAAAGAAGAGGTTGTTCTGCGTGTCTTACAGATGCAACAGGATTTGCTGATGGATTGGATGCAAGGTGGCAGAGAATGAGAATGCATTTTTCCTGATGACTAATGATGTTGAACACCTATTCATGTGCTTATTGGACATGTGTGTAAATCCTTTGGAAAAATATCTATTCAGATCCTTTGCCTATTTTAATTGGATTATCTTTTCATTACTGAGGTTTAGGAGGGGTACTTTTAAGTAGTATAATGTGGATACATGTTCCTTACCACATGTGGGATTCACAAACACTCCCATTCTGTGTCTTCCACCTCCACTTTCTTGATGGCACATTCTTATTACTCATGTTTCTGAAAACATAATCTTCAGCCTCATTGACCAATGACTCTGAATATTGACTCATATATGTTTAAGCAGGCTTGTCCACTTACTATATCTCACAAGTCCCATGGTTATCGTGACAGTCCACTGCTATCCCGTCCCTTGTGGCTGTCTCATCATTGTATGGAGACAATATAAGGATGCCGGGACAGATAAAGGGTATTAGGATAGAGTGCCATCAATGTGTCTGTGAAGAAGGGTTCGTTTCAATCAGTTCACCATGACTGGGGATTTGATTCTGTCAATTGCTGACTCAGGAATGTAAATGCTGAGTAAGGCAGGACTTGATCAGTCTATTGGGGGAAGGCATCATTGACCAAAGTGCAGTGCAAATTTATTCATTGACTATGAGGCATATAACTCTTTATAACTGTCAATAGAAAATGGACAAGGCATCCCTCCGTTCCTTACAAGGTTTTGTAATGAGCCCTGGATTTAAAAAAATACTAGTAATAATAAGAGAAAGAGAGGGAGACAGAGAGAGAGAGAGTGAGATAGAGTTTCTAGTTTAAGTGAAGTTAAAATGTTTTTTCTATATATACAAAACTAGCTTTGCCAAGGAAGATGTAGTAGTGGTTTTCATTCATTCATTCTTCTTTCATTCAAGAAACAGATATTGACAACCTGCTGTTTGACACATGGTATAACAACTTCCATTGAAAATGGAGTAGCAAACAAAACAGAGAAAAAATCCCCAATCCTACAGCATTTCTATCCAGTAGGGGAAAAAACAACGACAGACAAGTATCGTAAAATACACAGTAGAATATGATATCACAAGTGCTATGGAGAAATATTTAGTAGAGAAGGGTGCTAAATTAGAAATTTTGTGCCAAAATTTTGACTAAGGTGGTTATGGAAAGTTTCACAGATAAGGCAAAACTGATGTGAGGGAGTGATCCATACAGTTACCTGGAGGAACAGCATCTTGGGCTAAGGAAAGATCCAGTGCAAAGGCCCTGTGGCCACAGAGTCCCTGAGAATATCAGTGCAGCTGGAAAGTAGTGGTGAAGGGGATAGTAGCACCTGATTTCAGAGATGTCAGCATGAGCCACATTTTATATGCCTTTAAAGGACTAGTGTATTGTTCTTAGTGAGAAAGGAAATGGCTGTCTATGTAAAGGGGCATTAGGTTAGAAGGTTGTTGCATAATCCACCCAAGAAATAAAAGGCATTTCGATCAGAATTTAGCTCTTCTACTCCATGAAACTACTTATCAGTTCCATTAATGCCTTCCACTCTGCACTCTCAGGGTTCGATTTTCTGGAAAATTTTGAATTTTGATTTTGATTTTCCAGAACATTTAGAGTTCTCGATGACTCTCTCCTTCACGAAAAACATTCCTTACTTGGTATCTATATTTGTTTCTTTCCTATTGCTGCTAAAACAAGGTATCACAACTTGTTATAACTCTAATGTTAACTCTAGGGAATTAAAAGCAATGCAGATTTATTATCTCACAGTTCTGGGTGCTAAAAGTCCCAAATGTGTTCACATTCAAAGAGAGAATCCATTTCCTTGGTTTGTCTGTTTGTCTTCTTTTGAAGACTGGCTACATATCTTAGATCTCATTCTCTGTTTCTAACCTTCCATTTTAAAAAACAAACAAACAAAAAACATTATGATTACCTAGATTCATCCAGATGAACCGGGTTAAGTTCTCATCTTAAGATCCTCACTTTTTTTTTTTTTTCTCTCTCTGAGATGGAGTCTTGCTCTGTTGCCAGGCTGGAGTGCAATGGCGCGATCTCAGCTCACTGCAACCTCCCCCTCCCGGGTTCAAGTGATTCCCTTGCCTCAGCCTCCCGAGTAGCTGGGACTACAGGCCCGCACCACCATGCCTGGCTAATTTTTTTGTATTTTACTAGAGACGGGTTTTCACCATGTTGGCCAGGATGGTGTTGATCTCCTGACCTCGTGATCCGCTCTCCTTGGCCTCTTAAAGTGCTGGGATTACAGGCGTGAGTCACCGTGCCTGGCCAGGATGTTCACTTTTTAAAATTGATTTATTCTTATTTTATTTTAGAGATGAGGTTTTGCTCTCTCAGATAGGTTGGAGTGCAGTGTCATAATCATAGCTCACTGAAGTCCCAGCCTCTTGGGTCAATTGATCCTCCTATCTCACCCTCCTGAGAAGCTGGGACTACAGACATGCACCACCACGCCCAGCTAAGTTTTATATTTGTTTACAGAGGGGGTTTCACCATGTTGCCCAGGCTGGTCGTGAACCCCTAGGCTCAAGTGATCCACCGGCCTCAGCCTCCCAAAATGCTGGGATTATAGGTGTGCTTCCTGACACCAGTTTCTGAGGTCCTTGACGGCTGTGGTCATAGCTCATACTACCTCTCTCTCCCTAGTGTCTACCGGACAATAAGCAGTTTCTGAATGATTAGCCGTTGCAGGGTTTTTGACTCCAAATTGCAAAATGCAAGCTAATTAAAAAAGGAGTGAATCTATTTACTCATTTTTTTTTTTTTTTAGTTTGAGTGAACTGATTCTCAAAATCAGTGAATGCCCAGTTTCATGTAAACCGTGTTTATTTCCACTGTTTACACTCAGCAGCTGTTTCTTTTTCACAAACACTGGAGATTCCATGTTCCCCGAAATATCTATGTATACCTGTATCATAATTCATTACACATAGGTTAGCTGGAATGGAGATATTTTATATTTGTGGCATGCATTTGATCTTGAATTGAAACCTGTAGTTTAGAAAAATCTACATATCTTTATATTTTTAACAGATTTTGAGAATTATAAAAGCAAAACAGTAGAGCTCTACGGTAGAATTTTTTTTTCTTTAGGTCTTTCCATGGGTATTTTAAATGTCTCATTATGAAAAGACCATAAACCATGGTTTTCTAAGAGTTCTGCTGAATTTTGCAATTGGCTGGCACATTTTCTAAATGATCCTGTAATCTCCATGTATTAGTTTTCTAGAGCGGCCATAACAAATGACCACAAATGTGATGGCTTTAAAAGAGAGAAATTTACTCTTTCTCATAGTTTGGGAAACCAGATGTTCAAAATAAACGTGTTGGCAGGGCTGCCTTTCCCTGGGTGGTTCCAGAAAAAGATCCTTCCTTGCCTTTTCAGCTCTGGTGGCCTCGGTGTTTGTCTCTATCTTCCCAAGGCTGTCTTCCCTCTATTGTATGTGTCGTCTCCTTTTCTTATAAAGATACCAGTCATTGGATTTAGGGTTATACCCTCAATTCAGGATAATTTTATCTGCAGATCCTTAACTAATTATATCTGCAAAGACCCTATTTTCAAATAGGGTCACATTCTGAGTTTCCAGGTGGACATGTATTTTTGGAGGATATTACGCAACCCACTCCACCCAACACATCATTATTGCAATATATATGTATGAATATAGGTGTTTCAGATATTTACACTACACATGTGTGTACAACCAATGTATTCAGGATGCCACCTGGCTTTCTCCTTACTAGGCCACACTCTGGCAAGAAGATCTAAGGACAATCTGGGATTCTTCATCTCCTTCTTGCATCCTCTTTGCTTCCAAATAATGTAGTCATGCAGTATCTGAAAGTTTATTTCCTGAGCCTTTAAAACTTCTCCATCAGTTTGACAAGGAGTAAAAGCGTTTTTCCCCGTTGGCCACAAAACTTGTGCTTTTGCTCCAGCAATACGCAAAGCTATATTTCACACTTCCTTCTTAAATTACAGGCTATAAATATAAAGCAAAACCTTTTACCTTGGATATTCTTTCTGTCTTTTCCCTCTGTGATTAAATCTGATTACAAATGCTCATTAATGCTCTGCCTTGGAATTGCAATTTGGGCATGTGCCATGTGAAAATGGAGGTTCCTAAAAATTAAAATCAAAGATTAATGCAGGTTTTAAAAAAGGGTCTTATTCAAATATATCTCAAGTTTTAAAACGACTCATGGACTTTTAATGAAATCAATGGCCTTGTAATGCCTCATTTTTTTTTTCAAACTCAACTGTTTCATAGCCTTCTCTTTAGAACATATCTGATTTACCAGAACCCAAGATTTGTGAGATGGTGTTATTTTTTATCTTTACTTTTTCCTCACCCCACGGTACCATGAAGAGATCGTGTAACATCCTTTCCTGGTTTTAAAGACAGGTGAGTAACGATTACATAACGTTCAAACAAGTCAGGTGTTCTCCAGAAGATGGTGTTAATGGTGTCTGATTCACAGATGCTGCCTTGACCCCTGGCGGTGGTAGGACCTATATTCTGGTGAAAGCCAATTTTAGGCCATGGATTATAGGACCTAGATGGAGAAAAACGATACCTAAACCTCATGAGATCTTAATTCACTGATCGGTGGAGAGATATTTTTCTTTCAGATGGTATCATCTTATTGCATCTCCAGCAGAGTGTTTGGCCGGTGAAAATAAAAATGGCCATTATAAAGAAGTTCTTTAGACTTTTAAAAATTTTACTAGGATCATGCCAGAAATTCCTGCTGTAGAAGTAGATATGTATGTGTGTATACATATATATATATATATATATATTTCTGAATTTGAGATGTTGGGTATTGGTAGAGATTCATTCATTTGAATGGAAATACGCTTGCTTTACTTTTGGCCAGCATGAATGCTCTCATTTGCCACAGGTTGGCAAGCTTATTGGTTTAAATATAAAGGATCTTGTGGGTAAGACTAACAGCAGGTTTTCATAGTGCCAACATTTCTTTCTTTTTTATTATCATATTTAGGAAAGTCTCTTGACTCTGAGATACTTTATATTGTGAAATAATAGTTCTGGTGCAAGTATAGATTAATAGATTATTAAACACTTTAAGATATGGATGGAAGAGTACAACTAGGATATTATTAATGAGTCCCATTTACTATTCTTTAATTTGCAGTGGAATTTTCATTTAACTTTTGAATATACCAATGATAGGAAGTTAGTAGTGTTTGCCTGTAATTTATCCTGAGCTCATTTATTTGAAGTTCAAATTTGAAAGCTTCCTTTTGTTGTTTGGTAAATAGAGATTATTGTGATTCAAAATGAGTAATCCCTAAATTGATGTAGAAAAAGATATTTGAGGCTGGGCACAGTGACTCACGCCTGTTATCCCAGCACGTTGGGAGGCTATGGGAGGTGGATCACTTGACCAGGAGTTTGAGACCAGCCTGGCCAACATGGCAATACCCCGTCTCTACTATGAATACAAAAATTAGCTGGGCATGGTCTCACAAACATGTAATCCCAGCTACTTGGGAGGCTGAGACCCAAGAATCGCTGGAGCCTGGGAGGCGGAGGTTGTAATGAGCTGAGATTGTACCACTGCACTCCACCCTGGGCGACAGAGCAAGACTTCGTCTAAAATAATAATAATAATAATAATAATAATAATAAAATAAAAAGAACTTTGAGATATTCATATTGTCCAAAAAGTATAATTCAAATACTTAATGCAGAAGGCAGTAGGATCACTAAACTACAGACTCATTCATCAATTATAACAGATGGAAGGGTCTTTGTTAGAGTCCTGGAGGCTGATTGAGCATTTTAAATGGCAGGTTCATAGGGGAGATCCAGGAGGTCTAAAGGTGAGGGTCTACAAGCAGGAAGCACCCCCACTCCCACCCCCAAATTCATGACAACAACACTAACTAGGCAGCAAAGGGATATTTCCTGATGTCAGCAGTCAGCAGAATGGTACTGAAGGTTGCTAGATAAATGCAAGTTTTGTAGTCACTCACCTGCAAGTTATAGGCAAGATATTTATCTGTACTCCTACAGGAAATTAGCCCTAATTGACTGCTCTTAATCAGAACAAGACATTCTAACCTCTTATTCATGGTTAGCAGTATATCCCACTTGCTTCACTTTGTGATTCTCCATCACATTGGAATAACTGGACGTGGGATACATTTGGAATTGAGTCTCAAATTCAAATCGCCATAGAACCTGAAAAGAAAATGTAAGAAGAGACAAAACAGAAGAAAAATGCAGGATAGAGAGTTATGATTTAGATGTGTTCATTCTGTGAACAGAGAGCAGATTCTCTTGGATCTGGCTGAAACAGGGGCCCCCTGTGTTGTGAAAGTGGTGTATGTCTTCATACGTGTTCCCACGGGCCTGGACAACCAACCACATTTGAAAAATGAAGAAATGAAAGCTTGTGGTCAGGGTCACAAAACTTGACAGTGGCAGAAGTGGATCCAATTTCCAGTCAAATCTATGACTCGTTCCATCTTGGCCACAATTATACTGCAACTCAATTGCTTTTCTTCCAGTCAGTACCCACCCACCGAAATGTCAGCTCTTCAAGGGCATTAATTGTTGTTTGTTTCATTCATTGTTGAGTCTTAGGAGCCTGGGACAGTACATTGAAAATCTCAATTGTTGACATTCTCAATAATACACAAGAAATCATGTTTTCAGATCATGGAAATCATATCCATTAGGATGGCTGTTAATAAAGTAAACGTAAAATAAGAAGTTGTAATGGAGATGTGGAGAAACTGGAACTCTTTCACATTGCTGGTGGGAATGTAAGATGGTACAGTCATTGTGGAAAACTCTTTGGCTGTTCCTCAAAAAAGTAAACATGGAACTACCATATGTGATCCAACAATTCTACCTCCGGGTATATACTCCAATTCTACCTCTGGGTATATACTCAAAAGAATTGAAAGCAGGAATTCCAGGAGATATTTGTATACGCAGTCCTTAACCATGTTATTCACAATAGCTAAAAACTGAACTTTTGAACTAGCCAACTATCCATTGATGGATGAATGGATAAACAAGTGATATATATGTATATATTTATGCGTGTACACACACACACACACACTGCTGAAATGGAATATTATTCAGCCCTTAAAAGAAAGGAAATTCTGATACATGCTACAACATAAATAAACCTTGAGGACATCATTCTAAGAGAAATAAGCTACATGCTAGTCACAAAAGGACAAAAGCTGTATGATTTTACCAATATGAGGTACGTAGAGTTGTCAAATTCACAGAGGCAAAAAGTTGAATGGTGTTTGTGTGCGGCTGAGAGGCGGAGAGAATGGAAAATTATTTCCTAATGGATAGAGTTTCAGTTTGGAAAGGTACAAAATGTTCTGAAGATAGATGGTGGGGACAGTTGGACAATAATGTGACTGTTCTTAAGGCCACTCAATTATACACCAAAAAATAGTTTAAATGATCAATTTCATATTCTCTATATCACAGTAAAATAAAACATTATGGTATCTGTGATTTAATTGACTATTTGTAATCATCACCATGTTAGAGCATGTTCAGTATCTCATATCCTGCAATATTGGAATGGACATGGTAATTTTTGAGTGGTAGAAAATAAAGTAACTTTTAAAAACCCATCTCTATGTATTCACATAATCTTACATTTCATATAAGTGAAATCATACACTCTATATCTCATTTCTTTCTCCTAATAAAATGTTTACAAGGTTTACAAGGTTCATCCACATTGTAGCATGTATCAATCAGTACCGCATGCTGGTTTATGGCTGGATACTATTCCATTGTATGATAGACCGCATTCTGTTATGTTTATCTATTTTTCATTTGATGGATATTTGGATTCAATTCATAGAGACAGAAAGTAGATTAGTGGTTGCTGGTGCTTGGAAGAGGACTATAGGGAATTAGCGTGTCATGGTTACAGAGTTTCAGTTTGCGAACATGAAAAATTTCTAGAGATAGATTCACAAAAATGCAAATATACTAAATGACATTGAACAGAACAGTACACTTTAAAATGGTTCACTTTATGTTACGTGAATTTCCTCTTAAATAGAAGAAAAATAAAGTCTGAAGTTGTCATATCCTTCACTGGGATGCTCTCTTTAAAAGTGTAGAAAGGTCCTGAAAGGAGCATATAAACAAACTAAACAACAATCAAACAAAACATGTCATCGTACCCCACAGCATCCTGACATGGAAGACTAAAAACTGTCCCAGGGCTCTCTTCTTCCTTATCTGTTACTTTCAGGGGCATTTTAGCTTAGGATTTAATTTGACTATTGACAACCCCAGTGTCTCCATTTGATCTCAGAGCAAACTTGAATTGATAATTAAATTTCCATGCTTTTGACCAGGGAAAGACTTTAGGAAATGTCTTTGAAACTGTGAACTTGCAGAAAGGAGAAAATTTTATATGTATCTAGCTTCTATCCATTCCATTTGTCATATGGTCAGAACTTACATGATGCAAGCAGGCCATTTACAGGGCCCTGGGCTGACAGCTACATGCTATATTTTGTATTTGCTTCCACTATTTTGTTAGCAAATGTATGTACTTACTAACAAAATACGTGTTTTAAGAAATAAAATTATTTTAAGAACAAAATAATACAATGTTTTAAGAAAACCTGCTTTTATTTGCTTTTTATTTTTTATTTAAAAATGTTTATAAATTTATGGGTGTTACAAATTCAGTTTTGTTATATGGGTATATTCATAGTGGTGATGTCGGGGCTTTTAGTGTACTCATCACCCGAATAGTGGAACCTTTATCCAGTAGGTAGTATTTCATCCTTCATGCCCCTTCCTCCTCCTTCCACCTCCTGACACTTTATAGTCTCCAGTGTCTATTATTCTACCCTGTATGTTAATGTGCACCTGTTGTTTAGCTCCCACTTATAAGTAAAAACATGCAGTGTTGGACTTTCTGAGTTATTTCACTTAGGATAATGGCCTCCACCCAGTTTCATACATGTTGCTGCAAAAGACATAATTTCATTCTTTTTTATGACTACTACTGAGTTGTATTCCATGGATATATAAACCATGGTATATATAAACATTTATATATCCAGTCATCTGTTGATGGACACTTAAGTTGATTTCATGACTTTGCTGTTGTGAATAGTGTAGTGATAAACATATGAGTGGAGGTGTCTTTTTGATAGAACCATTTCTTTTCCTTTGAGTAGAAACCCACAAGTGGGATTGCTGGGCCAAATGATACTTCTATCTTAAGTCATTTGGGAAATCTCCATACTATTTTCCATAGAGGTTGTATTAATTTACCTTCCCACCAACAGTGTATAACTGTACCCTTTTCTCAGCATCTTTGCCAACATGTGCTGCTTTTTGACGTTTTTCAAAATGTCATTCATTTTCATTTTTATTATAATTACTTAAAAATGATGACTTTTAACAGAGAAGGGAAAAATAAAGTTGGTAATCTTTTGTAGTGCCATATAATTTCTAGTTACAAGACCACAGATAAGTCCCATGCTGAAGAGAGGTGGGTAAAATAGCTCGTTTGAAATGAAGCACATTTGGGAAGATAAAATTGTTTTTAGGATGATAACGATGTTTGATGTCTAACTTTGGTCTAGTTTTTCTAATGTTAAGTGTATTCTTAACATCTGCCCAAATTATTCACTCTTTAAACCACATGCCAAAACATTACTTACATTTACTTGGTTTATAATAAAATTTGGGACTATTAGTGGATGATATTTACTGCAAGAATTGTTAATCTGGCGTTTGGATCTAGTATTTAGATTACTTTATATTTTCAGCTGCATATGCAACTATTAGATATCTGCCCACACTTTTTCCTTCCCACTGTGGAAAATACACACTGTATTAAGGTGACAGGTTTTCCTATTTTCACCCCTTAGACTTGAGTTATTTTCTCATCATTATTAACTCATAGAACCTGTGCTTTGTTCCTGGCTTCAGCTTGAGCACTGTGCAAAAATTTATCTTATAAGATTTGGTCAAAACTGTTGGCTGTGTAGGCACTTCCCCTAGTAGAAACTTCCCCTTTCCCCTCTGAGGGTTCACTGAAAAATCAACTTAAAAAGGCAGATTAATTGAAGAAAAGGCATGCAAATTTCCTTTAATGTGGATAGCTTGGCAGGAAGGATTAGGAGACTGATTACCCAATATCTTAATGGAGTAGATATGCTTATATACTCTACTTCCTAGAGGAAAGGGAGGTGAGGACTCCTGGATGATACTTAGGGGGATAGTAAATGATTTTTAGGGGAATTAAGTGGGCTTGAAGAACATACAGTGGCTTAGAACAAAGTCTGTTGGGCTTGCAGAGCAGACAGTGGTTTGTCACAAAAGTCTGTCCAGGTGTGTTGACAGACTTCATTCTTTCTTCCTGCGATATGAGTCCAGTTACTAGAATCTCGGGGAAGGGACCAGAGGTCATTGTTTTCTTCTTTGATGGGTCCAGACTTTAGGCAGATAAACAACTTCAGAAAACAACTTCCTCCTGTGCTTTGGGGGTCACAGAGGGTTGAGAGACAAGAGGGAGTGGGAGAAGATGAGAGAGACGTTGAGGCTTCTTCTTCAGTTCAGCACATCAAAGTGCCATATTTTGCTGTATGGGTTTATGAGTCCCAACAACTGGGTAGTGAAGACAACCCAGGGCTGTGTGTTGATGGTTCCGCTGCAGACAGTCAAGGCTCACTTCTCTGGGAGGAAGCTAAATGCCACTCAGAGACACATCCCCATCTCAGATGTCTTTGTTATATTGATGACAGTTGGCACCCAGATGGCATGTATCCCTTGTGGTTTCAACCATTGGTTGACATGACCTTAAAGGCCCAAGGTATGTATTCGTTGGTCCATTTTTTGGAGGAATGCCATTTTACTTCCACAATGCAGCATAGCTGTTAACCATTCATCATGCCAGTAAGAGAATCCCCGGGATCTGCATTGGGACAGAATCCCCATTCACTGCCTTGTCTCACTTTTGTAGTTTGTTTTGTTTTGTTTGAATTTGTTTTAGTTTTTAACAAATAATCTGAAGGTAAAATACAATTGAAAGAAGCACTTATCTTATGATATCAGGATAAGTAAACTAGTGCAGTTTCAGAAACATCTAACCAAGTGTTGTTTTCTTGCTGGATTGCAATATTGATAGGCACATGGGATAATATCTCATGTAAATTCTGAAACATCTAATTGCATCTTGATCCTTCATCTTGACCCTCTTCTCAGTGGGCTGCATTTATCCCTAAACAGCAACATTCTGTCAATTCTTAGGAACGTGAAACGTTACAGTCTGCAGAGCAAATTACCAGCAGGAGAAAATATTACTGAATATTCAAAAGCATGCCTTTTGTGTGAATGATCTTGAAGCCCCAGGGAATGGGGGAAACAGGGTTGGGAGTACATAAGCCAAGAACCTTATTTGATCCAGCAGTTTCCGGCTTCTAAAACCCTACCCATGCAGTTCCAAGAAGAAAATAACAAATTGGCATCACTTAATGTTTAGTGATAGAAGAAGAAAAGCATGCCTTTGTTCATTTTCTACTCTTCTCATTTCCTGCTTCACCATTCCTATCAAATGAAACATTTCGTTTTCATTTCCTCTCTATAACTTGTACTATTTCTGTGAATAGATGATGTGCTTAACATATTGATGTTTGTGAGTAAAGATACTCTTGCTATCATCAAAAGAAATAGTATCCATTTGAGAAGCATCTAGTATATGAGGAAAAGTTTTGTTTTCATTTTTCCCTTATGTTGTTTTTTATATTTTAAATGTAGTTGTAAAATGACAGAACATGGGATCACAAAGAAACACAAAATTCGTAATTAATAAATGTGATTTTGTATTTATTTTAGGTATGCAAGGGGCACGTTTGTGTGGGAGTTCAAAAGCATTTAAATATTTTAAATCTCCTTTCATTCATTTAATAAGTGTCTTTTGAGGTCAGATGTAAACAGACAACTTGTTACACATGTTTCTTGTTTTTAGGGAACTTCCACCCCAACATGGGAAATAAACAGAGACCCTACTAGTTCTTTAACAGTTTCTTAATGAAACAGGATATTTCCCTGACCCCTTCACAGGTGGGAACTGGAGTGCACTGGTGCTGGAACTAGCCGGCTGCTTCCAGGCCAGCGGGGGTGAACCCTGCTCACTCGCTGCTCTACCCCTTGTGGGAGGGGAAGCACAGGTGAGCAGGTACAGGAGCCAGGGCGAACAATTTTGGGCACCAGCAAGAATGAACTCCATACCAGCCCCACGGCAGCATCTAGTAGAGGGTAGCCCGCAACCCCTGAAGACCCAGAGGAAGTGTTACACTGCCTGTTTGGCTTTGCCATCCGCAGAGACCGTAAGTGTTAACAGCTCAGTGGAGGGTCAATGTGACAGCCTTTTGCACCCACACTCATGGCACGCAAGTTTTTGTCCTGAGGTGGGAAATTAAAGAAAAATAAAATCAAAAAGAAAGAGAAATAAGTTTTCCTGTATTAGGCTGACTTTTCCCAGAGGCAGCAACAGGCACAGCCCAGACCCAGGAAAAGTCTTGATAATATTATCTAATGTGCTCTGGAGACTCTCCCAGCACTCCCTCAACATAGGGAGAAGGAAAACAAATTTTCGTTTGTTTTATGGAATGAGTTTATAGATTCCTGTTCTCTGTAACTAATGACTTCAAGTATTCTGTTTTATCTAAAAAGTACAACGAAGGTCATGAGAAGCCTGATTAGGCCTGAACTACAGCTGCTTGGGCACCATAGTGAAGGTTATGAAATAAACCAGTGCAAGGCACTTTAGAGCAAAACCTAGGTAACAGACATCTGGATTGCTTGGCAATGGTCATATGCGGTCCTGAGTTTGTCCTGCCTCTGTATCCCTGCTTTCACGCCACTGTAAGCTTACTTCAAGCTAGCCCACCCCCTTTTGTTAAGTGTGTATGAAAGACAAGTGCTGTCTTTGTTCCGGGCCCAGTCGTTGGACGTTGAGTCTGCTGGGTCTGAGTGCACTCAATAATAAAGATATCCTCCTGTATACACCCCGAGGTCTCTCTCTGGTCCTCCTGATCCCGCAACAGACTGACGTCCAGGAGCAATCAGGTCACACGAACAAATTGAAGATGGTAAATGCAGGGGATTTTTTATTGCTGGTTGAAAGTAGCTCTCAGCAGGAAGGGGAACTGAAAACGGGATGGAGCAGGAAGATAATCTTCCCCAGGAGTCCCGTCATCCCCGGCCAGAATCTTCTCCAAAGCTATGCCATCAAGCTGTCCCTCTGAAGTCAAGCCACTTCTCTCTGATGTCCAACTATAATTTCCGATGTCCAGCTGCTTCTCCCCTTTCCAAGCTATGCCTGGAGTTTTTATGGGCACAGGATGTGGTGCAGGGCAGGCCATGGGTGGTTTTGGAAAAGGCAGCAGTCGAGTGGGAAAACAGGAATGTAAATTCTCACTTTGGGCCCTGGTTGCTTTTTGGCTTGAGGGTGGGGCACTTACCGGGAACCCGCTCTCTTCTGCCCAGAATTTCCCTGCCTTCTGTCCCTATCGGTTTTGTATTTATTTTAGGTATGCAAGAGGCACGTTTGTGTGGAAGTTCAAAAACGTTTAATTATTTAAAATCTCCTTTTATTAATTTAATGAATGTCTTTTGAGCTCAGATGTAAACAGGCAAGTACAGCTTATAGCTGCAGTGAATGCTGAGAATGAAGTACTCAAACAATTCCAGCTGAACGGGGCGGGGAACAGCTCTTCTGAGAGAGTGCTGCCCCAAGATCCATCCACCTGAATATTTATTGAGAGAGCTTGTTTAAACTACAGTTCAGATGAACAAAAGACATCCACCAGGTGGCTCTTTGCGGTTGGGTCATGAGGCACATATGACCTTGTAAAAAACACTCAAACCACATTCTTAGGAGGCTGTGTTCAGCACTCCTTATCACACATACTACTCCCTGTCCTGTTTTCAGGGACAAGGAGTTCTAGTCTCATGCACAAACAACATGCACACAGTGCCTCAGTATTTTTCCATGCCTCGACCTCACGTGTCTTCTACATTAGCTTGAATATGTTGCCATGCACCCCCCACAGGAAGTCATTACACATGTTTCCTGATTTTAGGGGAGCTTCTACCCTAACATGGGAATTAAAGAGAGATCCTACTAGTTCTTTCAAGTGTCTTAGGTAACCAATTAGATATATTCTACACCCCTTAGTGGCAAGTGCTCATGTTGTCAAATTTGCATTTGTTTTCAAATGAGATTAAAACACAACAACAACAATGTTTAAATGTTTCTACTATTAGAAAATAAAATCAATGTATTCTATCTTGGATTTTTCCTTTATTTCTTTATAGAGTTCTGGTTTGCAACAAAGTTTTATCAGTAGCTTATTTACCTTCCCAAGAGCTCGGGCAGGATTTGATGGTGAATGTACATTTAGTGGTTTCCATATTTAAAAAAAAAAAAAAATGACTCTGAATAAGCTCCCAGGCTCTCAGTTTCTTCTAGTTCTTTCTGAAATGGTCCACAACATGATTGTTTTGAAATTGAAAAATTAAATGCTTTTATTTCAAACCCCACCGATCTAAAACCAGTAGGTGTACCTTTCATGAGCACACTTCATTCTGCAGGTGAAAAATTTTCTTCCAACAATTGTCTATGATAGTGATTTATAAGTCAGCAATTTGCTCTAAAGAATGTGTCTCTTTCTAAGCATCACAAGAAGTAATTTAAATTATGCTGTTTCTTAGTAAGCATGTTGATTGAACCTCACATATTTCCACTGATTCTACACTAAACACAGACTCTCTTTTAGTTGTACTCCATTTGACTTGGTTTATACAGTTCACATAGTCACTTTTGTATGTCTAAACTTGCCTGACCATTTTACTAGATGGCATGGTGATATGGTTTGGCTTTGTCCTCACCCAAATGTCATCTTGAACTGTAGTTCCCATAATCCCCATGTGTCATGGGAGGGAGCCAGTGGGAAGTAATTGAATCCTGTGGTGGTTACCCTCATGATGTTCTCATGATAGTGAGTTCTCATGAGATCAGAGGATTGTGTAAGGGGCTTTTCCTCCTTTTGCTCAGCACTTCTCCTTGCTACCACCATGTGATGAAGGACATATTTGCTTCCCCTTCCGCCATGATTGTAAGTTTCCTGAGGACTCCCCAGCCATGCTGAACTGTGAGTCAAACTTTTTTCCTTTATACATTACCCACTCTCGGGTATGTCTTTAATAGCAGCATGATAATGGAAATTGCTACTGAGAGTGGGGTGCTGCTGTGAAGATACCCAAAAATGTGGAAGTGACTTTGGAACTGGGTAACAGGCAGAAATTGGAACAGTTTGAAGGGCTCAGAAGACAGGGAGATGTGGGAAAGTTTGGAACTTTCTAGAGACTTGTTGAATGGCCTTGACCAAAATGCTGATAGTGATATGGACAATGAAGTCCAGGCTGAGGTGGTCTCAGATTGATATGGGTAACTTGTTAGGAACTAGAATAAAGGTGACTCTTGCTATGTTTTACCAAAGAGACTGGAGGCATTTTGCCTGGCGTTGTTGTTCCATGATTTTTTTTTTTATGTTCAACAGGACGATGGCACAACCTAGCTGCAAGGCACAGACCAACTCCCAGCATTGCCAGGGCTTAGGGTACATTACCAGGTCAGCTGCTGACCAGCAGGGGCTGCTTTTCTCTTTTGTGAGTAACTGAGAATTAAATAAACTAAGTAACATGCCTCAAATCCTGCAGAGGGTTGGAGATAATACTGGAGTCTCAACATAGACTATATGGGAAAGTCTAGCCCATTAATCTCCAGGCTTTTTTCTAAGAAACCAAACGCCAATATTTTATTTGTTGCAGAAAAGGGACATCCTGTGGTCAACACAATCTTCAGTGGGAGTTAATTTTAATCAGGTTCTTTAGAATTCAGGAAAGCTGGAAAAAAGAGGAGTTGTGTAACTCACATACTGGGAGGCATCTTCTGTGGCCAGTCAGCAGATACCATCTCCATTGGAGAGATGCAGGCATCTTAAGGATGGGAGAATTCCATTTATAGCCTAGGACTTTTGTCCATGGGCCTGGCTTGGATAGGGATGGCCCATATTAATGTCTTTGACTCTTGGTTTTATTGTTACATTCTGTATGGCTGATTCAGATTTGTCCACACTGATATATTTGTTCTCTGATTCTGATCATTGTGGCCATCTTTTCCTAGAACAAAGGGCTTAGGTTAATTTTTGCGGAGTAATGACATTTTCTGTGGCAGCCAAACTCCGTAGAACAATATTGCTCCTACTTCTTGTTTTCTTCCAATGGTAATTGAACGTGCAAGCCACATTCAGGAGTAGGGTCTGAAATTCCCCAAGAGCTAGCCAGCGATAATAGTGCAAATCTAATACATGCCCTTGAAACACCAAGGGATAAACTCATGTGCATTTGTTCTTTTGGGGTTTGAAGAACCAGATGACATGCAAAAGAAAAATATTGACAAAAGATATCTCATCGTTTACTTTCAATTATTGAGTTTGATTTTCATGCATTCAACCTTAGTTTTTTTAAGAGGTAAGTGATTCTAGTTTGTGAGAGCCAGAAGCATGCACAAATAAACCTTATTTAACAAATTAATCTCATATTTTCTTGGTTCTGATGATTGCATACTGCTTATTTTAAAAAGGTTGTGAGCAAGCCAAAGTTATCATACTTATTTTTAAAGTGACAGCATGGCTGAGCTTTCAAAATATGTTTAAAGATTCTAAGAGAAACAGGTTAGAAAACAAGATGATTGACAGCTTTTTGGGTTATTAGATACAGAAAATTATACTTAGATTTATTTAGGTTGAAAATTAATCCTACAGCATTTAAACCAGCTGGGAGAGCTTGTGCATGCACAAGAGTGTTCAAGCTGCAACTTAAGGCCATTGGGCAACAGTAGAAAGAAAAAAATGGTTATTTCTTCTCTTTCAGAACCAACTGTGACTGATTAACCACAAAAGATCAGTGGGGGTATTCAGGCCTAGGTCGTCTTGGTGGCAACTGGGGTTTTAGTTTGCTTTCAGGCTCATTGCTGGAAAAGGCTGTTCAGAAGCTTCCTCTACAACAAGGGAGATGACAGTGCGTGAGTACAAAGCAGAGAGGTGCAGTGCTTTCTACAGCACCGAGTGGGCAAATTGTGCAGATTTTTCAGTAGAATCTACTTAACACCAATCCATGCATTTGCATTTTATTAAAATGAAACTGTGATCATTTCAACTGCACATTGCAGACATGCCCTATAAAATGTTTGAAGTCCTGTTTTGGACAAAAGTTTTGAAAACATGCACCCCGTATCAATTTCTCTACTTATATTTTGTATTTAATTTGTCTAAAGAATGCCACATTTTCAAAGCAAGCAGGCCAAGAGAATGATCTTTTTTTCCTCTTTTTTTTCCCCAGTGTTTAAAATGCAACTGCCATGGGGCTGTGCCATTTTAGCTGTTGGAAAAAATAATCTACTATGCCTTGGTTGTATGTCTGAGTCATCAGAGCTTCTGGGAATGATTCTTTGGCACATTCTACCAACAATTTAACATGACACAAAATCATTTTCATATCTTGTGATAGTGTCAGCCAAGTGTTTCATACACATGGTGCTAGGTGCTGAAAAAGGTGTCTGAATAAAATTGTTTTCTTAAAGGAACCATAGGGGACATGATAAAAAGATGCACAATTATATATCTTTTTTTTTTTTTTTTTTGAGAAGGAGTTTCCCTCTTGTCGCCTAGGTTGGAGTGCAATGGTGCAATCTTGGCTCACTGCAACCTCTGCCTCCCAGGTTCAAGTGATTCTCCTGCCTCAGCCTCCCGAGTAGCTGGGATTACAGGAGCCTGCCACCACACCCAGCTAATTTTTGTATTTTTAGTAGAGACGAGGTTTCACCATGTTGGCCTGGCTGGTCTTGAACTCCTGACTTCAGGTGATCCACCCGCCTCGGCCTCCCAAAGTGTTGGGATTACAGGTGTGAGCCACTGCGCCCGGCCTAAAGATGCACAATTACATTTCATAAATTGAGAGAGTTTCCTAAACAAGAGAGAGCATACCTGGAAATATCAGAGAAAAATACAAAGGGCTTAAAGATGTTGTATTAAGCAAAGTTAGACTAAGGCAGCTTGGATGTGCATCTCCTCCACTTTATGTTTATACCTAAGTAGAGATTAAAAGCAGAGGAATTTCAATTTCCACATGACTTGTATATGAGCAACAGATGGGAGTTCTAACTACTGACCACATTGGCACATCACACAATGTTTTCTTTCAGGTTTCTCTACCTATGGCAAAACCAGTGCTGTATTAGAGCCTCGTGAGCTGTGTGTTGTTGATTAATTGACTTAACCTCTCTGGGCCTCATTTTTCTCACCTTTAAAATAAATGAGTCTTATGGTGTTTTGAGGATCAAAAGAGTTACTGTACAAACAGTGCTAGTAAGAGTCCCTGCCACATGGAAAGGCTATTATATATATATATATATACGTGTGTATATATATATATATATGTGTATATATATATGTGTATATATATATACACACACACACACACACACATGTAATTTTATATATTAAATGTGTATAATTTATAAATTTTTGTATTATAAATGTAAATCTGTGATATATATTAAAACTATGAAATACAGATCATGTAATATATACTACCTATTGTTTTTTTTTTAATTTGTAACCATATTTTGAAAATTTTATTTTGCTTATAGGTCTTGAAAGTCATTCCCCAATCAACCTTTATTAAAATCCCTTTGATTCATTGGAGAATATCAATACATATGAGGTATTAATATATATAACATATGTAACTCTTCTGAGTTTATAAATGTATGTATAAAACATAAAAATTACTAACTCTTCATATATATGTTTGTATCTATATATAATTTATATATATAGATATATATACATATTTGTATTACATATGAATAATCATCACAGTGTGTCTGCATTTGTTAATCTAACCTCCTCCAACCCCACCCCCAAAAAAGCAGAAACTAAAAATAGAGGAATTTTAAGTTCCACATGATTTATATAGGAGCAACAAATGGAACTACTAACTTCCGACCGCATTAGCTAATCATACAATTTTTTTCTTTCGTGCTTTTGTTGTAAATATGATTTTTATTTAAGAGGGTATTATTGATTATCTACGCAAGAATTAGCCATGTTCTCCATACTTCTACTTCAGTTTTTTAAAAAAGGATGAGGATAGACCGGGCATAAGTGGCTCATGCCTGTAATCCCAGCACTTTGGGAGGCCGAGGCCGGCGGATCACTTGAGGGAAGGAGTACAAGTGGCCTGGCCAACATGGTGAAACCCCATCTCTACTAAAAGTACAAAAGTTAGCTGGGCATGGTGGCGCATCCCTGTAATCCCAGCTACTTGGGAGGCTGAGGCAGGAGAATCTCCTGAACCCGGGAGGCAGAGGTTGCAGTGAGCCAAGATCACGCCACTGTACTCCAGCCTGGGTGACAGAGCAAGACTCTGTCTCAAAAAAAAAAAAAAAGGTGAAAAGGGTGAGGATTGTTATTTCTGTGGGCAGGCCCACACAGCATCAGATTCCTCAGAAACTGCACCGGTAAATGGGAAAGTCTTTGAGTCCCTCTGACAGAGCTTCAAGGGGCTGGCTGTTCATTATCCCACAGCCTCCTTTGCTCTGTGTAAGTGGAGGCTCTGTGCCTCTGTTATCTTGCAGTCCCTAGGTGACCCCGGCAGGGAGAAAAATCAGTGGAATCAAACTCGGTAGCACAGAAAAACGCCCCAAAGGCAAGGATGAGAGGAAAGTTGTGATCCCACATATCAAAGTCGGACTCTTATCTAGATGGGCACACCTGAGCCACAGGCTGGCAGGCTGAGATTCTGCAAAGGCTCTGGACCCCAGATAAGCTTGACTGATTGCATTGTGATCTCTTCTTTTCATCAGGGGAGGCGCTGCTTTGAATGACTAAGCTGGATCTGACTTTCCAGGGAATCCTTTCAGGGACTGTGACCATCCAGCTATCTTTGGATGGCTTTGATGCCCTAATTATTTTTCACTTGGTTGAGGATACTTTTAGGTATCTGTTCATGTGTCATCTTGTACAGAAATGTGTGTTCTGGGCTTATAAAAAAAGTTTAATTGTAAGACAAAGGGCTCTAGGTTTCATATTTATTCACAGTCTGATGAATGGCACTTATGGATACGTACGTGTATACAGTAAGTGCTCACTGAATTTCTCTTGAGTGATAAACTGGGATACAAAATGTCAGAAAAGAAAGAGTGAGGATGGGCACTGGATCCAGATGTCAGTGAACTCTGAGGGTCTCTTGCTGGTTAAAAGAACAGGGTACTTTTATTTTCATTCTAAACCCTGCCTGACCCTTGCCCTTATATCAGTGAATCACCATCTCGATGGCCCCTCAAACATGGCATCTTTGAAGTAGAGCCTCATTGAGAAGGACTCCTTAGAAGTCTGTCATGGCTACTAAAATTCATATCTGTGCTTTGTGCCTGAGCACTAGTACATGTGTCAGCTGTTTCTTAAGCCTACATTGAACCATTAGGTAAAGCCCAGTGTGCTCCCAGTTCCTAAAATCTGGTCAAGTCTTGATGTTGGTCAACATCTTGCCTGGCCCCAGTCAGATGTCTCCAGCTATCTGTAACAGGACTCAGTGTCTTGTTTACAAAATGCATTAGTCATATGGCTTCGTTGCTGGCTTTGCTGTATAGGTCAGGAATAAGTCAGAAATAACCAAAATGCTCCAAATCAAGTTCTAGCTGTTTTGATACCAACATCTTCCATCAACTTCGCTTCTCCCTGACTCATCTGTCTGTCTGTTCCTGTGCTCTTCGCACACAGAGGCAATTTTGTGTATAAAGCTCCCCAAGGGAAGAAGAGGACAGTGCCTTCATGGGAAACTCCTTTCTCTTAAATAGGATTTGCATACTTAACCAGAGCATTTGCTTCAGTTAACCAAGTGAGAGGTGGAGAAATTCTTGCAAAACTATAGCTACATTGAGAGGGATTATTAAAAGTATTGACTCATTCATTAGAGGAGCTGTTACAAAGATTGTAGCAACCAAAGCAAAATAAAAAATATTGCCAAAAGTATTCTCAAACGTATTTTAAAATGTCCAAAATATTGGGCAAGACTAACATCAAAGAAGGTATATGTTTTGACATTGATTTACTAACTACTTATCAGTGTAAGTAAATACACCTTCAAGCACTTATTTAGGATTAAGGTAGTCAAGTTATATGAGTTGTATGAGTATGTGCAGGCCACAAGGGTTGCAAAACATAGTGAATTCAATATCCCTCTGCCATATTGAATATCCTTCTGCCGAACTTCTGCATCACAGTTGTGGCCTGCAAACAGGTAACAGTTGTCTGCCAATCCCTTAGGGATCACTGCATTCTATAGGGCTTGACCAGGAAGTAAGAGGCTCTTCCCAATAAGCGATATCGTTATGGTCCTTGTGGTTCTGCTAAGAATCTCAGAGAAGAAATGAAAGATACATGAAATTGTTTGCATGCTACTAGCTCTAGTGGGTAGGTTGGTAGCGTAGTTCTTCATGGCAAAAGACAGAATATATCCAAAATTTTCACCATTTTGCCCCTGGTTTGAGGGATGCATATTCCTTTAGACCATTATGTTGAAAAGAAAGTTAAAAATAACATAAGAAGAGACCTCCTAAGTTGTTTAATCCAAGCCCTCAATCTTAGCAAGTGCCTGGTGTAAAATGTCTCATTAGGTAATTACCCATCTCCTGTCTACCCACTAAGAGGTTCTAGTAAAGTACATACTGGCTGGATTCAATAAAGCACAAATAGGCAGCAAATGCTTCTTACATCTCAATCTAATCGGTAGCCTTCTTTATCCTCACCCTTGGCTGACTAACGTGCATAAAGCATAGGAATTCTGGCCACTCAAGGATCTTAACCATCCAGTTCAGTCTGTTGCAATTTCTCCTCCATTACAAATTTTTTTCACTTTCCTTTCCTGGGAAAGCCACAGACAGGACAACCATTCAGTGAGAAAGGAGTGTGAAGCTGACGTCTTTCCTCACTAAGAGGAGAGGGGCCATGAGAGGAAAAGGCAACTTCTTGCGTGGCTGGTGGTAGAGTTAAAGTCTGATGCTACTGTCTTCTGGGAGCAGCAGCTGTACACAGTTGAACTTTACTTTGGAGGCATATATGATTTCCAGGGTTTCTGTGGCAAGTTCCACCCACTGCAGTTCATTTGACTTGGGTTGAATCTCTTTCCTCCCTCCATCACTTCAGCTGAACCTCTTCTGTGATCCTCACCTGTTCTCTAGAGGTGAGACCAGGGCACAGTCCCTTTCTAGATGACCAAAGAGCACTTCTTTCTATGTGGTTCACATTTGGCTCCATCACCATCGTAGCTGACAGGGCCAACCCTCCGGCATCTTCATCCTTCACCACTGTCTTTGCTGTGCCCCATAAGGCCTGAACAAGGCTGATGGGCCAAGTATGGTGTGGCCAGCCCCACAGTCTGTTACTAGGCCTTGCTTTGGTAGACACACTTCTTGATTTAGAACCATGGCTCTCAGTCATGGGCAGTTGTGCCCTGCTTGGCAATGTAAGGAGACATTTCCAGTTGTCAGAGTGAGTTTGAAGGGTGTTAATGCACTTAGTTGGTGGAGACCACGGTTACTGTTCAACATCCTACAATTCGTAGGACACTCATCCATAACAATGATCTGATTCCAAATGTCATTGATGCTGACATTAATAAACCCTGCTCTAAGTTAATGTTTTTTTCTTACTCATATTTAAAATGCTTCCTCTAGCTAAACCATTAGCCCCCAGTGAGGTATAAGTTTTCCTCTCCAAGGGACATTTGACTATGCATGTACATACTTCGGGTTGTTACAGCTGGAGATTGGTGATGCTTCTGGCATCTAATGGATATAAGTCCAAGATGTTGCTCAATATACTGCAATGCAGAGGACAGCCCACGAGAACAAGGAATTATCCCATTCATAATGCCACTAGTATTAAGGTTGAAAAACCTTGGTTTAGAATATGGGGATACTTATTGGTGCTCCCTAAGGTGCTATCTGAAAGCAGCTTTGAAGACAAGCAGAGGCTTTGAAGACATACTCACAGGGTATGATATAGTTTGGATATTTGTCTTCTCCAAATCTCACGTTGAAAACTGATCCCCAGTGTTGGAGGTGTGACTTGGTGGGAGGCATTTGGGTCATTGGCCGGATCCCTCATGAATGACTTGGTGCAGTCTTCCAGGTGATGCCTGAGTTCTTGCTCTATTATTTCTCAGGAGATCAGGTTGTTAAAAAGAGCCTGGCACCTTCCTCTCCTCTCTCTCTTGCTTCCTCTCTCACCATATGATCTGCGCACACAGCAGCTCCCCTTCCTCTTCCACCATAAGTGGAAGCTCCCTGAGGCCTCACCAGAAGCAGATGCTGGTACCATGCTTCTTGTACACCCCGAAGAACTGTGAGCCAAATAAACCTCTTTTCTTTTCTTTTTTATTTTTCTAATTAGAGACAAGGTCTTGCTCTGTTAGACTGGAGTACAGTGGTGCAATCATAGCTCACTGCAGCCTCAAACTCCTAGGCTCAAGCCACCCTCCCACCTCAACCTCCCGAGTAGCTAGGACTACAGGTGCATGCCTCCATGCCCAGTTAATTAAAAAAATTGTAGGGACAGTCTTGCTGAGTTTCCCAGGCTGGTCTCAAACTCCTGACCTCAAGCGGTCCTCCTGCTTCAGCCTCCTAAAGTGCTGGGATTACAGATGTGAGCCACCATGCCTGGACCGTCTTTTCTTTATAAATTGCTCAGCTTCAGGTATTCCGTTATAGCAATGCATATGGAGTAAGACATTGTACAAGTCCCACTTTGGGCACGTCTAGATCTGTCTGTGATCCTAGACAAGTTATGTAATCTCTCTTTGTGTCTAAACCTGTTGTTTGTTTCTGTCTTTATTCCTCATTAGGTCCAACTCTAAAGATAGTAAAATTATAGGTATAAATGGAGTTAAGAGGGGTGCCTTACCAAGAGTAAACCCTCCAGGAGTGTTATTCTGTCAGTATGACTTGGTTTTTAGCTTTGAAACTTTTAGCATGAAACTAACATGGCAGGAAAAGGCCTAAATTAGAATTCTTCACACACAAAACTCCTTCTATCAGGAGGCAGCCCATCTGTTGTCAAATAATCCTACTCGTAGAAATGTATTAAATTTTTCTTTTCCTTCCCTTTTCCCCCTTCATTAAATGGAATTAGATTGTGACACTATGAGGAAATTAAAGTGAAGGTAAAATAAAACAAACAGGAAGAAGTCTGTCTTCAGATTGGATATGCAATTATCCTGTCTTTACTGCTGATTTCAATTATAACTCATTGGTGTTACCAGCCCACGATAGATGTCCCCTGCCTATGTGGTGTTTAAATCAAGTGTTGGCATCATTCACACTTGTTTACTGTTATTAGCACTGATGGATGTAATCTTCATGTCTTCCTCTGAACACTGCATGCTGAGAAAGGGGCCTTATTTCCTCGTGGATTTTCTAGGCAAGAGAATGTCAGGCCCTCACCTGTCCTATTTCCATCTCACTCAGCAGAAAACACACTGGCTCATGGAAACTGCAAGCATCGTTGTCAGCTGCACCTGCAGGCACCATGGGGTTGCAAGTCAGCATCCCCTTTCAGAAATGAGGATGGAATTAGAGGTGGAAAGAAAATTCTCCACAGTCCTCTCACTTCTCTGGGCTTAGACAGGGAGGTTTCTGCTATGTTTTCATTGATTATGCTGTGGGGGGAAGGGAGAGGAGGAATCCCCTAAGAAGAACAATGTCTCATTGGATATTGTTCCTTTGGGGGAAAAAAAAAAAGGAAAGGAAATATTTTCATTTTTTCTTACTTTTTCTACCCTAGAATCTCAATGCCACCTTCAAACATTTGAATCTCACAGGGAGAAGGCGGCCACATATTTCACCCCCAAATGCTAGGCCATGTCTTCTCATGTCAGAAATGCCCTATTGTGCGTGTGTCCTTGTTGCAAGCCATCTTAGACTTGTTGTTTCAGGGATAGGGAAACCATTCTGCAATCCAAATAAGGTTGCATTTCTTGCAATTCAAAATAAAAGGTGTGCATGCACACACGCATGTGCTGGTATTATTGTACAGCTTGCGTGGTGCAAGGCTGAAGGCTAAGGGACTAATGGAGGCTGAAATTTAGCCCTAGATACACTCTGCAAGCTGAGTACCTGTGGGGCCGTATTACCTGGCTAGAGGTGTGCCTATTTCTCATGCATCCAGTATCAGGTACTTTTCTGACTTAGAGGGTCCCTCAACCCTCTCCTCCTTCCCCTCCACCTATCGTACTTAGCATACTGTATATTTGCCCTTAGTCTGTTTCATCCAACTTGATCACTTGGTAGCCTGTCTTTATCCCCACTGTCTAAATCAGTATTTGGAATGTAGTAGGGACACAAAAAAAATTAGTTGAATAAAGGAATAAATGGGTGAAATAGTGAATGCATGAAAAAGGAAAAAATGAATATTTTGGCTGCTGTGTATTCTTGTATTGTTGTTATATATAATTCTTCTGCCTGTCTTTCTTCATACATACCTCATTATTAGTATAAACTACCAGCATTCGTGATATGCAGGTCTTTGCTTTTGCAGAGAGCCATGGGTTTCTCTAAAAGGCATCTTGCAGCCTCCCGCCCAGGGTGTCTCTGTGCAGCTAACCTGGTTGCTAATCTCTGCAAGCTCGTACTTTTTCTGCAGCACGTGATTCTGTTCTCATTTACTCTTGTAATCCTTCTGTTTCCTTCTGACCAGCTTGAGCTTCTGTATCTAGTGCCTTGACGTTCTCTTTCTTTCTTGGTCTTTTTAACATTATTATGTCAGTTATAATGTTTTTCAGTTGCTTTTAGTATTCAGAAAATTCTTGAAGCCTTCTTATTGCCCACTGGTATTTTGTCTTCGCCGCTTGTTGTTTGGGTGGATTTAGATATAGCAGAGAGAGAGAGAGAGAGAGAGAGAGAGAGAGAGAGAGGAAAATAGAGACAGAGATATGTAATCCCCCCAACCAACCCCCGTTATCTGTGATTTCCATTACCCATGGTTAGGTTAGTACAGTACAGTGATATTTTGAGAGAGAGAAAGAGACATCACATTCACGTAACGTTTTATTAGAGTATATATTGTTACAGTTGTATTTTATTTTAATTGTTGTTAATCTCTTACTGTGCCTAATTTATAAAATAAACGTTATCATGGGCATGCAGGTATAGGAAAAAACATTGCATATATAGAGTTTGGTACTGTCCACAGCTTGAGGCATCCAATGGGGGTCTTGGAAAGCATCCCTCACTGCCCCTGGTAAGGAGGAGCTACTCCAGTTTTGAGAGGAGAAACTAAACAGATATGAAAAACATACAAGTTGTAACCTAATAGGAAAATTTTTAAAGTGTTATTAAAAACCATATCTTATATATCTCATATATTAAAGGACTTCACAATGGACTTTAGGAAATTAAGATGGAAGTTGCAATAGCAAAAGTTTAGCAATGCGTATTCTTACATATGAAAATCAAAATTAACCTAGCAGTGTTCTGAGCAACTTCACTTTAAGAAGTAAAACTAGTGAAATGATAAAGGTATATGGGTGCTGACTGTTACGTAATTAGGCTGATATAATTTAGCAAGGATATCAGAAATCATATACCCAAAATGAGCTTTATTATATTCAAATTAGTCACTTCAGAGGCAGTACACTAATTACAATAAGGTAAGACTGCTGGAAACTTCTTTATTTCTCCTCACTTTAAAACGTTTCAGAGCCCATAGTAATTTATTTTTAATATCTTGCTGAGGCAAGTCTTAATCCTTAAGGAGGCATTTATATTTGGATACAGCCAGGGTTCTGTTGAGTAAGGTCAGTGACCACATTGTATAACACAATTTTAATTCAAAGACAAGGAACAGCTATAAATAAAGGTGAGCTTGTTTCAACTAACTCTTTTTTATTTTTTTTTTTATTTTTTTTATTTTTTTTATTTTTTTGAGACAGAGTCTCGCTCTGTCGCCCAAGCTGGAGTGCAGTGGCATGATCACGGCTCACTATAACCTCCACCTCACAGGTTCAAGCGATTCTCCTGCCTCAACCTCCCAAGTAGCCAGAAATACAGGCACGTGCCACCACGCCCAGCTGATTTTTGTATTTTTTTTAGTAGGGACGGAGTTTCACCATGTTAGCCAGGCTGGTCTCGAACTCTTGGCTTCAAGTGTTCTGCCCGCCTTGGCCTCCCAAAGTGCTGGGATTACAGGCGGGAGCCAATGCGCCCAGCCTCAACTAAACCTTAAGGCACATTGAAAAGAAAATCAAAATGCATTGAGCTAAATGCCAGGCATATGCCTTTCCAAATGGACTTGCCATGAAGGATGTCATTCCTGTGCAGCCAGGTGTTGTCTTCTATGTATTTTTAGAATGCCCATCATATAGTCTCACCTTTTAAAGTCTGTTTAGTGGAATGTTTTCTAACTTTCCCATGTACCTCCCATGTCATTTTTTGCCAGTTCTGCCTTCCCTAATAACCAATGAAGGTACTTGCTTCATGTTAAATTCTAGGTAATCTGGTTTCTACTGAATTAGAACATTCCCACCCGCCAATGTCTTTGAATAATTAAAGGTTTTATAATGTGGTTTCCATACAACTAACTGAATATTTCATGTGGCTAGATAAATAGGTAAATTGCAGTACAGTAGCAATTGGTGTAGACACTTAGAGGGTCCTAATAAATTATTGCACACGCCAATGTGCAATCAGAAAGAATAACTGTAGTGTTAAGCCTCAGACAATGCTATAGACCTGAGGATGGGCCTGTGATGGACGGATCAATGGCTCAGTTCCTATTGGAGTTTCACATCTAGGAATAAGTGAATTCACGACTATTCATCAGCTGCTGCTACTGTACGGAAGTGTGTCCATTGAGAAGTTGCAGAAGGGGCTGGGAGATTGGATAAGGCTTTTGCAGTACCCCTCCTTTTTAAAAAAGCAGACAGGGTGTAACTCTATTGCAGGCTGGAGTGCAGCGTTGTGACCATGGCTCACCGCAGCCTCCAACTCCTGGGCTCAAGTGATCCTCCTGCCTCAGCCTCCTGAGTAGCTAGGACTACAACTAGGCACCACCATACCAAGCTAATTTTTTTAAATAAATTCACTGAGACAGAGTCTTACTATGTTGCCCAGGTGGGTCTCAAACTCCTGGCCTGAAGCAGTCCTCCCATCTCAGCCTCCCAGAGTGCTGGGAGAACAGGCGTGAGCCACGGTGCCCAGCCTCAATACCTTTTAAATTAACAGGAAGTGGAAAACAGAAATTCTGCAGCATGTTTTTCTCATTAGCATGAATCACTCTCTGGTGATGTGTTCATGGTTTCTAATGGTATTTTCAAGATGGACAATATAAAGACAACCATTAGAAACCACAAATAATAGGGCCATATGAAACAATATAATAGATGCATGAGGTTAACTGGTCAACATTTATGCTGAACTTAGATTTACACTGATTAAAAAAAATAATCCATTTGAAGTGTAACACACAGAAACCAAAGTTCTGTGTGTTCTGTTATCTTATATTATCAATGCTCCATGCAATGTGAAAGCTTAAGGCAAGTGTTTCTATAACCAACACCCATGTGAAGAAATATAGTTTCCATCTTCAAAGCAGTGCATGCTCTTTTCCCATTCTATCTCCTTATCCTCCTCCGTGATAACCATTATTCCCTTTTACTACTCATTTCCATGCTTTTCTTTATATTTTCCCAATGATAAAGGCATCCCTGAATCACATAATTAAATTTTGCTTGTTTGGAGACTCTAAATGAATGCAACTTTCTATTACTTTCTGGTGTGTTTTTTTCATGCATAATACTGTTTTATAAATTTCATATGTGTTGCTGTGTATACATCCATTCCACTCATTTTAATTGTTGTATAGTGTTCTAAAGTCTGAACATACCACAGTCCCTATGTCCATTTTATTCCTAATAGATATGGTTATTATTTTGAGTTTGAGGTTATTATAAATTCGTGTTATTAACATTCTTTTTCAGGCACCCTCCTTTCTCACAAGCATTGGTTTTCTGAGACATATACCATTATGGAATTGCTGGTTCAAATCTTCAACTGTATAGTTTATATAAGGATGAACTGTTTTCCAGTACAGAAATGCCTGTTTTCACCAGGAGTGTGCAATCTTCAACATGTGGCAGTATAAAAGTTCTATTTTATTTTTCTGATCTAGCGTGTGTACATGGAAACCCATTGTGTGTTCACTGTGTTTACTCTGAGGTTGAGACATTTCCATATATCTCTTGGCCATTCATATGTCCTGTTTGGTGAAGCGTCTGTTTTTGATCTGTTTTTCTACTGGGTTGTGTGTCTTATTGCTGTATTTCGATTAGAGTGCTTCACTGATTATATATGTTGCAAATATCTTCTGATTTTCCTTCCATGTTTTTAATGATTTATTTAAATAAGCTAAAGTTCTTAATGTTAGTTTATAGACTTTACAATATTTTCTTTCAGATTAGTGCTTTGGAATTTTTGTTTAGGATATCTTTTCCTACCAAGAGATATGAAGATTTCCTTTTATTTTATCTGAAAAAAGCTTAATATTTTATCTTTCATATTGAAACCACACAGGGAATATATTTATTGCATTCTGTAAGAGGTCTAGTTTATTTTTCCTTAGAATATCACAATACAATTTATTTTAAACAGTTTGATCCATGTCACTAAAGTTCAAGTGATCTCTTTGTCTACCTCTGTGCCAATCATCACATTTTTATCTTCATGATTTTATAATAATCCGCAATTTATATTTTTATACTTTGTTTATTTCTTGCCAATATGCATTGCATCCCTGAGAAAAGTGTTTATTTTGCGATGGTTGGTGCAATGTGCTATATGTCTAATATCTCAAACTGTTGAAGTATGTTGTTCACATACTCTATATAGTTTTCCAGGTGGTAGTTTACATATTCTTTCAGTAACTAAAATAGGTCTATTAAATTTTCCCACGATGTTTATGGATGTTTTAAAATCTTTTCGTATATTTTTCCAAAATTTAGTTTCTTGCATTTTATATGCTTATGAATTTTAGTGGATACAGTCTAGAATTTTTATTGCATTGTGGCAAATTAAGGTTCTTCTCATTATAAAGTGATCCTCTGTAAGTCTGTGGTGCTTCATGCCTTAATGTCTGTTTAGTTTGACGTTAACATTACCTTTGTTTTGTTAGTAATCCAATTGTGTATAGTTCCCATGTGTTTACTTCAGGCCTTTCTGTTGACTCAGGTTTTGAGTCTTTTCTACATAGCGTCTATTTGGGTCTCATAATCTTTGATTTTCAACCGCAGATCCACTGATATTTACTTTTATTTTTGATATATTTGTGTTTAAGTCTTCTATCCTAAATTGTGCTACTAATATCCCACTTCTACATCTTGCTTGAATTGCTTTTTAAAAAATCATTCAGGCCAGGCACAGTGGCTCACACCTGTAATCCTAGCACTTTGGGAGACCAAGGCAGGAGGATCACTTTAGAATCCTCCAGGAGTTCAAGACCAGCCTGAGGAACATAGCAAGACCTCATCTCTATGAAACATAAAAAAAAATAAATAAATAAAAAAAATAAATTAGCCAGGTGTGGTGGTGTGCACCTGTAGTCCTAGGTACTCCAGAGATAAGAGTTGACAGGAGAGTCTGATCCCATGAGTTCAAGGCTGCAGTGACCTATGATGGCACCACTGCACTGCAACCTGGATGACAGAACAAGATCCTGTCTCAGAAAATAAAGAAATAAAAGACAAATAACATTACTCCATTTCCTTCACTCCCACTTCTCCCTCTACACTAGATGTTAAAAGACTGTACTAGTTTTAGTAAATAACCCTAGAAATTACAACACAGATCCTTAATATAATCACTAATTTTAATTAATACATTTTCCACTTCTCTGAAAATACCCAGTAGTCAGTGTATTTTAGCTCCATGTTTATGACCTAACCTACTTGCTGTTAGTACCTTTCAATGTTTTGTGTTTTTTAGGAATCTTTTTCAGATATGATTGCTTATCTTATTATTTCAATATTAATTTTGATTTTCTGATGATTACACTATTTTATTTATGTTTCATTACTTTTTGTACCTCCTACTTTTATCTGTGATTATTGTCTTAAAAGAATCTATCGGTGATCTAAAATATATTTTCAGAGCTAACAAGCTGTTGGAAACTCTGTTTGCATGGCTAAATGTGTCTTTATGACATCCTCTTCTTGAACAATATTCTCATTGAATTTTAATTTGCAATTACTTCTTTCAGCCATCTGAGAAATCATTCTCCTATTCTCTGGATTCCATTATTGGTATGGAGAATTTAGCTGTCAGTTTAAGTGTTGCTCCTTTAAAAATAATATATTTTCTGCAGATAGTTTGTCTATATCCCCCTGATACCTTTAAGATAGTTTTTCTTTGAGTTTCTGCCGTTTCACTGTGATACCATTAGGGGTTTATTAATCTGATTGGAATTCCTTGATGACCTTGAAATTTGCAATCGTGGTTTCTTCCATTCTGAAAATAGTCATTACCTCTTCAAATTTTGGTGCTGTTTCTCTTGTTTTCACTCTGTTTGCACATAATTTAGATTTTCTCCCTCTGGCTCCTTTTTTAGTCTTTTTTTTTTTGTATTTTGTATTAAATTTTACTTTCAAGCTTCATTCTGGATTACTTTTTCTCAAGACCTATAATCTATTTCATTAATTCTCTTTTCTACTGTATCTAATGCATGGTTAAACCAATGCATCAAATCTTTATGTTTGATATATATTTTCATTACATTTCAAGGATTAATTTTAGTTTCTTCTTATAGTTTCCACATTTTCGAAGTTCTCAATTTTATATTTTCTGGAATGCATTCTTCCTAGTTATTTTAAAGTCTGCATTTTGTATTTCTATTTTTTTCAATCACCCTTTTGTTTCTTTCTCTTTTTTGCTTTTTGGTTTCATTGACTAATATCTTCATGGTCTAAGTATTATAATTATGCATATATTAGATATTCTCATATTGTTTTCCTTATTTCTAACTCTCTATTTTATATTTTTTGTATATGACAGCTCCCTGTGTTGCCCAGGCTGGAGAGGTTGTGCTCTGTGCCCAGTGGCACAATCATAGCTCACTGTAGCTTCGATCTCTTGGGCTCATGTGATTCTCCTGCCTCAGCCTCCTGAGTAGCTGGGACTACAGTCACATGCCACCATGCCTAGCTACTATTTTATACTTTAAAATTTTTTTAGAGACTAGGTCTTGCTTTGTTGCCCAGGCTGTTCTCTAATTCCTGGCCTCAAGCAATCCTTCTAACTCAGTCTTTTGAATAGTTGGGATTACAGGTGTGGGCCACTGCACCCGGTTTCCCAGCTTTTTTCAGATTTCCACGATACTCTCTGGATCGTTTCTTCTCACCTCTTCTCAAGTTTGTCCATTTTTCTCTTCAGCTTTGTTTAATCTGCCCTTAGGTGGACCCATTCATTTTCTCATTTTGTTTATTTCTCTGATCTAGAAGTTTGATTTGATTTTTATTTTTTCATTTTTAATACTTTCTTATTCCCTGCAGATGTTTTCCAACTTTTTGTTTTCAAGCTTTTTGAACATTCTTCAAAAAATTGGTTATCATGTATATATTTTCATGGCATCTTAATTCCTTTGGGATTTCTGCTGGCTCTTGTTGGTGACTTCTTGTTTCTTTCTTCATGGGCTTGGTAATCATTGTGAATTGGCCATTGTATTTGCAAATGGATTAGTGGCATCTTTCTCCAAAGCAGATAACCCATGGGTAGCGAAATTCTAGGTTCTTTCATCCATGGGGCCATGCTCTTCCCTGAATTGTTCATAGATGTTATGAAGGTAGACTGCAAGCACTTGCAAGACTGAATTTAGTTTTGTTTCATGTTTGCCTTGAGGGTGAAACCCATGAAGGTAGGAAAATGTTAAAGGCAAGTATATTAGATTGGGACCTTCAGGCGTGACTAGGGTCTGAGAGTTGCCCCATTACATGGTGATGCTGCAAGAACTCCCACAGTTTCTTCCAGATTGGAACAGTGCACTAGGGCAAAGGCTGCTTTGTGTGCTGGGCATCTAGCTGGATCATCATTTGGTCGTCAGTGTGTTTTTGTTTGTTTCTTTGTTTTTTGTTTGTTTGTATTGTGTTTTGAGACAGGGTCTTACTGTGTCATCCAGGCTGGAGTGCAGTGGCACGAACAGGGTTCACTGCAGCCTCGAACTCCTGGGCTGAAGACTTCCTCCCACCTCACCCTCCCCAGTAGCTGGGACCACGGGTGTGTGCCACTACGCCTGGCCACTTTTTAAAAAATTTTTTGTAGAGACAAGGTTTCACCATGTTGCCCAGGCTGTGATAATCAGTTTTGAAGCTGTAATCTTAAATATGATTTTAGCACTAAAATGTTTTTAAGAGACTTAAAAAAATCACACATATTACAATCCATTTTCAATAAGAAGGTTGGTTTGAATAATCTACTCTGTTACTGCTAGATGTAGGCTTCTGATTTATTCTAATATATTACAGAAATGAGTAGGTGGAACATGAGTTTATAAAGATAATGCAAATATTTTATTAGCACTGTATTCTCTTAAGAGCAGTTCAGAGTTCAAAGAATTGTGACTTTATTTCACAGGCATTAAAATAAATTAAATCAGCAATCTCATTCCTAACAACTCAAACTTCAAAGAAATTTCAGACAGTTAATCATCACCTGACACCACAGCCTATGCAACTTGGGTTTAATTAGGATTTATGTTACTGGTAGCATTGTGGTTGAAAAGATATTTTCATTAACATTTCTCTCTGAAGCACTGAGTCATACTCTTGTTTATTCGCAAGTTTCTTTACACTTTTCAATCAATATTTGAGTGTTCCTTGGGAAATGTATGTTTGGCTATTTTGGTGTTTTTGAGAGTGTTTGATCTTTGAAAATGCATGATTAAAAGCCATTTTAGAAATAAACATGAGTGTTTTAAATACAAATTACTAAAGCCACTGTTTTGTTTCAAATTTAGGGATTTAATTTTTTTAATGAAAATGCTCCTGTTTATATATGCATGAGGTTATGTAAGGTCATCAACTTAAAGATTGATGATGGATTTAGTGCCAGCTGTTGATTAGTATGTCTGCAATCAATCTACAACATAGCAATAACGCTAGCTACCTTGGAGAGTTACTGGGAGAAATAAATAAGACACAATGTATGTAATTGGCCTAGCAAACTTCTTTGTATACTATAATTATTCAGTAAATAATACCCTTGTGATTATTTATCTATCAATCAGTCTTAGAGCAGTGAATTTACCTTTAAAATCTAGACACATTAGGAAAGAATAATGGTAGATTTTAAGACAAAATTAAAATTTCTTGGTGTACTCAAAAATATATATTTTCTGTTAATGCAAATTAGGCTTTTATATTTATTATTTTTAATATTTGACTCTGGAATGTTTTCAAAATTTAGTTGAGTAGATCTTAATGCAAGTCTACTTTTAAAAAATCTCATTATCTAGTAGGCTTTACTAGTAATTAATTTGAATTTGGTAGACATGAAACACACCAATTTCTTGTACACAATCATAAATCCTGTATACTATGTATACTCTGTATGCCTGTATCTTGGTGAAGTGGGAATTAAACTTTATCAAATTTCCATTGAAAAACTGAAGAGCAAACTAAGATGTAATCAGAATGTTAATAAATATTGTAGAAATGGAAAAGTTTCAGAATGTTTAGATTTCTCAAGGAAATCTCAAAGCATGACACTTTTCATTGGTCTGTCATGGATAATTAGGTCTTTTGCTATTTTTATTTATTTATTTCCAATCCGTCACAAACGTACTTTGGTTGATGCATATATCAACTATAGAGTAGTAAATCTGACAAAGTCTATGCACTGAAAACTATACTCTGTCACTGAGGGACACTGATGAAGGCTTAAGCAACTGGGAGACAGACTGTGTTCACAAACACAACACCCTCCTGAGAAGATACAATATTGTTAAGATATTTATTTTGTACAAATTAATCTACAGACTCTTTGCAATCCCAAATAAAATAACAGTAGACTTTTAGAAAATACATAAATTAACAAGATAAATTTAAAATTTTAATGAAAATACAAAAGATCTACAATAACCAAAACATTTTTGTAGCAGTAGAACATACTTGGAGGGCTCCTGCTACCTGAGCTCAAGACTTAGTATAGAGCTATATTAATTGAAACAGCGTATTATTGACATAAAGATGTAAAACCTGATCAATATCATAGACTAGAGACACCACATAGAACTGTACATATATGGACAATGAATTTTCCAAGGAGATTCAAAGGTAATTCTATGCAGGAATGATTTTTTTTTCAAGAAATGGTGTTGGAAACATTAAGTATCCATATACAAAAGAAAAGAAAAAGTAAACAAAAAGCTTTGATCTATAACTCACAATTTGTACAAAAAACAACTGAAAAGTGAGTCAAATACCTAGATGTAAAGCTTAAAATTGTAAAACTTCCAGGAGAAAAAAAAAAAAAAGAAAAATTTTGTGACTTTAGATTTTGGCAAATATTTCTTACTTAAAACAAGAAGCTTGATTTTTAAAGGAACCAATTAATACATTGGACTACATCAAAACTTAAAAAAATGCTTATGCTACATGAAAGACATTGCTAAGGGAATGTAAAGAGAATTCACAAACTGGGAGGTAAGATAGGCAAATTAAATATCGGATGAAGGTATTGTACCAGTATAAATGTATGCATACATACATATATATGATGCAGTTTCCTATAAATATATAGTATATATGGTATTAGACATATATGTATAGACACGTACTGGTACAATTATATACTATATATACAATATTCATATATAGTATATATGATACAGTATTGTATACTATATATAAAATATATCATATATTTACCATACAGTATACTACACATATGTATATATATGATATACTGAGTATCACTATTACTAAAAATTACAGAATGTGAACTATGAAAATGTAAAAGCCTATTTAAATAAAATAAATATTTAAAATACTGTGTTTTTTATATATATAGCACATGTAGTATACTAAATTGTATACAGTATAGTATATATAGTATACTGTATCATATATATTGTCAATATAGTATATAATTTACCCCTGTGTGTGTATAGATGTGTGTATATGTGTGTATATATACACATATATATGTATGTGTGTATATATACACATATATATGTATGTGTGTATATATACACATATATATGTATGTGTGTATATATACACACATATATATATTCTAAAAGGAGAATTAAAAAGAAACCACCCCATAACAATTGGACAGAAAATTGAACAGGCAGTTCACCTAGGAAAACATACATATGACCAATAGCCCAATGAAAATGTGCTCAGCATCATTAGTCATTGGATAAATGCACAAATGAAACCACAGTGAAATACCACTACACATCTGAGAATGGCTGAAGCCACAAGACTCGCTATGCCAGGGCTTGGTGAGGATTTGGAGGAGCTAGAGTCCACCCCAAGCTGCTGGTGGGGAAGTGATATGAAACCAGGACTTTTGAGAAGAGTTTGGCAATTTTTTTGTTGTTAAACCTACAAGTACCATGTGGTTCAGCCATTTAACTCCTAGGTATTTACACAAGAAAAAGAGGAGCATATGTCCATACCAAGACCAAGAACCTGAATGTATTCATAGGCTGGAATGCTTCTGAGCAGTAAAAATGAATGAACTGTTGGTGCATGCTACAACCTGCATGAATATTAAAATGATTATGCCAAGCCTAAGAGGCCAAGCAATGAAGAGACCGTAATTCTGTTACTTCGCTTTTAATATTTTGGAAGCTGTAATTCATAATGCCTGTCTGTAAGCAGATAACTGTTTGCCTGAGATGAGGAGGAGGAGCAAGAGATATAGATTATAAAGGGATATGGGTAAACTTTGGGGTGTGATATATATATATGTACATGTATATATATGTGTGTGTGTATATGTGTATAAAATACACATATATGTATATTTTAAACAGAGTCTCACTCTATCACCCAGGGTGAAGTGCAGTGGCACAACCTCGGCTCACTACAACCTCCACCTCCTGGGTTCAAGCAGTTCTCCTGCCTCAGCCTCCCCAGTAGCTGGGACTACAGGTGCATGCCACCACGCCCTGCTATGTGTGATTGATATTTCTGTCACCTTGACTGTGGTGATGGCTTCATAACTGTATACATAAGTCAACATTTATTATACTGTATACTTTATGTACAGTTTATACTTTTACAACTATAACTTCAGAAACCCACTACCCTATTTTAAAAAAGTTAATAATTACTCTCAGCCACTGTGAGACCTCACTGTTTCCTTATGCTCATTTTTCCCTTTAACAACAATGGGGAACTAGTATTTTATCAGATAAAAATAATGTTTGATAGGATTTTGTGCAAAGTCTGTTTTGCCTACTAATTCTGCCTTATGGCATCTCAGACATGTAAATTAGACAAGAGCCTTCAGTATGTCTGATCTGTTGTCACGTTATTTTCCACTAGTTTGTGTGATTTAGATTATTTTTAAAGAGCTGATAAAGGAAAGGAAAGGAAGAGAGAGATAGAAGAAAGAAAAGAGAGAAGAAAGAGAAAGAAAGAGAAGGAAGGGAAAGAAAGAAAGAAAGAAAGAAAGAAAGAAAGAAAGAAAGAAAGAAAGAAAGAAAGAAAGAAAGAAAGAAAAAAAGAGACGCCTGTCTTTTTAATTCCAGTTGGAAGCAGCTTTAGTTATAAAATTTCCACTCTCTAGAATATTCTTGGGGAAAAAATGAAGTGTCAATTAAATTGATTTTTTTAACTTGCATCCTATGTCTCTGAACATGATTCTTTTTCAATCAGGCATGTAGTTATTGAGGACCCATTTATGAGCTGTGCATACATCCCATCCAATTCCATCCAATTCCGTCCAATCCTGTCCACAGACATGTTGAAAGCATGAGCTTCCTGCAAGAGCAATGCACCAGCCGTTTTCCTAGAGATGGGTCTTCAAAGAGAGGGTTCTTTCTCGGAGCACCTGCTCAGGGAACAAGACTGACTTTAAACCAGTGTTAGCAATATGCATGGTACACTGAACCATCTGCTGGAGGACCTCCTTGTGTCCAACACAGTCCTTCTGTTGAATGTCATGGAAAAGACTGAGGGTTGAAGCAAATCATTTTATGCAGTGAGGAGAAGACCGTGCTCATCTTTCAGTTTTTGAGCCACATCTACCTAATTTATAGTCAGGTTTGGTAGCCTCAGCACTACTGATATTTGCTGCATAAATCTATGCTTTGTTGGGGTTGTCCTGTGCATTTTAAGGTATTGAATAGCATCCCCAGTTCACACCCACCAGATACCAGTATATAAATATATACCGTTTTTGCCAATTAAAATGAATAAGAAAAAAATCATTGTTACAGATTAATAATAATAATAATAATTAATAATAAGTGGCTGGACACAGTGGCTCATGCCTGTAATCCTGGCATTTGGGAAGGCCAAGGCAGGAGGATCCCATGAGCCTGGGAATTTGAGGCCAGTCTGGGTAACATAGTGAGACCCCATCTCTAAAAAAAAAATGAAAAATTAGCCAGGCATGGTGATATGTGCCTGTAGTCCAAGCTACTCAGGAGACTGAGGCAATAGGATCACTTGAGCCCAGGTGTTTGAGGCTCCAGTGAGCTAGCTATTGATGGTTCCACTGCACTCCAGCCTAGGCGACAGAGCAAGACCTGGTCTCTAAAAAATAAAAATAAGTAAATAAGCTAAATGCTCTTGAACTGAAAAAAAGAATGTATTCTATGAGAGATACCTGATAATCACCTACTTTGACCATGTTTTTATCCTTCAAGGATTTCAAACTGTTACAACAAACTTCTAAACGTGTATCTCTTTAGTTCAGCTTCCTTACATGAATTTAATGCTCCAGTATGTGAGACCAATTATTGATTTAAAAAAGGGTAGATCTGTTTTAAAATTCCTTTACCAATATTCCTCATGCTCATGAGAAAGATATGAGGCAGTGCTGTTGACTGCATTTGTATTTAGTTAATACCACGAGCAAGTGGGAAAAATTCAGAAGTGACACTGAGTTGGTCATCTCTCAATTATCATCATGAGAAGTACGCACAATGTGAACATTCTGCCATAGGGCTTGTCTCTGTAAACTGCTGGTCAAGGGGCATGGACAGATTCTACTATTTTTAAAAACATCTTTCTGAACAGATAACGGAGGCTTAATTGTAGTGTAAACACACTGATGTACAAATCTCGAAAAACATAAAATAAAGTGTGTTGAGATTGGAGGTGCTCTGTTCAACTTTCGAGGGATAGAAAATATGCCTATCAGCTGTAAAAGCGGTGCATTTATTTTCATTTTTTGAGACCAACACTAGAGCAGAAAGACACATTAACAAAAGGGTAAGAGTCTTCAGAGCAGATTACTCCCACTTGAAAAATGAGTTAAGTGATTTCACAGCGGGAGAGAGGGATATTTGCAGCAAGAAGTTTCATTAGTCACTGAATGAGGTTTCTCTGACATATATTTTCACAGAATGAGAAGCATGATCTTTAGAAGCAAGAGCCATAACCTTTCTATATTTTTCTTCTGTTTATTCATTTTGCTGGAAGATTCCCTTCCCTAGCCTTCTGGAAATTTCAGCCTTCTAGTCTGATTTGGTGACCTTTGTTCACTAGGAAGAACATAGTCCGTTTCTCTTTGCCAAAAGGTAGTTGCATGCATTTGCAATTTAAACAAGGAACATCCAAAAAAATTAGAATGTGTGTTTGTTGAAAATATTGTGATTATTAAAGTCAGAGAAGATAGCTAAAACAGAAGATGCCCATACTTTGAAATCAGATGATTATTAATAGATGCTGCTTTGTGTTGACTGGAGTTTAACTGCCAGTCCTTTCTTTTGCCAAGATATTTTCCCAAAAGAAACATTTCAGTTGTAGGCTCAATAAGGAGACTGGAATCTGCTTTGTGAATTGGTGGCAAAAGGAAAAGGTGGGGAAGGTAGGAGAAGAAAAGAGAGATGGAGCCTTCAGGTAGGAGACTACTTTTTCTTCCTTTGGTGTCTCATCTTAATATTTAAAAAATTAAATTGAAGACTCAGCTAAGGTATAGAAAATATCAGGCTTTTTCTTTTTGACATATAACCAACATTATCTCTTGTCAAGCAATTTATTTTTTTATTTTATTTTTTTAATTTTCTAATAAGACTAGGTTTATTCAGTACCCTAGTAAAAGTTTTTATTATAAGTATCCAACAGTATAAAAAGTACAAAACAGACCTGTAGATTTCTAATATATTAATACAAAGTGCTTATTTTTTAAACTGCTTTTTTTTTTTTTTTTTGAAACGGAGTCTTGCTTTGTCGCCCAGGCTGGAGTGCAGTGGCGCCATCTCAGCTCACTGCAACCTCCATCTCCCGGGTTCAAGCAATTCTCCTGCCTCAGCCTCCTGAGTAGCTGGGATTACAGGCACCCACCACTATGCCTGGCTAATTTTTTTGTATTTTTAGTAGAGATGAGGTTTCACCAAGTTGGCCAGCCTGCTCTCAAACTCCTAAACTCAAGTGATCCACCCACCTCTGCCTCCCAAAGTGCTAGGATTACAGGTACATGTCACCACGCCCAGCTAATTTTTGTACTTTTAGTAGAGACAGGGTTTTACCATGTTGGCCAGGTTGGTCTACATGATGACTTCCTAAACAAGTGCATAACTTCGATTCTACAAAAGATGACAGAATTCATTAGTACTACTCGTTTGTCCTCAGTTATACTTTCTGCAGTTTCAGTTATCTACGGTCAACCATGGTCTGCAGAAAATTCCAGAAATAAACAATGCATCAGTTTTACATTGCCCTTGGTTGTGAGTAGCATGATGAAGTCTCCAGCAGTCCTGCTCCCTCCCAATCCATCCTGCCCAAGAGGTGAATCCTCCCTCTGTCTGGCATTTTCATGCTGTAGAGACTGCCTGACCCTTAGTCACTTAGTAGTCTGCTCAGTGACCAGATCATCTGTCATGGTACTGCAGTGTTTGTTCTCAAGTAACCCTTATTTCAGTTAACAATGGCCCCAAAGTGCAAGAGTAGTGATGCTGGCATAGTGTTATAATTCTTCTATTGTATTATTAGCTATTATTGTTAATTTCCTGTGACTAATTGATAAATTAAGCTTTATCATAGGCATCTATGTATAAGAAAATGCACAGCACATATAAGGTTCAGTACTATCTGTGTTTTCAGGTAACCACTACAGGTCTTGGTACGTGTCCCCCGTGGGTAACGGAGGACTCCTATTGTCTGTGTTTTATTTGAAGGGATTTTGATTCATTTGTGATCTGTTTCACGCCCTCTTCCTTTTCTCCTCTGGCAAATTTGAGTTGGCATGCCCTCCACTTAATCTTTTAAATGCTTGATCCATTCTATTCTGCAGAAGAATGTTAAATTTTTCATTATGTCAGTCAATATGCTTTTGGAAAAAGGGACACTCCTGTTTGTGTTTCCTCTTTAAATTCATGGTTTAGAGTTTTCTCCTCTTCCTTTCGCTTGAGCCTCCCCAACTGCAGTGTCTCCTCAGTCCTCTAACTCCATGACTGTGGATGAAACTCCATCTTGTTTTTCTTCAATGTGCTATTTCTCAAGTTTACATCTACAAATGTGCTGCAAATATCTGGTACTGAATGATGTTTCATTTCAGTGAAGCGTTTGTTTTTGTTTGTTTTGAAAGTTAATTGTGCATGTGGTTTAAAAAATCCAATATAACAAAAGGCATACAGGGACACCATTTGACCATGCCATTCCCCACCCCTTCATTCAGTTGTTTCAGCGACCACCTTTCTTTGTTGTGGCTTGAGAATCCTTCCAGAGACGTGACTAAACAGCCATGGAAATGCCAGTGCAACAGAGCATTCTTTACATCTTGCTTTTTCCACTTAATAACATAACTTTGAGGTTGTCCTATTTTGACACATAGACATCCACCTCATTCTTCAGGAAGCCTCTGTCACAGGCACATATATGGACCTACCATAATTCATTGATTGGACTGCCATGGTTGGACACGAAGATTGTTTCCAAATACTTGCTACCATAAACCCTAGTGCAGTGAAACTTCCTTCACACACCTTTTTTTTTCTTTTTTGAGAGGGAGTCTAGCTATGTCACCCAGGCTGGAGTGCAGTGGCACGATCTCGGCTCACTGCAAGCTCCGCCTCCCGGGTTCACGCCATTCCCCTGCCTCAGCCTCCCGAGTAGCTGGGACTACAGGTGCCCGCCACCACACCCGGCTAATTTTTTTGTATTTTTAGTAGAGACGGGGTTTCGCCGTGGTAGCCAGGATGGTCTCCATCTCCTGACCTTGTGATCTGCCTGCCTTGGCCTCCCAAAGTGCTGGGATTACAGGCATGAGCCCTTCACACACCTTTGAGTGGGGGTAGGATTCCATATCTATTTTAAATGTATATAGATGTTATTGAGTTTTAGAGGACTAAACAATTTAGCTTCCAAGCATAACCTATAAATGCATCTTGGCCACTTTCTTGCCAACAGAGTGTGTTATAAAGCATGTCATTTTTGTCTGTCTCAGGTCAGTGAAACTCCTGTAAAGGACCAGATAGTAAATGTGAGCCACATGGTTTCTGTCCTGACTACTCAAATCTGCCCTTGCAGTGTGAGAGCAGCAATAGATGATTTGTCCATGAGTGGTGTGGCTCTCTTCCAATAAATCTGTATTTACAAAAGGAGGTCCTGGCCAGGTTTGCTTCCTGGATCATAGTTTGCTGACCCCTGGTCTATCTAATAACAACAATAATAATCTTTAGTTTGTTTCTTTTGTATGAGTTAGGCTGTTCATCTGTTTAAAAATCTACTTAGGTATTTTTTTCCTGTTAATTACATCCGTTGCTCATTTTGCATAATGCAGTTTAACTTTCTCTTGTTGGTTTATTAAAAGCAATCTATATATTTGAAACTTAATTACTTTTATATATTCTGAAAAATAATTGATCTGTTAGCTGTTGCAACAGTTGGCTTTCTGATAAATTTCTATTTGACATAGAACCAAGTAAAAATTATGTTACCTTGGGTTGTAACAGTTACTCTTAAAAACATTTAGATCTGCAAGGCACAGTGTCTCATGCCTGTAATCCCAGCACTCTTGAAGCTCCTGGCTTCAAGAGACATCCCCGCCCCCACCCCGCCCCCGCCCCCCACCTTGTCTTCCCAAAGTGTTGGGATTATAGTTGTAAACCAGCAGGCCTGACCTTGTGTAGACATGGTAATTGACAAGAATCTTGTAGTCACATTTTCATAGACTATGCAGTAGATGCAATAGACTAACTTCTGTATGAATCTTTTTCATTTTGTATTAATTATAATCATTTGCCAAGTTTGCTTCATTCATTTGTTTAGTAAAAGAGTATGTGTAAGGAATTTGGTAGGCAATTTTTAGAACTTTTAGTGACAACTTTGTTTTTGATTGTTTCTTAGTGAAAGAAGGATTACAATAAGAACTTAGCCACAAAATACAAGTTTCCATGAGTCACTGCAAAATAACAGGGATAGTTTGGAAAGGCAAGGAGTAACCAGAAGCTTTGGGGCATAGTTTTCCTTAGTTAAATCAGTATAATAAATGGGGTACACATTGCAAATTATTTATTCATAGTTTGGTAGTTTGCATTGGTATGTCTTAAACCTGAATACTTTAGAGTGAATGAAGTAAATAGGATGAGATGATGGGGAATGCACACACACCCACACACATGCACACACAAACACACATGCATGCATGCATACATACATGCACACACACATATACATATGTGTGTGTGCCTGTGTGTGCACATGTGTGTGTATGTATGTTACGTTTACATTATTTCTGCATATTAAACACTTTCCCCTTTCGTTAGATATTCTTTATTGAGAAAATGCACTACACTAGATTACCATTACTTAAAAGTTGCTCTCGCAGCACAAATCAATTCATTATCTTTAAGGATAAGCCCATGTCTGGAGGTAGGGAAATCATTTTTTAAAAATTAAAGTTTCTGTCTTGAAATATTGTCATCCTTCACTTTTTCTATGCACTAGGATGCTCTTTGCTTTCAGGAAAACACGTTATGACTCATTTAATACTGTTGTCCCTCTTATCCAGAACAGAACATACCGTGGTTGCCTAACAGGAAGGCTGCATATAAAACCCAGTTTTGTCTAGTATCATTTTCCCCAAGTCCATTATGTGTGTTATTGTGCAGTGCATGTCCAAATGAGGATTTGAGCAGTAGAGAAGAAATTCATTAAAGAAATGTGTCATCTCCTTGCAAAAAGGAAAGTATTGTTGAGGAAATTGTTACTGATAAGACAAAAGTGGTGAATGAACATCTACCATTTGAAGGCATTTCTCTGAAGTGAAAATTACCTTGAATTGTCTTGGGATCAGTTGTGACTTGATCCTTCTATTAGGAGCTGTTTCAAACTCAGAGAAGGGGTGATGATTCACACTGATGACTGAAGGTTTCTTGGAGCTGGTGTGAATAAGAAGGGAAAAGTATTGCAAATGCATCATTGTGGCTTTCACTGAGACTCAGTGGACAGAATTCATCATGATCTTCCTGGGCTCCAGAAACACAGGCTTGAAATTTAGTAGCCAGTCTGCCAAGCATGGAGTTAGGCACAGATGGGATCTGAGTTAGAGAACTCTCCTGGGACTGGTACCCAGGGAGGGTAATGTAGGGTGAAATGTCATTGTTCAACATGCTTATTATTCACCTGAACATGGGTGACATTCCTTTCCTGAGAAACTCTGGTCTGACAAATGGGTTCTTACAATTATTTCTGAAAATAGAAAATGTATTTCCAATAATTATTAGTTATATCTATTTATTATTTCTAGTCATATTATTCCTAATAATTGAGCTCTATGGCTATTGGGTGAGGTTCCTCAGGGAACAGCGGATTCTCTGTTACTGAAGGAGTTTAAACAGTATCTATACCGAGAGTAGTCAAGACATGCAGAGATGATTTCCATATTATAAGAGAAGTTGGATTGAATTAAGTCTGTGATTCCCTGCCATTCTGAGATTTTAAAAGTCCAGGCCTTTAATGTACCAATTCCCTGTCATCATTAGTCTAATTATTGGCAACTACATTGAATTATACAGTATAGTATCAGTTGATGAATATAGTATCAATTGATTGGTACAACACTGTATCAGGTTGAATTTAACTGAGTTAAGGTATGGCCCTACCTTCTAAGAGCTTACCAGTTGACAATAAAAGCACATGGGTAGGCAAGAGACACCCACATTATTAGATATAACTATGTTATTCATGTTACCTAAAGTTGGAGAGTAAGAAGAATGAATTTCTTGAGGTAGGGATGAAAGTATATCCCCATTCCAACAGTTTAGATCCAGAGAAGAAAAAATGTTTCAGAGAGGAGATATGATTTTAAAAATTGCTTCAGAGGAAAAATTCAGATTGGTAATGGCAGCCTAGAAAGATGCTAAATGAGGAATTCTAAGTCAAAGGCCTTGCAGAAAGCTAGGAATGAACATGTCACTGGTTCTCATGGAAAATGCTTAGAGTCCTGCAGGGAATAAATTCCTTTTTTTTTCTTTTTCTTTTATTATTATACTTTAAGTTCTAGGGTACATGTGCACAACGTGCAGGTTTGTTACATATGTATACATGTGCCATGTTGGTGTGCTGCACCCATTAACTCGTCATTTACATTAGGTTATCTCCTTTTTTTTAAATCATTATTACTATTGTATTTATTTATTTATTTTTTATTATACTTTTATGTTTTAGGGTACATGTGCACAATGTGCAGGTTAGTTACATATGTATACATGTGCCATTTTGGTGTGCTGCACCCAGTAACTCGTCAATTAACATTAGGTATATCTCCAAATGCTATCCCTCCCCCCTCCCCCCACCCCACAACAGGCCCCGGTGTGTGATGTTCCCATTCCTGTGTCCATGTGTTCTCACTGTTCAATTCCCACCTATGAGTGAGAACATGCGGTGTTTGGTTGTTTTTCCTTGTGATAGTTTGCTGAGAATGATGGTTTCCAGCTTCATCCATGTCCCTACAAAGGACACGAACTCATCATTTTTATGGCTGCATAGTATTCCATGGTGTATATGTGCCACATTTTCTTAATCCAGTCTATCATTGTTGGACATTTGGGTTGGTTCCAAGTCTTTGCTATTACGAATAGTGACGCAATAAACATACGTGTGCATGTGTCTTTATAGCAGCATGATTTATAATCCTTTGGGTATATGATCAGTAGTGGGATGGCTGGGTCAAATGGTATTTCTAGTTCTAGATCCCTGAGAAATCGCCACACTGACTTCCACAATGGTTGAACTAGTTTACAGTCCCGCCAACAGTGTAAAAGCATTCCTATTTCTCCACATCCTCTCCAGCACCCGTTGTTTCCTGACTTTTTAATGATTGCCATTCTAACTGGTGTGACATGGTATCTCATTGTGGTTTTGATTTGCATTTCTCTGGTGGCCAGTGATGATGAGCATTTTTTCATGTGTCTTTTGGCTGCATAAATGTCTTCTTTTGAGAAGTGTCTGTTCATATCCTTTGCCCACTTTTTGATGGGGTTGTTTTGTTTTTTCTTGGAAATTTGTTGGAGTTAATTGTAGATTCTGGATGTTAGCCCTTTGTCAGATGAGTAGATTGCAAAAATTTTCTCCCATTTTGTAGGTTGCCTGTTCACTCTGATGGTAGTTTCTTTTGCTGTGCAGAAGCTCTTTAGTTTAATTAGATCCCATTTGTCAATTTTGGCTTTTGTTGCCATTGCTTTTGGTGTTTTAGACATGAAGTCCTTGCCCATGCCTATGTCCTGAATGGTATTGCCTAGGTTTTCTTCTAGGGTTTTTATGGTTTTAGGTCTAACATTTAAGTCTTTAATCCATTTTGAATTAATTTTTGTGTAAGGTGTAAGGAAGTTGAGACTGGTAGAAGACTAAGCTTCTTCCAGACTTTAATCATTGTTATCTGGAAAGGAATTGAAAATAGTTTTTTTCTGAATCATTGTAATCATGTGAAATCACTAAATGTCAGTGTTGAATTGACCACAAGGACCAAGCTAATTATGGAAGAAATAGGTGGGGGAGACATTGAACACAGCAATCCACAGGAGTTTGAGTAAGTCTGGAGTGTTGAACTGGTGAAAGTCCTCCCTGCAACAGCTCCATCGGGGCAATTCTGTTAAGTCAAGACTCAAGCACTGGACGGTGAATGGTCCAGAAAAACTATGTCATTAAAAATGCACATTTGTTTAAAATAACTAACTGCTCTTTCGTGGATGATTGGTACTAAGATTTTATAAACTGTTTAGGGACCACCATGATTCCTCACACACATTAATTAATTCATGAGAGTTGATTTTCTTTTCAAACACATTGATACATTATTAGTAGATAGCACCCCAACACACACACACACACACACACACACACACACACACACACACACACACACAGAGAGAGAGAGAGAGAGAGGGGTACTTACAATCAAAGACAGCCATACTAGATCCAATTGGTAGCAACAAAGTGAGAAAAGTACCAGAACACACAGGCAAATTGAAAATACACAAAGCCACATCCACAGCATGCCCTTTAATGGAGGAAGTGGGAAGAAGGTTCCATTTTCCACTCTGCTCATTTTCTTCCCCACCACCCATTAAGAGTGTCAATTCTCATTCACATTCCTTTTAGAGAAGAACGAACCATCGAAAAGGGAGCTGAGAGTTGTAATAAAAATATTGCATTACGGATTTCTCCAGTTTCCTTTCAGTATGAAGTATTTGTTACTTCATTGAAAAAAGTAGAAGTATTGATCAGCCGCTTAGCTTGTGGCTTCTGCTCTCAAGGAGTCAGCACATAGTCTGATGTGGAGGAAAATCTATAAATGGATTTCTGCAATCTGCAGGTAAGCATGGGATGAAATGTTCCTTGACATCCAACCCAGGTTAGAAATCAGTTTTCAAGACTCTAAATTTGAGGACCCCTAGGAGCTCAAATGATAAAGAGAAGAAGGTTTATAGTCCATGATGGGGGAGGGACTGCACACTACCTGCAGGGTGAGCAGAAAGGATGCAGGGGCTTGGTATCACAGGACCAGCATTGTAAATATTACAGGAAGTAACCTTTCCTGTGTGTCCTTCATGTGCTTTTCTTTGTGCATATTCTTGAGGCTTAAAGGAAAGGGAGCCAGTCTGTGTCCATACTTCTCTCCCGTGCACATCATCCCGGCATGGCACTGCTGATGCAAATTAAAAAAATAACCTTTGACTAGAAGCATTTTCCCAGCTACCAGTTTCCTTCTCCCCAGTGCAAGACAATGTGACAGCAAAGGTTCATGCACAGAAGCAGAAAGGTAGTGGAATGACTCAGCTTCTAACTAAATTCCTTCCACCTTCCTTAGCTTTGTGGTCTCAGGATTTTATAAGAGGTCTCTCATGTGCTGCTACAGAACCAGCAGGAAAAATCAGACAGGGCCAAGACAGAGAGAAAAGAGACACCTTTCTCCTATATTGCCCCTACCTAGGGCTCCTATCCAAAGCATGTTCTAGTTCCTAGATGGTTGATTCCAATAAAATAACATAAAAATAAACTGTGCAATAAAAATTTAAAGGGAGTTGCGCTGACCATCATTTTTGAAATATTTAAAAATGAGTCCTCAGTAAATTTTGGTGTGAACATTAGTATTTTGTCATGGATAGAGGCACAAGAAAGGAGTAAATGTGAGACCTACATTGCATCCAATGCCTGCATCAGTAGAATCTAATCTCTTCCCCCCATGATAAAATGGCCTCATTCTGTCAACTACAGGCTTTGCTAGCTTTTTCTCAGACAACAGACCAAATTTATCCCCAGCCTGATAAGGATCTTTATTGCATTTGCTCCCACCCCACCTACTGTATTTAGGGTAATGGTGAAAAATGTACATTGATGCTGAATTTTATAGAAATAGTAGAAATGGAAATGATCTTACAGAGTTGTCATCTACTATCTGGTGTAGGTTTGGTTACAAAGCTGTATTTCCTCTTCCAAGTTTTAAGTAATCAAGTTTCAAAACAATCTTTCCTGACATCCAGTTTGTGTTAAAGCCAATTTCCCAAATGATTTTCATTTGCATTCTGGAAATGCAGTGAAGCCTTGACATTTTACAAAATGACCTATCTTCTACTCAAGTCAATGAAACTACAGTAAACATTTTATGTGTAGTTGCAATGCTTGTATCTCCCTCAAGATTAAACACAGAAAAGCATCTTTGGGGAGGATATTTAAATACGATATTAAAGCATATAACATGTGTCTGTATTTTTTCAGTTTTAAGTATACTTACTAATAATAACAGGCAAAGTGGTACGAGGTAAAACACTACTTTTCATTGTTCAGTTTACAGTAGTCATTGACTATTCTACATATGCGCTTAGCATAATATTTACAGACTATGTAATACAAATCACACTCTGTGAATTCTCATGTCCTGTGAGACACAGGAACAGAAGAGCTTTGTAAAAAAACAGCAAAGTACAACTTGAAAAGTTAAGCCATATGAGTAAGAAATCAAAGTGATGAATTTACTAAGTGTTTATTAATATTTAAGCTAAGTTTACACATGACTCAACATCATATTCATACTCATAGTCTGTTACTGTACTTTGCCAAACTGTCTGTACTATTTTGTGAGAGGATATTATCTTTAATATTGCTCTCACTGCAATGAAGCATAAATAAAGTATATGTCATGTTCTACCTTTTCAGGAGCTCCAATGAACACATGCTATGGTTTTTAATGACTGTAAAGAAAATTTCAAAGCCATATCTTATCTGTTTCTATGGAGAAGTTGATCAATGATCAATACCATTTGCAAGGACCCCGATGTGTGACTTGTTTCTCTTTATACTGTGACATGTTTCCCTGAAGGTGGAACGTCAATGAGACATTCATTTTCTACTAAATGAAAATGATGTTAAAGTTGCAGTCTAGTGATAAAGTTACCAAGATCTGCTTCTTGGATTTTTTATGGGGTTTGGGCAACACATAAAGAAACTTTCCTCTCATTCAAGTTGAACATATCCAACCACTTATATATATGTTGCCCAGTGAGGTCAGTGTTACATGAAGTTGTAGAACATTTACTTTGAAATGAGGTTTTCTCATTTAATAAAAGTGTCACCTTGTGTCAGTGGCTTAGCTAGTTCCAGCTTCTATTTTATCTCTTATCCAATGAGAATATGCCTATCACATAAGGAGTGTGGCTGGGAAGAATGGTGGTCTGTCCTTATCTCCTGGGTTCTCTGGTTTCAGAACCTGCACAGCGGACAGTTCCAAACACTGCATTCCACCATCATTTCATCAGCATTCCTCTTGGAATAAATGTGTCTTGACAGTCTCTCTTAGAAGTGCTTTCTCTGAAGCTACTGAGGACCATGCCATGTGTAGGCATAACTGAAGCGTGCACATTCTATAGAGTGCCTCGAAGATGTGCACATTCTATAGAGTGCCTCCAAGGTTTTCAAGAAGAATGGAGCCCAACTTGGCCACATTGGTTACACACTTGTGCATGGTCCATTTATTGACTATCCCACCTTCCAAGTAATTTACCTGCACCCGACTTCTTGTCTCATGTGGGGCCTTTAGAGTAACTCCAAATAAGACCAGGTGGATGTGCAGATGAAACGTTTGATGCTTGCATGTGCTTGCCTGATTATGACTGTTAATCACCAGGTGTGTCAAACTACTCTAGATGCTCATTGTGTGTGTATGACAGGTTTTGGTGCTCTTTCTGCTTTTGATAAGCCATTCAATTTAATAGGGTGTTCTCTGAATGCCCAGCTTTTCTTTAAACTTAGCATGTATATTCACTACCCCACGATCCACCTAAGACAGTTGCGTATCATTTCTTTATGCCTGTTCCGTGTTCTATGTATATTAGATGATTTCATATAGATAAGGAGGGAAAGCTCATATTTTATACATTTTAACTATTATGATGAAAACCTTATCTAGAAGAGGTTCTCTTCTTTTTGAAGTTGCATAGCATTAGTAAAGCTATAGGAGCTATCTCTTGTATCTGACTAGAAACGATACACATTTAAGATAAAAAGCATGGGCCAGGTGGTGGCATATGCCTGTAATCCCAGTACTTTTGGAGGCCAAGGCAGGAGGATCATTTGAGGCCAGGAGTTCAAGACTAGCTTGGACCACATAGCAAGCCCTCCCTCCCCACCCTGTCTCTACAAAAAGTGAAAAAATTAGCCAGTCATGGTGGCATGTGCCTATAGTCACAGCTGCTCGAGAGGCTAAGTTGGGAGGATTGCTGGAGTCCAGGAGTTCAAAGATACGCTGAGCTATGATCATGCCACTGCAGTTCAGCCTGGGTGACAGAGTGAGACCATGTTTCAGAAAACAAGTGAGTAAAATAAAATAAAAAGCAATAACAAGATTGCATTATGCTTTGAGGGCATTAATTTTCAAATTTAACTTTACTTGCATTTTTTTCCTGTCATTCTTTCTGTGTCGGCTAGTTCTTATTTTAGTTGTAATCTTTTTTTAGAATACTTATGAATAGAATAAATACCACTGTATTCACATAGTATATTTACTATTATTTTTGTCTCCTTGCATTGTATTTTAATTATCTATGTCAGACACTTTCCTCAGTCAAATGTACTACTAGCCATCTAAATGGAGAATTTATCTTAGGAGGAGAATTCTTCTCATTTATTTTTGCATACCCAGCAAATTATTCGGGAGTGAGTGCACTGTTTCATCCTGTTGATAGTCTTCCCTGAACATTTATAACCCACCCCTGACTGGCTCCAGTCTTTACACCTTCCTCAAGACCTAACTTAAATACACTGAACTGCCTGAAGTCGTCTTTGAATTTTACATCCTTTCTCTTAACTCTCATACACTTTGCATTGTTTTCCCATACAGGGGCATCAAGAAATAGACCATATTATAATGAATGTACAATAAAGTACTAAGAGTAATAAAAGTAAATATATTCCGAAGCAGGAAAGAGCAAATGCTTGGGTTTTTTATAGAAGGAGAGAAACGATAATTTGAGAATGTTTCATGGAAACTCTTGCATTTGAGCAGAACTTTACAAATTAGGCTTAGGCTTCAATAGTTAAAAATTAGTGAAGAGAACATCTCTGCAAAGTTGAATGTTCTGGTCTCCTTTCTGTTTGTTTAGTGAGCAGAATTGATAATCGACATGCAAGTGGCTTTTAAACTTTTCCAAGGACCAGTCATTGGGGAATTAGTGTGGTTCCTCTGAACCTTTCTAGTAATCCCAGGATTTGAGTATTAAGAACAGTTAGTTGTGTTAGCCTTAAGATGAAATTCTCCTACCTTGTTGTTTTGAAGATGTTACTTAGAGGGAAGGAGATGTTTTGGTCTGTTCGGGCTGCTAATACATCTTTTTCTTCTCAAATTTTACTTTAAGCAGTCAGGAGGAACCAAGCCATTCCTTCAACACTTTTCTTAGAAATAGCTTCAGCTAAATCTACTTTTATCACTCACACGATCTGCCTTCCACAAATTACTAAAACATGAACACAGTTCAGCCAAGTTCTTTGCCACTTTGTAGCAAAGATCACCTTTCTTTCATTGTGCAATGGCATATTTCTCATTTGCCTCTGACAGCTCATAAAAATGGAGCTTCCTGTCCATATTTCTAGTGTCATTCTGTTCAAAATTGCATAGATTTTCCCTAAGATGATTGAGGCTTTCTGTACAGCTCTTCTCTTTTTTTTTCTGAGCCCTCCCCTCACTAGAATCACCTTCAAAGGTCTATTCATGGCAACGTAGGCTGTGTCTAGCATACACTTCAAAACTTTTCTGGCTTCTACTTATTACCCAGTTCCAGAGCTGCTTCTGCATTTTTAGGTATTTGTTATCTTAACACCACACTCTCAGTACCAATTTCTGTCTTAGTCCACTCAGACTGCTATAACAAAATACCATAGTCTGGGGGTGGGGGTGGGGGGGGGTAATAAACAACAGACATTTATTTCTCACAGTTCTGGAGGCTGGAAGCCCAAGATCAAGGCAGCAGAAGATTCAGTCTCTGTTGACAACCCACTTCCTGGTCCACAGACAGTGACTTCTCCCTGTGTCCTCACATGGAAAAAGGGTGAGGGAGCTCTTTGAGATCTTTTCTTGAAGGACACTAACCTCATTCACGAGTACTCCATCCTCATGATCTAACAACCTCTTAAAGATGCCACCTCCTAATACCATCTCCTGGGGGAAGGGAGTTTAGGATTTCAAGGTTGAATTTTGGGAGAATGCCAACATTCAGCCCATAAAAGGAGATAGTATAGGAAAACTACAGAAATCAATAAACTCTTCTACTGTTTTGATTAAAATATAGCAAGTGCATTTTTGGTGTACATATTTTACTTTATCTTTGTTATTATTCATCTAGAAAACAAACGTACATAGTGATAGTTAATTCTTCCATGACTTTTTTGCAAAAGTGTTGGTATGCATTGGCTATAAGTCTCCTCTCTGACTTCATAAGACCTTGGAAAGCTGCCAAATATCTCAGAACTTGTTGTCTTGAGTCTTAAAGTGACTAAAATGACCTTAGCTCTACCTGCCTTATAGGATGCTCTGCCCAATGATGCATGCAGTATGCATGTTCTTTAACAGAGTATGTTTTGAGACTGCAGGTTTAGGCGTTATTAGAATCCATTTGACTCCATAGCCCTTTTTATGGAAACATACATACATACTTAATGTCAAATAGTTTATATCTTTTTACTAGCTAATATGGATAAGTACTGTCTCTTCCCATTTGACTGTGTGTAACTGCCTTCTCTTAGAACTCAACACAAAATGAGCTTTATGATTCACATTTACAGTAACATGGAGACAGAACCACCTCATTCAAAACAGGAAAAAGCAGGTATAAGATGCCATGAAGGGAAATGAGACTGAATGTGTTCAATTTTTCTTTGTTTGGCTTATCACATATCGTAGAGAGATGTCCTCTTACATGCAGTAGAAATAAGAACATCCTTGAAAACTCGGTTTGAGCAGTTCAAAATCATATATTTTTTAATGTTGTATGAGTTTCAGGTGATAAATCCTCTTCAGGATACCTCAGGGGTTCGCAAAAATGTAAAAATATGTTTAAAGTTTGAAATGACTCACATTTTTTAGTATCCACGGCAAAGAACTGCTTTTCCAACCTTAATAGGATTTCAAATTGACATTGACATTTTAGTAAATCAGAATTAGCTTTTTCTTTTTAAGCTCCTGTGTCTTATGTAAATGGCTGTGCTGACTTTTATGGAATTGAATATTCCAGAAAATGTCATGGAACCTAATATAAAACAAGTTAACATTCTCATTTTTAGATCTTAAAGGGATATGGTGTTAAAATATAGCTTTTGATACCCATCCAACCTGTGCAAGGTTTTCTGTGTATATGCGAATTTCAAATTTGAGAACTTAGCATGTCGATGAAGGCAAATCTATATACCTGTTGAAAACAAAATTGAAATTCTGAAGGAATTATTGTAATTTACTTAAATAAGAACTGTAAGAAGTCAGACTGTTAATGGAGTGTCAATAGATTTCTTCTGAGAGCTTCAAAATCTTTTCACTGCCTTTATTACAAGTCTACCAAAATATCTGTTAGATTCTGAAAGCCAATCTCTCATTACAAAAAGCATTATTCACAATTTTAACTTATTTCCACAATGAACATTCTACAGAATTATTGTATCTTTGTTTAAAGATAAAAAATTCTCCCTCGGGAGGCTGAGGCAGGAGAATGGCGTGAACCCGGGAAGGCGGAGCTTGCAGTGAGCCGAGATCGCGCCACTGCACTCCAGCCTGGGCGACAGAGGGAGACTCCGTCTCAAATAAATAAATAAATAAATAAAAATAAAATAAAGTAAATAAATAAGTAAATGAATAAATAAATTCTCCCCCCGAGGTCTGAAATTTATTATTAATGTGAATATTTTAAGCATTTTTAGAAGAAAATAATTTTGTAAAAAATATTGTAAGTTATGGAAAATATGGTGGTGAAGTATAACATTCACGAACTTGCTAGAACCTTGCCCTAAAAATGAACTAATTATTGGATCATATGGCAAACTGATTAAGAAGAATAAGGAACTACTTTATATCATGAAAAAATACATGACTATCCACCTGCCTTCCTAAAACTTCTTCCTCTCATGTGCCGCTATTTTACTTAGAGTTTTCTTTCGGGTTAAGGAACAATATCTTTAGAAGGCTATTCATTAAAGTACTAATTAGAAAAGGTAGTTAATTAAGCTTGTCACACACAATTTATATATTTTCTTATGATGTGTAAGAGAAAACAGCATAAAAAAGATAAATTATTTATTTTCAGTCAAAATAGGGCACTTTTTTTGCTTTCCTGCAGCTCATTATACCTAAATTCCTTTGTGAAAGTATTTAAGTAAGTTCTTTGAAATATTGCTTTTAAAATATGTTTACTCTTTAAAGTTTTAAAAATAAGGAAATGTATAATATAGTGAAATTTCCCCATCAGTGTGTTCTGTGTATTTTCTCCAGCTCTTTCTTGAATTACAAACAGCAGTTCTACAACTTTACCACCCACACACACACATTTATTCATTTGCACATATTTCTTTTTAGTGTTTTTTTTTTTTTGCAAAATTGGCATCATATTAATTATACTACTCTGCAACTTGCTTTATTTACTGTTTTTAATATGGAAATTGACTGAAGTTAATTTTCAAGCAGTTGTGTAATATTGATTGAACTTAATTGATATACTATAACTGATTAAACTACCTCACTGTTATTTGGAACACTTATCGACAACACTGCAGTGTAAAACCCTCTTTCTACTTTTGCAGCTTTATGATAATTCTATAAATAATCAGACACCGATTGTGATGCAATCGTATCACAAATTCAAAGACACATTATAATGTCAGTGGAATAAGTTAGACATACAGTGCCAATTAACTCAGGGTTCCAGGGGTAATTCTTTTCGTATTGATGAAACGCAAATGCATCTTACTCATTCAGAGTTGCCAGGGCCCTGGTGTAGAAATCTAAATCATAACCAAAACAAACAGCATCACCACGAAGAAATCAACAAAAACAATTTCATGAGGGTTTTGAGTATTTGAATAATATTTCAGTAATTAAATTTTAAAGCAAGAACTGACAGGTTTGCCCACCCCATCCATCCTGTGATGTCAAATGCACGGTATGTATCTGGCTGACAGGGAAATTGAGGTAGGAAAATAGAATAGATAATATGCTATTATGTACCTGCGCTTCAGTTTGAGGAGGATAAAATTGTTTTAACCTTATGTCCACATTCCTGGAGTGGTTTGCTAGACCTGCATCAGAAAATCCACATCTTAGTTCTTCAGCTGTTCACATCTCAATCCACACAGCCTTTTGTCATTAGCATGCCAGAAATGCACTACATTCATGAAAGGAATTACTAGTTACATCATGGTGAATGTTAGCATGAACTCTCATTGGCCCATAACATTAAAATATTCAAAACATACAAATTGGCTAAAATCGTTTAGAGAAAATGTTCACAATGGCATGATGAAGGTATAAAAATCCAGAAATGCCTATGCCTTTGACCTGCTCCAGTGCCCATAACTTGAAGTCTCTTTAGTCCTACGCTCAGCCATGGACTAAGGAAAATTTCTCATTACCTGATGCTGACTGAGAAAGATAAAAGAACACCACTTGTTTTGTCCTTAAAGACTTGAGAGGCAAAGAGCTACATGATAGAAGTTGTACCTCTCACAAGTTTATGGAAGGAGACATATGAACTGTTTTCTGTCTGCTGTGGAAGTCAGATGAATGACTGCCTATATGTGTAACACATTTGGGCCTGAGACACACATGATGAGGGGAGGAATTACAAACTATCACTGGTCTCCTTCTTTTTCTGCGATTACTGTTACCTTACCTAACAGTAGGTAACTGTAATCTAAAATGAACCTAAAAATTGTGCATGAACAAATTAGCTCAGGTAGCTTGCAACATTGACTTTACAGTTTGACCTAGGGGAGCCCCACGGGCTGAACCTAATGAAACTCAGCCAGGTTATATTAAAACTGCGATAGCCTGTATCTCTACATTTTCTGCAACCTGGTTTCTACATAGGGAAATGCTGCTTGTGTTTGCTGTAGGCAAATCTTAAATAAACCATGACTCAGCAAGAAGAAGAGAATGATGTGCAGAGATATTTTAGGGAAGGGATAAGATGGCAGTTTTGAATGGGAGCCCACATGGTACAAGTACTCATATTCCATTACCAACTTCAGGAGCTTTTTACTTTGGAAAACCATTTTTCACCTTATTTCAGTAATATGTCAAGCATTTCAGGTGGTCTGCAAAAGCCACATAGCTCAGAGGCTTAGCAAACCTCCTCAGACATCAGGCAGAAACACTTTCTAAACCCCTTAATGAGTGTCAAGCAGGAAATTGTGAGTATATAGTATTAAGGAGATGGACTTGCTATTCTTAAATTTACAGAAAAAAATTCTGGATTTTCTTCCTCAGTCTCCACTTAATGACAGATTTTTTTTTAACAAAAAGATGCATGACAGTACCTATTTAAACTTACTCTGATAAATTTGATGAAATATTCTTTTTTTAATCCAGACATCTCTATGAGTTTCAGAATTATTACCCTTGTCAAATTCATCTATGCTTTTTTTGTGGAAATGTTCAACTTTTGTTCTCACTGCTCCCTGCCTTCCCCCATCAACAAACCCTGAATATCTGGGAATTTCTCACCAGCTATTATTTAACTCCATTCCACATGTCCATCAGATGTCCTACACAAGATTGGTTAAATAGAAGTTTGTTCGCTGGGAGAAGATGACAACTTTTTATATTAAATGCATAAAAATTTTCTCAATACTGCAGGGTGATAAAGACAAAGAAAAGGCCAATTTAAAAGGAAGTCTTTAGAAAAAATACAATAAAGCAGAAATGCTTCACTTTCCTACACAATAGGGAAAAAATTTTAATGCTTTTGCAAAAATTAAACTCTAATGATGGAACAAAGTTTATTTTATACTGGGTAAATTTATGTTAGGCATGAAACTACATAAAAATATGTGGACAACAAAGAGTGATTCAGGGCTGCTTAATCGCTGTTGCTCTTGGTGTGGTTTTTAGGGGATTGCATAATTGGTGAGTTCCTTACACGTTGATTTCTCAGATTCACCAGGCAATACATACCAGCTGTCTTGGTAAATGCATGAAATGTTGCAATCTTTGCAAGTCCTGCAATTTTACTTCACCAGTAACTTTCCCTGGTCAACTAACAGTATCTAGAGATCAGGCAGAGGGTGACCAATGGCTGCTCTGACGTACACATGGAGATACTGAAAGATGTGGAGTTAAGGATATTTGAATAAATATTTCATATAATGACAACTGTCTTTGTTAGCAAGCAGAAATATCCACTGTGATGCAAAGGCATATCCTTATGTCATATATATTTGCTGTGAAAGGTACTGATTCGTGCTTATGTGAAAACCTCTTAAATCCCGAATCTGGGGTCTCCTCTCCCCGTTTTTTCTGGAACTCAGATGCTAAAGTTGATACAGGAGGAGTGGACTGTCCCAAATAAAGCAGTCGGGGAAAGGAGGATCCATTGCAAATAAAGGGTAAAAAAGGTACATATGAATAGTATATCTATTTGCACGTAATGCAGGTTATTCTGGAGGGTATTAAATATCTATCAGTAACTATCATTTGTTAAAAACCAGGGATTCCCAAGGATGTTAGTGGATGTATGAGAAAGAGTTTCTGGAGATATATGTTTGGGTGTCCACTACGATTGTTGCATTTCTTTTCTTCTTTGTCTCTCTCTGTCTCTGACTGTCTCTCTCTCTCAGTTTGCTTCTCTTTCCCTTTAAACACACACAAACACACACACACACACACACAGACACCACACAGAATATTCCCAACTTCTTAACACACAACACCAATAAAAAATGCCAATAATCAGATTGTAAAACTGGCAGTTCTTTTCTTTCAATGTGGCTTTCTATTCTATTGTCTCTCACATATCAAAGAAACAAGAGGACAACAGATCAGGATACATTTTGTCATGTTTACATTATGTAGTAACCTGAAACAAATGCCCAGTGAGTGGAGGGTTTCTTAGCTTTCTGTCAGTTTTCAAATGTTTTCCCTCCTCCTGCCTCCCTGGCTTTGGGTTGGTGATGCACGTGCTGGTGCTCAGAGATGCCGTGCGCCCTGACAAGAGATTTTGAACTGGGGCATAGATGATTGTCCCCAAAGTGATCTGCTCAGTTCCCATAATTCTACACATTTCAGGCAATGGAAACACAATGAGAGAGATAGTTTGGGTGGTTTTTGGATTGCAAACTTAGGCAGCCACAGTTTCAACCAGCAATACTGATTTTTCTCAGCCTTTCCATTTCTACCCAGTGCATAACTTATATAAATTTTCTTCCAAAACTTCACAATTAAACTATTCCTTATTTTATGAAGTTATCAATGTGTGTATGTCTTAGAATATAATTGGTGTCATACAAACCAGTTTATGCCTCTTTAACTTTAGTGCTATGATCTTAAAAATTTTGACTCCCAGGCAAATATAGATATAAATATAAATATACATGCATTTTTTTCTTGAGAGTCAAAATTATATATTTATATATATGTGTGTATATTATATATATGTGTGTATATACACACATATAGATTAAATATATATATTTCATATTATATATTACAGATTAAATATATATTATCTATATTTAATTTCATTAGTCATATTGTTTTCTACAGTTTGATTTCCAGTTTTGCAGGACTTTGTATTCATATTCCTGATATCAGGAAAGGGTGCATATTGACACTACAGCTCAGGTGGAATATTTAGAAGACACATGGTTGTAATTAGTTACTTGCATTTTTCCTGAATGCTTTTTATGGTGTTGACTGTTTAAGAATATCTTGCATTGCTTTCCAAACAAATATACTACACAAGCAGCATTTCTTGAATCTCGTTGATCTGTGTGGTGTGTTGGTGTGGTCTTATACAGGATTTTGTCTTTTTTTTTTTTTTAGTGTGGTTGTTTCTCCTTTTTTCCTTTAATCTAACAAATATTGAAGTACTTTAAAATTTTTAATACTGGTTTTTATGGAGAATGAGAGTTTCCTATCATTTTCCTGGGGTAATGTCATACAATGCATTTCTGAAAAAAAAATACTTCTTAAATTTTGTTAATGTTCTGATTATTTTTCTGTCATTATTTTGCCACTTTGTATTATGTTACATTACTATTCCATAACCTCCTTTGATTCCAGCATTGGGAATTGGTTTTCATTTCCATGGACTCATTACTGAGGTCCTTGTTTCTTTCGAGATATTAAACCTGACCCTGAATTTTTTTTCTTCCCTGTGAGAGTGGAAATTATAATTCTTTTCTACTGGTTCAGGAAAAAAAGAAACTTTACTTTCTAAAGAATATATTTCTTTTTATGGTCAGATACGTTTTAAATAAAACGAAAGCTTTCAATATCTGTCTGTAAAAGAGCAGGGTTTGGAATTCTCATTGGTGATGGATATGTTTATTTTCTTACCTGACACGTCAGCTACTGCAGCTAAAGCCAGTGAACTATTTCTATATCACTTACTGATGAAGAAATAAAGGGCTCTCTCATGATACTAAGTGTATTGCTGTTCCACCATCCGGATATTTTTGGCTTAAACCCTGAGGTGTTACCAGATGGTAAGGATTTTAGAAATGCTAAAATGATAATAGTAGGGACTACTTTCGATATTGTGAAGTCAGATATATCATTGCAAGTTTTAAAAAAATGGAATATTTTATATTTTTAAGTATCTGATTTACCTTAATAAACACTTTCATCAATTTCAAGAGCATCTACATGCTACATTCTGGTCCTGAATTTTCATGGTTAAAATAAAGCCCCACCCAGAGACTAGCTAATAACTATGGTGATCAACAGTGGACAGAAATTCAGAGATACTAGTTATGGTAACATCCTTTAATGCTGGAGCCTTACTGTCATAGAAACATGTGAATGTCAAACTAAAAGTTTAAAAGCCAGATATTTCAAAAGAGTGGGGAGTGGGAGAGTATAAATTACCCCCAAGGACCCTGGAAGTGCTAGATTCTGGGCAAGATCCAGATATTTGCAATTTGTTTAACTCCCAGTTGACCATCTGAGAAATATTGAGCAAGAGAGACAGAGAGAGAGAGAGAGAGAGAGAGAGAGAGACAGAGACAGAGAGAGACAGAGACAGAGACAGAGACAGAGATTGCCAGGGACCAAGGGATGATGCTAGTGAACCATTTAGCTACAAAGTGTCAATGTATGAGGCTGGCGTGGTGGCTCATCCCTGTAATCCCAGCACTTTTGGGAGGTCGAGGCAGGAGGATTACTTGAGCCCAGGACTTTGAGACCAGCCTGGGCAACATAGTGAGACCTCATCTCTTAAAAAAAAAAAAAAAAAAAAAAAAAGTTAGCCAAGCATGCTGGTGCCTGCCTGTAGTCCCAGCTACTTGAGAGGCTGAGGCTGGAGGATCATTGAGTCCTGCAGTTGGAGGCTGAAATGAGCTGTGATTGCACCACTGCACTCCAGCCTGGGTGACAGAACAAGACCCTGTCTCTAAATAAATAAATAAGTACTATGTATATGCTGACTCTCCAGCCTTGCCTAGTCCCCAGAAGCCTTGCAACCTTCCAAAACTTGATTGTTTTTCTCCTAAATTTCTCAGATAATTGAGGGGAAAATAGAGCTCAGAATTTGACAACAGCTGTCCACATCTCCTGGAATCCCTGGCAGAATGCTGGTGCTGTCTCTTCTCTGGGTTTCACAGGGCGGGCATAAATTATAACTTTATTAGGTTGAGCACATATGGCCTTTAGCCCCAGGAGACCCTCCATGGGGCTAGTCTGTTGGCAGAGGCAGCTTCTGCACTTTCATTCAAATTCACAATCCATAAGGAAAAAGAGGCCTTCAAGGCTGCAGCCTGCCTTGGGCTTCCGTGGGGCATCTCCTATCATTGCCAATAATGCTGTGGTGAAACCCAGGCCAAATATTCCAACATCTTTTTGCTGCTTGTATGAACACGATGCATATTGCAGTTCAAAACTAGGAAAAAAGAAGAGCATATTACAGGCGAACACGAATGCATCAGAATATGGTACCTTTAAATTAAAAGAGAAGGCTCTTGATTTTGAATTCTCAAGTGTTTCTCTTCAAATACACACAATGATGTCTTTCACTTTAATTTTAACTATTATGGATACATAATAGATGTATATATGTATGGGGCACATGCAGTGTTTTCCTACAGGCATACAATGTGTAATAATCAAGTTAGGGTAATTGGGGCATTCATCACCTCAAGTATTTATCCCTTCTCTGTGTTAAGAACATTCCAAATCCACTCTTTAGTTATTTTAAAATATACAACAGATTATTTTTGACTATAGTCACTCTGGTGTGCTATCAAATAGTAGATTTTTTTTTCGAGGCAGGGTCTTGCTTTGTTACCCAGGCTGGAGTGCAGTTTTGTGATGATAGCTCACTGCCGCCTCAATCTCCTGGGCTCAAGCAATCCTCCCACCTCAGCCTCCTGAGTAGCTGAGGCCACAGGCACATGCTACCACAGCTGGCTAATTATTATTTTTTTAATTTTGTGTAGATTAGGTCTCACTGTGTTGCCCAGGCTGGTCTCAAACTCCCGAGCTCAAATGATCCCCCTGCCTTGTCCTCCCACAGTGCAACGATTACAGGTGTGAACCTGTGCCCGGCTGATAGGAATTTTTGATGGAGTTTCCCAATATCTGGGCTTTCAAAGATTTTGGATAGTGAACGAGATACTGCAAAGATCTCTCTAAATATCACCAGCCTGACCAGGGACCTTGTGTTACCTATATGAATACACTGAGGTTGCTGTCTGTTTCTCTGTTAATGTATAAGCAGAGAAAGTTACATTGATGCTCATCAGATTTTCAGTTTAATATCAGAGCATTGCAAATTAAAATATAAGGTGCGGGACATGTACAATTTTACTGCGGGGCATGCAAAACCTGAGGGCCCCCAAAGCAGAAGAAGGCATTCGGCCTCTAGTCTGCATTTCCTCCCTCCTGAGTTGCCAGCCAGCCAGCCAGCCTGTCTTACAGATTCCAGACTTGCCAGCTCCCACATTGCATGAGCCAATTCCTTAAAATAGATCAATTTAATAAATTTAACCTATATTGGTGAACAAATTTAGCAGAGAACTTTGATATACATTAGTACCACTTATTATTTTTAGAAAAATTGGAATTCGAATAACTAACACTAAAGTCTAATTCGTCATCTGGTGTGTATGTTATAAATGCACACCCACTCACCGAGACCTATTCACAGCCACAGCCTCATATAAAAATAGGCAATAGATACAGGAAATGAGAAGCAGCCATAGAGGGTCTTACGTAAGAAACCCCATCCTTCTCACACCTACTCAAGAACGTTGTTCCCAACATCTACATCTTTTGTAGTTTATATCCACTGGGCGCACCTAACATCACATCCACATTCTTTTGTTTATCCCGTTTGGAAATACGTGCCTGACCTTCACTTTCTCTGCCTGATGTGGCTGCATGTTTTTGTTTCTCTGGCAACCATCTCCTCGTCTTCCAAGAGTCCTCACCGATCACATCTCAACTCCTCTCCACCTATCCTTTCTTAAATTCACTCCAATCAGTATATAGCCTCACCACTTCACCAGACTCCTCTTGCCAATTATACCATTGCATCCTAGGCCCCACAAAAGTGGAGTTGCTATTCCTAATGTTTCTAAAAAATGGCCATTCTGCATTTTCCCTCGAATCTCCACTGCCTCTATTTTTGGAAAAGAGTTTCATCTTTGAAAAAGCATTTAAGACCAACTTTTTTCCCACTCTGGAGGGAAATGAAATATTGCTGAATGCAGAGGATATCTCCAAGGCTTCATACTACTTGCTCTGGCAATATTTCCAGATCCTTATCCTGCAGCATTTGCGGTAGTTGTCCCCCTAGAAATCATAGTTGAAACCTACTCCTCAACTGTGTAGGTATTTGGAAGTGGGGCTTTGGAAGGTATTTGGAGAGTGGAGCCTCATGAGTGGAATTCCTACCATTATAAAAGGGACCCCAGAGGGCACCCTCGTCCCTTTTATCATGTGAGGACACAGCAAGAAGGCGCTGTCTATGACCCAGAAAGTGGGTCCTCACCAGCCACTGAATCTGCCATGCCTTGATCTTGGACTTCCGGTCTCCAGAACTGTGAGCAATTTTTTTTACAAGCCGTGGGGTCTGCAGTCTTTTGTTTTAGCAGCCAAAAGAGGTAAGATAGGGCATGTTGGGAAGGAATGGAGATGTCCACAAACACCCTGAATCATATACTGCTCCCCAACCCCCCGTCCTCCCAGCAGAGAGAGCAGGAAAGAGAAGGCTTACTTCCTCCAGGTTCGATGCTCTTCTACACACAGTTATGACAGACAGATTGCCTTATATTTTTATTCTTTTTAGTTCATCTGACCAATTGTCAAATTGCTCAAATGTCAGAAAAATGGCTCAAAGGGCCGCTATGGATTTCTGCAGTAGAAAAAGAAAAGACAGAAGACTAGATCCCAATGTGTTCCTGGACTGGAAGAAAGTTCTTATTTTATGGAGCCATAAATAAATATGACATTTCTTGTGCCTGAGAATTTGAGGCAGGTAGTACTCCTGTGAAGTAAGATAATGTCTTCTGTAAAAGAATAAATTCATTAAAAACCATGGGAATCATTGTAAGTTTCATTGTCAAGAAAGAAACAGACATGATTTTGGATGTAGGTGAATGTTAATTATTGAAGATGATTATTGTTCTCAGAACAAGTTTATTCTGATTCGTAGCCACAGCAGTTCAAGAGAAAAGCAATAAAGGAACCACAACCATATGACCCTTCTTATAATCATGTTGTGGTGGGGATGTTTCTTCTCCGTCCTACTTCCTGAGAATGACAGAAGGGTTTTGCAAGAGTGAAGGCAGCTGGGAATATATTCCAGCCGCTTCCATAGTTCATGCTGTGGTAAGGAGTTTCAAGGTCACAGTGAGGCAAGGAGTTTCAAGGTCACAGTGATTGAACACTAGAACTTGTGCCTCTGTTCTCTGCTGAACGTCTTCCATGACTGCTACATCAGGGCTTGGGGTTCCCACTGACGTGGTGTTTAAGTAACATTTAGAGTCCTTATGGTTATACACTTTCATCTCCTTGTACAGAAAGTTTCTGGAAACTGCCCACTATTATATGACACATATTAACCTGTTGAATTTGGTTATTTATGTGAGGAAACCACAGAAAACCATAACAAATCAAAATACCTAAGAGCCACAAATTTCCTCCAGTGCAGCCACATCCCATAGACAGGTAATGTGCACTACATGTGTAATTTTAAGTTTTCTAGTAGTTGCATTCAAGAGTGCCCGAAGAAACCATTGATACCAATTTTAAAAATACATTTAATGTATCCCAATATTTATAAAGTACTAAGTCAGCAGACAATAGAGACAAAATATAGTTTGCATATTTTTTACTACATATTTGATATTCAGAGTACATTTTACACTTACAACACATCTCGGTTTGAACAAGCCACATTTTATGTGCTCAATAGCCACATGTGGTTATTGGCTAGCATTTTGGAAAACACAGTGCTAGAAAATGCATTCTTCCTGCCATGATCAACCATTGTCTCTCACTTACTCCTGGGCAACTGTGTTCTAATTGATTTCCGGGCATTGATTATTGCCTTTCAGGGAGAACAACTGATCACCGTATTATAGTAGGTCATTCCTACACATGGCCTTCAGGTCCCAAACCCGTCTGATTTGCTAAGCCGTTTTTCCCTCTTGTCATGCCATCTTCCCTTCATTTGCTACATTCCAGGTTTTCTAGTCTAATGCAGTCACTCCAGGCACTCTGTACTTGTACTCAGCATTTACTGGGTGGTGTATATCTGTCGTAGGCTGTTGGTTGTAAGTTTCATGACAGCATACACTATGCCTCCCTTTTTCCACATGCACCAATCCATCAAACCTCATTGAGGACATAAAACACAGCATATAAAGCACTCCATCGATTGAATTGAATTAATGTGTGAACAATTGCACCTGCAAGTGTAACTGAGGGCTCACGTGGTTGTCATGTATCATTTTTAAAATGTTTAAATAATGCGAGTTTTCATCTATATTCTTATTACTTCTGTAGAAATTAATCTATAATATTTCAACAGTAACATGGTTGAAATTGAGGCCTTATGTAATGTTTGAACACAAATGATAACTTGATTCTGAATCAACACTGTATGTGCGATTTGATGTCTGATGTATGATTTGGGGCAGTTTGAGGGTCAGTCATTTATTTGTACTGAGCCTCTCAAATTCCCTGTATGTGAAGGGAACAGTTGAGAATAAGTGTCTTCAGTGGATAAGACAGTCGTCTTTATCCCTGGAAGGCATCACCAACTGATCACAGCAGTCTGTTTTTCTGAGTCAAGAGGCAACTTCCCCTCTATGTAGGATACTACTTTTAGTGTAGTGTGCTCTTCCATATCTATTGGAATCATTACACCTGATCAATCAGGTTTAAGATAAAGGGTGTGATAGATAGAAATGGATGCAGATGCTCTTGCAAATTGAGTTGAACCCTTTGTCTTTGCATCTTGTGCTGGCCTCAGTGACTGTCTTCTTGAATAGAATGTTCTGGGAGTAAAGCACTGGGACTTCCAGGGCTGGATCATAAGAAGCTATTAAGCTTCCATTTAGGGCACTTGGAGTACTGACCCTCAGGGCATTCTCTCTTGGAAACCACATCTCATGTTGCAAAGTGTTCAAGCCCCATGGAGAGGCTATGCATGGTGCTCCAGTCAGTAGCTTTAGCTTCACTCCCGGTTGACAACCATTAGTACCGCCATGTGAGTCACCCATTGTGGACATCCCAGCTGATTGAGGACTCCTGTCTCTTCCTATCCCTTAGCTGACTAAGGAGATCTCAAGAGAGAACTTCTCAGCTAAGCCCAGTCAGCTCACAGAATCATGGGAGATCCTCATAAAAGGTTGTTTGAAGCCCCACATTATGGGCATGTTTGTTACACAACATTAGCTAACCAGAGCAGGCACTGAAACTGGAAGTGAGGTTCTGTTTCAACAGAAACCTAAAGTACATGGTGTTGGTGTTGGACCCTCCATAGGGCAAGACTAAAGGCTTGAAGAACAAGGGAAGAAAATTGGAGGCTGGGGAAATGGAATGGACAAAGAGAACTCTTTGAATGACTCACTCACAGCCTTACAGGACGAGAAGTAACTTTTAGCACTGTGCAACTGCAAGCAAACTGGATTTTGTCCTTTAAAATAGAAAGATGGCATCTCAAAGAACACATTTGTCATGAGTAGTTCCTAATAAGCATAATACTTAACATAAAGTTCACTGGCGTATGTTATTTATAATCTTACTATAGTATAATTTCCATTGGATAGCAAAAGGTCAAGGATATAATTACAGAAATATATTCTTTTAAAATTTCTTTTGGTTACACTTAAATGTAAATTGTGAACACCATTTTATTTTCTATTGTATCCCATGACTTTTCTATTGTTTGGGTCATATTAAATCTATTTTTACAGTATAAATTTTGCAGCATATATTCCCACAGGAAAGAACAAATTATAAAACACACAGTTTGTATATGTCTTTCCTTTAAAAGTGAAATTTTAACTAGTTTTTCTTTTTTTTCTGTTACTATGTCTTTCCATTCTTTGGTTCAATACATTCCCACCTACTCTTGAACGTTTTTTGGAAAGTTGGCAATGACCCTTTAAATTCTTTTCAGTCTCTATCTGCCTAACATATATTTAGGTTCCGTATATATTTATATCATTTCCTACTTAAATACACATATTTCCATTTTTGTGCTCATGCTATTCTGCAAATGCCTGCATTTTAAGGATGAGACATACATTTAAAAAGGGCATCTATGCCTTCTTTCAGAATTTTTTTTCTAAATATCTATTACTTTGATATTTGAAATTTTGTACCCACAAACATACACATACACCCATGTGTGCATAATATACATCTCACAGAAATGCCAGCCATGTCGGGAAAATGACAGCTCCATCAGAAATGTCTTTACATCCACGTAATATATCTTATTTCCTTGTATAAGGCACAGATCCTCTGTTACCAATATCAACTTATCCCCAGGCTCTAAATCACTTGAAGCTACTTTTGATTCTCTGGAGAATTTCAGAATATATTTTTTTCCTCAAAATTTCATGAACTTGTATGCATTTTGTGCCTCAGACTTTGAACGCCTTGGACAAATTCCTTTATCCCTGTGAATTTTTAACGAATTCTAAACAAAATACCTGACTCCACTTTCCCCCCAAATTTCCTGACCTTGCGTGCATTTTGAACTGCAGACTTGAAAACACTTGTGCAAACGTTCCTTCATCCCTATGAATCTTTAATCCTAAACAAAATGCCTGTATCAATGCTGGCAAGGTTGTGGAGAAAAGGGAATCCTTATACACTATTGGTGGAAGTGTAAATTGGTTCAGCCATTGTGGAAAGCAGTGTGGCCATTCCGTAAAAAGCTAAAAGCAGAACTACCATTAGACCCAGCAATCCCATCCATTACTGGGTATATACTCAAAGGATTATAAGTTGTTCCATCATAAAGACACATGCGCACATATGTTCATTGTAGCACTATTCACAATAGCAAAGACACAGAGTCAACTTAAATGTCCCTCAGTGGTAGACTGGATAAAGAAAATGTGGTACATATACAGAATGGAAAACTATGCAGCCATAAAAAAGAGCAAGATCATGTCTTTTGCAGGAACATGAATAGAGCTGGAGGCCATTATGCTTAACCAACTATGTCGGTAACAGAGAATCAAATACTGCATGTTCTCACTTATAAGTGGGAGCTAAAGATGAGAACACATGATCACATAGTAGGGAACAACAGACACTGGGGCCTGCTGGAGGGTGGAGGGTAGGAGAGGGAGAGGATCAGGAAAAATAACTATTGAGTACTTGGCTTAGTACCTGGGTCATGAAATAATCTGTACAACAAACCCCCATGACACTAGTTTACCTGCATAACAAACCTGCACATGCACCCCTGAACCTAAAATAAAAGTTTTAAAGAATGCCAGTATCCACTACATTTATGGGCGGTCTTTCTGAGTTTCACCTCAGAGAAACACTCCTAAAATTCAAGTTATGACTATTTAGACTATTTGTTAATGATAGCTCTGTGTGTGTGTCTTAGCCCCCTCTCTGTTTCCTATGTGTTCTACTTGATTTTTAAATAAACTATAGGAGCTCCACATACTAATTTGATTCTCTACATAAAATGGTGCCATATTCTCTTATTTTTCCTTTAGGATTTGTACAGAGACTGTACAAAATATTTTTTGAGTTGTGTAATGGTATCCAATATGGACAATAAATGATAAGTAAATTTTGGAAAAATCAGTTAAAAGAAGTGTAATAGATACATAGGTGTCTTAATTGTTTTCCGTCCTCAAGTATGGACGTTTTTGCAAAGACACGAGCTTTTTACTTCAGGAGACATTTGTCGACGTCTGGAAAAAATTTTGGTTGCCACAGCTAGATCATGGGGGTGGGTATCACTTGCATCTAGAGGACAGAGGCCAGGGATGCTTTTAAGGGACCCACAAGGCACAGAACAGCCCCCCATGACAAAGAGTCTTTCATCTACATGTGTCAATATCGATGAGATTGAGCAACCCAGGTATAGAGTAATACTGATGAGCACAAAGTATAGCTTGAAGCCTCTTTTTCCATATGGCTGTGATAGATTGTTTTAAATGATCATTGGAAGAAATAAACCCTTGGTTCTATGGAAGTCATGAGGAATATTCTGCCCATGTGCTTGTGAAACCTCAGCTTGGAGCAAAGAGGCGAATATCATGCAAGTGGCTTCCTAGAATCATGGGGTTTTGTACAGATTATTTCATCATCCAGGTATTGAGCCAAGTACGCATTAGTTATTTTTTTGATCCTCTCCCTACCCCCACCCTTCACCCTCAAGTAGGCCCCAGTGTGTGTTGTTCCCCTCTATGTGTGCATGTGTTCTCATACTTTAGCTTCCGTTTATAAGAGAGGACACGCAGTATTTGGTTTTCTGAGCTGGAGGCCATTATCCTTAGAATCTTCTATGTTAAAAACAACAGAGCACCTCCTGGCTTTCCTGGGAATCCTTGTTTCCTGATTCCAGACAAGCGCCATGGCTGTGAAATCATGTATTTATGTGTATGCTGTTGGATTTTAATGTGAAATACCTTTTCACTGCGCCAAGTTCGCTTCCAAATGTGATCCCGCCAGGCTGACCAACAAGGCATTCAGTCAGCCTACTTTCTTATGCCGGGACCTTTCACAAAATGAATCATATGTCACTTTTCTTTTCAGAAGCATATGCCATTTTATTTTATTCTGGGAGTTTGAATCACACCATGCATCTGTTTTAGTGTTGTTTTTAGTAAGTTCACTATCAGTGCTTCCTGAGCATGGTTTCTCGTATGGGGTACTCACTGACCTGTCCCATCCATCTTTTCTTCCTATAAAGCCTTTACTGCTATACTTGTCTACTTGCAGAACCTCCACACTTTTTATGAGCTCCCATTTTTCTCTCTTCTTGGTATTTATCATTACTTATTGTGACTCTTGCATATTGGATGGTCAAAAGAGATCCCCAGTGGTTACACTACAACAAGATAAATGTAGGTATACTTTTCTTAATTGTTATTAGTGTTACTTATTATTTTGTTTTATTAGACACTACTTTCAAAGGCTTTACAGCACTGGGTATGTGTTCTACCTTTTTCTTTCATTTTATCCTCCACAACAGTTCTGTGATGAAAGTACTATTATTAACTTCATAGTTTACACGACAAAGCATGGTTTCATAACTTGTCAGGATTTCTTAGCCATTATTTGATAAAATTAGGGATCTAAATTCTGTCTTCTAGCTCCAAACAGATGGTTCTTTCCATGCTATTTGCTATTATCTTGTCAAAAGTAATGACAAAATAGAACTCAAATAGTATTTTTCTTTTGGCTGATTTCTTCTTTCAGACCAGAGAGGTTTCCAAGGTTAAAGTAGTTCATTAATTTCAATTTCTTCTTCTTTTTTTTTTTTTTTTTTTTTTTGAGACAGAGTCTTCTGGTTCTTTTGCCCAGGTTGAAGCACAGTGACACCATCATAGCACACTGCAGCCTTGGCCTCCTAGGCTCAAGCAGTCCTCCTCTCTTGGCCTCCCAAAGTGCTGGAATACAGGGGTATGCCACCATGTCAGGCTACTTTTTATTTTTATTTTTTTAAGAGACAGTCTTGATCTGTTGCCCATGCTGGTCTCGAACTCCTGGGCTTGAACATTCCTCCCTCCTTGACTTCCCAAAGTGCTGAGATTACAGACATGGGCCACCATGCCTGGCCTTAATTTGGGTATCTTCTAATTGATGTGGACTCTTATGCCCTATTCATTTGTGTTTTGAAGTGAACTGACTCTGAATGTCAGTGATAGGGCACTGCTTAGTGTTGGGGGTGGTTAGGAAGATATGCAAGTTTCTTAGAGAATAAAGCAGCTTGCTGTTCACAGCAGAGGGGGTGTAACTGTTTCAAGAATTTTAGAATACTACTGTCTGTGAGTTCTGCAAGAAGTTAGGGAAGCCTCCCACTCCTGGTTAGACTGGCAGCAACTTTTTGCATTATAACACAACAGACATTTCATGTCCAAGCCAGGTAATCTGAGCTACCCTTGTTCATTCCAGATCCAGGGTTGGTGAGGCAAAAAGGGTGTCCCCAAAATAGATGGGTCTCTTTATTGAACTTCTGGGTTATCTCCATCATGTACAGAGATACAGAATCATGCATTTATAAACTTTATGGTTGAAGATGGCACCCACAGTTACAGTTTCCTCCCAAACCTCCCTGGCCTATCTCAGTTCTTAAAGATGTCTGGGGATTCCCAGTTAGGCATAGAGTAACAAGGCAGCTCTATCCTTAAATGATCATGGCAAGCTGCCATATGGCTGGTATTCATCCTCAGTTAATGTGGATATTCTAGTAGGAGGGCACAGTGACATAGGAAGAAATGGTCACTCTGTGTTCAAATTATTCCTTTAACTTAGAAGGCAAGTTTACCACCCTGTGGGTACTGAGCATTGCAGACTTCATGTAAGCATATTTTTGAGCATTTTCTACAAACCCTCATTTCTCCAAATCCCATCCTTTGCAACCTCAAGTTTATCCAGGGGATTCACACTGCCTGCATGTCCTTGTATGCGTTTCTTATTGTTCCTGTAACAAATTATCCAACCTGTAGTGGCTTAAAACACACGCATTTGTTATCTCACCATTCTGAAGCTCTGAAGTGTGAGTAGCTCGGATGGTTTCTCTTCATCATCACCCAAGGGTGATTTCTGTGTGTTGGCAGAAAGGCTGTGTTTCTTCCTCCAGACTCCAGGGATGCATCCACTTCCAGGAACATTTGGGTTGATGGCTACATCCAGTTCCATGGGGTTGAGGTTCCTGCTTCCTTGCAGGCTATTGGCTGAGGGCAAATTTTGGCTTCTTGAGAACCGTAGCATTCCTTGACTCCTGGCCTCCTTCCTCCCCCTTCAAAGCCAGCAGTGGCAGCTTCTAATGCACTGAATCTCTCCGACTTCCTTTTCTACCTCTTGTCTCCTTTCCCAAGTTGCATGGCTTGTCTGGACTGATTGTTCCATTACCATTTTCCTGCTTCTCAGTATCATGGACCCACTTGGATATTCTAGGATAATCAGCTTATCTTGACATCAGCTGCCTAGTAACCTTAATTATATCTGCAAAGACAATTCACAACAGTACCTAGATTCATGTTTGATTTAATAACCAGGGGAACGAGAATCTTGGGTGGATGACTTTATAATTCTGCTTACCACATTCCTGTCTATAAACTAATCTTAAGGTTGGTGGACAGGCCCCTTACAACTGACTTTGAGTACCCAGAACACTGGCTTCCTATCTTTACTCAACCAGTGGGCTCCTCCAGGAAAAGCCCAATCAAGGAAGATAACGCCATTATTCTCATGCTTTTCCTTTCCCCTTCCCTCCCCTTCTCTCCCCTCCCCTCTTCTCCCCTTTCCTTTCCTTCTCTTTCATTTTGAGACAGAGTCTTTCTCTGTCTCCCAGGCAGGAGTGCAGTGGCATGATCTCGGCCCAATGCAACCTCTGCCTCAGCTTCCCGAGTAGCTGAGACTACAGGACCATGCCACCACACCACCTAATTTTTCTATTTTTAGTAGAGACGAGGTTTCGCCATGTTGGCCAGGCTGGTCTAACCTCAGGTGATCCACCTGCCTCAGCCTCCCAAAGTGCTGGGATTCCAGGCATGAATCACCATGCCCAGCATGTCATGCCCTTTCGAAGTCTGGGTAATAATCCTCAGATGGTAGTGCACATAGTTATGGAGAATTAGTGAACCACTCCTCCCTGATGTGGCTCGCCCCCACTGCAAATAATTTGTCTATTTTTATTTTTATTTTTATTTATTTATTCTTTTTTGAGACAGGGTCTTACTCTGTCGCCCAGTCTTGAATGCAGTGGTGCAATCATAGCCCACTGCAGCCTCTACCTCCCAGGCTCACGTGATCCTCCCACCTCAGCCTCCCGAGTAGCTGGGACTACAGGTGCATGTCACCTCGCATGACTAATTTTTAAATTTTTTGTTGACGCAGGATGTTGTTATGCTGCCCAGGCTGGTCTTAAACTTTTAGGCTCAAGCAGTTCTCCCACCTAAGCCTCCCAAAGTGCTGAAATTAACAGGTGTGAGCCACCCAGCCTGGCCTATTTGTCCTTTTTAATTTAAAAGACTCAACATGTAGAAACCATTTTACCCCTTCACCTTGTGCATTAAGAGCTTCCTTTTTCTTAACATCCTGCTCCTTGAAATCAACCCACTCTACTTGTATGGCAGTTGTTATTTTAATATTTCTAATTAAGATACAGTTTTCATTTTACCTTACAGAGACAGTGAGCGGGTGCTCTTGAATTCCAGTCTGGCTTTCTCCATTCCTTTGGGTAATCACAGGTTAACTTTTTTCCTTCATCAGTTTTCAGCAGTCAGTGAAAGGTGCATTCATTTTCATAAATCAGCCATTTGGCAACATTTGAATGTTTAATCAGTTTGCGATCACATCAAAGAACAAGGGAAGTTCTTGGGAGATTTATTACCTCCTTTGGAATCTGTGTTCTTAGCTACAAAGGTGCAATGACTTTTTCTAGTTCTCTGCCCCAGATGTCTGAACTGTTAATATTTACAGTGCTCCTTTCCTGAAATTCAGAGTCAGCACCTCATTTTATCCTATTTGTATCCCAACTTACTTTATTCAAAGAGATTTTACAACCTGAGATAGCTCCGTAGGAAGAGTTCAGTTGTCAGAAGCAATCTGATCCATGGAAATTTTCTGGTGTTTGTTTTTCCTTGAATTAATTTGCAGGTTTAAATTCTTGCTTAGGCCACTCTAGGACTTTTAATTGCTATTTCTTAGGAAATATTCCTTAGAACATGAAGCAGTCTGTCTTTCAACACACACACACACACACACACACACACACACACACACACACACACACACACCCCCTAGCATACGATCCAGAACAACGTTTTATCTTTTTTTTTTTTTTTTGTAGGAGGGAGTGTCTCACTCTGTCACCCACGCTGGAGTGCAGTGGTGCCCTCATAGCTCACTGCAGCCTCGACCTCCTGAACCCAAGTGATCCTCCAGCCTCAGCTTCCCAAGTAGCTGGGACTAGAGGCACACACCATCACACCCAGCTAATTTAATTTTGAAAAAACTTTTTTTTTTGTGGAGACAAGGTCTCCATGTTGCTTTGGTTGGTCTTGAATTCCTGGGCTCAAGTGATTCTTCTGCTTCAGCCTCCCAAAGTGCTGAGATTTCTGGCGTGAGCCACCACACCCAGCCCTAACATTTTATTCTTTTACTGACTGTGAGATTTTCATTGACTTACGCTATGTCAGGCAGACTTTTCAAGCCATAACCTGGCTTTGGTGATTTATTATTTTAGCTCTTCATGTTTTAACAGCTTCTCTGCTACCATGATAGGTTATAATAAGTGATAGAAGAAAGGCATTTTAAAGTAATTTATGAATGTGGATCTCATTTTGCTTAGCTAAAAAAAAAAAAGTTTTTTTTTTTTCTAGAGAATAGAACCAAACAGTGTTCACTGTATCACATATTCCTTTTAGTGTATTGAGCATTAATGGGGTATTTTTGCAGCATCAGATCTTCACAAGGCTGGGGTTCATCAGCAGCACAGTAGCTATTAGGTGATTTTACTCAAGGCAGCAAAATTCGTTTCTTATAACACAGTCTCTATTGAAGACACACTCTAAGGCAGTTTGCCTCATCTATTTAGCTTTCCAAAATTCTCTCTTAAATTGCAGTTTAATGAATAGACTAAAACACAAATTTTAAGAAAAATGTAGTTATAAGATATGAAGTGTCTTTTAAATCTGCCAGTGGTTTAAGGGATAGTATACATTTAAAATAAAGTTATAGGCACTGATTTAGTCCTGGAAAATAATGGCTTTATTTCAATAAGCCAGTATCAGAAATTAGTTTTTGTTTTCTTTTTTTTTTTCCGTGATGAAATGTGGTTTCTAGTACTGGATAAGAAATGCATGAGAAATAATGTATCCCAGCATATTTAATATGCAACAGTGTGATCTCAGTAGCCTTGCAGATGGCTGAGCTGAGGCACTAAAAGTGATGAGATGACATTTTGTATTTTTCCACACGTTCTTGCCCATTCTCAGGTGAGTCTGGGCTCTCATCAGTATTTAAATGCTGTTTTACCTTGGCAAGACATTTAGGTCCAGAAAATAGTTTAAAAAATTAACATCTACGCAGAAAGAACCTCCAGGTAGTTAAAAATAGGGCAATTTGCGGATACACCACATCCTGAAGACTTAGTGTTGCTAAGTAAACCACATTATTTTAGGTGTTTCTTCCTGACATTTTTATTTTTTTCTTGTGTTATTTTAATTCTGGAACATAACTGGGAACTGAGAATACTACATGGGACCCTTATCTCTTTTCTTTGTTATGACTGAAAATCATAATTTGAAAGATGCTTGGAAAAGGGAAAGCTTAATATCTTACACATATTTTTATAAGACAAAAATATGGAAAGATATGAACCATAAAATCAGTTTAGAATGGGAAGGGTTAGTAAAACATTTTTTTTGAGCAGAAAAGGAATCATGGAATGGACACTTTATAATATAGTAATTCAGCCAATTTATTTGATGGAATTCAAATGTCATGTCCTCTTTGTAGCTAAGAGTGCACATTAGCATTAACCCTAAACCAGACCACTTGGAGCCAAAGAGATGTGTATGTGTGTGTGTGCATCTGCTTCTGTGTGTGTGTGTTTGCCCCATCTGAGTGATTTGATTTTTCACCATCTCTCTATTTTTCCACTTCCAAAATTTAAGCATTTAGACATTTATTATATTAAATATGTTTGCATTCTCCCTCCCTCCACATGCAGTGTTTTACAAATTTCCTATCAGACTGTTCCCATCCTGCAAACCCCCAGAGCTCTATGGCTGAGGTACTCCTCTTTCTGTTCCCTTCTCCATGCAGATGGAATGTCTGCTGGGAACTATCTTCAATCTATATGTTTCCCATTCGTAGAGGTGGCTAAATCTGTGACATGCATCCATCCTCATCCAATAGTGTCTCCACATGAGTGAGCTGGATAATGCAAAACCAAGCTTCGACATCAGTGGTATGAAGTACACACACACACACACACACACACACACGCACACACACAAATACAAACACACATAATCTCTGTAGCTCAGATTGGGATTGTCTAGGGTTAATATCTTTTGTGCTAAAAATATCCCTGTGCCACATTGAAGCTTATTATAATAATTATTAATTACTGATATATTTCAACTGTTATGTCTCCTAAAAATATGCATAGATTATTAAGTTTTCCCTTCTCCTTGTGTTTTTCTGATTATGATTTTCTATCATAAAGGTGAAAGTGATAAGGGTCCCATGTAGTGTTCTAACTCTAAACCTAATACTGACCCTAAACAGAATTGAACGCTTTAAACTAACCCATGGCCTTTGACCATTGCTTCTTGACCGTTGAGTTAACCCATAACCCTGAACAGAGAATGAGAAATTGAACCCAAATTTGAACCCAAACCCTAACTAGTGACTGGATATGAAACCTAATCCTACCCAACTTTGAAAAAGAACTCAATTCTAAACTCAAAAGCAAAGCCAACCGAACACCTAATCTAACTTTAATGTAAACCTTTGAACTTACCCTTAACTTTTGCCAGTAGCCCTTGACTCTTGACCCCTGATCTGAACACTGAAGGCATCCCCCAAATTCTCCGACCCATGGCCTTTGATCCTAATCTTGACTTTTGATCACTGTCCCTAATAATGAATATAATCCCTTGATCATAACATTGAACTTTGCTCCTACCCTGACATTCAATTAGTGATCTAACCATACCACAACCTGAACTTGAACCCAAATCCTAACATGAACCTTCCTCCATACCTGAAAGCTATCCTAACCCTTGACCTTTGATCTTTATTTTTCTCCTTGACTCCTGACTGTGAGATCCCAGCCTGGACTAAAATGTATACACACACTCAAAATCTTTTTTGTTCTGAATCGTTACCCAAACCTGAACTTGAACCCAAACCCTGACCCTACCCAATTACAAATCTGAATACAAAACCTATCCCTATTCTAAAGTTGGGGATTTGAGTCTCTTAGTCCCGTAGGGTAGATGTGGTGTTTGCAGCCCTGCAGCCACTATGGACACCACAGACTTGGACAAAATCTCCAACGTATTTTTGGGAAAAAAGGATGCAACCATTAGAGAACAAGATGTTGAAACTTTCATCCATAATCTCTGTTTGTACAGACTTCAGGGTGAAATACATGTGGTTGGAATTGTGATATTTCCAGCCACAAAATTGTATTATGTTGAGATAATGTGGGTTTCCCTATCCCTGAAAATGTGTTCATCCAACCAATAGTTACTTGTACCAGCAGTGCACCAGGGACCATTTTGGGTTCCTGGAGGCAGCCGTAAGCAAAAGCATCCCAGATCCCTGCTTCTGGAATCCCTGACTATGGAATTGGCATCCTCATAATGAATGTAATAAAGAAATAAGGTAAATAAAGAAATAATCTAGACTCAAATGTGAACTTTAGTCGCTCTGGAAGTCCAAACCCTGTCCAAACATGTCCGCCGATTACTTTCAGAGGATGGGTGATGACTCAGGTTAATATGGTTATTTTTGGAGCCCGTCTTACCTATTGTCCTTTATAGATGATGTGTTTTCCACCTCAGATATCAACATGAAAGACTGGGTCACTTCTCAATTCAGAAATCCACTCAAGGTTAGGCACTTTGGGAGGTCGAAGTGGGAGGATCGCTTGAGCCCAGGTGTTCAAGACCAGCCTGGCCAAATGGTTAAATCCTGTCTCTACAAAAAATAGAAAAAAATTAGCTGGGTGTGGTACCACCTGCCTGTAGTCCTGGCTGCTTGGGAGGCTGAGGCTGGAGGATACCTGATCCCAGGAGTTTGAGGCTGCAGTGAGCTGTGATCATGCCACTACACTCCAGCCTGGGCAACAGAGTGAGACCCTGCTTAAAAAAAAAATTCATTCAACTATGTGTAAGAGAGAGAGAGAGGTGTTTATTAGATTTAACTGAGGATTTGGGGAGAAACTTGGGGGCATTTTATCCTATGGGATAAGAGGGAAAAATAAACCTTTTAAATTAAACATCTCGCCCTTTTGCTGACTACCTTTTGGCTATCCTAACATGAAATATTCTTCTGGATGCTACAACTCTCAGCTCCACTGATCGGCTAGAGCAGATTCACCATCACTTCTTGTTTTTGGATTTCACCCTCTGCCACTCGTGATTTAACAAATAATTCTCTGAAAGGCAGTTCTCTTTTGAAAAAGAGTTTTGCTTCTCTGTGTTAAAATAATGTGTGCTGCTGTTAAAATAGTTTTGTATACACGAGGGAACTCCTTTAGAAGCTTTATCACGTCTCTTAGCTGTGCGTGCAATTTGAGTAATTACTATGTACCAATTCCAGTAACATAGCCAATACATCAGAACTCTCAGGGGACGTAGCTGGGAACTTTCTTGCAAAACAACTCCCACGTGTTCATTCCTGTCTGGAAACCACCAGTAAAATTTATAATCAGTAATAATTTCTCCAGGCACAGCAACTGAGAATGGTAGAACATTAGTTTTAAAAACCATTTTAATAAAATGCCTTTATAAATATTGAGACTTAATTATTTAGATTAATTTGTTCCAGTTAATGAAAGATCTCTTAGCACAAGACTGGGAAAAATTAGAACACGTATAATTTTCTTCATTCCAGATAAACAATTATTTTAATGTTTATCTGGTATTTGACCACAAACTTAAATTCCTGGGTTTCGTAGGATTAGAAATTTTAAGGTTAGTAATCACTCCCGTTGTTAAACTGCTGGATTTTACCTAAAATTACTGCAAGGATGTATCATTTTTTTATACCTCAAGCTGTTTTGTGCAGTTCTGCTTCCAACTTCCATAGACAATTTTAATCATTTATTTTTGTTTTTTCTTATCAGATAATGTTTCATAACATGGATGTGAAGAATTAAATGAACATCCTTCTGTGCACAAATTAAGATTAGAACACGAAGATTTTGGGATTCCCCTCAGTTCCTTTTATAAATTGTATTTCTTTGGACCTGTCCTAAGGATAACCACTTTTGTGAATCTGATTCATTATTTCCTTCTTTTATTAAGTTTTATTTCTGCAAAATTGTCATGACCAGCATAACCCAAAGAATATATTGTTCGCTCTGCTTTTGATCTTTTATAAATAGGATCATCCTATGTTCTTCTTGACCTGGCATTTCCCTTTTCATTGAATAGTATGTTTTTGATTTTAACCATGAAGATGCTTGGAGCTGTAGTTTATTTGTGTTCACTGATATATGGAACCTCACCCGATGGTTATACCACAAGATATTTAACTCTTTCAGAAGCTGGAAATTTGAATTGGCCTTATGTAAAGAGTTCAGCTATTAGGATTCTGTGCGTGTCTCTTGTTGAAAAAAAATGCAGAAGTTTCTCCAACTAGAAATGTATTTACTGGACCATATTTTATGTGCATATTTGGATATACACTCTCAGGTTAAAAACTGTTTAAGTGGTTGGACAGTTTTATTCACCCAAGAACAGTATCAGAGTTCCCTGTCCTCTCTGCATTCACTGCACTGAATCCAAAATTGAATAGAAATGAAATTAGCTGTCTTTGATTTGTTCTCTCTTTAGACAAAAGGCTTCCAATGTTGTATCATTATGTATAATGTTTGAAGTAAGATATAAATAAACTACCATTTTCAGATAAAGAAATGTTTATTTCTTTCCTTAATTTGATAACATACAATCATAAATTGGTTCAAGGCATTTTTCTTTATCTTGTAAGATTATCCTTGCTTTGCATTTAATTTTTTCATGTAGCAAATTAAATAACTTAACTTTCAAATGTTAAACTTAGCTTGATATTCAGTATCTTCTTTAATACTGTTTTTGTATTTGTTGTTAGATATTAATCATTTTTTTCTATCTCTGTACAAAACAAGATAGACTATAATTTTTCTTTGTTGAGCTTCCCTGGTTTTAGCATCGACTAATAGTAGCTGTGTAGAAAGAGTAAGAGAACATTTGTTTATGCTTTCTGGGAGAGTTCATATAAAAACACAAATTATTCATTCATTAATAGGTGGTAGACTTGCCATTCAGTCCACCTTGGACAGATTATTTCTTTGTTGTACTTAAAACCATCATTTATTTCCTCCTTGATTTGTGGACTACATTACATATTGACTTCTTGTATATATGAAGAAAAACATGTTTGTATGTCTGCACATGTCTGTTATCACTCTATTATGTTCCCTTTCTGCATTTGTCTGTCTGCTATATACATTTTGCTAAACTGTCATAACAAATTATGAGAAATTTAGCAGCATAAACGAATAGCCATTTATTACATCAGGGATCTGTAGGTCAGAAATCCTGGTGCAGTGGAGCCTAGCTTGGTCCTCTTCTTAGGGTCTCCCATGGCTGAAATCAAGAGATTGGCAGGGCTGCATTCCTTTCTGGGTGCTGTAGGGATGAATATATCACAACATATAGATTTTTAAAATCTAATTATTTGCACTAACTTCTGATTTTACCACATTAGATTCATAGGGTGAATTCCTGTCATATTGATCATTCGAGTCTTATGGAAGCTTTCTTTCTATCTTACAACATCGTCAGATTGTTACAGGTTTTCATATGTATTTATTCTTATGCTTTAAACAAGGGGTTTTCTCTGTTTTATGTAAAGTTTGACCTAATATTTTCATCATATCTGTGTTATACTTGAGATGTATATTGTGAATATATAAGCACACACAATGAACTATTCTTCAGCCTTAAAAAAGAAGGAAATAAGAAGGAATTCATGTAATTTGTGACAAGATGGATGTACCTGGAGGACATTATGTTAAGTGAAATAAGCCAGGCACAGAAAGGTAAACACTGCATGATCTCAATTATATGTGGAATCTAAAGAAGTCAAACTCAGAGAAACAGAGAGTAGACTCATGGTTGTCAGGGACTGGAAGTTGGGTTCATGGGGGAATTTTGGTCAAGAGGCATAGACATCTTTCTTCTTCTTATAATATTATGTTCCTATGTTCTAGTTTTTGAGCTATTAGGATTTCCATATCAGCATTTTAGGTCTTATTTATGCTTGCATTTTTTATATTCTTGATAATTTTAGTCTTTCTATATCTTTTGGGTTTAAATTTGTCTCTTGAGTTGGATGCATTCTTCATCTTAGGTTTTGTTACAAACATGAGATTGTCTGGAAATTTTTTTAAATTCATGAGTTTAAACCATTTATGTTTGTTGAACGTTAATTTTACCGATGCTTATTTCTGCCATCTTGTTTTATATGTTCAATTTAGTTACTTCAGGATAAGCGTAACTGTACATTTTGTTTTTGAAAACATAAGTTTCTACCTGTCATTTAATAGATATTTAAATACATAGTTATTTAAAACTCTGTTATCTATTTTTTATCCTTACTATGGTTAACCATAACTGATCACAGGGAATGCTGTTTATTTTTCCCAGTTGTTTTTATAAATTTAACAACATAATATTGGTTTATACCAATTTTGTTCAATTTCTATATGAAAATCAAAAATATATAGAATACATCAAGGAATTCATTGACAGATCTGGGAATTTCTAACAAGATAAACTTTTTTCAAACATGCATCTTTTTTAGTCCCACCCCTAGTGCTATTTAAGTAGATATTTCCAAGAATTTAAGTTCTGGGCTATTATCCATATATGATTTTTGTCTTCCTTTTTCTACCCATTTTAGCCAAATAGAAATTATAGTTATTGGTTGTGCTTGCATTTCATATATTTTTCAGAATTCTTACCAAATTAGTTATATTCTTTGATAAGTATTTTCTCAAAGATAATTTTCAGTCTTTAAATCTTTGCTTAGCAAAATGATTGAATCTCTTTTTGATCTTTTTTTTTAACTTGGCCTATAGTATTAAATTTTTTTAAATTCAGAGTTATTTTTCTTCAAACTTTCAAATATGACTCCTGTGTCTGCTAATGTCTTGTGCTATGACTGGGAAGTTTGATGTCAATCTGATTCCTATTCATTCATAGCTCACCCATTTTTCTCTCTGAAGGCTATTAGAATTTTCTGTTTGTCTTTGATGTTCTTAAATTTCTTAGTAATATATCTATTCAGGGCACTCTTTGAGCCCATTCAAAATAAGGTTTTTGTTCTTTTTGTTTGTTTCAAGTGTATTTTCATTCTTTCATCAACTTAGTTCTTCCTCTGTATTTTTTTTTCTCTTTCTGTTACCTGATCCTGGTATCTCTAACAAAGTCATCCATTTTTCCAAGGATGCCTTTCTCTCCTTTATTCTTTCCTGATGCTTTCTGGGAATTTCTTCCATCTGATCTTCCAATTTGGTAATTCATTCTATGATTTATCTTAACTATTAGGTTCTTGTTCATCTTTACTATTATTTATTCTATACCTACTATATTTACCAAGTTCTCTTTTACTTCTTATTATAATCTCCTATTTGAAATATATTCCCTTAGGTGATCGAATATATTTATTTTGTCTATTGTAATTTCTTCATTGATCTGTTCCAATCATTATATTTAACGTAGAAGAATTTTTTTTTCTGTTGAGAGAGAGCGTTTGGTACCTTTGTAAATGTTCAGGTATATAGCTCTTTGTTAAACATTTAGCCTGTGTTCTCCTTAGGTGAGTGGAAACTCATCCATCACTCTGGTTTGTAATTACGCATGTGATGGGACCTAAGGGCAGACCCAAGTCTATGTTTCTTCTATGAGATTAACATTCAACAAACACTTTTAGATCACTCTGGCGCACTGAAGAAGTTTGAAATTTGAGATTTGGCTTTAAACTCTCTAAAGGAGCCAGCATTAGGAAGAAACAGCCTCTTTAGCTTCATTCCTGGGGGTGTGGAGGGGAAGGGGGTGAAACAGGAAAAGCCCATAGTGGCCATAAGTGACTGGTGGCCCTGAAAGTTTTTAACCAGCTCCTCAACGCAGCTGAGTTTTCCGTGGGCTTGCCAGAGTCCCACTACCTGATGGCTGCCCTCGAGTTCTAAGTTGTATGGAGAAGAGAAGATGGGAGGGAGATTAGACAATGATTAACTCAAGGCATTCTTTATAAGAGACAAGAGTGAACTTAATACTTTGTTTTTAAACCAGCATCTTTCTATTACCACTTCCACCCTCTGCCAGAAGGTGCAGCCACTCCCATTCACCATATATACATGATTCATCAGCTTGTAATCTCCTCGGGATGGCTTATAGCTTACTGATTTCATGTTCTATTATTGCTCTTTCCGCAGATTGATGCCTCGTCTTATCCTCTGTAGTTTTTCAAAAGTAGATTTCTGTGGAGGAAGGGGCATTATGTTCTATTCACCATCTCAAAAGAAGCATAACTCTCTTTCTTGGATATATTACTATTTTTCCCACGTTGTGTATGCTTCTCATTAAAGGTAGGATTCTAAACCATCCAAATGAATCTGTGCCACCACCTGCCCCTGGACTTTGGACTGAAGAGGATTGAGAAATGGTGAAATACTTAACTATTTGATAGCTTCCTTCATTCCCACAGACCACATCAGATGTAGTTAGCTAATATACCAATTAACAAAATTACCCAGGAAATGCAACATATATACTTATTTCATTACTTGTCAAAACTTTCTAAATGGCTTTCATCTATTTCTAAAAAGAATCCCAAATGTTCCAGGAACAATTTCCTAATGTTCTGGTTTTGAATATCACAGCTCATTTATCAGCGTATATCATAGCTATGACTATAGACGCCAAAATATTAAGTAATTCATAATGACAATTTGGACAATGAAGGGTATATTAGAACTTCTTTGAGTATTTTTTATTGCAATATGAATTTTTAACCAAAGACTTGTATGAGCTCCAGAGAGCAAATCCACTACATTTCCCCACTCTGCCTCCCAACCCATCACTATATAGATCCATTGTGGAGCTTTTTTACTTCTTTGTGGTGTATTAAAACAAAGGATATAATATCCCCTGATTATGGATGAAAGTGATGGAACATTTACTGCCATGAGAGTCCCTTATGATAAGTGGTAGCTGAACTGGAAGTTTAAAGAACTGTGGCAGACAGGATGGGGTAAATCAATAGGATCCAGGACCTAGGAATGCATCAGGAAAGACAGCAACAGGGAAGGATGAGCTAGAGCAATTGAAAGGGTGATACATATATTTGGAGCCAATTCTTTTTATGCTATCATCAAGATAAAACCAGTATTCCTCACCTGGTAGATATTTCTCTTTGCAAAGGTGGATATTCCACAGTTCACTTCCACAGACCTCATGCAAATGTCAGATTCAGCGGGGAGAGGGAGCACCCCAGTTTCTTTGGCAGCACAGAATATAATGCATCATGTTTATTTGCAAGCCTGGAGATATTCTTGCATACATATTTTATCTAGCAGATGACACTGGATCCAATTAATTGGTGGCTTTGAAATATATTTATTGGAATTCATTATTTTGGGTTATAGTTGTTTCTGTGATCCATGCAATCTACCAGGATACTCTTCATGCTTTTGCATTTAAAAGAATGACACCAAGGGCTTGTGAAAGGCACATTCTGGGGTCCATCCCCCACAATTTGTGTTCTGTTGCTTTAGGGGAGGGTGTGAGGATTTGTGCATCTACCTGCTTTCCACAAAGTAGGGTCCCTGCTGGTATAAGGGCACACCGTTTAAGTGCTACTGCACAGAAGCATCAGATGTCATTAAGATTGTGTGTTATCTACATTTCTTATTGTTGCTCAACTGCCAGTTACTCTTTTCATAAAATATGTATCTGTCCTATATAGGGCTAAGAATTAATTTATCCCAGTCTATAACTACAGAGAGAAGCCTACTTAATGAGCATTCTTGATGGGGCATACCACCCATAAATATGGCACCTTAGCATTTGAAAAAACAGAAGAAGCAGGAAAGTTCTCTCTGACCTTCTCCCCATCCTTCTCCCCTAAAGCCAGGTCATAAGACCCTCCTATGAGAGGTGACTCTCTATACCAAGAGGAATAGAACATTCTTATCTCTGAGGACAAAAGGACACAGAGGAGAATCTGAACACACAGGCCTTGCTAAGTTCTCCCCAGTTTTTTCCCATTAGATAATAAACATTTTTACTTCAATCATACTTTCCAATGACTGTCCACTCTTTATCAAACCTAAGTATCTAAGCACAAAAATCCACAGGTTTCCCTGTTTCTTTTGGGTCTTCATTGCCTTATGAAGGCTCCTGTGTCATATAAAACTGTTATTAAATGAAGTGCACTCTTTGCTTAATCTGTCTTTTGTCATAGGGGCCTCAGCCATGAAACTAAGATAGGAAGAAAAGATATTTCTTTTCCCTTATATTATTCAACAATATTCTAGTTATACATGTAAGCTTAACCAAAAGCTTCTAGAATATCAAAGTAATAAGTGTGAAATATGTGTGTGTGCACACATGTGTGCATGCATATATATACACACACTACATTGTAGGTGTGTATATATATGTATATACATATACACATATATATTTTATAAGATGCGTATACACATATACATTTTTGTATGTGTGTGTGTGTGACAGAGTCTTGCTCTGTTGTCCAGGCTGGACTGCAGTGGCGCTCACTGCAACCTCCACCTCCTGGGTTCAAGTGATTCTCCTGTCTCAGCCTCTGGAGTAGCTGAGATTACAGCCATGTGCCACCATGCCCGGCTAATTTTTGTATTTTCTTTTAGTAGAGATGGGGTTTCACCATGTTGGCCAGGCTGGTCTCGAACTCCTGACCTCAGGTGATCTTCCCACCTCGGCCTCCCAAAGTGCTGGGATTACAGAGGTGAGCCACCACGCCAAGCCGGCACATAATACATCTTGTAAAATATATTTAGCAAAGTCTATTTAAAAATAATTAATAGTTTATTAAATCTTATGTAGATTTTTTTTTCAAAATGAACAAGCTTCTGTCTTTCCAACAAAGCTTTGGAAATAATAATCATTGCATTTTCCTCTAACAGGTTAATCAGCAGATCAACTAAAACCAAAATGAGTCTTTCTCTGGGCACGGTGGTGCATGTCTATAGTCCCAGCTACTCAGGAGACTGAGGCAGGAGGATCACTTGAGCCCAGGAGTTCAAGGACCAGCTTGGGCAACATAGCAAGATACCATCTCTAAAAAAAAACTAAAAATTAAAAAAAAAAATAAGTCTTTCTATAACTGTATGACAGGGCTAAGGTGATTTTATTTGACAGAGGAATTAAATTTCAATGTACCAAGTTCTATCCGTATGATATCTTTTCTGATGGTTGGAAGGGCACCAAGGGGCTTCCATGAAGCTCAGTGACAGCATTTTCACATGGAAGTCACTGCAGCGGAAAGTAGGGTACACATTCTTGGTAAATAATATATGATTGCACTATTGATGAATAGCATTTCAAAAGCTCTGCTATTTATTGTCTATTGAAAGATAAATGAATCCAGCAAGTAAACTGCCTAAAATATTTGTACACTGTTATAAAATGTAAACACCTCTATCATACTATAAATCTCCCTCCCCTCCGCTGGAAAAGACTTCAAGCTGAGATCATCCTCGTCCTCATCACATGATTGCTTGGAATAGAGTTGTCCCTGAGGCCACCTGTCACCTAAGAGGACTTGTATTCATTTATTCAGTGTCCATGTAATGAAAGAATAAGACAGACATACTGTGAATATAAGAACACAGAGTTCAAAAGACTATTCTGATTGAGCAGAAGGAAGATACTAAACAAATATTAGATGAACAAAGCTTGTGTGTATGGCTTTGGAAGATAAGCCTAGGATCTTAATCTTGTTTATATAACACAACTATTAAACCTTCCTGCGTAAAATACATTTTAATTGAGACTTAGCATGAAGATAGAACACCAAGTCTGGGCATTCTGAAAAGTTTAGACGCAGAGGAATAACTGGCAGGCAGTGATTTAAAGTGGATACAGATTTTTGCCCTGGAGTTGCAGATGCGTGTAGGAATGAAAAGGAAGTAATGGGTGTGATAACCGATTTAAACACTAATCAGTGAGCCCCAAATATTAACCATATACTGGGATTCTACAAAGAGATGCCATGGTAAAAATATGAATTCAAGTGTTTTAACCTGTATAGCTGGATACATTCTTGTGATATTAACACGGGAAATAAGAAAAGAGACGAGTTTGAATGAGAAAAAGATGTTTAGCTCAATATAGCACACACTGAGCTTTAGGCTCGAATAAGACATCTGAGTGGTGGAAGACTTAGTCAAGCATGGGAGAAGTTAGAGCTGAAACCCAGGTAAAATCCTTCAAGTTACAGGCAGAAATCATTACCAGATGTGTGGTGGAGTCACACGGGGGATGTGAGTCCTAATGCCTGTGCAGATGCATGGGGAATGCAGTGTCTTTTTGAAGGACTGGTTTTAGCGCTGCAAGAAGTAAAGTAAATTCTCTTTTACCTGCATTCTTGTTCCCTCTGGTGCTTTTATGAGGACCTAGGCAAGAATAGTATTGAACCACTTATACCATCCATCTGTTAGAAGAACCTATAATACAGAAATATTTGCTTTGGGCTGAACTCCAAACGTAATACTTAATGATTTCTCTTCAAGTTTGTTGACACATTCTACATCTCCACATACAATTTGCTCCCAGTCGTTTCTGAGATATGCTACAGAAAGTACAATTGATCAAACGTTGGCTGTAGGGATTCAAGAACAGTCCTGTGACTGCATTTTCGTTCCTTCCTGAAACTATTCCAAGGCCATAAAACACCTTTTTTGTGTGAACTGTCTTTCTGTATCCCATTTCAGATGATATCTTCTTTCCTTTAAATACAGTCTTTTATATTTTTCTAATTGTCTGATTGCCAAAACAATATATCTGCATTGCTATAAATTTACAGTATCAAAGATCATACAGAAGAAAAATCTTTTTTAACAAAAGAAAACCATTGTTGATAATTTAGTTTACATACATACATATGTACATACATGTATCCTCTTAGCACTCTGGGGCCCGGAGTAGAGAGCAAACCTGTGAAACAGATAGATAGATAGATAGATAGATAGATAGATAGATAGATAGATAGAAGATATAGAGATATGTTAGAGCTATAGAGATATAGTCTCTAGATAGATAGATAAGAATATCTGTATTCTCTCTCTCTAGACAAATGATTAGGAAACAGTCTATAAGAACGTGTGTGTGTGTGTGTGTGTGTGTGTGTGTGTGTGTATGCTCTCTAGAGGTAGAATATATCTCTCTGTAGGGAGAGAGACTTATGCATGTGTATGTATGTATAAAACAAAGAAACAATAAAAACACAAAGGCCCAAATATCAACCAGAAGAAACTGACCAGCTGTAATGGGACAATTAGAACATCTGTAAGAATATTTGTACTGGATTTAAAATGATAAAGACATAAAAGTTCATGTATTCATCATGACACCCAGAATAAAACTCACTGGTTATATTACTAGGCCACGATCGCATTTTCCTGAATCTTGATCAATAAAAGAATCATGATTTTTTCCCACTTTTCCTATATATATTGAATTTCAGAGTAACTAAAAAATTGGTGATTACAAGTAAAATTTCAGATAATATATGCAGAAAGAACAATACTATCTGAAAATCATTATTTTGTGAAACTCCAAATTAAGTAAGTATATTAATCTGTCCTCACACTGATCTAAAGAACTGCTGGACACTAGGTAATTTATTAAGGAAAGAGGTTTACTTGACTTGCAGTTCCACATGGCTGGGGAGGCCTCAGGAAGCTTACAATTGTGGCAGAAAGGGCAGCAAACGTGTCTTTCTTCACATGGTGGTGGCAGGAGAGAGAAATGAGTGCCCATTGAAAGGGGAAATCCCTTATAAAACCATCAGATCTTGTGAAAACTGACTCACTACCACGAGAACACCATGGGGGAAACTGCCCCCATGATTCAATTATCTCCACCTGGTTCCTCCCACAACATGTGGCCATGGAACTACAATTCAAGATGGGATTTGGGTGGGGACACAGCCAAACCATATCAATAAGAGATCTAGGAAATTATCTCTGATTATTTGAAAAGCTTCGCAGGTTTATATATATATATATATATATATATATATATATATATATATATATATATATGTGTGTATGTATATATATATATATATATATGTGTATGTATATATATATATATATATGGTGGGATTCATTACCAACTGAATGTAATTATCAACCACTCTTAAGATAATTAAAAGTAACACTAGCAGGTATCATGTAAGTCCTTATTAAATTCAGTATAAAGTACAGAGCAGTTCCTGGGTGCGTTGTTTCCTACAAAGGGCACCATAACCCTAAGGAAGAAAAACAAGATGTGATTAGGAAACATTCTGTTAATTCTAGAACATGGGGTGTTCTTCAGCAGTAATGTTCAAAATGTGGTTCACAAAGCAGCAACTTGTTAGAAATGCAAAATTTAAGATCCTATACCTGGGAGGAGGCTTCCTGAATCTAAAATTGAGGGTGTGGGTTGCAAACTATTATTTCTTTCCCAAACCCATCTGTGATGTTTATGCTTGATAAAATTTGATGGGACGGTTCATTGATTTCCATAAGGAATTAACGATGTAAGAAAATGAGAAGAAGAATTGTATTATGGAAAAGAGGGTGTCAATATTTTCACTTGCTTTCTCTTTAAATGTGTGGATCACAAGATTTGCTTTTCATTAAAAGTATTCAGATATATATACGTATTTGAAATACATGTGCCTATACACTAACCCCAAAACAGCTCATTAAGAATCTCTTCCACTGGGACATAGGCATCAGTATTTGTTAAAATATCACTAAGTGTTTAGTGTGGTTGATGAGTGTAGAAAGAATATATTTTCACCAAGCTTATGAGATGGGCAGTTTGGGGCCAGGAGAAGAAGGCAGTGAAAGAACTTGGGTGATAAGCAGCTGTCTACTTGCAAAACAACTTATTATTAATGAATTGGGACTTTAAATTTTTTTTTTTATTTTCATAGGTTTTAGGGGAACAAGTGGTATTTGCTTACATGAGTCACTTCTTTAGTGGTGATTTGTGAGATTTTGGTGCACCCATTACCCAAGCAGTATACACTGAACCCAATTTGTAGTATTTTATCCCCCAACCCCCTCCCACCCTTTCCCTCTGAGTCCCCAGAGTCCATTGTGTCATTCTTATGCCTTTGCATCCTCATAGCTCAGCTCCCACTTATGAATGAGAACATAAGATGTTTGGTTTTCCATTCCTGAGTTACTTCACTTCCAATAATAGTCTCCAGTCCCATCCAGGTAGCTGTGAATGCCATTAATTCATTTCTTTGTATGGCTGAGTAGCATTCCATCATATATTTATGTACCACAGTTTCTTTATCCGCTCGTTGATTGATGGGCATTGGGTTGGTTCCACATTGTGACCCAATGCTTTTAAAATAATGTGTGTGTTTGGCCACGAACATAAGCCAGAACACTAGAAAAATTGTTTACTGAAAGCCATCTTAGTTTCAGGAACACAAAGGAAATGAGGTAATGTGTGAAAAGAACTTTAAAAATTGTAAGGCATTTTGCATAAAGATGTTAGGTGCTTTTTGAAGTTTCTATTTAAATGTGGTCAATTAGAGAGGTTTTTTTTTTTTCATTTTATGTTTGCCTTGAAAGCATTTAGAAGTATGAGAATATATAATTTCATTTTGTAAAACACAATATGTTGAACCTAATAGGATCTTTCTTGGAAACTGAACATTGTCCTGGGTTTTGGAGGCATCCCATTGAAATTTAGCCATGATTCCATATTCAGCAAATTGCTGTGGACCCAGATACATCTTCGCTGACCAGAAGTCTTTCCAGAGTGGAAGATTTTAGTAAATGTACAAGTCAATCTTGTAGAATTAGATAAAATGCATTCTGTTTTCCATCACTTGCCGATATCCCCCCACTGCTAATTAAAGGAAACACAATCCACAATTGATTTACTTATGTAAATGTAGATTACAAACCAACAACATGATTTTAAGAGTCTTAAGAAGTTGAGGGCTATTTTGAATGTTTACTCTTGGAGACATGTATATTTAGGTGTCCTGGTCAACAAGATCAATTGTAGGAATGGTTGGTGCAATCACATTGGTCATTAAATACAGACATCACACATAATCAAGCAGATTTAGCTCAGGGTATGGGTAACTCAACATATGAACACCATTCAAAGTATTTCCCCAAAAGGCTGGCATGGTGGCTGACATGGTTTGGTTGTGTCCCCACCCAAATCTCGTCTTGAATTCTTGTGAGAGGGACCCAGCGGGAGGCAAGTGAATCATGGGGGCAGGCCCTTCCTGTGCTGTTCTCATGATAGTGAATAAGTCTCATGAGATCTGATGGTTTTAAAAAGGGGAGTTTCCCTGCATAAGCTCTCTTCTGTTGTCTGCTGCCATGTGAGACATGCCTTTTACCTTCCACCATGATTGTGAGGCCTCCCCAGGCACGTGGAACTGTTAAGTCCATTAAACCTGTTTCTTTTGTAAATTGCCCAGTCTCAGGCATGTCTCTATGAGCAGTGTGAAAATGGACTGATATAGTGGCTTACGCCTGTAATCCTAGCACTTTGGGAGGGCAAGGCAGGCAGATCGCTTGAGCTTTGCAGTTTGAGACCAGCCTGGGCAACATGGTGAAACCCTGTCTCTATAAAAAATACAAAAATTAGCTGGGTGCAGTGGCACAAGTGTGTATTCCCAGCTACTTGGGGACACTGGGTCAGGAGGATTGCTTGAGCACAGGATTGCTTGAGCTAGAGATGCCCAATGCATCTCAAGGGTGCAGTGAGCCGAGATGGCGCCACTTCAGCCTGGGTGACAAAGTGAGATCCTGTCTCAAAAAATAAAAAAATATTTCCCCAATGGGGACATATGGCTTAATAGTTAGGGTTATTGTTTGTAGTGATGAATAGGTTTGGAAATAGGTAGTGGTGATAATTATACCACATTGTGAATGTAATGAATCCCACTGAATTGTACATTTTAAAATGATCAAAATGGCAAACTTATCCCACACACAAATAAATAGATATAGATATACATAGATATCTTCATATGGTTTTTCTTGTTTTTTAATTTTTTATTTTTTTATTTTATTTATTTATTTATTTGAGATGGTGCCTCCCTCTGTCGCCCAGGCTGGTGTGCAGTGGCATGATCTCGGCTCACTGCAACCTCCTCCTCCCAGGTTCAAGCGATTCTCCTGCCTCAGCCTCTCAAGTAGCTGGTATTACAGGCCTGTGCCACCATGCTCTGCTAATTTTTGTATTTTTAGTAGAGACGAGGTTTCACCATGTTGGCCAGGATGATCTCGAACTCCTGACCTCAGGTGATCCGCCTGCCTCAGCCTCCCAAAGTGCTGGGATTACAGGTGTGAGCCACTGCGCCCTGCCTTCATATAGGTTTTAATATTAGTTTTGCTTAATTTAAAGACAGTTTGAGGCAGTACAGCATAAAGTACTCCCCACATTTCATTTATTTAGTTTTAATTGACAAGTAATAATTGTACATATTTACGGGGTGCATACTGATGTTCCAATACATGTAATATACAGTGATCAGATCTAAGTAATTAGCATATCCATTATCTAAAACATTTATCATTTCTTTGTGTTGGGAACATTCAATTTCCTCCTTCTAGCTATTTGAAACTACATATTATATTATTTTTAACTACAGTCACCCTGCAGTGCTATGGAACACGAGAACCTATTTCTCCTGTTTCCCCCCCTCCCCACGAAGAAATAAAAGAGGTGAAATCTGACACACAAAGCAAAAGGAACAAAGACATTCAGGTACTGGAGTTGAGCATAAATTTTACCTCACAATTTCTGGCAGATAAAGCAAAAAGAGAGAGAAAAACAATTGGTTCTGGGATTAGTATTTCCGGCAAGAGAAACCTTCTCCCTTTTCCCTCTGATTCTTGGTGAAGAGCAGTTATGATGTTGGAGATAAAAGGAGAAGATGGCAGTGATGGTTCCTTGTTCTTTCCTTCTGCAGTGGCTGTCAGCTTCGCCTGTGATATTAACAGTAAGACAGGAAAGCCTGAGACCGCCTCACTAAAGACAGACCCTTCCCATTATGTGTCACGGCAGCCTTCACCCTTGAATCTAGAAAATACTACCTGGGCCGTGCTAAGTTTATTCTTAAAAGCCTAACACCGTGTAGCTACCGCTGCCCAATGCATCTGCCCAAAACAGCACTCCCCAAATCCTGAATATGCATAGAAATAACTTTTCAGTTTTCATGCCTACTGCTGAATTGTACCAACAGAGATTCTGATTTGGAAGTCAGCGGAGGAGTCTATGCAGTTTAAATTTTTACAGACAACTCAAGGTTTTGTGGATATATCCTGAGACAGCTCAGCCCTCCCAGGCTGTTGGTACCATAGGGGCTGGGAGAGATTGCCCTCACTTACCCCGCAAACACCTTGCAGGATGCAGAACAGCTCTTAATAAATATGTGTTATGGAAATAAAGGAATGCCCCTGTGCTTGGAAGTATTAGGCTGCCTCTCTCTCTCTCTGTCTCTGTCTCTCCTCTCTCTCTCTCTCTCTCTCTCTCTCTCTCTCTCTCTCTCTCTGGTTCATTTTCAATGCCGCTGAGTCATACAGTGAGAAGCAGCTTAGGGTCATTAAGAGATTGAAACATCGGAGAAAAAAAGTGAATATGTTTTCATTTGAATCTCTATTTTTAACTCTTTCTGACCTTGTCTGTCAAATTTGGCTACCTTGAGACTGTTGCAGTGATAATGAAATAAGCCTATGCTGTTCTTTGGAATCATTTTAGACATATACATGTCTAATATATATATATATATATATATGTATATATATATATAAAAAATACTTACCATATGTGATCTTGTTTGACATGCCTTTTTTCTATACAAAAGCACATGAATCACCATGCTTCAGCAATGAAGATGTTGTGTTTTGGACTAAAGGCAGTGTAAACACAACATGCTATTAGCGTTTTTCTTAATCATCACTACCACCCAGCCGTTGTCTTCTTGCACAAGATAATAATAGCATCTCACATTTCCATGGAGCTTTATAATGCATGAAGGTCTTTCACATTCATCGTTTTGTTCAATGTCATAGGGAGAGGGAGCTTCAGTAACCTCAAAGCCCAGTGTGCAGAAAGAGAAACTGGGATTGATTCAATCATTTGGCCACAGTCCTAGGACTCTTGAATGTGTGTGTAACTCAGGCAGGGTGAAAATCCCAGAAGATACGTCCCCATCCCAAGGGCACCTACCCAGGTTCAATAACTTGGTTACAACTGACACTCTTTGGATGATGCTGCACTCTTCACAAACACGTATCCAAACCTATCATGAACCCAAACCAGAGCAAACATCAAATCCCCACTCCTACCACATATTCACATCTTGTATTAACACCAAAGCCTGAAGCACCCTGACAACATGTGTCCAGAAGATGTTGAATGTCTTCCACCCTGACTGCCACTAACCTGGTTAAAGCCAGGACCAACTTTCACCTGGACAACTGCAGTGGACTTCTTCCAAATGCACGCTCCTGCAGTCCTGTCCACCAGTGGCTCCTGTGAGCTTTACGAAACCATAAATCCTATCACATCCCTGCCATGTTCCAACTCTTATGCCTTGTCATTGCTTTACAATAAATTCCAACATTTTACTCTCTCTTTCAAGTGTGAACTCACTTTGCCTACTACTGGAATAACGTTCTTTCACATTTCCTTGCTAATTATGAGTCAGCTACACCGGGACTCCTTCTGTTCTCCTCACCCTTTAAACATATTTCCCATTTGAAACTTCGCAACTCGTAATTCTTTTCAGGAATGTTTCCCTTTCATCATCTTGTAGCTGCTTCGTTGTCATCATTTCCTTTCATTTCCACCTCTTCAGAGAGGCTTTGTTTAGAAGAAAACATAGGAGCAAATATTTGCCAGCTAGGGTTATGCAAAAGTTTCTTACATAGAACACAAAATGTAAGAACCGCAAGAGAATGTATTGACGGGGCCTTCCAGACACCATGAAGAACTCAGAGAGCAAGCTTCAGATTGAGAGAAAACATTTACAACTAATATAGCAGAGAATTGACTTGTAACTAGAATATATAAAAATATCTCCCAACTCAATGATAAGTCAGGCAACCTGCAGAGCAGCAGTCCTCAACACTTTTGGCACCAGGGACTGATTTCACGGAAGATAATTTTTTCCAAGTAGGGGAACGGTTTCAGTATGAAACTTCCACCTCAGATCATCAGGCATTAGATTCTCATAAGGAGCAGGCAACCTAGATCCCTCACATGTGCAGTTCACAGTAGGGTTCACACTCCTATGAGAATCTAATGCCACCGCTGAACTGACGAGAGGTGGAGCTCAGGTGGTAATGTGAGTGGTAGGGAGTGGCTGTAAATACAGATGAAGCTTCCCTCGCTTGCCCACCACTCACCTCCTGCTGGCCCAGTCCCTAACCAAAACTGGTCCATGGCCTGGGGCTTGGGGACCCCTGCTGTAGAGGACAGAATAACAGCCCCCTACATATGTTCACATCCTACCTTATGCAGTAACACGGACTTGACTGACGTGATAAAGATTTTGAGATGGGGAGAGTATCTTCAAGTATCTAAGTGAGCTCAGTGTAATAAAAATATCCTTATAAGACAGAAACTGGAAGCTTGGAACAATAGAAGAGCTGAAGGTGAAACAGACGTCAGACAGAGATTAAGATGCTATGCTACATATATATATATATTGCCCAGGAGGATTGCTTTGTTGGAGCCTATAAAGGTTTGTGAGATAAAAGTTCTGAAGTGGAGAATTGTTACTATGATGGTTGCTTAAAATCTAGTTTTTAGAACAGAGATGAGTTTGACTGTAGCTACTCTGGTATGTGATCCATTTAATCATTTTTTTGTTTAATTAATTCTCAGAGAGAAATCTTAGCCCCAGCTATTTAAATCTCTCCGTAAATAAAGACTTCCAAGCACCACTGGGACAGTTACCAAAAATGACAAATCATTGGCAATTCTAACAATAAACTTCAGTATAGTAAGAGATATAATGGAACTCTGTAACTGCTCTCAAATCAAACATCCCTCCTGCTTAGTGAAAACAGAATTGCACTAATTTCTCTGATGCAGTCTCAAAATCGAGCCACCATTTCAGGTTTTGTAAGAAAAGTTGCGGACTTCAGGCTTAGAAAAGACATCCCAGTTATGCATTGTGGCTGAGGATAACAGGAAGATGAGAATTAGGATGTAGCTATCTTCAAAAAGAAAAGTGGCAAAAAGAAAAGAAAAACCTTAATGTTAGAACATATAAAAATAGTATAAATAATTAAGAACTTAAAGAGGCAGAGAGTTTGGTTAAAGCGGCAAGCCAGCAGGACAATTTTAGTGTGTAGCTTAAGGTACAATACATGCATAATTTGCCCTTCCTCAATTCTAGAAAATATTGTCTGTCTTCTTCAATAACCTTTCTATTCCTGAAACTAAGCATTCCACTGGGATTTTAGCTATTTTATGTGCTGTCATATTTACTTTGCTAGCGTGCATGTGATTACACCTAAGTTCTAATTCGTCTAATATATGTGCATTACTTTTTCATTCATCTAATATGAATTCTAGTTCATCTAATATAGTGTTGTATAAGTTGTCTTTTTACTTATGTGCATTTTACAGTGCCCTGAATAGCACTGAAGTACAAAAATGTATCTTAATATGTGTTTTATCTCCATACTTTGATGGTGAAAAATATTACTTTCCATTCAAATGAGTTTTAAAATAGTCTCATTAAAAACAAATGAAAACGTAAATAAAAAAAAAAAAACCTTTTCTGTAATTTTTCAAGATATCTATGGAAATGCACTGAACTACCGCATCATTTTCCCCCAGATTTTGCTTGGGTCATCATGGCTACTTCATACATTGTTGAATGAGCATTTGCCGTAAATCTCATAGCATAAGTAAAAGCTATGATGTGTTTTTTGCCTTGTCTTTCTCTTTTCACTTTAAACACATAATAAACACTTAGTAAGCAGATTGAATAAATACATACCCCCTTTATGCAAAATTATTCACAAATAAATGGTATCTAATTCATTCTTTAAAATACCATCAGAGAACACACAATCTCGCTTTACCATCTGTATAATTTTAGAGTCAGAGTCTAATTTGACCAGCAGTCAATTTTTTCCCTTTGGACTCCTTTCATATATTTGAAGGACATTTCTTTGTTCCTAGTATGTGTAGATGAAGCAAAACTAAACTGCATGGCTTCTGATATTCATGAAAGCAGGCTAGTAGGTAACCATTTTTGAACACATTCCACACCAAGCGCAATGCTGTTTCTTGTGCATTGTCCAGTTGAGTTCCTCAGCTTGCTATGGGAGGGCTACTATTCTCCTCCCCATTTCACATATAAAGAAAGTGGGACTTTGAAATCTTAAGTCATGTGCTTAAGGCTTTGCAATAGATTCCACCACAAGGATGTCCAACTGAATCCACTGCAAGCAATAACTCCAAAAGCAGTCACCAAGCAAATGCCCTTGGTGTAAAGTGATACGTTCTGTAACAGAAATTCATTGAAAGCCATTTACTGTGCCGAGAGAATAAAGATAATCACACAGGACACTTTTGGAAAGGGGCCCACATTGGTAGAAAGAGATGGCCAGGTTCTACCTGAGTATGCAGGTGACCCCTGCGGAAGATGCAGAGACTGACCTTCAGGTCACACTGACCTTTCAAATGGTGCTGGGTCCATGAGCTAGCCGTGCAGCTTTGTGTGCGGCCATAGCCCACAGGAAGGTGTGCTCAGGCAAGGTGTTCAGGGCAATTTATGGTCGCCCAGCAGCCCTGCAGAGGGCGCCCTAGATCTTGGGGATTTTATATAAAAAGGCAAGCATATCAAAATGCAAGGCATATTCTGGAAATAGCTAGCAAGTGTTGTGGTGGATTTGCAGGCTCCAGAACATAAGTAGGGCATCCCTTGAGAAGGTCACATGGGCCCACACAGAATGACCCTTGGATTCCCAGTAATGAATTTGTTTCTCCAACACATTCACTTTATTGTGTGGGTGTGATTGTTGTTGCTAAAAAGGGAGATTTAAATTATGAACTAAATATAAACAGATATTAGCAATAAAACGTGAATATTCCCTATAGTCACATGCTTCGAAATAATCACCCTCCACTGTTTGAATTCCTTCCAGATTTTATTTTTTAATAAAACAAATGGGCTGGACGCAGTGGTTCATGCCTTAATCCCAGGACTTTGGGAGGCCGAGGCGGGTGGATCACTTGAAGTCAGGAGTTCAAGACTAGCCTGGACAACATTGCAAAACCCCATCTCTACAAAATATACAAAAATTAGCAGCAAATGGTGGCACATTCCTGTAATCCCAGCTATTTGGTGGGCTGAGGCAGGAGAGTCGCTTGAATCCAGGAGGCAGAGGTTGTAGTGAGCTGAGATTGCACCACTGCACTCCAGTCTGGGAAGCAGAGCGAAACTCTGTCTCAAAAAAAATACATAAAAATATAAAATAAAATAAATGATATGTAGTATTCAGTAGCCCACTATTTTTTCTTGAGATACCTTAGATGTGTTTCTGTGTCCATGAGTGTAAATACTCCTTAGCACTTTATGCAGACAAACAGCATCTCATAATATGGATCCAACCAAGACAATCCATTCAAATCTCTGACTTGTGAACATTCAGCTTGCTGCTATTGTTTGTTTTTAAAATTACAATCCGTGTGAATTGTGGAGCCTTGGGTGGCTATTTTTATGCATCAGAATAAAGATTTTCATAGAAAGCATTCAAAGAATTAGGACTCCTGGGTCAGAAGTTTCCAATTTTCATTTTCCTGGGTTTTACCCATTCTCTTACACTGGAGACCATCATGATGATGTTGATGGTGTGGAAATTCAGAGGAAGCTAAAAGGACGTAGCACCCAAATGAGCAGGTCCTGGGGAACTGTAGTTCTCATCTCAGACACTAGGATGCAGACCCAGGGAGGGACAAGATGTACAAGCTCTTTGAAAACTAAAACCAAAGGATGTGACAGCCAATTGAATGAGAGTCAGCAGCAAATCTGGAGAAATATCTAAGGAGAAAGTGTTCAGTTGAAAATACTCAAAGTTGGCTGGGCATGGTGGTTCACACCTGTAATCCCAGCACTTTTTGGAGGCTGAGGCAGGGAGATCACTTGAGCTCAGGAGTTCGAGACCAGCCTGGCCAACATGATGAAACCCCGTCTCTACTAAACATACAAAAATCAGCTGGGTGTGGTGGCGTGTGCCTGTAATCCCAGCTACTCTTGAGGCTGAGGCAGGAGAATTGCTTGAATCCAGGAAGTGGAGGTTGCAGTGAGCCAAGATCACACTGCTGCACTCCAGCTTAGGCGACAGAGCGAGAGTCTGTCTCAAAAACAAAAAAAGACTCAAAGTTGACTCAAAGAGATTTGTTTCCAGGCTGGCTATTCCCAAATTTTCATTTGCATGAAACTCATGTATCAATTATCTTCAGATTTTGATGTTTTATTATTTAATAAACAAGCTGTATTTATTATTTTATATTTTGTTCCTAAAATATGATCAATTTCAGTTTTTTTTTATTTCATGTAATTTAAACTAAAGCACTTAAAAAATACCTATGCAGCTTAATTCTAGATAAATGTGTGGAGATATTATCTTTTATGAAAATACTGCAAGGAATCTCAGTAGTAGTTCATAGGCCCCAGAGTTTGGAAACCTGTGGTATAAAAATCATTTGTCTCTTATAATTGTTTTAGATTTTCTCTACTGATATTGGAAATAGACTTTGGTTCTGTGCAATACTGCTGTGATAACATGATACAAATGGAGAATGGTCTGCTAGTACTCATTCCATTTTTAAGTAAAAACTAAAAATAAAAGGCACTGAATTTCAAGCAGCCCTGCCTATCAGGCAACAGAATTTGAATGCAGTGCATCAGCTTCCCTCCCTTCCAGCCCATCCTGTGCAATGTGTGCTTCTGTGTTAAATCCTACATGTTTTCAGTCAGAAAGTTAAAGGTTCACCTTGTAATAAATGCATTTCATGAATGATTTCCTATAACCGTCAAAGATTAGTTTTGCATCTGTTTGACTGATTTTTGTTTTTTATGCTTCTGCTTTCATTCTAACCAAACAGATTTCTTTTTGACCTCATTTGCATGATCTAACACAAATTCTGTTTATTCTCACTGAAGAATTTTTATTTAAACATTTTCTGTTTGGTGTCACTTCTTTAGTTTAAAGGTGCACAAATGTATGTCTGTGTACATATACATTTATAGTCACACATGCATATATATGGTTATATATGCATATATATATATATATACATACATATATTATATATATATACATACACACATATCAGGTGAATTACACACACTGAAATGGTGAAAGATATTTAACTTCTGTATTCACTTAATTATCTCCTGGTTACTTGTCTCCAAAAAATGCCTACTGTGTTTATGCAAGGAATGATCTCCTAAAAGAAAATGGATTGCAGCTTTAACGTCATGTAACAATGTCTTTTGTTAATTGAAAGAAAAACAATATCTGACCCCATTGGGTTCCTTACCATTTACTTGCAGTGCATGGAGAAACAAAAGACCTGCAGTCATTCTCCTAAGCCCTGTGATGTAATTCAAAATGAGAGGAAACATCTACATTATTATCTATAATAAAAATTGGAAACTTTTCTGTTGTGACAACAATATTAAGCCTGAATAGAAATATTCATGTTTATATGATATTCACTTCAGTCAACATGCAGCAGGAAAAACTGTGATTCCTATTTTTAGTCATCTTTTCCATGAAACCTGTTTCTAAATAACCATACTAACTAAATGTACTAGTCTGTTCTCATGCTGCTAATAAAGACATACCTGAGACTGGGTAATGTATAAAGAAAAGAAGTTTAATGGACTCACAGTTCCACATGGCTGGGGAGGCCTCACGATCATGGCAGAAGACAAAGGAGAAGCAAAGTCACATCTTACATGGTGGCAGGCAAGAGAATGTGTCCAGGGGAACTGCCATTTATAAAACCATCAGATTTCATGACACTTATTCACTATCATGAGAACAGCATGACAAAAACCCACCCCCATGATTCAGTTACCTCCTACTGGGTCCCTCCCATGACACATGGGGATTATGGGAGCTACAGTTCAAGATGAGATTTGGGTGAGGAGACAGCCAAACCAGATCACTGAACGTATCTATTGATATCTCTTGTGTGTGTATATTTGTTATTGTTGTTCCTTCCAGGACCCTGGATGAAACTTGGCATCAATGTAGCCATTAACATGGATTCATTCACATGGTCTCTTTTGCATCTTTCTTTCGTTGTTCAATTATTGGAGGAGAGGTTGCTGATTACAAGCTTCATATTAGGGAGAGTAAAGCTCAGAAACCAAAATTTCATCGGCTAAAATGCTTAGAGAGTTTGTAGCCTAAAGGACCTGTCAGTTAAAGGAGCCATTTGTTGTAAATCTCTGGTTTTAGACAATCAAGTAGCTTGTTCTCTTCATTCACCTTGAACATATATTTAAAGTTAAGTGATCTATCCGAGGAATGACTTCTCAGGAGCAGCACTCATCTTTGGTATCATGTGTGGCTCTTTCCAAGTTGATGAGCTACCATCATTTTGCTTTCTACAATCAGGAGGCAAAACCCAGTGGTTTAGGTTTGCAGGATTCCTAAAAATATTAATTTTAATTTGCTACAATAAATACCAGGATTCCTGGTGTCAAAAAGCTTGCAAAAAATCACACCATTAGAATTTTTTAAGATCACTCTTTATTTACACTTAAGAAGATAGCTTTGCCAGGAAAATGCCTGCCTTCCTTCTTTCCTTCCCTCCTTCCTTCCTTCCTTTCTTCCTTCCTTCCTTCCTTCCCTCCCTCCCTCCCACCTTCCTTACTTCCTTCCCTCCCTCCCTCCTACCTTCCTTCCTTCCTTCCTCCTGCTCTCCCTCCCTCCCTCCTTCCCTCCCTCCCTCCTTCCCTCCCTCCCTCCTTCCCTCCCTCCCTCCTTCCCTCCCTCCCTCCTGCCTTCCTTCCTTCCTTCCTTCCTCCCTCCTGCTCTCCCTCCCTCCCTCCTTCCCTCCTTCCCTCTCCCTGCTCCATCACATCACAGAGCTGTAGTGTGCTGCCTGTTCCTTGCCTCCAGTCTTATTCACAGGAAAACCTGGCCAGGTGCTGATGAATAAAGAAGAAGACAGATTGATAGTGAGATCTAATTTTCACAGATCAGGCGACTTGGGAAAACAGGTCTTTTTATTTTCAAATGCTAACTTTCTGGGCTCATAGAATTCTGTATCAGTAAGCCCACATGCTTTTTAAGTCTGATTTATAGAAAACATGATTTGGCCCTCAAAACAATGTAACCTCCCAACAGATTCATCTTTACCACTACACAGATAGAGCTGATTAGTCAAGACAGAAGAATTGCAATAGATAAAGGGTTTAATTCCTGCAGAGCTGGCTAAATGGGAGACTGGAGTTTTATTGTTACTCAAATCAGCCTTCCCAAAAATTTGGAGGCTTGGGTTTTTCCAGAATACTTTGGCAGACAGGGGCTAGGGAATGAGTGCTGCTGATTGGTTGAGGATGCAATGATAGGGGTGTGGAAAACAGCCCTGGTGCACCCAGTCGGCCTCTATGTGGGGACACAGAGGAGTCACTGGTCCTAGTAGGACCAATCAGTTGTCAGAAATGCAAAAGCCTGAAAAGACATCTTAAAAGGCCAATCTGTACTATGCTTATTACCTGGGTAATGAGATAACCTGTACATCAAACCCCTGTGACATGCAGTTCACCTACATAATAAACCTACAGGTATACCCCTGAACCTAAAATAAAAGTTTTAAAAAGGCAAATTTTAGCTTCTAGTGATTGGGGAAGTTGCAAATCTTGTGACCTCTGGAATAATGGCTGGTAATCATTCAACTAAGCTTACATCTTAGCAGAATTCAGGCCTCTCTCATTCTTTAACCTGGTGGCCTTTCATTACTTTTACAAAGGTGGTTTAGTTTTAAGAGGGGCTATTATCATTTAAACTACAAGTTCAATTTCTCCCAAAGTTAGCTTGGCCCGTGCCCAGGAATGATCAAGAACAGTATGGAGGTTAAAGGCAAGATGGAGTTGGTTAGGTCAGATCTCTTTCACTGTCATAATTGTCTGACTATTGTAAGTTTTGCAAAGGTGGTTTCAAGGTGAAAGGACTATACTCTTAAAGAGCATAAAATTATTGCATTCATTGTGTACCTGAAACAGGCACTCCCCCTTGTTGATAGTTTAAAAAGAAAAAAATAATAATCCCTGGATGTTGCAATAAATGAAAATGCCATGGCAGAAACTGTGGAAACACCAGCCTCAAAACACCACATTGATTTGTTAAACTTCAGAGATCCATGGATTGTCGTTTCCCTCAGCCAGCCTGTAGGATATTTGGAAGAATTTCAGAACCTCAAAGATCAAACCATCCAATAGGATGCTGTTAGAAGAACTAAGATTTTTGAAGGCAGGGGATATTCATTAGCCTGCTTTTGGAAAGGTTAAAACACTCTGATTTTGCTAGGGAGGAAGAGTTTATGGTGGAAGAAAGGCCAATGATTTCCTGCGTGTTGAAAATCTTCATACTCCTCCACAGAAACAAAATAAGTCAACAAGTCATTCTGCAGAATTGAGAAAGAGAGAACAGTGAGTGAAGAAAAGACGTGCTGAAGACAGAATCGTTCTGTTAGAAAATTGCTCGTGCCTTAGGAATTAATCACCTCTTTCTTTAATAGGGGAAGAAAGCATTGCCCTGTGGTATTATAGGGCACCTAAACTGACATGATTCGTCATTGTCATATAAGGATCTTCGATCTTTTCTCCCAAGCAAAGCCTGATGCCTTTTATGAACGATCGTGTCAAAGATATAGTGATGGAGACAGGTGTTGCAGAACATTTTTGGCATGAAGCACTAATTAGTAATTGCTAATTAAATGGGGGAGGAGGCTTGGGTAATGTCTGATCGCACCCACTAATCGTAGCTAATCTCCCGTCACATCCCTCTGAACTTTAAAGAAGATCACATTGGTAGGATGTGTCTTAAGTATCCAACCTCGCAGTTGCGACGCTGCCTCTCTTTGAAGCTGCAGGAGATAGTGACTCCCGATTCAGGCTTGGAGTTTTTATTGTCATTGTTGAACGAAAATCGTCCTGTGACTTTCTTTGGAGCCAGGCCATTTCCTCCTTTCCAGCTCAGAGCATTTTTCCACAGGTGCTCAGGAAAGCTCATGGAAGAAATGCTGGTTGACTCAATTGGTATGCAGCCTCATCCTCTACTCTTTTTGTTTTAAAAGTAGAAGCCGGCACTCAGTCACTCCTTGGAATGCCGTCAACTTTGGTTAGGGACGTGCTTTGAGGGAATTGGTTTGATGTTATTTTAGGGCTTAAAGCAGCCTGTCTTCATACAAACATGACTGCAGGTGGCCATAATAATGTGCTGAGCATCCCTTGAAATGAGTGAATGACATGGCTCTTGGAAAAAAGAAATTGTATAGAAGGGGCAAATATCATAGTTGGGTAGTTGGGGAAGGCTCAAATAAGGACGTGAAAATGGTTAAAAAAAAAAACTTTTAAAAATTCTTTGTCTTTTTGGAAGGCATATCCAGTACAGATTTGGACATAAAGTTGGATTAAAGTTTATGCAATGAACTAAACTTGCAGGAGGCCTTAGAAAATATTCCTAGTTTTGAATCTGAGTAGGAGAGTGTATGTCTTCCCAAACTTGACTTCAAAACATCAGAAGAAAGCAGTTTTTCCAGGTCAAGCTATTTTTCAATACAGAAGGAACAAAAAATAAAATAGATTAACTCATAACTTTGCTATCATTAATACCAAAATTGCCATTTTTCAACTACTAAGGAGAAATTAAGAATCGTATGCCTTGAGTAAAATCTAGATCCTCAACTCACAGAATCCTTCTTTTTAAAATAAGGAAGGCCAGTTCCTGATATTTTGGGAACAGTTGGGGAGATGTGAATATTCATTAGCTTTTGGGTGAGGTTCAATAATTACATTTTTTTGTATGTGACTAATATTTTCGCTATGTAGGAAAATAGAGGTGTATACTATTTACGAGTCGGATCTAGTGGAGTCTGTAACTTACGTTGTTTCTTAAGCATTGAAAGGAGTTAAAACAAAATGTTAATAACTAATTCAGTGAGAAAGACAGGCGCACACTGCCTTTGTATACATGCACATATTCTTAGACACAGACACACATGTGCACTTACGCCCCCTCCCCCCCCCACACACGTACTGTTTTCCCTGAAAAATTTCTTGTAGGAGTCTGTTGCATTTTTCAAAAAAGAAAATGAAAATGTGCACAGAAATGATACCTTGAACCTAGTAAAATTTACGACGTCTTCTGGGATTGCTTCATGTTATTAATATTTTAGATTCATTTTGCCTTCTCTATTAGCCACATATATACACAAAGATGCCATGGTATCATAACATCAACCTAAAATAACCATTATTTATATAATTATTTCTGCCACAAAATTTTTTCTCCTGTTCTTCCTCTAATTGGTGGGGGTGAGAGTTGAGGAGAGAGAGAATGAAGAAGACAAGCTATGAGATATCTTTTCAAATAGCAGAGACACGTATGCACTTTTTCTATTTGGCCACCAAAAATATCTTGTGTTCTTTTGTAGGGTTTTTAAGTACCGGTGACCAGGCAGCAAAAGGCAACTATGGGCTCCTGGATCAGATTCAAGCACTGCGGTGGATTGAGGAGAATGTGGGAGCCTTTGGCGGGGACCCCAAGAGAGTGACCATCTTTGGCTCGGGGGCTGGGGCCTCCTGTGTCAGCCTGTTGACCCTGTCCCACTACTCAGAAGGTAATAATGGCACCCCCAGGGTGGGCGGGCAAATACCCTGAACCAAGAAATGAATGGTCAGAGTTCATATCTCAGATGCATGTCCTGGTTACCAGAAGTCACTCTGGCAACAGAAAATGCCCAAAAGATCAAATGAATCCATCTTCATGTCTTTTAACTCAGCTTTTGTTCCATTTGCTCTGTCACCCAGGCTGGAGTGCAGTGGTATGATCATAGCTCATTGTAGCTTCCAACTCCTGGGCTTAAGGCTTCTCCCATCTCAGTCTCCTGAATACCTGGGACTACTGGCTGCTTTTTAAAATTTTTTATAGAGAAGTGGTCTTGCTATGTTTGCCTGGGCTGGTCTCAAACTCCAGGACTCAAGCGATCCTCCTGCCTTGGCCTCTCAAAGTGCTGGGATGACAGATGTGAGCCACCATGCTTGATCAGTAATATTTTTCTCCTAATTTAAATGTGTGACAATTAGGTGTTGGTTACAATGATTGGAACAAAATAACTACTTTAGAAGTCCTGACACTTTTGTTTTTTTTTGCCATTCTGACTGTATTTGACTATTTGAAATTTTATTAACTTCTAGCTACAACTTAGTAAAAGTAGTATGGAAGAGAGACAGTATGTCGATAAGGGATGCGGGTGTATAGATTTTGTAACCATCAGGGCTTTTAGCCACATGTTTTTTAAGAAGTCGCTCCTCTCTCTAATTCATATTAATTCTTTAAATCTTCTGGAAATATTGAAACACGTCTGGTGCATTCATTTAGAAGTAGATTCTGGGTAGAAGTAGATTCTACCCAGAGGAATAGTGTCTCTCTCCCTGATGGTCTCCCTCCCTCCCTTGCTCTTCCCCTCCCATTCTTCTCTTTCCCTCTCTCGTCCTCTCTGTCTCTCTCCCTCTCTATGTCCTCCTCCCTCTACCTCTCTCCTGCTCCCTCTCTCTCTTTTGCTCTGTCTCTCACCCTCTCTCTCCCCCTCCTTCCACTGTCTCTCCTCCCTCCCTCTCTCTCTCCCCCTCACACTGTCCCCCCACTCTCCCTGTCTTTCTCCCTCTCTCTCTCTTCCTCTCTCTCCTTTTCTCTGTCTCCCCACTCTCTTACTCACTATCTCCTTTCCTCTCTCTCTTTCCCCCCTTTCCCTCTGTCCCTCTCTCTCTTTGTTTCTTTCTCTCTCTCTCCCTCCCTTTCTTCTTCTCCTGCAAATATGACTTTCACCAAAGGACCTCCTTCCTGGTCAGGTCAGCATGCAGCACTAGGGAGTGTCCAGAGTTTGCTTTCCCCTCTCCCTTCCTCTCTCTCTCTCCTGCAAATATGACTTTCACGAAGGACCTCCTTGCTGGGCCAGTCAGCACGAGGTCCTCTGCTTGTCCCCGTGGGAGCTCCAAACCCTCCCTGGGGCCCTGCTATTAACCTGGAAAAAGCTGATGTTGGCAAAGTGGAGAAAGAGGAAACCACAAAAACACATGTGCATCATGTTACCTCAACCAGATGTGCACTTGAACGTGTAGTCAGCATAGGCACCCGTACCCAACCAGATGTGCACTTGGACGCCTAAGCAGTAGATGGTTATGCTGCCTAAGTAATGGTCAGCATAGGCAGCCACACCCCTGAGCCCTGCTGGAGTGCCTGAGGCTTTCCCCGGAGGCTCACTCAGTGGATTCCCAGCTGTCCCTTTGTGAAGGAGGCTCCCTGCAGTATCCGATGAGAGACTTCAAAGAGGAGTCCACAGGAATTTGAGGCAATTGGTTCTGGAAGCAGGATCACAAATTCCTGGCTGTGGCCTAAAAGGAAGAGGCAGGAAAATCTGCAGTGCAGATCCAGCCCTGGGTTGCCTGGCCACACGCAAGTGAATATTCCTAATAGCCGTCTCAGTCATCAAGACAGCTTTGTAATTTGTTCTGTGTTGTCAGTGGTCTTCAGAATGGCACCACACTGACTGAACCTGAAGTTCTCAAAACCTTCATGGAATTTTTTTTTTTTTTCAGGGAGTCTCACTCTGTCTCCCAGGCAGGAGTGCAGTGGCACAATCTTGGCTTACCGCAACATCCACCTTCTGGATTCAAAGCGATTCTCCTGCCTCAGCCTCCCGAGTTGCTGGGATTACAGGCGCCCACCACTGTGCCCGGCTAATTTTTGTATTTTTAGTAGAGATGGGCTTTCACCGTGTTGGCCAGGCTAGTCTCGAACTTCCTGACCTCAAGTGGCCCACCCACCTCGACCTCCCGAATGATTATTTTTAAAGTTATCAGCTGGATATGGTGGCTCATGGCTGTTATCCCAGCACTTTGGGAGGCTGAGCGGGGAGGATGGCTTGAGCCCAGGAGTTTGAGACCAGCCTGGTCAACATAGCGAGACCCCGTTTGTACAAAAATGAAAATAAAAACCAGCTGGGCCTGGTGGCGCATGCTTGTGGTCCCAGTTACTTGGGGGGCTGAGGTGGGAGGATCGCTTGAGCCAGGGATGTCGAGGCTGCAGTGAGCTGTGAGGTTCCACTCCAGCCTGGGTGACAGAGTGAGACCCTGTCTCAACATACATACATACATACATAAAATTAAAAAGTATCTTTCTTTAGAGTAACTGCAGGACTTTCTTCACTTCGGCACCGTCTGGACAAGTTTCTGGATCGCTGTGCTCCTCAGTGTCTTCATTGGCAAGATAGGACAGATGAGGGTTTCCTGAAATCCTCCAAACTCTGAATTCCTTGAGTTTTTAGTTCATAATGTTTTGCCCATGAGACCAAATGGCCTTTGATTTCTTACTAGTGCTAATGAGAGGAAAGGCTCATATTTGTATTAACTTTATTTCAAAAACACGATAAGTGAAGAATCTGATGAACCATTTGGTAGAGAGATTTCTATGGCATTTTTGAAAATACCTCGATTTTCACTTTTCTCAATTGATATAATCACAATTGTAGATTTAGAAAGCAGTCAGAACCAACTTCAGGAGTAATCAAACACATGTAAGCCACATTAATTGGAGGGAGGTGTTAATTATTTAAGTCAATAGGTTGGAAATTATTATACTTTTGCATCGGTCATTTCTGCAAGGCATGCTTCTAAACAGCCCATCAATATAATCACGAATTATGAAAAATACAAGCCAGGCACTGAGGCTCCTGCCTGTCTATCATCCCAGCAATTTGGGAGGCCAAGGTGGGCAGATTGCTTGAGTCCAGGAGTTCAAGACAAGCCTGAACAACATGGCGAAACCCCGTCTCTACAAAAAAGAGACGCATCTGTTGTCCCAGCTACTTGGGAGGCTGAGGTGGAAGGATCATTTGAGCCTGGAAGGCAGAGGCTCCAGCGAGCCAAGATCCCGCCACTGCACTCCAGCCTGGGTAGCAGAGTGATACCTTGTCTAAAATAAAAATAAATATAGCCAGACTGTTTGCCTTAGGAATTCCTTGCCTGGTTATATGGTCTAATGAAGACAAAGTACACGTGGAAAGTGATAGTTTTATGAAGATGTTCACCACAGTATTAGTATCGTAGCAAAGAATGAAATGAAAAGCTACAAGATCAAAAGGAGAGGAAAATTATAATGAACCATATGTATTTACTCAATAATAATTTAAGAATTTACCTAAGATATACATCAGCTGGAAAAACAGTTTAGACAGCTATATAAATATTGGGCTCAGCTATGCAAAACAGACATTTGAATGGAGGGAAAGAGCTAAGAATTATGTGAACTCCTAGCATACTCATTACGCTAAGGTGAGTTGTGTTTAAAGTATGAATTCTGGGTGATTTTTTTCATTATCCAACTATTTTAGTCTTATCAGGAGTTCTGTTACTTCCCTAACATACAAATAAATGTTTTATGTATGTTACTTTATATACACTACTGCCTAAATTATTGCCAGTACTTATGAGAAGGGCGGGAAAGGAACTTCTCACAGCATTTTTTCCAATTCTGAATGTTTTAACTAATGAAAGTATCCAATAGAATACATATTGACTTTCTCTTTTGGTTTTTTTTTTTTTGGACATTTTAAAATAATCTTCAGAGCCAAGCACTCAAGTCAATACTTGCACATTTCTGACAGAAACGTTCCCAGGATGGCTTTGATGACATACTGGTCAAAGCCATATTGGTTTCAAGTTGCGGTCCTGTGTGTCATCTTTGGGCAATCCTCCAGTCTTTAAAATCACGTCTTCCTGATGACAGTTATATTTTCCTCATATTTGATTGCTTCTGTGACCTTAAAAATCGACAGGGCATGAACTTCTGGACTCACAACTGAATGCCTTATTCTTTAGTGCCCGACTCGGGCTGGGATTCACGGAAATGGCAGGAAGCAAGTGTAAATGGAATGCTGATTTTTACAGCGCACCTCTCTTGTCCTATCGTAGTTAAAAATACAGATTTTATACTTCTGGACATCCGTGTAGTAGACTGAACTCATGGAGAATTTTAAGCTACACAGAATTTTACTCCTAAAATTGCCCATGCTTTTTCAAGTTTCTCAGCAAGTGGAGCATTTTTATATGTGGCAAAATAAAATATACACATCTCTGAGTTTCCAATGGATGTAGTTTTGAAAGAAGTGACCTAAAAAATACTCCTTACTTGGGCACCCAGTTGAGGATTTCTTTAAGCATAGCTAGCTGAATGTATTTATTTTAATTGGCAAATCTTAATATCTTCATTAGACTCAAGGTAGAAGTAGAAATGCGCTCCTGAATTAGCACTCTGAAGTTGATTCAAGTGGATTTCTTTTTTTCCCATAATGAAGAGATACCTAGTTTTGCTTGTGAGACAAGAGGGCCTTTGAACTGGTACTAGCTTAAAGCATTTTTTTTCTTGGAAATGGGGAATGCAGTTGCTCTTGGAGTTTTTATATATGGCATCTGGAGGCAAGGAAGCAAAAACGACACTAAATTGTGGAAGGAAAAAGAAATCACATGTATTTTACCAGTGCAGGAGAAGTGTCAATGTGGTTTCATTTCCTTAAACTCGTGTGTGTGTGTGTGTGTGTAGAATAACATTCCCTAAAATGAATGTTCAGGAGGAGGGGTGAAGGGGGAATGGAAATGAAAATGGGTAAAAGGGCCCCTGACAGAGCTGAATGCTACTACATCCAGAAACTCACATGCCTGAGAGACAATCACAGCCTTCATTGCTCAGTAAAAGCTGCATTTCTGTCCTGTGGGTTTTCATTTGCATGTCCACAATTTTGCACCTGCAGGTCTCTTCCAGAAGGCCATCATTCAGAGCGGCACCGCCCTGTCCAGCTGGGCAGTGAACTACCAGCCGGCCAAGTACACTCGGATATTGGCAGACAAGGTCGGCTGCAACATGCTGGACACCACGGACATGGTAGAATGCCTGCGGAACAAGAACTACAAGGAGCTCATCCAGCAGACCATCACCCCGGCCACCTACCACATAGCCTTCGGGCCGGTGATCGACGGCGACGTCATCCCAGACGACCCCCAGATCCTGATGGAGCAAGGCGAGTTCCTCAACTACGACATCATGCTGGGCGTCAACCAAGGGGAAGGCCTGAAGTTCGTGGACGGCATCGTGGATAACGAGGACGGTGTGACGCCCAACGACTTTGACTTCTCCGTGTCCAACTTCGTGGACAACCTTTACGGCTACCCTGAAGGGAAAGACACTTTGCGGGAGACTATCAAGTTCATGTACACAGACTGGGCCGATAAGGAAAACCCGGAGACGCGGCGGAAAACCCTGGTGGCTCTCTTTACTGACCACCAGTGGGTGGCCCCCGCCGTGGCCACCGCCGACCTGCACGCGCAGTACGGCTCCCCCACCTACTTCTATGCCTTCTATCATCACTGCCAAAGCGAAATGAAGCCCAGCTGGGCAGATTCGGCCCATGGTGATGAGGTCCCCTATGTCTTCGGCATCCCCATGATCGGTCCCACCGAGCTCTTCAGTTGTAACTTTTCCAAGAACGACGTCATGCTCAGCGCCGTGGTCATGACCTACTGGACGAACTTCGCCAAAACTGGGTACGTTCATCTTCGTGTTGGGGTATCACTATCCTTGCCACTTGTTTGTGTCCTCAATATAGGTGTTGCTTCTACTGCCACGTGCAGGAGCACACACGCATACACACACATACACATGCATGCACACACATACACACAGACACACGCTTACACACACAGCAGTAACAGGCAGCTTCTCCCCCAACATCTATGGCAACTCATTTTTTTCTTTACTCCTAAAGTGTTATAGGAGTAAAACACTTAACTGTCAAACCAGATTTTTACTAGAGTTCTAATTGCCCATTGGGAATTCCAGAGTTCCTACCTGCAGGTGCAGGACTCATACATATATGATGGTTCTGTTAACAGCTGATTAAACGGTTTTGTTTTTGTCCTTGTTGTTTTAGAGACACAGTCTCACTCTGTTGCCCACACTGGAGTGCAGTGGTGCAACAGTAGCTCACTACAGCCTCCTTGAACTCCTAGGCTCAAGCCATCCTCCTGCCTCAGCCTCCTGAGTAGCTGGGACTACAGGTGCCTGCCACCATGCCTGGCTAATTTTTAATTTTTTTTTTTTGGTAGAAAGAGGGTCTCACTCTGTTGCCTAGGCTGGAGTATAGTGGCGCAATCATAGCTCACTGAAGCCTCGAGCTCATGGGTTCATGTGATCCTCCCATCTCAGCCTCTTGAGTAGCTGGGACTACAGGCGTGCACCACCATGCCCTTACATGGATTTTTGTAGACACAGGGTTTGCTATGTTGCCCAGGCTTCTCTCAAACTCCTGGGCTCAAGGGATCCTCCCACATCAGCCTTCTGAATAGCTGGGACTACAGGTGCACACCACCTTACTCAGCTAATTTTATTTTGTTAGAGACAGGGTTTTGCTGTGTCACCCGAGCTGGTCTCAAACTCTTGGGCTCAAGTGATCTTCCCACCTCAGCCTCCAAAAGTGCTGAGATTACAGGTGTGAGCCATCACACCAGCCCTCATTACAGAGTTTTAAGTCTAATTTCAACCATATCTCTTTTGTTAATTTGCAAGGATATCACAGCACATGTACCACTTGGGGAACTGTGTTGATTGCCTGGCCATAGGAATGAAAACAAATATCATAATAATTATAAAGAAATATAAATATATATTCCTATATATATTTAATGTCTATATAAAAATATAGATATTCCTATTTGTATAATATAGTACATTTATATTTGTATTTGTATATATATACACACAAATATATTTGTATATACAAATACAAATATATATACAAATACTATATATATACAATATATATACAAATACAAATATATATATACACAAATACGTTTGTATTTTCTCTGCTATATAAATAACTAGAGAGAGAAAATGAAAATATATGATATTTGTATCATATTGCTATATGTCATGCATACATAAACACACACACACAAACACACATACATGTGTATCTCACAGGAAAGCTCATTTATTGGCCTAAATATAGTAGAAAATATAAAATATACAAAAAGCATATATACAACAGAGTCTGCCAATATTCTGCTGAGCGGATTCTCTGCAAACCATGGGAGAAAAGAACCCAAAACAACCTAAATAGCTCCAAACATTGTGGCATTTTTTCATTTTCTCTTGTCTAATAATGTAACTGTGGAAATGGATGGGGTGTCATTCTGTTCTACCAGTGTGTGCCTCCATCATCACCCTGAGCCTCTTTACACTGAATGAGAGAGAAAGATGTGCCTGTCGCCCAGGGAGGGTAAATCTTCCCGTGCGGAATGAGGCTCTGAGACTGCAGTGGCCCTGCCACACATGAGTTATGCACAGTAATCCTTAGAAGATCTGGGGATGCTGGTGGTTTCAATGCCTACGTGTTTAGCAGCTGGCATACTGTACAAAGATTCCAAAGTGGTTTGGGTAGGGAGTGGTTTGAGAATGTTTTGTGCCCTTGGCGAAAGTACAGCATGTTTTTGGAGTGGAAAAGGTATCACCTGGATACCACCTTTCAATAATCAGACTTTGTAGATTTGGTCTGAGAAAGGCTACCCAGAGGAGAAGAGAGGAGGGACCCACATTTGATGCAAATGCTTGTCTATCACTCAACGGTTCTTTTTTGTGTGAAGAAATGATTGAAATCAAATTAATACTTTTTTTAAAGTAAACCTTGTTTATTAGTTTGTTGGGACTGCTGTTATCAGAGTATCCAAAACTGTATGGCTATGCTGGGCGCAGTGGCTCATGCCTGTAACCCCAGCACTTTGGGAGGCCGAGGCAGGAGGATCACCTGAGGAGGCCAAGAGTTTAAGACCAGCCTAGGCAACATAGTGAGAGTCCGTCTCTACAAAACAAATGAAAAAATTTAGCTGTGCATGGTAGCATATGCCGAGAGTCTCAGCTTCTCAGGAGGCTGAGGCAGAGGGATCACTTGAGCTCAGGAGGTCAAGGCTGCAGTGGGCCATGTTTGCACCACTGCACTCCAACCTGGGTGACAGACCGAAACCTTTATCTTTAAAAAAAAAAAAAAAAAAAAAAAAAAAAAGCACCAAAAACGGTGTGTCTTATAACAACAGAAATGTATCGGTTCACGCTTTCTAAGGCCAGAAGTTGCAAATGAAGGTGCTTGCAGGGCCAAGTTCCCTCCAAATCTGTAGGGGGAGGGTATTTCCTTGCTCCTTCTTAGTTACTGGTGTTTGGGTGCAGTCTTTGGCATTCCTACCTTGCAGGTGCACCATCCCACTCTGTGTCTTTGTCATCTTACGGCCTCCCTGTGTGTCTCTGTCTCCACATGGCCGTCTTCATATAAGAGCATCTGCCAAGGTGCATTAGAAGCTCACCCTACTCTAGTATGACCTCAACTTAACATAAATAGTCATATCTGCAGTTACCCTATTTCCAAATAAGCTCACATACTGAGATACTGGGGTTATGACTTCAGCGTATCTTAATTTATGGGGAGACAGTATTCAATCTCTAATACCCTGTGAAATCAGGGCCAGGCCCTCTTTTGTGACAGCACTGAGATAGGCGGTGTCTGCCCTTGCAGAGAATTTCATCCTCTTGAAGCCTAAAGACTTCCATGAGAGTTTCCCAACATGGCTATACTCATTCAATCTTCGCTACATTGGCATCCAAACGTATTACCGACTTGGTCTGCAAACACTCTCTTTACTTACTCTCATTAAAAACATATGCTTTTTCTTTTCCTCCTTACATGATTTGAAAATAAACTTTATATGATTATCTTAAGTGGAAAGCTAGAATCATTCCTCATACATTTTATGGAACCATTAAAACAATAGTGAAATCTAAATAATGCTGTTAAATTCTCATTAGCTCTTCCTGACTTCCAAAGGCTATGAGACTGAGGCTGGCTCTCTCATTATTAAAAAAAAATAAAAAAAAAAAAAAAGGAAAAAAGACAGAAAAAGATAAAGGAAGTTAATTAGTTCCATGAGGTGATCGTTATCACTGCTGACACCAAATGGACGCTTTTACCAAGACATCACGAAGGTCTGAGAGAGCCGTGAGAAGAGAATACCACAATGATCTCTCTGTTATTGAGTGCTTTTAATGCCATGAATCTGTTTCTTAAAATCACTTGGCTTAGAGCCTGTGATTTCCACCCTGCATTTAGGGAATACATTCACGTTGCCATTCATGGTCTGTGTTGAGGGTGCTTCTAGCTTTCATGAAGGCCCTGACATGGCTGGAAGAGATGAGGAAGGAATAACTGCTAGAACTTGGAGAGACGCTCTGATGCTACTGAAATCAAAAGCTGCAGGTAGAGAGAGTTCATTGAGGTACCCAGAGCTCGAATGTCAGTCCGTCTGAAGCCTCTATTTTTGTTTCTTCCGCCCATGGGAAACATCCCTGAAATAACACTGAGTGTATTAATGCAGTGAGCTCTTTTAATTCATTGGAAAGGTATTAGAATGACTCAAATGATTCCTCAAGGAAGTTACTCAGAACTTACATCTCATGTGAAATGCAACGTGTGGATTCAAATACAAATAGTTTAAGTGATCACACCTCCATGGCAGCCCCATAAAAGAAGGAAATGGGGAATTTCACTGTCGGGCACAGTCTGGTGAGCTAGGTATTCGTCAGTGGATGACAAGGACTTCAGTTGCAGTTGGTAGTTATTTGTTTATTGTAAATTGGGTGGTGGCCCGATCACTCCAGGGCAGAGAAGGATTCCCTGGTCACCAGGTGCAGAGAATGAACCAAACTGATGCCCGCAAGGAGAAAGTATGGGATGCACCTTATCTGCTGTCATGGTGTGAGCTGCCAAGTTTAACGCCATTTTGCAGAGCACACACTCAGATGATGACTCACAGAACAGGAGGGCATATTTCTGCATACCATCACTGTTCCCTTCCAGCACTGGAGGTGACAGGAGGAAACAAGAATAGCTCCCAGCGTGTCTGTCACTACACGGTGCCGTGGAGAAAGGATCGCATTGTGCCAGGACATACTTCACCACTCTCAGTGGGCGTTAAGTCAAGCGTTCTAAACCTGCAGGCACAGCCAGTCTCTCGATGGCGCATGTGTTTGCCAAGATGAAGTGGATGGGGTCTGGATGCTTCTATATAGACATCTCAAAGTAGATGGTTCTGACCTTTAGTCTAGGTTTGAAGGCACATATACCTGGTATACATAAACCTTTGGTTTTGGGATGAGCACAGAAAAATGATGTTGGGATGTGCATGGCGGAGAAAAGGAAGGAAGGAGGGAGGGAATGGAGGAAAGAGAGTTCAGACAAAGGAACGAAGGGAGGGAAGGAGGGAGGGAGGGAGGAAAAGAAAAGGAGGGCGGGAGGGAATCAAGAAAGGAGTAAAGGAAGGGAGGAAGGGAGGAAAGAAAGAGGTAAGGGGGGAGGAGAGGAAGGAAAGAAGAAGGGAAGGAAGGAGGGAGGGAAGGTGAAAGGAAGAAAGGGAGGAAGGAAGAAAAGATGGAAGGAAGGAAAGGAAAGAGAGAAAAGAGGGAAGAGAGGAAGGGAGAAGGGAGAAAGAAGAGAGGAAGGAAGGAAAGTAGCGAGGGAAGGAAGGAAAAAATGGAGGGAGAGAGAAAGAAGGAAGGGAGGGAGGAAGGAAGAGGGAGGAAAAAGGGAATGGAGGAGGAGAGGAAGAAGGGAGGGAGGGAAAGAAGGAAAACAGGGAAAAGGAGGGTAAGAGAGAGAAAAGAGGGAAGGGGGAGAGGAGGAAGGAAAGAAGGAGGGAGGGAGGGACAATTGGATCTTTGCTTATAAATTATGTCACCTGTATATTTTCATGGTAGCATTAGGTGAGAGGGCTCTCCCATCTTAGAAAGGCGGAGTCAGCGAGTACGCATAGTAGAAATGAGGAGGAAGTCCCTACGGAGGCTCTAAATTATGAAAACCTTGATCAAGAAAGGATGTTGAAATCATTGAATGCCAGGGCCTCAAGTAATCCTTGCTATTTCTTTTTTATTATTATTTTGAATAGGGAAGCAGTTGCCCAGGCCTGTGCCTGAGGGGGATCCTCCCCTGTAGCAAGGAGGTGTTTCAATGTTAGTCCAGGTCAGAGGACTAAAATCATGCTGGAAGAGAACCGTGTGAGCCCAAACATGCAGAGGCATTGTAGAAATAAGGTAGATTGAGACCGTTTTTGGAAATCAGCTGCAGTGTCAAGGAGAAGTGAAAACTAACTCTAAAGTTTCAAAAGGGTTCTAGAGCATTAAAGTCCTTTTCCTGGAAAATTACTTTGGGAATAGGAGAAAAAGGGTTCGTCCAAGCTGATCAATGAAATTCAGGTGCTCAGTGATCCAGGATTCTTTCATTTTGAGCTCTGTGTGGAAAGAGATGGACAAAAAGGAGTGGGGAATCTTGGTTTATTTATAAGGTATGACAAAGAACAGTGCTTTAAAGTAACCAAACAATGCATTATAATATAGAATAGAAGACCTTATGTGCTATTGGAAGTCAGATATGAGAAGAGAGTTTTGTAATGGAAAATCAGATCAACACATATTTTGATTTTTTTATGTTGTTCCATCGAGTCTGGGGTTTGTACGGCAGATTGATTTCTGTCCTGTTTGCATCAGCTACCATCACTGCTTTTGAATGTGCTGGTATCCTATGATTAATTTACGTTCAACTATTGTTAAATCTTTGGGAAAAAAAAGAAGTTCCAATGAGGTATTTAGTGGGGATGGGTTACAGAGAGTTGCAGCGTAATTCTGGCTGTAAAGGCGACCTTTATTACCAAAAAGGAATTTTAAGCTGAATGAATGAACATCCCCACCTGGTGTGGAAGAGGAGTCACTGAATGCATAATAAACTAGTCCGGTAATAATCGTTAACTGCGAACAATGTTTTGGGTATGAGGAAAACCTGTACTACTTAAAGGAACAGCTGAGAGGATTCACAGATATTTTTAGAGAGATCATAGTACTATATCCATCTCCAGCTAAAGAAATGAACTAGACCTTAGAAAGGCACTTGAGTCTCTGCTGCCAAGATGACATCTCAAATAAAACAGGACAGGTGGAAATGGCTGTGTTAGGTGCTGGGGGATAAGGAGGAAGACATGCATTGAGTCTTTTACTAGAGAGACCAACTTGTGTTTCTGTCCTCAATCATTATAGTCTTTAATTTTACTCACAGGAGTTTAAACACTTCTTAGGCTGAATAAAGTCTAAAAAACAAAACACTGATACCCCACATCTAGACCTCACTGTCTGGAGGGTTTGGTAAGGGAGAATGACTTGGGCTATCATAATCTCCACAAGTTTATCTGGCTTTAAGAATTCTGGCTGTGCATCTCCGAGATCTTTAATAGACAGACGGTATCAGGTGGCAGCTCATTTATATGGATTTTCCAAATCCTCTGCTTTATTCTTCAAGAACAAAATATAATGTGTTTTCTTTACCTTTCAAATATACCCTGAGTTCCTTCGAAAATAGCCTTGTACCCAACATGAACAGAATACTCCTTTTCCTAGATGCTCACTGCTTAATAGATGAGGTAGCCACACATCTAATAGATCCAATTCAGTAAAATTGGATCCATGGAAAAAAAGGTAGAATCTTCACTTCCATTTGTTTCTTTAGAATATTAAAAATCAATAACTAATATTAGTGGATTTTTTTCCTAAAATATTCATTCACTTATTTTTCTTTCAGTACACGTTAAATAACTGAAAATTTTAAAATTATTTCAGAGGACTTAAAGAGCAAAAGAAACATGAGTTGCTGCATTGAATCCAACATTTTTTCAAAACCATGTAAGAATACATGCATAATAAATAAAAAAAGCAGAAGACTTTTCAAATATATTGTTTATCAGTAAATAAGAAAACTCATGGTATTAGAACCTATGAGATTATATATATTTGTTCTCACCCTATTAGTAAAGTGAAAACACAGCAGTTAGTGTGCATTCAACTAAAGGGTAGAGGTCAACTTTCTTTTTCTCCTGTATTATGTTATACATCTAATATCTATATCTATAGATAGATATACACATACACATATATACACATGACATACATATATATACTGCATATAGTATATAGTTAGTATGCAGTATAAACTGTGGTATGCAGTATACTTGTATATAGTATGTAATATACAATATACTTTTATGCACTCTACAATGTATACAATATAGAAATTCAGTATGTACTCTGATATACAGTATATCACTCCCTACTTCTCCCTCCCTTGCAATATTATAGGTGTTCTATTTTTTATATTGGAAGAGAGGGGGTAATATTTCCTGAATTCTTACCATATGCCAGACATCTTGTCATTATCTTTCAACCTTCATCACTTACCTCCAACCCTGATATTTTCATCAGCCATGTAGAGGAGTAAGTTAAGGCCAATACTGGCTGGAAAACTTGCTTAAGATTTCACAGCTCTTAACTAGCCAGAGCTGCAGAAAGTTGAATACAGGGAAATGATTTATTTTATCACCACCACAGACTCAGACTGAGGGGATAAAATCTTCCTTCAGCAAGTGTGGCGCCTCTGGCTCAAGTATATTGTTTGAATCCTGCACAGTGTCTGGTAATGGCTACAGATACATGATCTTCCTTGGTCCTGCAGCCTTCTGCCATGCAGGCCATGCAATGACTGGAGGCAGTTTCACAGAAGTCCCGCCAAGGAGAAGTTACCTGGAAGATAGCCCTTAGCTCACACCTGGAGCCATTGATCAGGATGTTGCAACTCCCTGCTTGCCTGGTTCTGCACATCACATCTCAATGCTCAGTGCTAACTAGTACATAACATTTTGCCATGCATAATCTCAAATCGTTTTTATAACAAATAAACCTTAAGACGTAATTGTTTTTTAGCTTACTTTACAAGCCATAAAAAAAATGGAAGAAATGAGCATTTGGTAATTTATTTTTTGAAGGGGAAGTGTTATCCTAAAAGAGTCAGTTGCAAAGATGTTTATTAAAGGCCCTATGTTTTATGAATTATCTCCAAATTTTTATGATTCTCCTTCTACCTGTGACCACTTGTGCAAATAATAAGAAGATAATTCTTTGGCTCATAGTTTCCAAGCACAACTTAGCATCTGTAACAGCCCTTGACTTGTTTCTGGGTGTCTTTTTTATCTTAAACATGTTAACCTCATCATAACTATATGTACCATTTTAGCAAACTTCTTACAGCTAACATAGCGTGCTTTCATCTTTTTACCTTCAAATAGAGAGCAAACACATGGTGCATATGTCTATTTACAAACACTTTGTAATTATAAAGCCTATTTTTATTTCTACTGTTAATATCAATTTTCATTGCTAAAACTGCAACATTTATTCATTTACTTCAAAAGCAATTCTTGAGCAAGAAAGAGAATACCCATTTCTTGGACAATAGCTTCTTAATCAGAATTTCTCAACCTCAGTACTGTTAACATTTGGGTCCAGATAACTTCTTTGCTGTGGGGGTCTCTCCTGTGCACCAGAGGGTATTTAGTAGCATCCCTCACCTCCACCCTTCATAGAACAACCCTTCGTCTACGGAAACCAAAAGTGTCTCCAGATACTGCCAAATATCCCTTTGGAGCAAATCAGTCCTGGATGAGTTTTACAGTTCGACAAGAGTGAAACTTGAAATACTGAAATTTTTCCTAGAGACACTTAGTTTTCCTTCTTTCCCTTTATTTTTGAAGATCATTTGATGCCTTAAAAAATAGTAAACATGTTATAAAAATTGCATAATGCTGCTATCAGGATTTATATTTAAAAGAAAAATAAGAGCAATTTTTAAAGGAAAAGACAACATGGTAGACAGGTCTAGGATTAAAGCAGAATGTACCTTTGCTGCTTGGGTATTTTGTGCTCATTGATAAATATATATGAAGAGCAGATTGTAACTTCCTGATTTATTGGTTTAAGATAATTTCACGTCACATGTGGAAGAGTATGACCTTTCTTTTTTTCTTCCTTCTATCCTCAGTGATCCAAATCAACCAGTTCCTCAGGATACCAAGTTCATTCACACAAAACCCAACCGCTTTGAAGAAGTGGCCTGGTCCAAGTATAATCCCAAAGACCAGCTCTATCTGCATATTGGCTTGAAACCCAGAGTGAGAGATCACTACCGGGCAACGAAAGTGGCTTTCTGGTTGGAACTCGTTCCTCATTTGCACAACTTGAACGAGATATTCCAGTATGTTTCAACAACCACAAAGGTTCCTCCACCAGACATGACATCATTTCCCTATGGCACCCGGCGATCTCCCGCCAAGATATGGCCAACCACCAAACGCCCAGCAATCACTCCTGCCAACAATCCCAAACACTCTAAGGACCCTCACAAAACAGGGCCTGAGGACACAACTGTCCTCATTGAAACCAAACGAGATTATTCCACCGAATTAAGTGTCACCATTGCCGTCGGGGCGTCGCTCCTCTTCCTCAACATCTTAGCTTTTGCGGCGCTGTACTACAAAAAGGACAAGAGGCGCCATGAGACTCACAGGCGCCCCAGTCCCCAGAGAAACACCACAAATGATATCGCTCACATCCAGAACGAAGAGATCATGTCTCTGCAGATGAAGCAGCTGGAACACGATCACGAGTGTGAGTCGCTGCAGGCACACGACACACTGAGGCTCACCTGCCCGCCAGACTACACCCTCACGCTGCGCCGGTCGCCAGATGACATCCCACTTATGACGCCAAACACCATCACCATGATTCCAAACACACTGACGGGGATGCAGCCTTTGCACACTTTTAACACCTTCAGTGGAGGACAAAACAGTACAAATTTACCCCACGGACATTCCACCACTAGAGTATAGCTTTGCCCTATTTCCCTTCCTATCCCTCTGCCCTACCCGCTCAGCAACATAGAAGAGGGAAGGAAAGAGAGAAGGAAAGAGAGAGAGAAAGAAAGTCTCCAGACCAGGAATGTTTTTGTCCCACTGACTTAAGACAAAAATGCAAAAAGGCAGTCATCCCATCCCGGCAGACCCTTATCGTTGGTGTTTTCCAGTATTACAAGATCAACTTCTGACCCTGTGAAATGTGAGAAGTACACATTTCTGTTAAAATAACTGCTTTAAGATCTCTACCACTCCAATCGATGTTTAGTGTGATAGGACATCACCATTTCAAGGCCCCGGGTGTTTCCAACGTCATGGAAGCAGCTGACACTTCTGAAACTCAGCCAAGGACACTTGATATTTTTTAATTACAATGGAAGTTTAAACATTTCTTTCTGTGCCACACAATGGATGGCTCTCCTTAAGTGAAGAAAGAGTCAATGAGATTTTGCCCAGCACATGGAGCTGTAATCCAGAGAGAAGGAAACGTAGAAATTTATTATTAAAAGAATGGACTGTGCAGCGAAATCTGTACGGTTCTGTGCAAAGAGGTGTTTTGCCAGCCTGAACTATATTTAAGAGACTTTGTAAAAAAGAAAAATGTATATAGCTGTGAGTTTAAACAAAAACCACAAACAGACAAACAAGAAAAAAAGCTTTTATTGGTGTTTTCACTTTGAAAGAGCTTTTAGCAAGGTTGTGCTTTTCATTGTGCTCTGTACGTATATAAATATATATATATATACACACACACACACACATTAGTCATATCACCTCTGTTTCCTCCCCAACAAAAGAGGCTTTTCTTCTTAATTACTTGTGGTAAACAAAGACATGGGATTTTCTTACATGAGATTCTCATTTGTAGGAGGATGTGATGTCCCACAGAAGACCCAGACGGTCTGTGTGGCCTATTTCCCCCGTCAGGTTGCACAGGTGCATGCAAGAGCATTCTTAGGAGACCACTGTTTTGAAAAACTTTTGACTTGTACGTGTTAGCCTTCATGAAATTGCAGTACAGAGATGGGTCCCCAAAGTGGAGTGTATTTACAGCTTGTTAAATTAGAGACATGCACACACAAAGAATCAGTAGGGAGAAACAAAAATACAAGTCCCGTTCTGTAGCTCTGGCCCTTTGAATATGTTTAGGAAGAGTTGCTTCCCATTTCAGGGCCCTGCCAAAAAAAGAAGAAAGCTTGCCTTTGGTGGGGCTATGCCCCTTGGAGTAAATACGGCTCTGTGTTCCCTAGCAGCTGCGGGAGGGTTTGGCCGATGAAGTACCTGCTCAGCTTAGCTAATCAGATTGAAGGAAGACATGTGTCTTTCCTTTTTGTTTAAGCACTCGGTCCCTTATTTATCAGTAAGCAGGTTTTTAAAAATCTTTTATATCATTTATGGGATCAAACATATGATTGTCTGAAAACATCACTTTTTGTGGATTTGTGTATCCGGTCACCAAACGGTGAATATTATAGAAGAATGGGGGAAGAAAGGATAGAATATTAAAACTGCTTTGCATGGGTTTTCTGGGAAATTAGGATAACTTCACTGAGAAGACATTGAATGGAAATTATTCACCCATTTTAAATTGGTGACCTAGGGATCAGAGATTTGTCTTTCCAACAGCTTGTCATTTTTTCATTTCTCTTCTCATTTTTCAGGAAAGTTTTGAGTGTTATAAGGTGGAAGGAAACATAGTAGCAATGGATACTTTTTTGAAAAATTATTGCATTACCAAGAAACAGTAGCCAAAGATATTTGAAGATCATGTTCCTCGGCTCCATTGTGGGTTATTCTAGAAATCCAGTCTTAAATCTCTCCGCTAAAGTGGACATTCCCCATAAAAATTGTCCAGCTGCCTGGCTCTTTTGCAATAACAACCTTTGATTACTGAATCCCTACACTCAAACTATAGTGATATATCAGTGTTTGAGAGTGACCTCTAGAAAAAAGAAAAGTGTTTTTAGAAATGCGTACAAGTCACCCCCAAATCCTATTGCTTATCTTGGGTTAAATTTGAGAGTGATTCTCTGTATATAAATATGTGAAATATTATTATCTCAACTTAGCACACGTGAAGCAACATTTCTTTCCTACAGAGAGGTGTCATGGTAAGATTTCATTCCGAATTCATTGTTTCATAGAGCTATGATCAGGCCATTTCTGCAAGCAATGTATGACCCCACCTGAGCAACCACAAATAGGCTCTCTGTGAAACTACAAAGGAAGTTATGTGTGGCATCCATGTTGGTTTCGTCTGTCTGTAATGTGAATTCCAGTATTTGTTTAGTATTTCCAGTTGTCTCCTGCTAGCAATATGTACAGTAACGCGTCAGGCTTGTGACATTTGAATAAGGAAAAACAGAGTTCCTGTTAAGTGAATAACTTTAGCTTTTACAGGGGATTATGATCAAAAGTGATTTTAGTACATCTTAAATGATATCTTATTTCTACATGGAAAGAAGTTATAGAATCTTCATAGAGTTCTATGAGAAAAAATATACTTGCTATCTATAAAAAAGAGAAAAAAGAAAAAAAATGAGAAAAAAGTAAGAAAAAAAAAAATCCTGTCCTAGGCTTTTACTCTTGATCTTCAAAGGCACGCAGGGTTTAATGGTTCCTTGGGTTATTATTTTGCAGTTTTGTTTTTTATTTTGCCTTAAGTAATGATAGAAGATATATATGGCCGGACACATATGTATAAACTTTTCAGCAGCATTTTTAATAATAAAATATCACAGTATTTTCTAATGCTTTGTGCAAATAA

Primer annealing sites are highlighted in red;

Exons are highlighted in yellow

rhAMP-assay SNPs are highlighted in magenta

>NLGN4Y_Homo_sapiens_GeneID:22829
[truncated: 223,720 more chars]
