# Supplementary figures and images for: Novel human sex-typing strategies based on the autism candidate gene NLGN4X and its male-specific gametologue NLGN4Y
Source: Biol Sex Differ. 2019 Dec 18;10:62. doi: 10.1186/s13293-019-0279-x (PMC6921425; doi:10.1186/s13293-019-0279-x)

# Indel PCR using MX17673/MX17674 oligos

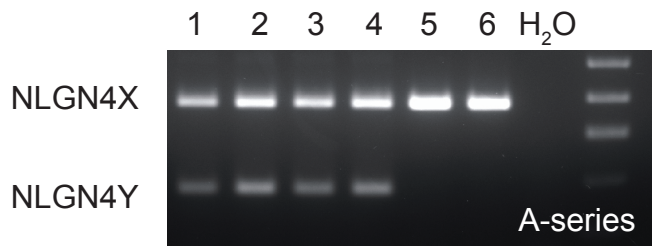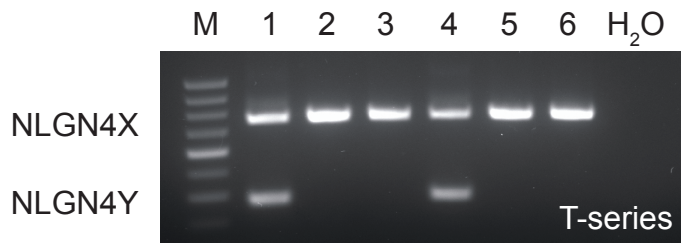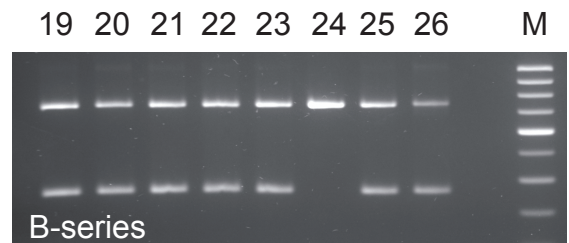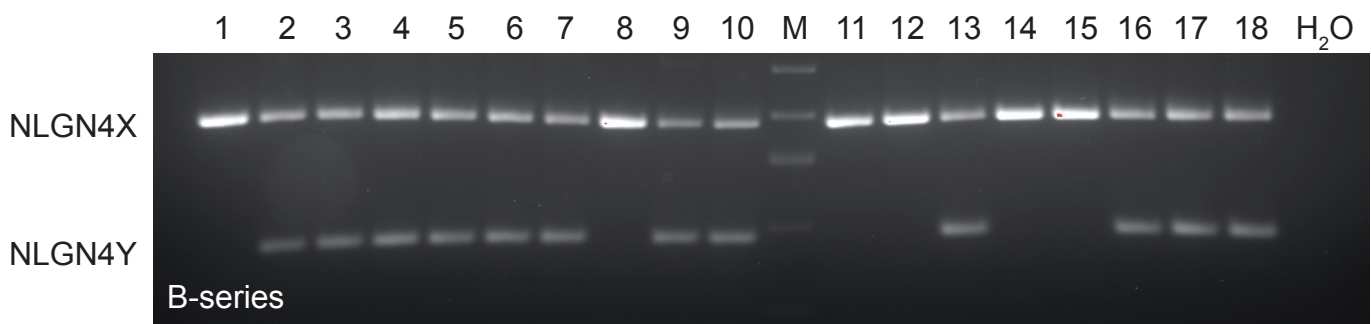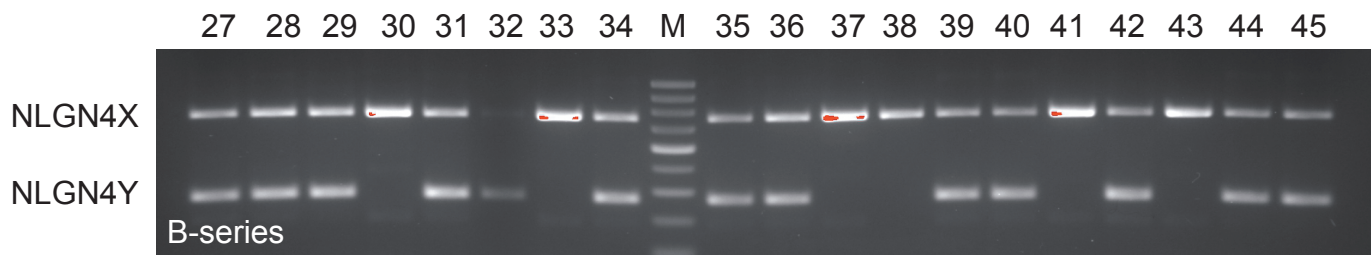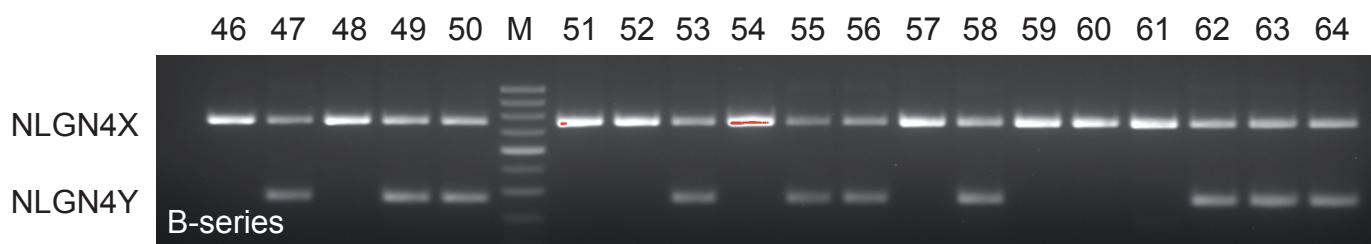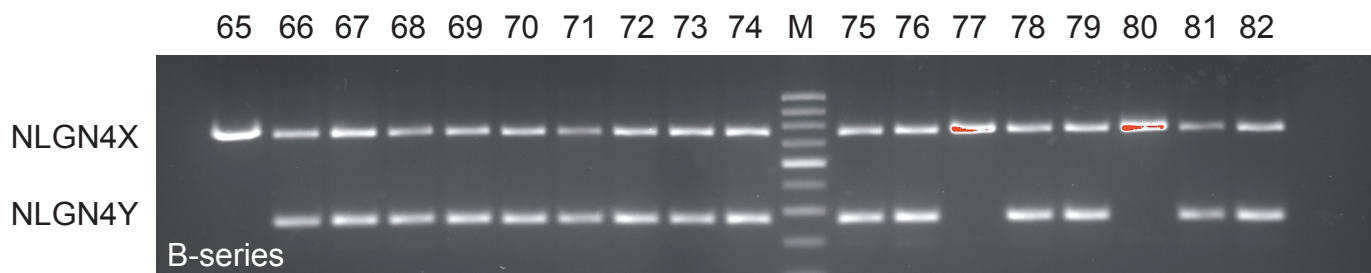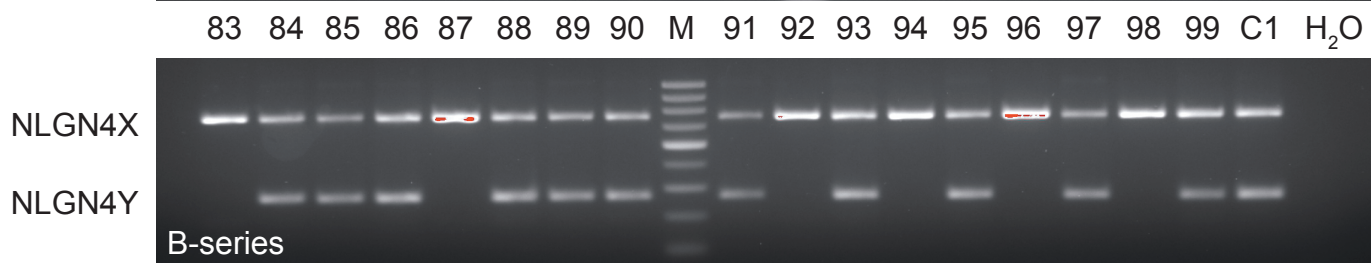

Supplement: Supplementary file 2 — Additional file 2. PCR results of testing for the indel polymorphism [file 13293_2019_279_MOESM2_ESM.pdf]
