# Supplementary material for: Novel human sex-typing strategies based on the autism candidate gene NLGN4X and its male-specific gametologue NLGN4Y
Source: Biol Sex Differ. 2019 Dec 18;10:62. doi: 10.1186/s13293-019-0279-x (PMC6921425; doi:10.1186/s13293-019-0279-x)
Supplement: Supplementary file 4 — Additional file 4. Alignment for SNP_C [file 13293_2019_279_MOESM4_ESM.pdf]

rhAmp™ SNP Sex-typing
Alignment shows *NLGN4X/Y* exon 7

```
           1                                                                                                                               130
   NLGN4X  TGATCCAAATCAACCAGTTCCTCAGGATACCAAGTTCATTCACACAAAACCCAACCGCTTTGAAGAAGTGGCCTGGTCCAAGTATAATCCCAAAGACCAGCTCTATCTGCATATTGGCTTGAAACCCAGA
   NLGN4Y  TGATCCAAACCAACCAGTTCCTCAGGATACCAAGTTCATTCATACAAAACCCAATCGCTTTGAAGAAGTGGCCTGGTCCAAGTATAATCCCAAAGACCAGCTCTATCTGCATATTGGCTTGAAACCCAGA
Consensus  TGATCCAAAcCAACCAGTTCCTCAGGATACCAAGTTCATTCAcACAAAACCCAAcCGCTTTGAAGAAGTGGCCTGGTCCAAGTATAATCCCAAAGACCAGCTCTATCTGCATATTGGCTTGAAACCCAGA

           131                                                                                                                             260
   NLGN4X  GTGAGAGATCACTACCGGGCAACGAAAGTGGCTTTCTGGTTGGAACTCGTTCCTCATTTGCACAACTTGAACGAGATATTCCAGTATGTTTCAACAACCACAAAGGTTCCTCCACCAGACATGACATCAT
   NLGN4Y  GTGAGAGATCACTACCGGGCAACGAAAGTGGCTTTCTGGTTGGAATTGGTTCCTCATTTGCACAACTTGAACGAGATATTCCAGTATGTTTCAACAACCACAAAGGTTCCTCCACCAGACATGACATCAT
Consensus  GTGAGAGATCACTACCGGGCAACGAAAGTGGCTTTCTGGTTGGAAcTcGTTCCTCATTTGCACAACTTGAACGAGATATTCCAGTATGTTTCAACAACCACAAAGGTTCCTCCACCAGACATGACATCAT

           261                                                                                                                             390
   NLGN4X  TTCCCTATGGCACCCGGCGATCTCCCGCCAAGATATGGCCAACCACCAAACGCCCAGCAATCACTCCTGCCAACAATCCCAAACACTCTAAGGACCCTCACAAAACAGGGCCTGAGGACACAACTGTCCT
   NLGN4Y  TTCCCTATGGCACCCGGCGATCTCCCGCCAAGATATGGCCAACCACCAAACGCCCAGCAATCACTCCTGCCAACAATCCCAAACACTCTAAGGACCCTCACAAAACAGGGCCCGAGGACACAACTGTCCT
Consensus  TTCCCTATGGCACCCGGCGATCTCCCGCCAAGATATGGCCAACCACCAAACGCCCAGCAATCACTCCTGCCAACAATCCCAAACACTCTAAGGACCCTCACAAAACAGGGCCcGAGGACACAACTGTCCT

           391                                                                                                                             520
   NLGN4X  CATTGAAACCAAACGAGATTATTCCACCGAATTAAGTGTCACCATTGCCGTCGGGGCGTCGCTCCTCTTCCTCAACATCTTAGCTTTTGCGGCGCTGTACTACAAAAAGGACAAGAGGCGCCATGAGACT
   NLGN4Y  CATTGAAACCAAACGAGATTATTCCACCGAATTAAGTGTCACCATTGCCGTCGGGGCGTCGCTCCTCTTCCTCAACATCTTAGCCTTTGCGGCGCTGTACTACAAAAAGGACAAGAGGCGCCATGAGACT
Consensus  CATTGAAACCAAACGAGATTATTCCACCGAATTAAGTGTCACCATTGCCGTCGGGGCGTCGCTCCTCTTCCTCAACATCTTAGCcTTTGCGGCGCTGTACTACAAAAAGGACAAGAGGCGCCATGAGACT
```

SNP_C assay

```
           521                                                                                                                             650
   NLGN4X  CACAGGCGCCCCAGTCCCCAGAGAAACACCACAAATGATATCGCTCACATCCAGAACGAAGAGATCATGTCTCTGCAGATGAAGCAGCTGGAACACGATCACGAGTGTGAGTCGCTGCAGGCACACGACA
   NLGN4Y  CACAGGCACCCCAGTCCCCAGAGAAACACCACAAATGATATCACTCACATCCAGAACGAAGAGATCATGTCTCTGCAGATGAAGCAGCTGGAACACGATCACGAGTGTGAGTCGCTGCAGGCACACGACA
Consensus  CACAGGCaCCCCAGTCCCCAGAGAAACACCACAAATGATATCaCTCACATCCAGAACGAAGAGATCATGTCTCTGCAGATGAAGCAGCTGGAACACGATCACGAGTGTGAGTCGCTGCAGGCACACGACA

           651                                                                                                                             780
   NLGN4X  CACTGAGGCTCACCTGCCCGCCAGACTACACCCTCACGCTGCGCCGGTCGCCAGATGACATCCCACTTATGACGCCAAACACCATCACCATGATTCCAAACACACTGACGGGGATGCAGCCTTTGCACAC
   NLGN4Y  CGCTGAGGCTCACCTGCCCTCCAGACTACACCCTCACGCTGCGCCGGTCGCCGGATGACATCCCATTTATGACGCCAAACACCATCACCATGATTCCAAACACATTGATGGGGATGCAGCCTTTACACAC
Consensus  CaCTGAGGCTCACCTGCCCgCCAGACTACACCCTCACGCTGCGCCGGTCGCCaGATGACATCCCAcTTATGACGCCAAACACCATCACCATGATTCCAAACACAcTGAcGGGGATGCAGCCTTTaCACAC

           781                                                                 850
   NLGN4X  TTTTAACACCTTCAGTGGAGGACAAAACAGTACAAATTTACCCCACGGACATTCCACCACTAGAGTATAG
   NLGN4Y  TTTTAAAACCTTCAGTGGAGGACAAAACAGTACAAATTTACCCCACGGACATTCCACCACTAGAGTATAG
Consensus  TTTTAAaACCTTCAGTGGAGGACAAAACAGTACAAATTTACCCCACGGACATTCCACCACTAGAGTATAG
```
